# An ERK1/2‐driven RNA‐binding switch in nucleolin drives ribosome biogenesis and pancreatic tumorigenesis downstream of RAS oncogene

**DOI:** 10.15252/embj.2022110902

**Published:** 2023-04-11

**Authors:** Muhammad S Azman, Emilie L Alard, Martin Dodel, Federica Capraro, Rupert Faraway, Maria Dermit, Wanling Fan, Alina Chakraborty, Jernej Ule, Faraz K Mardakheh

**Affiliations:** ^1^ Centre for Cancer Cell and Molecular Biology, Barts Cancer Institute Queen Mary University of London London UK; ^2^ Randall Centre for Cell and Molecular Biophysics King's College London London UK; ^3^ The Francis Crick Institute London UK; ^4^ Dementia Research Institute King's College London London UK

**Keywords:** Nucleolin, pancreatic ductal adenocarcinoma, RAS, ribosome biogenesis, RNA‐binding proteins, RNA Biology, Translation & Protein Quality

## Abstract

Oncogenic RAS signaling reprograms gene expression through both transcriptional and post‐transcriptional mechanisms. While transcriptional regulation downstream of RAS is relatively well characterized, how RAS post‐transcriptionally modulates gene expression to promote malignancy remains largely unclear. Using quantitative RNA interactome capture analysis, we here reveal that oncogenic RAS signaling reshapes the RNA‐bound proteomic landscape of pancreatic cancer cells, with a network of nuclear proteins centered around nucleolin displaying enhanced RNA‐binding activity. We show that nucleolin is phosphorylated downstream of RAS, which increases its binding to pre‐ribosomal RNA (rRNA), boosts rRNA production, and promotes ribosome biogenesis. This nucleolin‐dependent enhancement of ribosome biogenesis is crucial for RAS‐induced pancreatic cancer cell proliferation and can be targeted therapeutically to inhibit tumor growth. Our results reveal that oncogenic RAS signaling drives ribosome biogenesis by regulating the RNA‐binding activity of nucleolin and highlight a crucial role for this mechanism in RAS‐mediated tumorigenesis.

## Graphical abstract

Oncogenic RAS‐induced ERK1/2 activity promotes the phosphorylation and RNA‐binding activity of nucleolin, boosting protein synthesis, and pancreatic cancer.

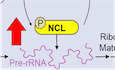

## Introduction

*RAS* genes are among the most mutated proto‐oncogenes in human cancers, with around 20% of all malignancies estimated to harbor oncogenic mutations in one of the three highly homologous *KRAS*, *NRAS*, or *HRAS* genes (Prior *et al*, 2020). The encoded RAS proteins are small GTPases which function as molecular switches that regulate several downstream kinase signaling pathways, including ERK1/2 (also known as the RAS‐MAPK cascade), and PI3K (Malumbres & Barbacid, 2003). Oncogenic mutations in RAS result in constitutive activation of these downstream kinase signaling pathways, which phosphorylate a plethora of cellular substrates, including a number of transcription factors, ultimately resulting in significant changes in the gene expression profile that promote various aspects of malignancy (Pylayeva‐Gupta *et al*, 2011). Although this transcriptional regulation has been shown to play a key role in RAS tumorigenesis, recent studies have revealed that a significant degree of gene expression dysregulation that occurs in cancers is post‐transcriptional (Nusinow *et al*, 2020). However, how RAS signaling post‐transcriptionally modulates gene expression remains largely unclear.

RNA‐binding proteins (RBPs) are the main post‐transcriptional regulators of gene expression, controlling all aspects of the RNA life‐cycle from synthesis to degradation (Gerstberger *et al*, 2014). Numerous studies have revealed a key role for many RBPs in cancer development and progression (Kang *et al*, 2020). These RBPs coordinate diverse aspects of post‐transcriptional regulation, including splicing (Fish *et al*, 2021), post‐transcriptional modifications (Barbieri *et al*, 2017), transport (Dermit *et al*, 2020), translation (Truitt *et al*, 2015), and turnover of various types of RNA (Yu *et al*, 2020). The recent advent of RNA interactome capture (RIC) methods, which allow global unbiased identification of proteins that are directly bound by RNA *in vivo*, has transformed our understanding of RBPs (Baltz *et al*, 2012; Castello *et al*, 2012; Queiroz *et al*, 2019; Trendel *et al*, 2019). RIC studies have significantly expanded the catalog of known RBPs, with around 10% of the human proteome having been demonstrated to bind RNA. These include not only “conventional” RBPs, which contain at least one classical globular RNA‐binding Domain (RBD), but also “non‐conventional” RBPs that lack any apparent RBDs (Hentze *et al*, 2018). When combined with quantitative proteomics, RIC can also be used for quantifying changes in the RNA‐bound proteome (RBPome), thus allowing systematic study of RNA‐binding dynamics (Sysoev *et al*, 2016; Garcia‐Moreno *et al*, 2019). However, a comprehensive understanding of how oncogenic signaling pathways dynamically modulate the RBPome is still lacking.

Here, we devised a quantitative whole‐transcriptome RIC approach, in order to define the impact of oncogenic RAS signaling on the RBPome of mouse pancreatic ductal adenocarcinoma (PDAC) cells. We focused on PDAC, as nearly all its cases harbor oncogenic *KRAS* mutations, highlighting a key role for RAS in the etiology of the disease (Waters & Der, 2018). Our results reveal that through activation of downstream Erk1/2 signaling, oncogenic Kras extensively remodels the RBPome of PDAC cells, with various conventional RBPs exhibiting enhanced RNA association, while nonconventional RBPs dissociate from RNA. Although many of the observed alterations in the RBPome are due to changes in the expression of RBPs, some RBPs show modulations in their RNA‐binding activity. Specifically, a network of conventional RBPs that includes the nucleolar protein nucleolin (Ncl) exhibits a significant enhancement in their RNA‐binding activity upon oncogenic Kras induction. Using quantitative phospho‐proteomics, we reveal that several of these RBPs, including Ncl, are phosphorylated downstream of Erk1/2. Phosphorylation of Ncl acts to enhance its binding to preribosomal‐RNA (pre‐rRNA), which in turn boosts rRNA synthesis and promotes ribosome biogenesis downstream of oncogenic Kras. Crucially, we demonstrate that the enhancement of ribosome biogenesis by Ncl is essential for oncogenic Kras‐induced PDAC cell proliferation and tumorigenesis, and can be targeted therapeutically to inhibit PDAC growth, *in vivo*. Our findings reveal a switch in the RNA‐binding activity of Ncl, triggered by phosphorylation downstream of Erk1/2, which governs rRNA synthesis and ribosome biogenesis, and demonstrate a key targetable role for this process in RAS‐mediated tumors.

## Results

### Oncogenic Kras reshapes the RNA‐binding landscape of mouse PDAC cells

**Figure 1:**
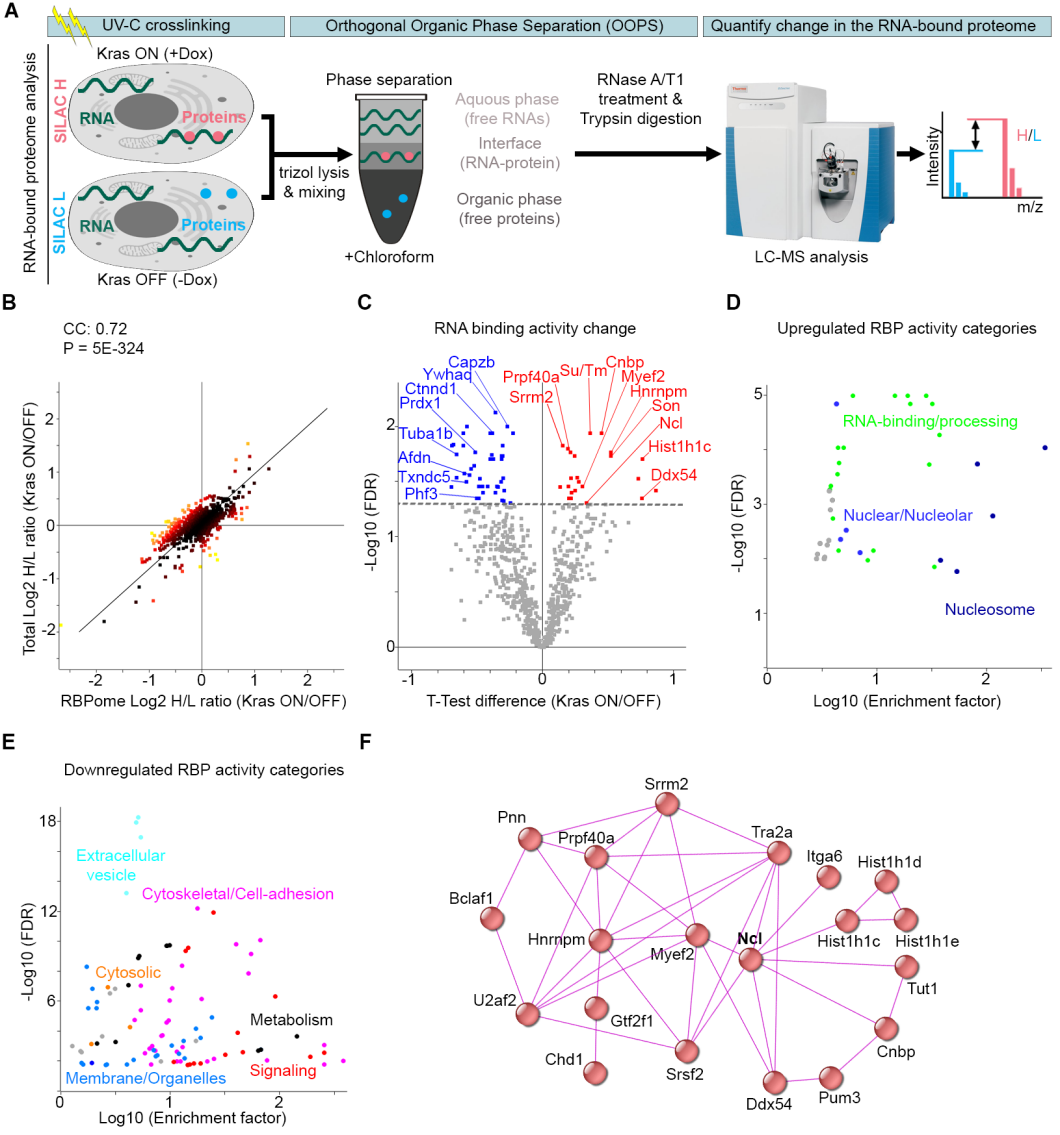
Kras^G12D^ reshapes the RNA‐binding activity landscape of PDAC cells Experimental workflow for qRIC analysis. Heavy (H) and Light (L) SILAC labeled iKras cells, with or without Dox to induce Kras^G12D^ expression, were subjected to UV‐C cross‐linking, TRIzol lysis, mixing of differentially labeled conditions, and OOPS analysis. RNA‐cross‐linked proteins, which separate into the interface, were then extracted and subjected to Trypsin digestion and liquid chromatography coupled with mass spectrometry (LC–MS) analysis. The SILAC (H/L) ratio values revealed Kras^G12D^‐regulated changes in the RBPome.Changes in the RBPome of iKras PDAC cells in response to Kras^G12D^ induction are primarily driven by changes in protein expression. SILAC Total proteome changes following Kras^G12D^ induction were measured and plotted against SILAC RBPome changes from Fig EV1F. Pearson's Correlation Coefficient (CC) and significance of correlation (*P*) between the total proteome and the RBPome changes were calculated and displayed above the graph.Volcano plot of changes in the RNA‐binding activity following Kras^G12D^ induction. RBPome ratio changes were normalized to total proteome ratio changes to calculate changes in the RNA‐binding activity (Dataset EV5), using a one‐sample *t*‐test analysis (FDR < 0.05). A total of six biological replicate experiments were performed. Twenty‐three proteins showed a significant increase in their RNA‐binding activity (red), while 42 exhibited a significant decrease (blue).Fisher's exact test analysis of categories that are over‐represented among the proteins with enhanced RNA‐binding activity (FDR < 0.02). Each data point represents a category from Gene Ontology (GO) and Kyoto Encyclopedia of Genes and Genomes (KEGG) databases, with functionally similar categories highlighted with the same colors (Dataset EV6).Fisher's exact test analysis of categories that are over‐represented among the proteins with diminished RNA‐binding activity (FDR < 0.02). Each data point represents a category from GO and KEGG databases, with functionally similar categories highlighted with the same colors (Dataset EV7).Interaction network analysis of proteins with enhanced RNA‐binding activity, using the STRING physical interactions database (Franceschini *et al*, 2013).

**Figure EV1:**
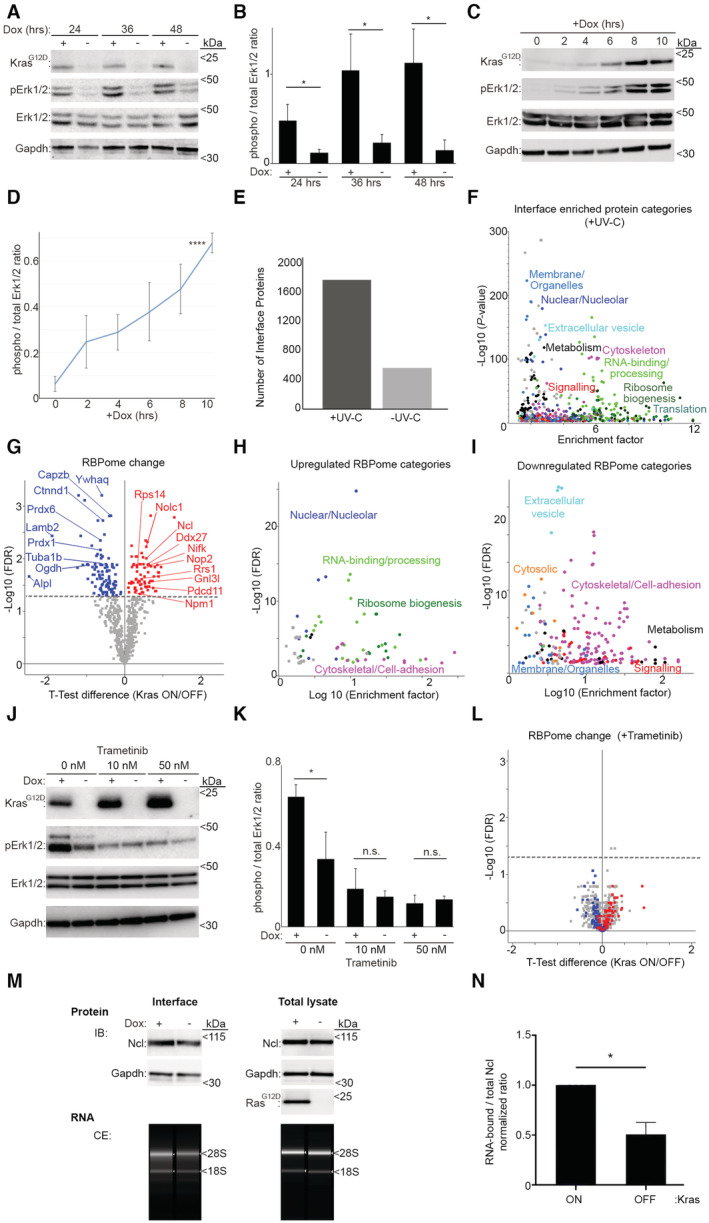
Kras^G12D^ reshapes the RBpome of PDAC cells Dox removal results in loss of Kras^G12D^ expression and ERK activity in iKras PDAC cells. Cells were grown in the presence or absence of Dox for the indicated amounts of time, before being subjected to lysis and immunoblotting (IB) with the indicated antibodies.Quantification of phospho / total Erk1/2 ratio values from (A), as a measure of Erk1/2 kinase activity. A total of three independent biological replicate experiments were quantified. Error bars depict SD (*P < 0.05—calculated from unpaired *t*‐test).Addition of Dox to Dox‐withdrawn cells results in induction of Kras^G12D^ expression and ERK activity in iKras PDAC cells. Cells were grown in the absence of Dox for 48 h, before its addition for the indicated amounts of time. Cells were then subjected to lysis and immunoblotting with the indicated antibodies.Quantification of phospho / total Erk1/2 ratio values from (C), as a measure of Erk1/2 kinase activity. A total of three independent biological replicate time course experiments were quantified (****P < 0.0001—calculated from one‐way ANOVA).OOPS‐mediated enrichment of proteins in the interface is dependent on UV‐C cross‐linking. IKras PDAC cells were treated with or without UV‐C cross‐linking, before lysis in TRIzol and OOPS analysis as in (Queiroz *et al*, 2019). Interface proteins were then extracted and subjected to mass spectrometry analysis. A total of two biological replicates per condition were analyzed, and the total number of proteins identified in both replicates for each condition were plotted. Enrichment of proteins in the interface was boosted by > 400% upon UV‐C cross‐linking.OOPS specifically enriches RNA‐binding proteins in the interface, following UV‐C cross‐linking. Fisher's exact test analysis of enriched protein categories in the interface of UV‐C cross‐linked samples from (E) (FDR < 0.02). Each data point represents a category from Gene Ontology (GO) and Kyoto Encyclopedia of Genes & Genome (KEGG) databases, with functionally similar categories highlighted with the same colors. Conventional as well as nonconventional RBPs are significantly enriched in the interface of UV‐C cross‐linked samples.Volcano plot of changes in the RBPome following Kras^G12D^ induction. RBPome changes were quantified, as described in Fig 1A, from six independent biological replicate qRIC experiments (Dataset EV1), using a one‐sample *t*‐test analysis. Seventy‐four proteins were upregulated in the RNA‐bound fraction (red), while 109 showed a significant decrease (blue) (FDR < 0.05).Fisher's exact test analysis of protein categories that are over‐represented among the upregulated RBPome (FDR < 0.02). Each data point represents a category from GO and KEGG databases, with functionally similar categories highlighted with the same colors (Dataset EV2).Fisher's exact test analysis of protein categories that are over‐represented among the downregulated RBPome (FDR < 0.02). Each data point represents a category from GO and KEGG databases, with functionally similar categories highlighted with the same colors (Dataset EV3).Trametinib inhibits Kras^G12D^‐induced ERK activity in iKras PDAC cells. Cells were grown in the absence of Dox for 48 h, before its addition to the indicated cells for 24 h, with or without 10 or 50 nM Trametinib. Cells were then subjected to lysis and immunoblotting with the indicated antibodies.Quantification of phospho / total Erk1/2 ratio values from (J), as a measure of Erk1/2 kinase activity. A total of three independent biological replicate experiments were quantified. Error bars depict SD (*P < 0.05; n.s.: not significant—calculated from unpaired *t*‐test).Volcano plot of Kras^G12D^‐driven changes in the RBPome in the presence of Trametinib (10 nM). RBPome changes in the presence of Trametinib were quantified from four independent qRIC experiments, using a one‐sample *t*‐test analysis (FDR < 0.05), with significantly increased (red) or decreased (blue) proteins from (G) highlighted on the plot (Dataset EV4).Analysis of the Kras^G12D^‐induced change in the RNA‐binding activity of endogenous Ncl. IKras PDAC cells were grown in the absence of Dox for 48 h, before its addition to the indicated cells for a further 24 h to induce Kras^G12D^ expression. Cells were then subjected to OOPS in order to isolate the interface (RNA‐bound proteins), or whole cell lysis (total lysate), followed by immunoblotting with anti‐Ncl antibody. For comparison, Gapdh, which also binds RNA but does not show a change in its RNA‐binding activity (Dataset EV1), was also blotted for. In parallel, a fraction of each interface or total lysate sample was subjected to RNA extraction, which was resolved and quantified by capillary electrophoresis (CE) as loading control.Quantification of normalized RNA‐bound to total Ncl ratio values from (M), as a measure its RNA‐binding activity. A total of three biological replicate experiments were quantified. Error bars depict SD (*P < 0.05—calculated from unpaired *t*‐test).

To study oncogenic Kras signaling, we employed tumor cells from an inducible mouse model of PDAC (iKras), in which oncogenic Kras^G12D^ expression can be controlled by the administration of Doxycycline (Dox) (Ying *et al*, 2012). We confirmed that removal of Dox and the consequent loss of Kras^G12D^ expression resulted in the downregulation of Erk1/2 signaling in this model (Fig EV1A and B). Conversely, addition of Dox to Dox‐withdrawn cells induced Kras^G12D^ expression and Erk1/2 activation (Fig EV1C and D). To study the RBPome, we employed orthogonal organic phase separation (OOPS), a whole‐transcriptome RIC method which uses UV‐C cross‐linking coupled with phenol‐chloroform based phase separation to purify *in vivo* cross‐linked RNA–protein moieties (Queiroz *et al*, 2019). We validated that OOPS specifically enriched for cross‐linked RBPs (Fig EV1E and F). We then devised a quantitative RIC (qRIC) approach by combining OOPS with stable isotope labeling by amino acids in culture (SILAC; Ong & Mann, 2006), allowing us to measure changes in the RBPome upon induction of Kras^G12D^ expression (Fig 1A). Inducing Kras^G12D^ expression resulted in a significant increase in the RNA‐bound levels of 74 proteins, while 109 proteins showed a significant decrease (Fig EV1G and Dataset EV1). Category enrichment analysis revealed that the majority of increased RNA‐bound proteins were conventional RBPs, belonging to various RBP families involved in nuclear RNA processing and ribosome biogenesis (Fig EV1H and Dataset EV2). In contrast, the majority of decreased RNA‐bound proteins were nonconventional RBPs such as metabolic enzymes and cytoskeletal proteins (Fig EV1I and Dataset EV3). Importantly, the effect of Kras^G12D^ on the RBPome was largely abrogated upon treating the cells with Trametinib, a specific MEK1/2 kinase inhibitor which blocks Erk1/2 activation downstream of Kras^G12D^ (Wright & McCormack, 2013), suggesting that the observed changes are largely dependent on Erk1/2 signaling (Fig EV1J–L and Dataset EV4).

Next, we investigated the mechanisms of RBPome modulation in response to induction of Kras^G12D^ expression. Dysregulation of RBPs in cancer is primarily thought to be brought about by changes in their expression (Pereira *et al*, 2017). However, signaling pathways have also been shown to modulate the activity of some RBPs through post‐translational modifications (Matter *et al*, 2002; Tripathi *et al*, 2010; Hong *et al*, 2017). To assess these two possibilities, we performed SILAC‐based total proteomics on the iKras PDAC cells, with or without the induction of Kras^G12D^ expression. We observed a strong overall correlation between changes in the RBPome and the total proteome (Fig 1B), suggesting that the majority of variation in the RBPome is likely reflective of changes in the expression levels of RBPs. However, not all changes were correlative, so to specifically reveal alterations in the RBPome that were independent of protein expression modulations, we normalized the RBPome SILAC ratio values to those of the total proteome. Analysis of this RBPome to total normalized ratio value revealed changes in the RNA‐binding activities of RBPs in response to Kras^G12D^ induction. We observed a significant increase in the RNA‐binding activity of 23 proteins, coupled with a significant decrease in the activity of 42 proteins (Fig 1C and Dataset EV5). Similar to the RBPome changes, the majority of proteins with increased RNA‐binding activity were conventional RBPs belonging to various nuclear protein families (Fig 1C and D, and Dataset EV6), while the majority of proteins with decreased RNA‐binding activity were nonconventional RBPs belonging mostly to metabolic and cytoskeletal protein families (Fig 1C and E, and Dataset EV7). Interactome analysis of the RBPs with increased RNA‐binding activity revealed that 20 out of the 23 of them are known to physically associate with one another (Fig 1F). We further validated the increase in the RNA‐binding activity of one of these RBPs, Ncl, as it has been previously implicated in various cancers (Abdelmohsen & Gorospe, 2012). For this purpose, the iKras PDAC cells, with or without Kras^G12D^ induction, were subjected to either OOPS analysis or whole cell lysis. The interface fraction from OOPS that contain RNA–protein adducts, along with the total lysate, were analyzed by western blotting with an anti‐Ncl antibody. In parallel, a portion of each interface as well as total lysate sample was subjected to RNA purification to assess the total amount of RNA as loading control. A significant increase in the interface to total ratio of Ncl, indicative of its enhanced RNA‐binding activity, was evident upon Kras^G12D^ induction (Fig EV1M and N). Together, these results reveal that Kras^G12D^‐mediated Erk1/2 activation reshapes the RBPome landscape of PDAC cells, with a network of nuclear RBPs including Ncl exhibiting a significant enhancement in their RNA‐binding activity.

### Oncogenic Kras signaling enhances the RNA‐binding activity of Ncl through phosphorylation

**Figure 2:**
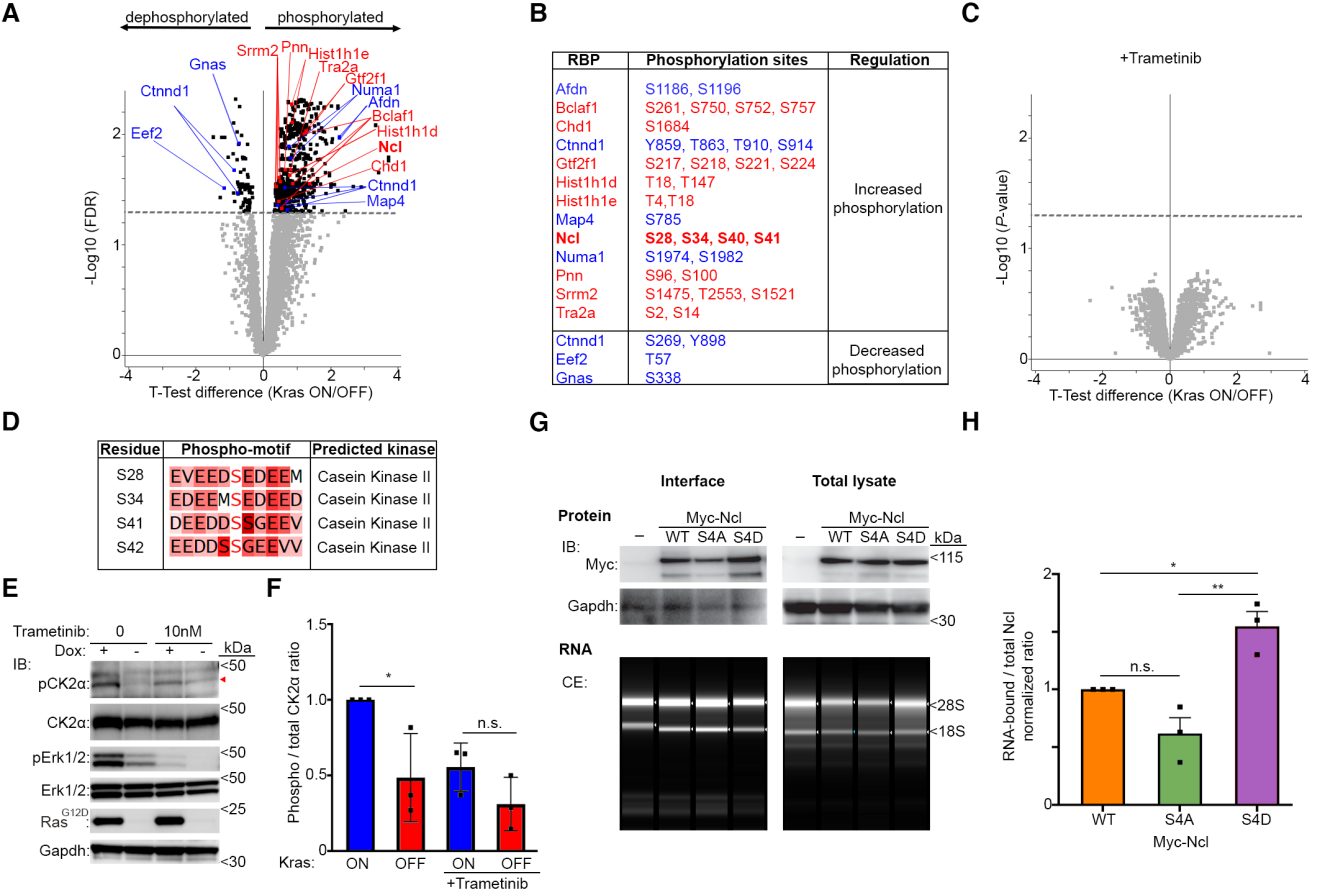
RNA‐binding activity of Ncl is enhanced upon its phosphorylation downstream of Kras^G12D^‐induced Erk1/2 signaling Volcano plot of phosphorylation changes in iKras PDAC cells in response to Kras^G12D^ induction. Phospho‐ to total proteomic changes were quantified from three independent biological replicates of Dox‐treated vs. untreated cells, via TMT (Dataset EV8). Eight hundred seventy two phosphorylations were found to be significantly increased, while 82 were significantly decreased (FDR < 0.05). Significantly changing phosphorylations on RBPs whose RNA‐binding activity was significantly increased (red) or decreased (blue) as per Fig 1C are highlighted on the plot.List of significantly increased or decreased phosphorylation sites on the RBPs whose RNA‐binding activity was enhanced (red) or reduced (blue) upon Kras^G12D^ induction.Volcano plot of phosphorylation changes in iKras PDAC cells in response to Kras^G12D^ induction in the presence of Trametinib (10 nM). Phospho‐ to total proteomic changes were quantified from three independent biological replicates of Dox + Trametinib‐treated vs. untreated cells (Dataset EV8). No phosphorylations were found to be significantly changing upon Kras^G12D^ induction in the presence of Trametinib (FDR < 0.05).Phospho‐motif kinase prediction analysis of Kras^G12D^‐induced Ncl phosphorylation sites, using NetworKIN.Kras^G12D^ induction enhances phosphorylation of CK2α (T360/S362), in an Erk1/2‐dependent manner. IKras PDAC cells were grown in the absence of Dox for 48 h, before its addition to the indicated cells, with or without Trametinib (10 nM), for a further 24 h. Cells were then lysed and analyzed by immunoblotting (IB) with the indicated antibodies. The red triangle marks the main phospho band which overlaps with the total CK2α.Quantification of normalized phospho (T360/S362) to total CK2α levels from (E). A total of three biological replicate experiments were quantified. Error bars depict SD (*P < 0.05; n.s.: not significant—calculated from one‐way ANOVA with Šídák's multiple comparisons test).Analysis of the RNA‐binding activity of WT, Phospho‐defective (S4A), and phospho‐mimicking (S4D) mutants of Ncl by OOPS. IKras PDAC cells were transiently transfected with myc‐tagged WT, S4A, and S4D Ncl constructs. Cells were subsequently grown in the absence of Dox for 48 h, followed by Dox addition for 24 h to induce Kras^G12D^ expression. Cells were then subjected to OOPS to isolate the interface (RNA‐bound proteins), or whole cell lysis (total lysate), followed by immunoblotting with anti‐Ncl antibody. For comparison, Gapdh, which also binds RNA but does not show a change in its RNA‐binding activity (Dataset EV1), was also blotted for. A fraction of each interface or total lysate sample was also subjected to RNA extraction, which was resolved and quantified by capillary electrophoresis (CE) as loading control.Quantification of normalized RNA‐bound to total myc‐Ncl ratio values from (G), as a measure of their RNA‐binding activity. A total of three biological replicate experiments were quantified. Error bars depict SD (**P < 0.01; *P < 0.05; n.s.: not significant—calculated from one‐way ANOVA with Šídák's multiple comparisons test). Source data are available online for this figure.

**Figure EV2:**
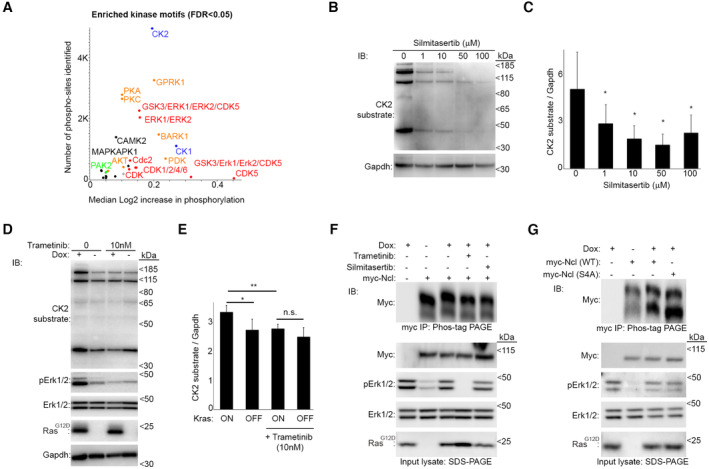
Kras^G12D^ activates CK2 to phosphorylate Ncl Motif analysis of phosphorylation changes in iKras PDAC cells in response to Kras^G12D^ induction. Phospho to total proteomic changes from Fig 2A were annotated for different kinase target linear phospho‐motifs in Perseus (Tyanova *et al*, 2016b), and subjected to 1D annotation enrichment test (FDR < 0.02). Kras^G12D^‐induced median shift in the phosphorylation intensity was plotted against the total number of the sites for each indicated motif (Dataset EV9). Colors indicate the major kinase family groups (red: Proline‐directed kinases; blue: Casein kinases; orange: AGC kinases; black: Ca^2+^/Calmodulin‐dependent kinases; green: STE kinases). In addition to the ERK1/2 substrate motif, motifs for substrates of several other kinases such as CDKs, CK1, CK2, and GPRK/β‐ARK kinases were significantly enriched among the Kras^G12D^‐induced phosphorylations.A CK2 phospho‐substrate antibody mix can be used as an indicator of CK2 activity. IKras PDAC cells grown in the presence of Dox were treated overnight with the indicated concentrations of Silmitasertib, a specific CK2 inhibitor (Chon *et al*, 2015), before being subjected to lysis and immunoblotting with the indicated antibodies.Quantification of normalized CK2 phospho‐substrate levels from (B), as a measure of CK2 kinase activity. A total of three independent biological replicate experiments were quantified. Error bars depict SD. Significance was calculated relative to untreated control (*P < 0.05—calculated from unpaired *t*‐test).Kras^G12D^ induction enhances CK2 activity in an Erk1/2‐dependent manner. IKras PDAC cells were grown in the absence of Dox for 48 h, before its addition to the indicated cells, with or without Trametinib (10 nM), for a further 24 h. Cells were then lysed and analyzed by immunoblotting with the indicated antibodies.Quantification of normalized CK2 phospho‐substrate levels from (D), as a measure of CK2 kinase activity. A total of three independent biological replicate experiments were quantified. Error bars depict SD (**P < 0.01; *P < 0.05; n.s.: not significant—calculated from unpaired *t*‐test).Kras^G12D^ induction slows the migration of myc‐Ncl through the Phos‐tag gel in an Erk1/2‐ and CK2‐dependent manner. Myc‐Ncl transfected iKras PDAC cells were grown in the absence of Dox for 48 h, before its addition to the indicated cells for a further 24 h, with or without Trametinib (10 nM) or Silmitasertib (10 μM). Cells were lysed and subjected to immunoprecipitation with anti‐Myc tag antibody, and the immunoprecipitates were resolved by Phos‐tag SDS–PAGE, followed by immunoblotting with the anti‐Myc antibody. In parallel, input lysates were resolved using standard SDS–PAGE, and immunoblotted with the indicated antibodies. The blots are representative results from three independent biological replicate experiments.Kras^G12D^‐induced retardation of myc‐Ncl migration through the Phos‐tag gel is dependent on S28, S34, S40, and S41. Control, wild‐type (WT), and phospho‐defective (S4A) mutant Myc‐Ncl transfected iKras PDAC cells were grown in the absence of Dox for 48 h, before its addition to the indicated cells for 24 h. Cells were lysed and subjected to immunoprecipitation with anti‐Myc tag antibody, and the immunoprecipitates were resolved by Phos‐tag SDS–PAGE, followed by immunoblotting with the anti‐Myc antibody. In parallel, input lysates were resolved using standard SDS–PAGE, and immunoblotted with the indicated antibodies. The blots are representative results from two independent biological replicate experiments.

We next set out to investigate the mechanism by which Erk1/2 signaling modulated the RNA‐binding activity of RBPs downstream of Kras^G12D^. Erk1/2 is a major cellular kinase that can directly or indirectly phosphorylate a plethora of downstream cellular substrates (Yoon & Seger, 2006). We therefore hypothesized that some of the changes in RNA‐binding activity could be mediated via Erk1/2‐dependent phosphorylation of RBPs. To reveal Erk1/2‐dependent phosphorylation changes that are brought about by Kras^G12D^ induction, we carried out a multivariate quantitative phospho‐proteomics analysis of iKras PDAC cells, using Tandem Mass Tagging (TMT; McAlister *et al*, 2012) (Dataset EV8). 7,478 phosphorylations were identified, 872 of which were found to be significantly increased upon Kras^G12D^ induction (Fig 2A). As expected, phospho‐motif analysis revealed that phosphorylations matching the ERK substrate motif were among the most significantly increased upon Kras^G12D^ induction, though phospho‐sites matching several other kinase motifs such as cyclin‐dependent kinases (CDKs), casein kinase I, and casein kinase II (CK2), were also increased (Fig EV2A), suggestive of their activation downstream of Kras. Crucially, several RBPs whose RNA‐binding activities were significantly enhanced upon Kras^G12D^ induction were among the phosphorylation targets of Kras^G12D^, often found to be phosphorylated on multiple residues (Fig 2A and B). The impact of Kras^G12D^ induction on the phospho‐proteome was abrogated by treating the cells with Trametinib (Fig 2C), suggesting that Erk1/2 signaling is the principal driver of phosphorylation events downstream of Kras^G12D^ in iKras PDAC cells, with other kinases likely to be activated downstream of Erk1/2. These findings are in agreement with previous work which showed cell‐autonomous Kras^G12D^ signaling in PDAC to be primarily mediated via Erk1/2, with PI3K only becoming activated through secreted signals from the PDAC stroma (Tape *et al*, 2016). Thus, we reveal that a number of RBPs, whose RNA‐binding activities are enhanced downstream of Kras^G12D^, undergo Erk1/2‐dependent phosphorylation.

Among the Kras^G12D^ regulated RBPs which were significantly phosphorylated downstream of Erk1/2, Ncl was strongly phosphorylated on four closely situated serine residues (S28, S34, S40, and S41) within its N‐terminal region (Fig 2B). This region, consisted of acidic‐rich sections interspersed with stretches of basic residues, is predicted to be largely disordered (Jumper *et al*, 2021). It is also well known to undergo phosphorylation by multiple kinases (Tajrishi *et al*, 2011), but no direct link between Ncl phosphorylation and Erk1/2 signaling has been reported. Phospho‐motif analysis using NetworKIN (Linding *et al*, 2008) revealed that the identified phosphorylation sites are not direct Erk1/2 targets, but most likely phosphorylated by CK2 (Fig 2D), which is well known to physically associate with and phosphorylate Ncl (Caizergues‐Ferrer *et al*, 1987; Li *et al*, 1996). In human cells, CK2α has been shown to be phosphorylated on T360 and S362 by ERK2, leading to its activation (Ji *et al*, 2009). Consistent with these findings, western blot analysis of iKras PDAC cells with a T360/S362 phospho‐specific CK2α antibody revealed that CK2α was phosphorylated on these residues upon Kras^G12D^ induction, but this was abrogated following Trametinib treatment (Fig 2E and F). Accordingly, by using a CK2 phospho‐substrate antibody mix, we could show that Kras^G12D^ induction increased the phosphorylation of CK2 targets downstream of Erk1/2 (Fig EV2B–E). To validate Ncl phosphorylation downstream of Kras^G12D^ via Erk1/2 and CK2, we employed Phos‐tag SDS‐polyacrylamide gel electrophoresis (Phos‐tag SDS–PAGE). Phos‐tag is a divalent metal ion‐containing molecule that binds phosphorylated amino acids with high affinity, resulting in retardation of their migration during SDS–PAGE (Kinoshita *et al*, 2009). We ectopically expressed myc‐tagged Ncl in iKras PDAC cells, purified it by immunoprecipitation with an anti‐Myc antibody, before resolving the purified protein on Phos‐tag‐containing polyacrylamide gels. As expected, Kras^G12D^ induction resulted in reduced migration of ectopically expressed myc‐Ncl, suggestive of its increased phosphorylation, but this shift was abrogated upon treatment of the cells with Trametinib, or Silmitasertib, a specific inhibitor of CK2 (Fig EV2F). Consistent with our phospho‐proteomics results, mutation of the four identified serine residues to alanine (S4A) also abrogated the Kras^G12D^‐induced retardation of myc‐Ncl migration, indicating that these four residues are likely the primary sites of Ncl phosphorylation downstream of Kras^G12D^ (Fig EV2G). Together, these findings demonstrate that Erk1/2 activation downstream of Kras^G12D^ increases the activity of CK2, leading to enhanced phosphorylation of Ncl on S28, S34, S40, and S41.

To reveal whether the observed phosphorylations and the enhancement of the RNA‐binding activity of Ncl were causally linked, we used phospho‐defective and phospho‐mimicking mutants of Ncl, in which the four identified residues were mutated to Alanine (S4A) or Aspartic acid (S4D), respectively. Myc‐tagged versions of these mutants, along with the wild‐type (WT) protein, were ectopically expressed in iKras PDAC cells, and the cells were subjected to either OOPS analysis or whole cell lysis, in the presence of Dox. The interface fractions from the OOPS, along with the total lysates, were analyzed by western blotting with an anti‐Myc antibody. As in Fig EV1M, a portion of each interface and total lysate sample was also subjected to RNA purification to assess the total RNA amounts as loading control. While no variations were observed between the levels of myc‐Ncl in the total lysate, the levels of the phospho‐mimicking (S4D) mutant exhibited a significant increase in the interface (Fig 2G and H). Conversely, the RNA‐bound levels of the phospho‐defective (S4A) mutant showed a relative decrease, albeit this was just below statistical significance (Fig 2G and H). Together, these results suggest that phosphorylation of Ncl on S28, S34, S40, and S41 acts to enhance its RNA‐binding activity downstream of Kras^G12D^.

### Transcriptome‐wide iCLIP studies reveal Ncl to be primarily associated with pre‐rRNA

**Figure EV3:**
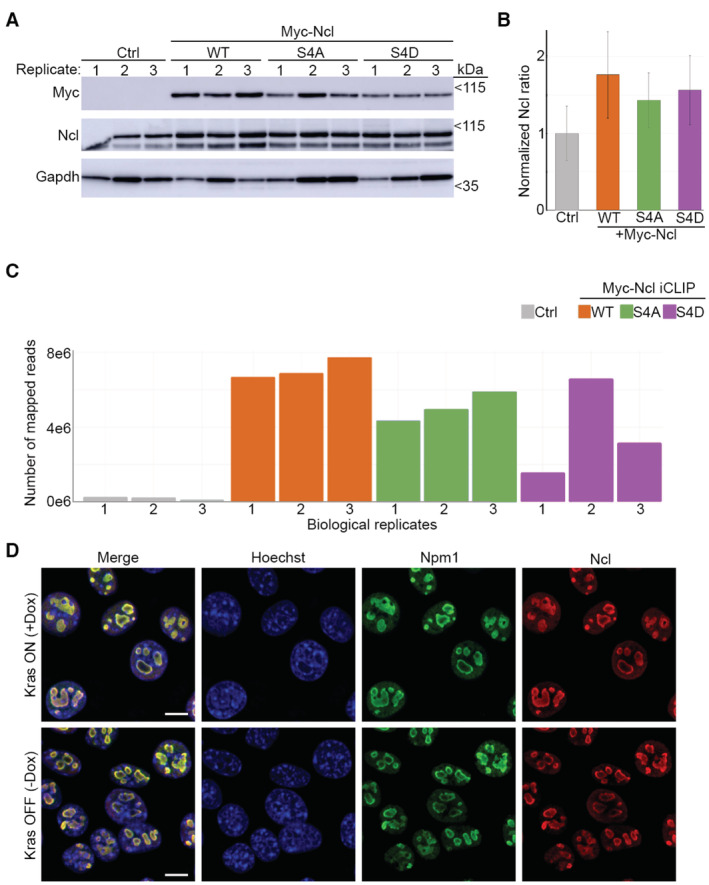
Ncl iCLIP quality‐control and localization analysis Assessment of the expression levels of endogenous and ectopic WT, S4A, and S4D Ncl, in iKras PDAC cells that were subjected to iCLIP analysis. IKras PDAC cells were transfected with constructs encoding WT, S4A, and S4D Myc‐Ncl, or mock‐transfected as negative control, before being seeded and grown for 48 h. Cells were then UV‐C irradiated, lysed, and subjected to iCLIP analysis. Aliquots of the iCLIP input lysates from three independent biological replicates were analyzed by immunoblotting with the indicated antibodies in parallel.Quantification of the relative normalized levels of Ncl antibody signal in the Ctrl vs. Myc‐Ncl expressing cells from (A). Myc‐Ncl transfected cells exhibit total Ncl levels that are around 50% more than those of the Ctrl cells. A total of three independent biological replicates per condition were analyzed.Ncl‐bound RNAs are specifically identified in Myc‐Ncl iCLIP experiments. Plot of the total number of mapped reads in each replicate of Ctrl vs. Myc‐Ncl iCLIP sequencing results. Few reads were identified in the iCLIP sequencing results of Ctrl, as opposed to Myc‐Ncl expressing cells.Endogenous Ncl is exclusively localized to the Nucleolus of iKras PDAC cells, irrespective of Kras^G12D^ expression. Cells were grown in the absence of Dox for 48 h, before its addition to the indicated cells for 24 h. Dox‐treated and untreated cells were subsequently fixed and immunostained with an anti‐Ncl antibody (red), an anti‐Npm1 antibody as a Nucleolar marker (green), and Hoechst (blue) as the Nuclear stain, followed by confocal microscopy analysis. Scale bar = 10 μm.

After revealing that the RNA‐binding activity of Ncl is enhanced by phosphorylation downstream of Kras^G12D^, we set out to determine the full spectrum of RNAs that are bound by Ncl and their dynamics in response to Kras^G12D^‐dependent phosphorylation. Although predominantly known to be localized to the nucleolus, where it regulates several steps of ribosome biogenesis, Ncl has also been shown to have diverse extra‐nucleolar functions in the nucleoplasm, cytoplasm, and the cell surface (Ugrinova *et al*, 2018). These include regulation of chromatin architecture (Erard *et al*, 1988; Angelov *et al*, 2006), microRNA processing (Pickering *et al*, 2011; Pichiorri *et al*, 2013), translation (Takagi *et al*, 2005; Abdelmohsen *et al*, 2011), as well as mRNA turnover (Sengupta *et al*, 2004; Zhang *et al*, 2006). In addition, cell‐surface‐localized Ncl has been shown to be involved in regulation of cell adhesion and transmembrane signaling (Reyes‐Reyes & Akiyama, 2008; Losfeld *et al*, 2009). In order to identify the full repertoire of RNAs that directly interact with Ncl and reveal the impact of Kras^G12D^‐dependent phosphorylation on their association with Ncl, we performed individual‐nucleotide resolution UV cross‐linking and immunoprecipitation (iCLIP) (König *et al*, 2010). Ncl–RNA complexes were purified by immunoprecipitation with an anti‐Myc‐tag antibody, from iKras PDAC cells that ectopically expressed Myc‐tagged WT, S4A, or S4D mutants of Ncl, or a mock‐transfected negative control. Western blot analysis revealed that the ectopic proteins were expressed at around 50% of the endogenous levels, ruling out potential artifacts due to high levels of overexpression (Fig EV3A and B). Moreover, little mapped iCLIP reads were identified from the negative control iCLIP samples, as opposed to the Myc‐Ncl samples, suggesting that the identification of Ncl‐interacting RNAs was highly specific (Fig EV3C).

**Figure 3:**
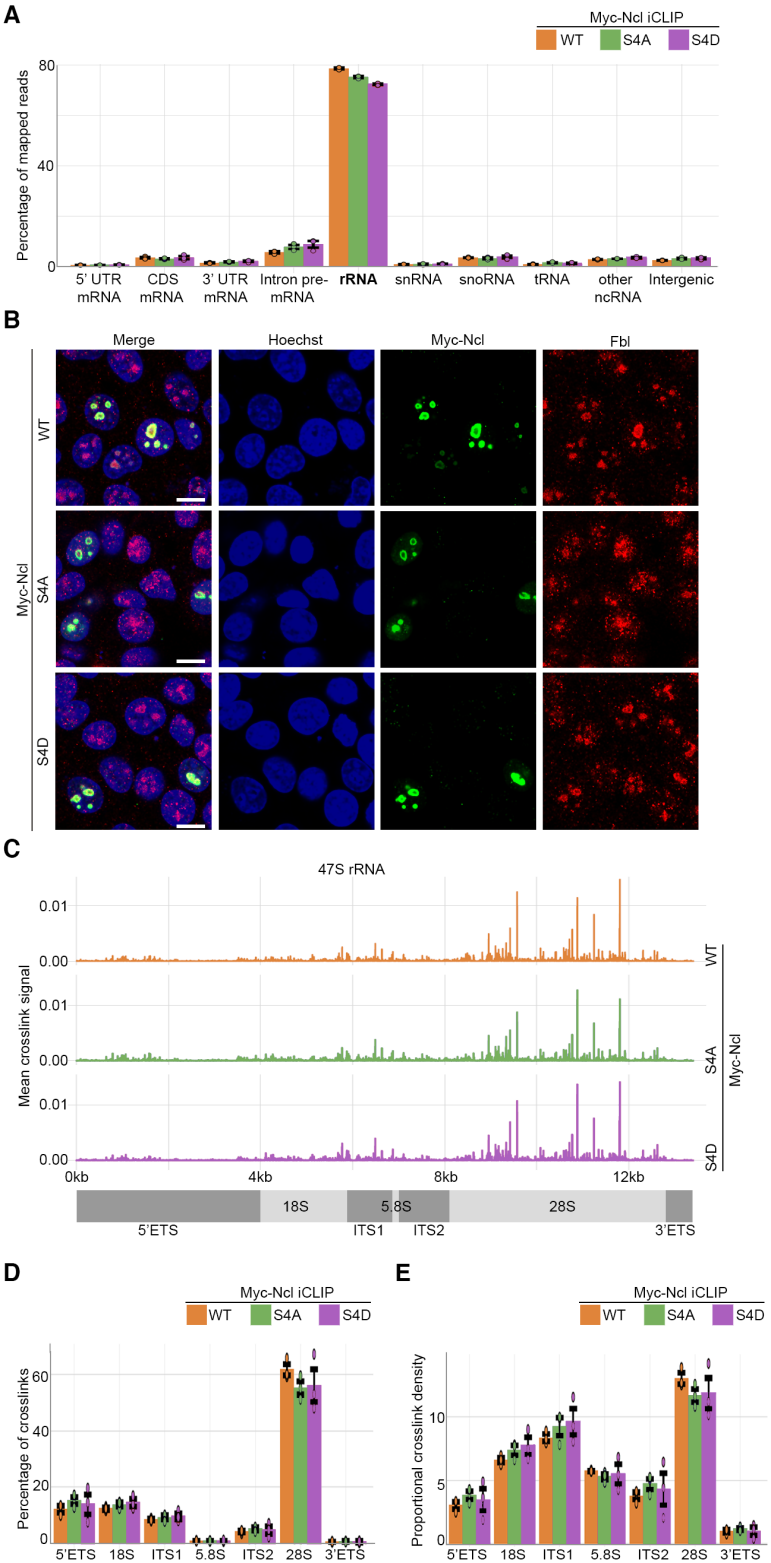
Ncl is predominantly bound to pre‐rRNA, irrespective of its phosphorylation status Percentage of mapped reads belonging to different types of RNA from three independent biological replicate iCLIP experiments of WT, S4A, and S4D Myc‐Ncl. Error bars depict SD.Immunofluorescence analysis of WT, S4A, and S4D Myc‐Ncl subcellular localization. IKras PDAC cells ectopically expressing either WT, S4A, or S4D Myc‐Ncl were fixed and immunostained with anti‐Myc‐tag antibody, along with anti‐Fibrillarin (Fbl) antibody as a Nucleolar marker, and Hoechst, followed by confocal microscopy analysis. Scale bar = 10 μm.Distribution of WT, S4A, and S4D Myc‐Ncl cross‐link sites across the annotated 47S pre‐rRNA genomic region. Peak heights represent mean cross‐link intensities from three independent iCLIP replicate experiments.Quantification of the percentage of cross‐links in each region of the 47S pre‐rRNA for WT, S4A, and S4D Myc‐Ncl. Percentage of cross‐links relative to 47S total was quantified from three independent biological replicate iCLIP experiments. Error bars depict SD.Quantification of the proportional density of cross‐links in each region of the 47S pre‐rRNA for WT, S4A, and S4D Myc‐Ncl. Proportional density was calculated from three independent iCLIP experiments by normalizing the number of cross‐links in each region to the sequence length of that region. Error bars depict SD. Source data are available online for this figure.

More than 70% of all the Myc‐Ncl iCLIP mapped reads corresponded to the ribosomal DNA (rDNA) locus, which codes for a long primary transcript known as the 47S pre‐rRNA that is ultimately processed into 18S, 5.8S, and 28S rRNAs (Fig 3A). No significant difference between WT and the phospho‐mutants was observed, suggesting that phospho‐regulation of Ncl by Kras^G12D^ does not affect its repertoire of RNA substrates (Fig 3A). In agreement with these results, immunofluorescence analysis revealed that ectopically expressed Myc‐tagged WT, S4A, and S4D mutants of Ncl were exclusively localized to the nucleolus (Fig 3B). Localization of ectopic Myc‐Ncl was in complete accordance with endogenous Ncl, which was also found to be exclusively nucleolar, irrespective of Kras^G12D^ status (Fig EV3D). Together, these results suggest that Ncl is primarily localized to the nucleolus of iKras PDAC cells where it predominantly binds pre‐rRNA, irrespective of its phosphorylation status.

Although Kras^G12D^‐dependent phosphorylation of Ncl does not affect its primary association with pre‐rRNA, the enhancement of the RNA‐binding activity could be resulting in generation of novel Ncl binding sites on the pre‐rRNA. Alternatively, Ncl could be binding to exactly the same sites, but with higher affinity. To differentiate between these possibilities, we analyzed the distribution of Ncl cross‐link sites within the pre‐rRNA. 47S pre‐rRNA contains the sequences of 18S, 5.8S, and 28S rRNAs, flanked by two external transcribed spacers at each end (5′ETS and 3′ETS), as well as two Internal transcribed spacers (ITS1 and ITS2) that are situated between 18S, 5.8S, and 28S. During rRNA processing, these spacer sequences are removed in a step‐by‐step manner via the action of different endo‐ and exo‐ribonucleases (Turowski & Tollervey, 2015). WT Ncl showed binding throughout the length of 47S pre‐rRNA, with numerous cross‐link spikes detected in various regions (Fig 3C). No difference between the cross‐linking patterns of the WT and the phospho‐mutants was observed, suggesting that phosphorylations do not change the binding pattern of Ncl on pre‐rRNA (Fig 3C). Accordingly, quantification of the percentage as well as the density of Ncl cross‐links within each pre‐rRNA region revealed no differences between WT Ncl and the phospho‐mutants (Fig 3D and E). Collectively, these results suggest that the Kras^G12D^‐dependent phosphorylations of Ncl do not affect its pattern of binding to pre‐rRNA. We therefore conclude that the Kras^G12D^‐dependent phospho‐regulation of Ncl must be acting to enhance its RNA‐binding affinity, without affecting its RNA‐binding specificity.

### Ncl phosphorylation enhances rRNA synthesis and ribosome biogenesis

**Figure 4:**
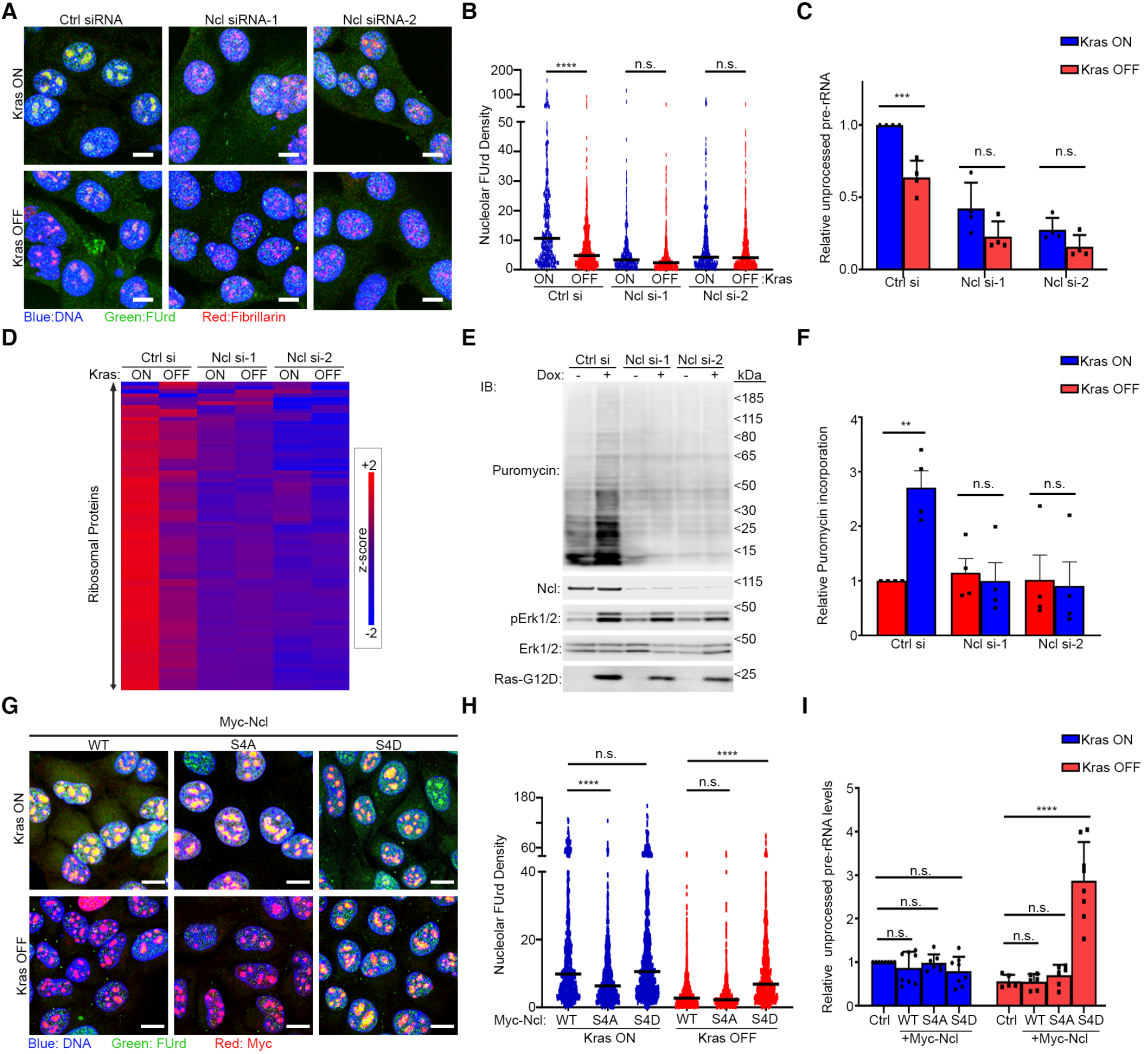
Kras^G12D^ promotes rRNA synthesis and ribosome biogenesis via Ncl Nascent RNA imaging in control and Ncl‐depleted iKras PDAC cells, in the presence or absence of Kras^G12D^. Cells were transfected with a nontargeting control siRNA, or two independent siRNAs against Ncl, grown for 48 h in the presence or absence of Dox, prior to pulse labeling with FUrd. Cells were then fixed and immunostained with anti‐FUrd antibody (green) to visualize nascent RNA, along with anti‐Fibrillarin (Fbl) antibody as a Nucleolar marker (red), and Hoechst (blue) as the Nuclear stain, followed by confocal microscopy analysis. Scale bar = 10 μm.Quantification of Nucleolar FUrd levels in images from (A). FUrd fluorescence densities in single nucleoli were quantified from 182 to 220 individual cells per condition, combined from two independent biological replicate experiments (****P < 0.0001; n.s.: not significant—calculated from one‐way ANOVA with Šídák's multiple comparisons test).RT–qPCR analysis of ITS1‐containing pre‐rRNA transcript levels in control and Ncl‐depleted iKras PDAC cells, in the presence or absence of Kras^G12D^. Cells were transfected with a nontargeting control siRNA, or two independent siRNAs against Ncl, and grown for 48 h in the presence or absence of Dox, before RT–qPCR analysis with a specific probe against the mouse ITS1 region. A probe against mouse Actb mRNA was used as loading control for normalization. A total of four biological replicate experiments were quantified (***P < 0.001; n.s.: not significant—calculated from one‐way ANOVA with Šídák's multiple comparisons test).TMT quantitative analysis of RP levels in control and Ncl‐depleted iKras PDAC cells, in the presence or absence of Kras^G12D^. Cells were transfected with a nontargeting control siRNA, or two independent siRNAs against Ncl, and grown for 48 h in the presence or absence of Dox, before lysis and TMT‐mediated quantitative mass spectrometry analysis. Z‐scores of TMT intensity changes for all identified RPs across the different conditions were plotted as a heat map (red → increase; blue → decrease).Assessment of the overall protein synthesis rates in control and Ncl‐depleted iKras PDAC cells, in the presence or absence of Kras^G12D^, by puromycinylation. Cells were transfected with a nontargeting control siRNA, or two independent siRNAs against Ncl, and grown for 48 h in the presence or absence of Dox, before pulse labeling with Puromycin (10 μg/ml) for 15 min to label nascent proteins. Cells were subsequently lysed and analyzed by immunoblotting with the indicated antibodies.Quantification of puromycin incorporation from (E), as an indicator of the overall protein synthesis rate. Total Erk1/2 levels were used as loading control. A total of four independent biological replicate experiments were quantified. Error bars depict SD (**P < 0.01; n.s.: not significant—calculated from one‐way ANOVA with Šídák's multiple comparisons test).Nascent RNA imaging of WT, S4A, and S4D Myc‐Ncl expressing iKras PDAC cells, in the presence or absence of Kras^G12D^. Vectors encoding Myc‐tagged WT, S4A, and S4D Ncl were transiently transfected into iKras PDAC cells, before reseeding and growing the cells for 48 h in the presence or absence of Dox. Cells were then subjected to pulse labeling with FUrd, fixation, and immunostaining with anti‐FUrd antibody (green), anti‐Myc‐tag antibody (red), and Hoechst (blue), followed by confocal microscopy analysis. Scale bar = 10 μm.Quantification of FUrd levels in Myc‐positive nucleoli from (E). FUrd fluorescence densities in single nucleoli were quantified from 160 to 281 individual cells per condition, combined from three independent biological replicate experiments (****P < 0.0001; n.s.: not significant—calculated from one‐way ANOVA with Šídák's multiple comparisons test).RT–qPCR analysis of ITS1‐containing pre‐rRNA transcript levels in WT, S4A, and S4D Myc‐Ncl expressing iKras PDAC cells, in the presence or absence of Kras^G12D^. Vectors encoding Myc‐tagged WT, S4A, and S4D Ncl were transiently transfected into iKras PDAC cells, before reseeding and growing the cells for 48 h in the presence or absence of Dox, followed by RT–qPCR analysis with a specific probe against the mouse ITS1 region. A probe against mouse Actb mRNA was used as loading control for normalization. A total of 5–8 biological replicate experiments per condition were quantified (****P < 0.0001; n.s.: not significant—calculated from one‐way ANOVA with Šídák's multiple comparisons test). Source data are available online for this figure.

**Figure EV4:**
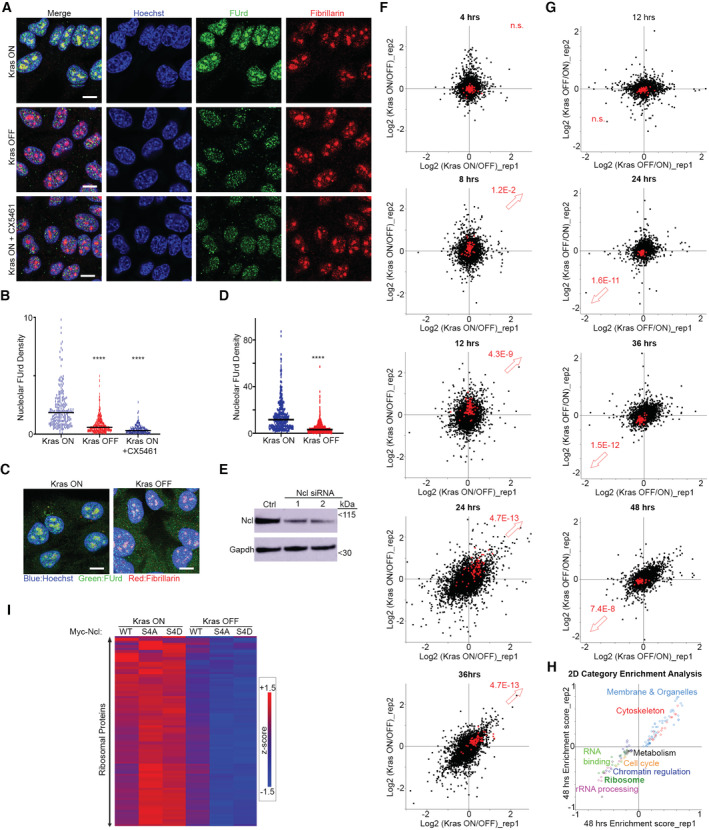
Kras^G12D^ promotes rRNA synthesis and ribosome biogenesis Induction of Kras^G12D^ expression triggers nascent pre‐rRNA synthesis in iKras PDAC cells. Cells were grown for 48 h in the absence of Dox. Kras^G12D^ expression was then induced in the indicated cells (Kras ON) by the addition of Dox for a further 24 h. The rRNA polymerase‐I inhibitor (CX‐5461) was added to the indicated cells for 30 min, before all conditions were subjected to pulse labeling with FUrd to visualize RNA synthesis. Cells were then fixed and immunostained with anti‐FUrd antibody (green) to visualize nascent RNA, along with anti‐Fibrillarin (Fbl) antibody as a Nucleolar marker (red), and Hoechst (blue) as the Nuclear stain, followed by confocal microscopy analysis. Kras^G12D^ induction results in accumulation of nascent RNA in the Nucleoli of iKras PDAC cells, in an RNA polymerase‐I‐dependent manner. Scale bar = 10 μm.Quantification of Nucleolar FUrd levels from (A). FUrd fluorescence densities in single nucleoli were quantified from 157 to 289 individual cells per condition, combined from two independent biological replicate experiments. Significance was calculated relative to the Kras ON condition (****P < 0.0001—calculated from unpaired *t*‐test).Removal of Kras^G12D^ expression results in loss of nascent pre‐rRNA synthesis in iKras PDAC cells. Cells were grown for 48 h in the presence or absence of Dox, before pulse labeling with FUrd to visualize RNA synthesis. Cells were fixed and immunostained with anti‐FUrd antibody (green) to visualize nascent RNA, along with anti‐Fibrillarin (Fbl) antibody as a Nucleolar marker (red), and Hoechst (blue) as the Nuclear stain, followed by confocal microscopy analysis. Loss of Kras^G12D^ expression results in abrogation of nascent RNA accumulation in the Nucleoli. Scale bar = 10 μm.Quantification of Nucleolar FUrd levels from (C). FUrd fluorescence densities in single nucleoli were quantified from 147 to 196 individual cells per condition, combined from two independent biological replicate experiments (****P < 0.0001—calculated from unpaired *t*‐test).Validation of siRNA‐mediated depletion of Ncl in iKras PDAC cells. Cells were transfected with a nontargeting control siRNA, or two independent siRNAs against Ncl, before being lysed and analyzed by immunoblotting with the indicated antibodies.Induction of Kras^G12^ expression results in accumulation of ribosomal proteins (RPs). IKras PDAC cells were grown in the absence of Dox for 48 h, before its addition to the cells for the indicated amounts of time (Kras ON), or leaving the cells untreated for the same period as control (Kras OFF). Cells were subsequently lysed and subjected to TMT‐mediated quantitative proteomics (Dataset EV10). Log2 of Kras ON/Kras OFF protein ratio values from two biological replicate experiments were plotted for each time point, with the ratio values of RPs marked in red. Benjamini–Hochberg‐corrected *P*‐values of the increase in RPs ratio values are reported on each graph (n.s.: not significant).Loss of Kras^G12^ expression results in depletion of RPs. IKras PDAC cells were seeded and grown in the presence (Kras ON) or absence (Kras OFF) of Dox for the indicated amounts of time, before lysis and TMT‐mediated quantitative proteomics (Dataset EV11). Log2 of Kras ON/Kras OFF protein ratio values from two biological replicate experiments were plotted for each time point, with the ratio values of RPs marked in red. Benjamini–Hochberg‐corrected *P*‐values of the decrease in RPs ratio values are reported on each graph (n.s.: not significant).2D‐annotation enrichment analysis of the 48‐h time point data from (G). Each data point represents a functional category from GO and KEGG databases, with similar categories highlighted with the same colors (Dataset EV12). After loss of Kras^G12^ for 48 h, protein categories related to Ribosome and rRNA processing exhibit significant downregulation, while those related to cytoskeleton and membranous organelles show upregulation (FDR < 0.02).TMT quantitative analysis of RP levels in WT, S4A, and S4D Myc‐Ncl expressing iKras PDAC cells, in the presence or absence of Kras^G12D^. Vectors encoding Myc‐tagged WT, S4A, and S4D Ncl were transiently transfected into iKras PDAC cells, before reseeding and growing the cells for 48 h in the presence or absence of Dox. Cells were then lysed and analyzed by TMT‐mediated quantitative mass spectrometry. *Z*‐scores of TMT intensity changes for all the identified RPs across the different conditions were plotted as a heat map (red → increase; blue → decrease).

The predominant interaction of Ncl with pre‐rRNA suggests that it must be primarily functioning by regulating ribosome biogenesis in iKras PDAC cells, so we next investigated the impact of Ncl phospho‐regulation on controlling ribosome biogenesis downstream of Kras^G12D^. We first assessed the impact of Kras^G12D^ induction on nascent rRNA levels, using single‐cell visualization of newly synthesized RNAs by 5‐fluorouridine (FUrd) pulse labeling (Percipalle & Louvet, 2012). Induction of Kras^G12D^ expression by the addition of Dox to Dox‐withdrawn cells triggered a strong accumulation of nascent RNA within the nucleolus of iKras PDAC cells. This accumulation was largely abrogated by short‐term treatment of the cells with an rRNA synthesis inhibitor (CX‐5461), suggesting that the nucleolar nascent RNA signal must be largely comprised of newly synthesized rRNA (Fig EV4A and B). Conversely, removal of Dox and the consequent loss of Kras^G12D^ expression resulted in a substantial reduction in nascent rRNA levels (Fig EV4C and D), collectively demonstrating a strong dependence of rRNA synthesis on Kras^G12D^ expression. Crucially, depletion of Ncl via two independent siRNA oligos abrogated nascent rRNA accumulation downstream of Kras^G12D^ (Figs 4A and B, and EV4E). As an independent approach, we also measured the levels of unprocessed pre‐rRNA by quantitative reverse transcription‐PCR (RT–qPCR), using a probe against the ITS1 region of 47S pre‐rRNA, which is removed during the first steps of pre‐rRNA processing (Pineiro *et al*, 2018). In agreement with the nascent RNA imaging results, Kras^G12D^ expression resulted in an increase in the levels of unprocessed pre‐rRNA, but this increase was abrogated upon Ncl depletion (Fig 4C). Together, these findings suggest that Kras^G12D^ acts to enhance pre‐rRNA expression via Ncl.

Synthesis of 47S pre‐rRNA is a critical step in the regulation of eukaryotic ribosome biogenesis (Pelletier *et al*, 2018), so we next assessed whether the Ncl‐dependent enhancement of pre‐rRNA levels by Kras^G12D^ leads to an increase in cellular ribosome levels. Time course TMT‐mediated quantitative proteomics analysis of iKras PDAC cells revealed a significant accumulation of ribosomal proteins (RPs) in response to induction of Kras^G12D^ expression (Fig EV4F and Dataset EV10). Conversely, RPs were depleted over time in response to loss of Kras^G12D^ expression (Fig EV4G and Dataset EV11). Category enrichment analysis revealed that protein categories corresponding to RPs and rRNA processing were among the most depleted in response to Kras^G12D^ loss (Fig EV4H and Dataset EV12). Since RPs are known to be highly unstable unless incorporated into mature ribosomal subunits (Lam *et al*, 2007), their stable accumulation in response to Kras^G12D^ expression is indicative of more ribosomes having been synthesized. Crucially, Ncl depletion abrogated the stable accumulation of RPs in response to Kras^G12D^ expression (Fig 4D), suggesting that enhancement of ribosome biogenesis downstream of Kras^G12D^ is dependent on Ncl. In keeping with these findings, the overall rate of protein synthesis in iKras PDAC cells, measured by puromycin incorporation (Mardakheh *et al*, 2015), was significantly boosted by Kras^G12D^ induction, but this was abrogated upon Ncl depletion (Fig 4E and F).

To determine whether the Erk1/2‐dependent phospho‐regulation of Ncl is important for regulating pre‐rRNA expression and ribosome biogenesis, we evaluated the impact of phospho‐defective (S4A) and phospho‐mimicking (S4D) mutants of Ncl on nascent pre‐rRNA levels, in the presence or absence of Kras^G12D^. In presence of Kras^G12D^, all iKras PDAC cells exhibited accumulation of nascent rRNA in their nucleolus, although nascent rRNA levels were mildly but significantly decreased in S4A expressing cells (Fig 4G and H). Kras^G12D^ removal abrogated nascent rRNA levels in the WT and S4A mutant expressing cells, but expression of the S4D mutant rescued nascent rRNA expression in the absence of Kras^G12D^ (Fig 4G and H). These results suggest that phosphorylation of Ncl is sufficient for driving pre‐rRNA expression downstream of Kras^G12D^, revealing a key role for this phospho‐regulation in the early stages of ribosome biogenesis. Importantly, RT–qPCR analysis revealed that expression of the S4D mutant caused a drastic accumulation of unprocessed pre‐rRNA in the absence of Kras^G12D^ (Fig 4I). This accumulation far exceeded the pre‐rRNA levels in the presence of Kras^G12D^, suggestive of a defect in pre‐rRNA processing (Dermit *et al*, 2020). Based on these results, we conclude that the phospho‐mimicking S4D mutant can enhance nascent pre‐rRNA expression in the absence of Kras^G12D^, but the cells are defective in mediating the downstream processing of pre‐rRNA. Accordingly, TMT‐mediated proteomics analysis revealed no S4D‐mediated rescue of ribosome biogenesis in the absence of Kras^G12D^ (Fig EV4I). Together, these findings suggest that Ncl phosphorylation downstream of Kras^G12D^ acts to enhance pre‐rRNA expression, but the subsequent processing of nascent pre‐rRNA likely requires other KRAS^G12D^‐activated factors.

### Ncl is crucial for oncogenic Kras‐mediated PDAC cell proliferation and tumorigenesis

**Figure 5:**
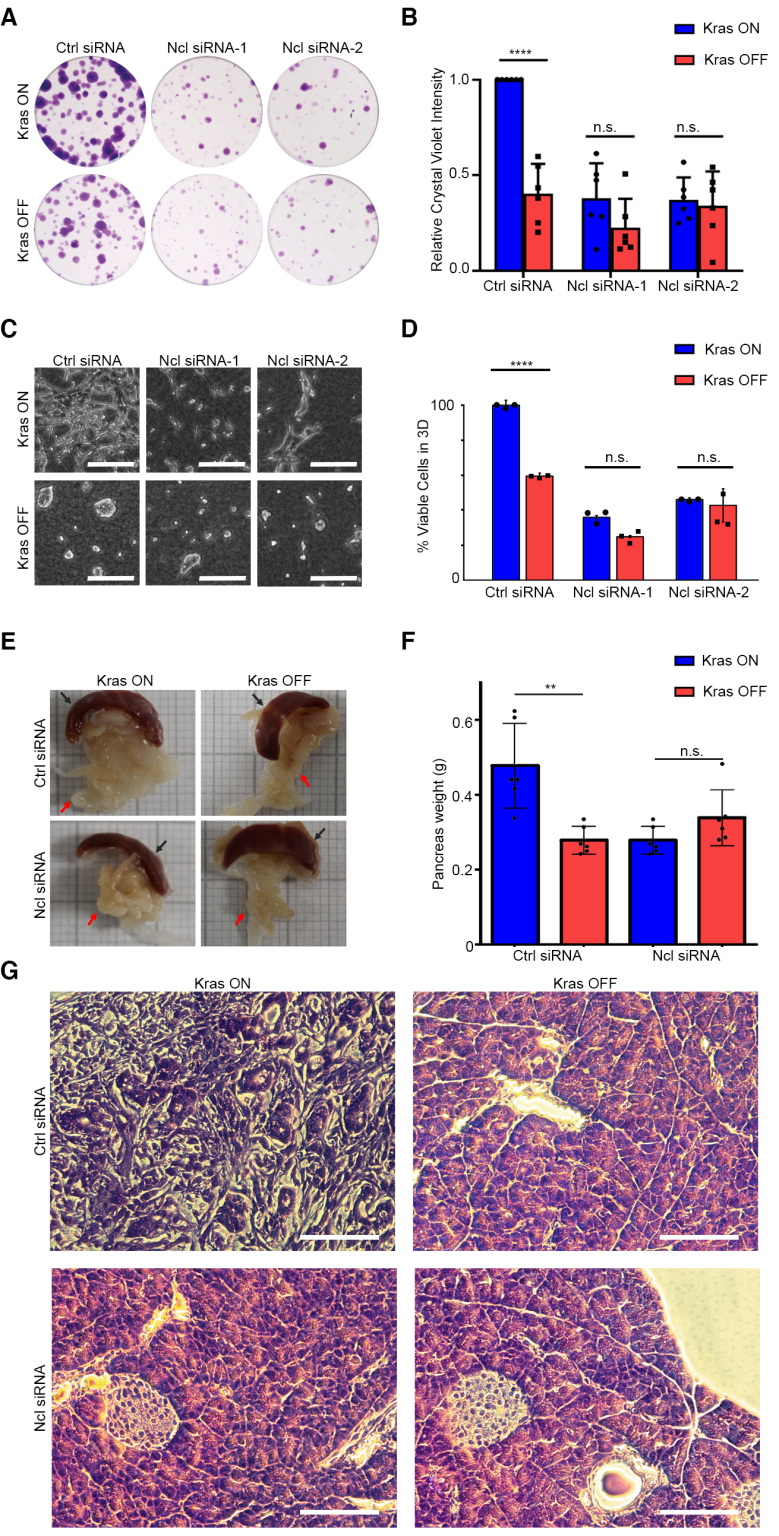
Ncl is necessary for Kras^G12D^‐mediated PDAC cell proliferation and tumor formation Colony formation of control and Ncl‐depleted iKras PDAC cells, in the presence or absence of Kras^G12D^. Cells were transfected with a nontargeting control siRNA, or two independent siRNAs against Ncl, followed by clonogenic assay for 7 days in the presence or absence of Dox. Colonies were visualized by Crystal Violet staining.Quantification of Crystal Violet staining levels from (A). A total of six biological replicate experiments were quantified. Error bars depict SD (****P < 0.0001; n.s.: not significant—calculated from two‐way ANOVA with Šídák's multiple comparisons test).3D proliferation of control and Ncl‐depleted iKras PDAC cells, in the presence or absence of Kras^G12D^. Cells were transfected with a nontargeting control siRNA, or two independent siRNAs against Ncl, before being reseeded onto 3D Collagen‐I gels, with or without Dox, and allowed to grow for 48 h. Cells were subsequently imaged live by phase contrast microscopy. Scale bar = 200 μm.Analysis of the relative percentage of viable cells in 3D collagen‐I cultures from (C). Cells were subjected to luminescence‐based viability assay by CellTiter‐Glo to quantify the percentage of viable cells. A total of three biological replicate experiments were quantified. Error bars depict SD (****P < 0.0001; n.s.: not significant—calculated from two‐way ANOVA with Šídák's multiple comparisons test).Representative images of the pancreas from control or Ncl‐depleted iKras PDAC engrafted mice, in the presence or absence of Kras^G12D^. Nontargeting control or Ncl siRNA transfected iKras PDAC cells were orthotopically engrafted into the pancreas of nude mice. Mice were fed either Dox‐containing (Kras ON) or Dox‐free (Kras OFF) water for 7 days, before culling and extraction of their pancreas (red arrow). Spleen (black arrow), which is located adjacent to the pancreas, was also extracted and included in the images for comparison.Quantification of pancreas weights from (E), as a measure of orthotopic tumor growth. Pancreas weights from six animals per condition were quantified (**P < 0.01; n.s.: not significant—calculated from unpaired *t*‐test). All samples were randomized and the measurements were performed blindly. Error bars depict SD.H&E analysis of the extracted pancreas tissues from (E). Extensive portions of the tissue in control‐engrafted mice fed with Dox display PDAC histology, with malignant ductal structures surrounded by stroma. However, typical pancreas histology comprised of acini, islets, and normal ducts is observed in all other conditions. Scale bar = 100 μm. Source data are available online for this figure.

**Figure EV5:**
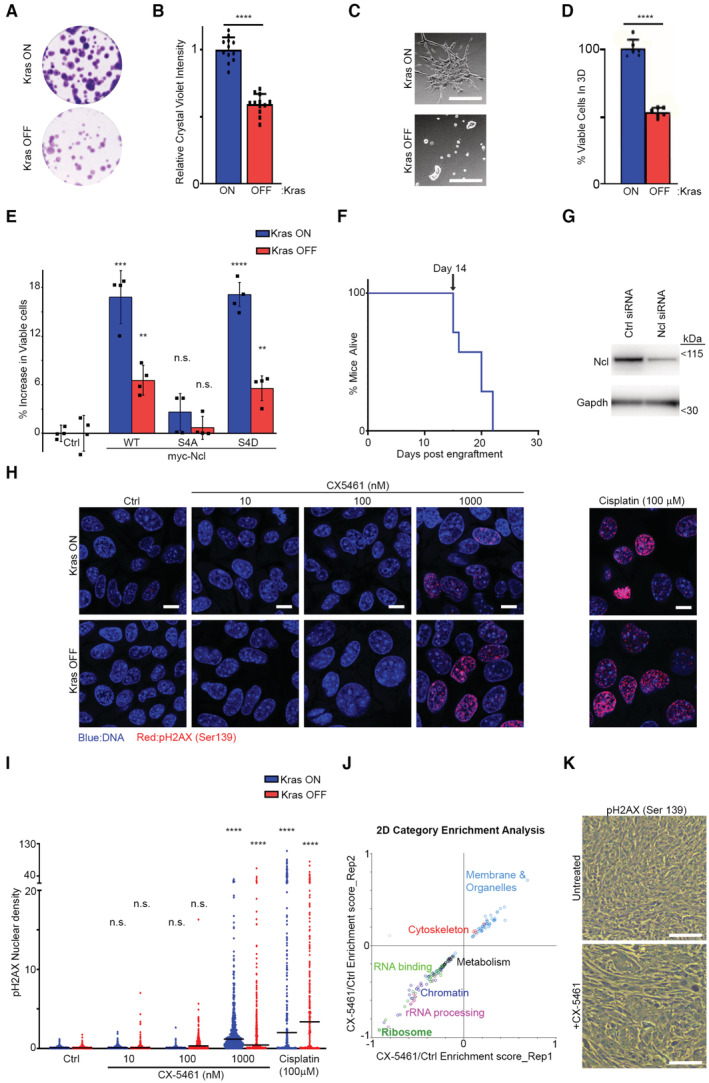
CX‐5461 does not induce DNA damage at low nanomolar doses, irrespective of Kras^G12D^‐induced cell proliferation Kras^G12D^ enhances iKras PDAC cell proliferation in 2D cell culture. IKras PDAC cells were seeded and subjected to clonogenic assay for 7 days, in the presence (Kras ON) or absence (Kras OFF) of Dox. Colonies were visualized by Crystal Violet staining.Quantification of Crystal Violet staining levels from (A). A total of 12 biological replicate experiments were quantified. Error bars depict SD (****P < 0.0001—calculated from unpaired *t*‐test).Kras^G12D^ enhances iKras PDAC cell proliferation in 3D cell culture. IKras PDAC cells were seeded onto 3D Collagen‐I gels, with (Kras ON) or without (Kras OFF) Dox, and allowed to grow for 48 h. Cells were subsequently imaged live by phase contrast microscopy. Scale bar = 200 μm.Analysis of the percentage of viable cells in 3D cultures of (C). Cells were subjected to luminescence‐based viability assay by CellTiter‐Glo to quantify the relative percentage of viable cells. A total of six biological replicate experiments were quantified. Error bars depict SD (****P < 0.0001—calculated from unpaired *t*‐test).Analysis of the impact of ectopic expression of Ncl or its phospho‐mutants on iKras PDAC cell proliferation. IKras PDAC cells were seeded with (Kras ON) or without (Kras OFF) Dox and transfected the next day with myc‐tagged wild‐type (WT), phospho‐defective (S4A), or phospho‐mimicking (S4D) mutants of Ncl, along with an empty vector negative control (Ctrl). Percentage of change in the number of viable cells relative to Ctrl was then quantified by CellTiter‐Glo assay 48‐h post‐transfection. A total of four biological replicate experiments were quantified. Error bars depict SD. Significance was calculated relative to each corresponding control (Kras ON or OFF) condition (****P < 0.0001; ***P < 0.001; **P < 0.01; n.s.: not significant—calculated from unpaired *t*‐test).Kaplan–Meier overall survival analysis of nude mice orthotopically engrafted with iKras PDAC cells (Cohort size = 7). Mice were Dox‐fed throughout the analysis. Arrow marks the day of the first mortality event.Analysis of Ncl expression in iKras PDAC cells that were used for orthotopic xenograft studies in Fig 5E–G. IKras PDAC cells were transfected with Ctrl and Ncl siRNAs, before injection into the pancreas of nude mice for orthotopic analysis. In parallel, a fraction of the cells from each siRNA treatment were lysed and analyzed by immunoblotting with the indicated antibodies.Dose–response analysis of CX‐5461 impact on DNA damage. CX‐5461 induces DNA damage only at the highest tested dose (1,000 nM). IKras PDAC cells were grown in the absence of Dox for 48 h. Cells were subsequently treated for 24 h with the indicated concentrations of CX‐5461, or Cisplatin as positive control, with or without co‐addition of Dox to induce Kras expression. Treated cells were then fixed and immunostained with anti‐pH2AX (Ser 139) antibody which marks DNA damage foci (red), and Hoechst as a Nuclear stain (blue), followed by confocal microscopy analysis. Scale bar = 10 μm.Quantification of nuclear pH2AX signal intensity from (A). Fluorescence density of pH2AX in single nucleoli was quantified from 237 to 570 individual cells per condition, combined from two independent biological replicate experiments. Significance was calculated relative to each corresponding untreated control (Kras ON or OFF) condition (****P < 0.0001; n.s.: not significant—calculated from unpaired *t*‐test).CX‐5461 treatment impact on the proteome of iKras PDAC cells mimics Kras^G12D^ removal. IKras PDAC cells grown in the presence of Dox were treated with or without CX‐5461 (100 nM) for 48 h, before being lysed and analyzed by TMT‐mediated quantitative proteomics (Dataset EV13). CX‐5461‐induced changes from two independent biological replicate experiments were then subjected to 2D‐annotation enrichment analysis. Each data point represents a functional category from GO and KEGG databases, with similar categories highlighted with the same colors (Dataset EV14). Similar to the impact of Kras^G12D^ removal (Fig EV4H), protein categories related to Ribosome and rRNA processing exhibit significant downregulation following 48 h of CX‐5461 treatment, while those related to cytoskeleton and membranous organelles show upregulation (FDR < 0.02). No significant change in protein categories related to DNA damage response was detected.IHC analysis of tumors from Fig 6I with anti‐pH2AX (Ser 139) antibody. No pH2AX signal, indicative of DNA damage, was detectable in tumors from either the control or CX‐5461 (50 mg/kg) treated mice. Scale bar = 50 μm.

Hyperactive ribosome biogenesis is a hallmark of most malignancies, acting to sustain augmented protein synthesis that underpins unrestricted cancer cell proliferation and tumor growth (Pelletier *et al*, 2018). We therefore investigated whether the Ncl‐mediated enhancement of ribosome biogenesis downstream of Kras^G12D^ was crucial for PDAC cell proliferation and tumorigenesis. As demonstrated before (Ying *et al*, 2012), Kras^G12D^ removal significantly reduced the proliferation of iKras PDAC cells in long‐term clonogenic assays under standard 2D cell culture settings (Fig EV5A and B). The impact of Kras^G12D^ expression on PDAC cell proliferation was also evident in a 3D matrix made up of collagen‐I, which forms the bulk of PDAC extracellular matrix, with rapid proliferative growth of the cell mass into the matrix that was strictly dependent on Kras^G12D^ expression (Fig EV5C and D). Depletion of Ncl decreased cell proliferation, abrogating the effect of Kras^G12D^ in both 2D and 3D culture settings, revealing a strong dependence of Kras^G12D^ on Ncl for enhancement of PDAC cell proliferation (Fig 5A–D). Conversely, transient overexpression of WT Ncl or its phospho‐mimicking S4D mutant, but not the phospho‐defective S4A mutant, significantly boosted PDAC cell proliferation (Fig EV5E).

Using orthotopic xenografts of the iKras PDAC cells, we next investigated whether Kras^G12D^‐induced tumor formation *in vivo* was dependent on Ncl. Orthotopic xenografts of iKras PDAC cells have been demonstrated to generate tumors which faithfully recapitulate the histological and molecular features of PDAC, in a Dox‐dependent manner (Ying *et al*, 2012). In our pilot studies, tumors were fully established within a week of engraftment in Dox‐fed mice, with animals having to be sacrificed 2–3‐week post‐engraftment due to rapid disease progression (Fig EV5F). Thus, we chose a time scale of 1‐week post‐engraftment for investigating the impact of Ncl depletion on Kras^G12D^‐dependent tumor formation. Dox‐mediated Kras^G12D^ expression triggered significant tumor growth in the pancreas of the engrafted mice, which was abrogated upon Ncl depletion (Figs 5E and F, and EV5G). Histological analysis further revealed an extensive Kras^G12D^‐dependent growth of the malignant component and its associated stroma within the pancreas of the engrafted mice, which was completely lost upon depletion of Ncl (Fig 5G). Collectively, these results reveal that Ncl is crucial for PDAC cell proliferation and tumor formation downstream of Kras^G12D^.

### Kras dependency on Ncl‐mediated ribosome biogenesis can be therapeutically targeted

**Figure 6:**
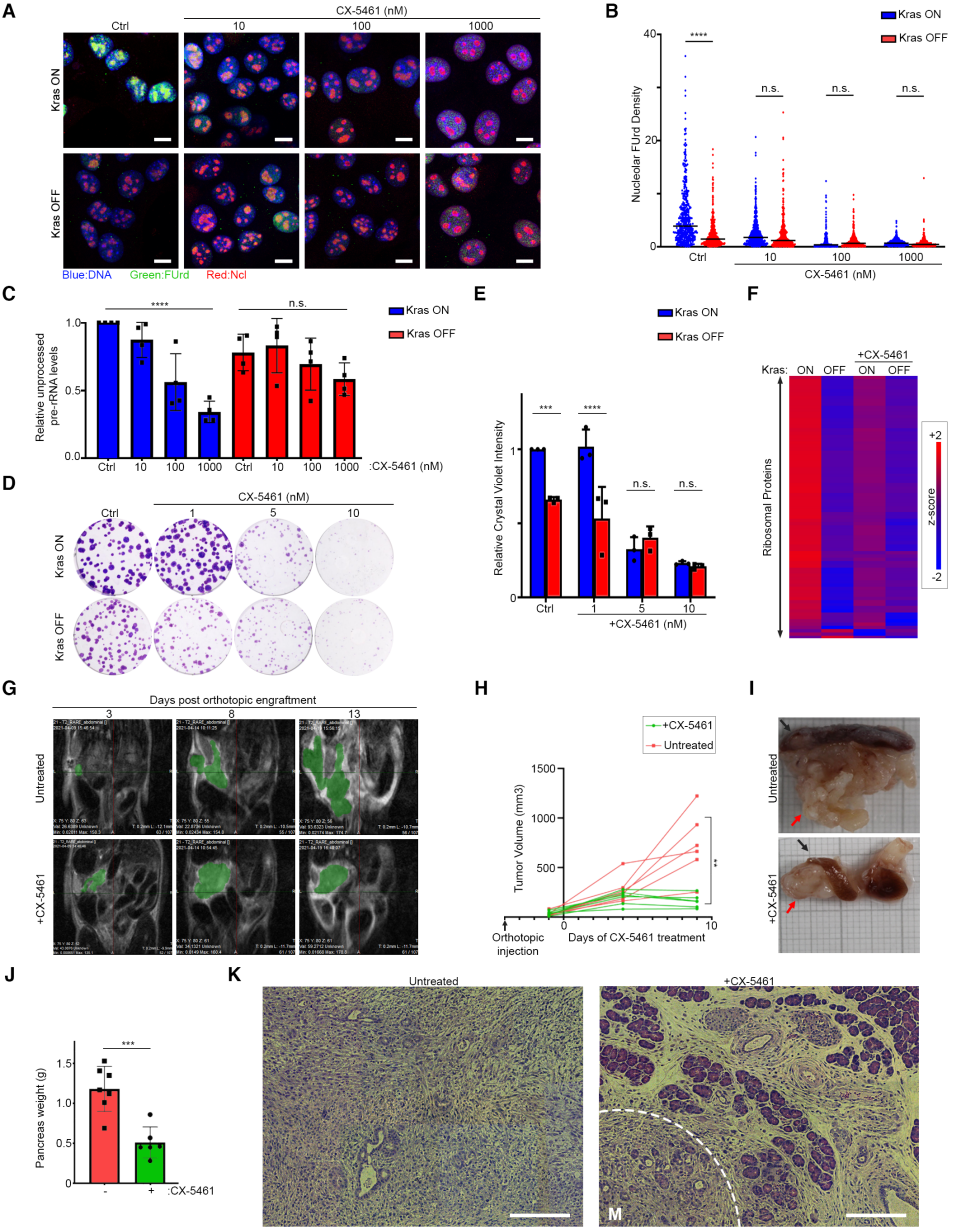
PDAC dependency on Kras^G12D^‐induced ribosome biogenesis can be therapeutically targeted Dose–response analysis of CX‐5461 impact on nascent rRNA expression. IKras PDAC cells were grown in the absence of Dox for 48 h. Cells were subsequently treated with or without Dox for 24 h to induce Kras^G12D^ expression, followed by pretreatment with the indicated concentrations of CX‐5461 for 30 min prior to pulse labeling with FUrd. Cells were then fixed and immunostained with anti‐FUrd antibody to visualize nascent RNA (green), anti‐Ncl antibody to reveal the Nucleolus (red), and Hoechst (blue) as the Nuclear stain, followed by confocal microscopy analysis. Scale bar = 10 μm.Quantification of Nucleolar FUrd levels in images from (A). FUrd fluorescence densities in single nucleoli were quantified from 251 to 479 individual cells per condition, combined from two independent biological replicate experiments (****P < 0.0001; n.s.: not significant—calculated from one‐way ANOVA with Šídák's multiple comparisons test).Dose–response analysis of CX‐5461 impact on ITS1‐containing pre‐rRNA transcript levels. IKras PDAC cells were grown in the absence of Dox for 48 h. Cells were subsequently treated for a further 24 h with the indicated concentrations of CX‐5461, with or without co‐addition of Dox to induce Kras expression, before RT–qPCR analysis with a specific probe against the mouse ITS1 region. A probe against mouse Actb mRNA was used as loading control for normalization. A total of four biological replicate experiments were quantified. Error bars depict SD (***P < 0.0001; n.s.: not significant—calculated from one‐way ANOVA on each set of Kras ON or OFF samples).Dose–response analysis of CX‐5461 impact on Kras^G12D^‐driven colony formation of iKras PDAC cells. IKras PDAC cells, seeded with or without Dox, were subjected to clonogenic assay in the presence of the indicated doses of CX‐5461 for 7 days. Colonies were visualized by Crystal Violet staining.Quantification of Crystal Violet staining levels from (E). A total of three biological replicate experiments were quantified. Error bars depict SD (****P < 0.0001; ***P < 0.001; n.s.: not significant—calculated from two‐way ANOVA with Šídák's multiple comparisons test).Quantitative analysis of RP levels in vehicle or 100 nM CX‐5461‐treated iKras PDAC cells, in the presence or absence of Kras^G12D^. IKras PDAC cells were grown for 48 h, with or without Dox, and in the presence or absence of 100 nM CX‐5461. Cells were then lysed and analyzed by TMT‐mediated quantitative mass spectrometry. Z‐scores of TMT intensity changes for all identified RPs across the different conditions were plotted as a heat map (red → increase; blue → decrease).MRI imaging of orthotopic iKras tumors in untreated or CX‐5461‐treated mice. IKras PDAC cells were engrafted into the pancreas of Dox‐fed nude mice and allowed to form tumors for 4 days. Animals were then divided into two groups, with the first group treated by daily oral administration of CX‐5461 (50 mg/kg) for a further 10 days, while the second group was left untreated for the same period. T2 scans were taken on the indicated days, post‐engraftment. Green areas mark the tumors.Quantification of tumor volumes from (G). For each condition (treated vs untreated), six engrafted animals were analyzed by MRI imaging (**P < 0.01; n.s.: not significant—calculated from unpaired *t*‐test). All measurements were performed blindly.Representative images of the pancreas (red arrows) from untreated and CX‐5461‐treated mice in (G), extracted at the end of the treatment (day 14). Spleen (black arrow), which is located adjacent to the pancreas, was also included in the images for comparison.Quantification of pancreas weights from (I). Pancreas weights from seven untreated and six treated animals were measured after extraction. Error bars depict SD (***P < 0.001; n.s.: not significant—calculated from unpaired *t*‐test). All samples were randomized and the measurements were done blindly.H&E analysis of the extracted pancreas tissues from (I). Representative pancreatic tissue images from the untreated and CX‐5461‐treated mice, showing typical PDAC histology with malignant ductal structures surrounded by stroma in the untreated, but a largely normal pancreas histology with a small malignant (M) component (marked by the dashed line) in the treated mice. Scale bar = 200 μm. Source data are available online for this figure.

Our results reveal a dependency for Ncl in Kras^G12D^‐mediated PDAC tumor formation, suggesting a possibility for therapeutic exploitation. Ncl has been a subject of significant interest as a therapeutic target for a diverse range of cancers, owing to its often strong upregulation of expression combined with its various proposed pro‐malignancy functions (Abdelmohsen & Gorospe, 2012). However, most efforts so far have been focused on cell‐surface‐localized Ncl, which is commonly observed in cancerous but not normal tissues. Accordingly, several compounds that can target extracellularly localized Ncl have been developed (Abdelmohsen & Gorospe, 2012), but these compounds are unlikely to reach nucleolar Ncl in significant quantities due to lack of cell permeability. We demonstrated that Ncl is exclusively localized to the nucleolus, associated with pre‐rRNA, and functions to promote ribosome biogenesis in iKras PDAC cells. We thereby reasoned that pharmacological inhibition of ribosome biogenesis should functionally mimic Ncl removal. For this purpose, we utilized CX‐5461, as it can be orally administered, *in vivo* (Drygin *et al*, 2011). CX‐5461 has shown promising results in early clinical trials against a number of human malignancies (Hilton *et al*, 2018; Khot *et al*, 2019). However, its *in vivo* mechanism of action has been subject to controversy. Early reports suggested that the primary antitumor activity of CX‐5461 arises from inhibition of ribosome biogenesis (Drygin *et al*, 2011; Bywater *et al*, 2012). However, recent studies have revealed that CX‐5461 can also induce DNA damage, which seems to act as the primary cause of its cytotoxicity in several cancer cell lines (Negi & Brown, 2015; Quin *et al*, 2016; Xu *et al*, 2017; Bruno *et al*, 2020; Sanij *et al*, 2020; Pan *et al*, 2021). This is proposed to be initiated by the irreversible arrest of RNA polymerase I on rDNA promoter regions, leading to nucleolar stress that propagates into a genome‐wide DNA damage response (Mars *et al*, 2020). Importantly, a recent study has shown that the dosage of CX‐5461 could be adjusted to minimize DNA damage induction, while still achieving an effective inhibition of rRNA synthesis (Prakash *et al*, 2019). In light of this, we performed a dose titration of CX‐5461 in iKras PDAC cells and assessed the treatment impacts on nascent rRNA expression as well as DNA damage. Short‐term treatment of iKras PDAC cells with low nanomolar doses of CX‐5461 induced a potent inhibition of Kras^G12D^‐induced nascent rRNA expression (Fig 6A and B). This was followed by a significant depletion in the Kras^G12D^‐induced levels of pre‐rRNA (Fig 6C). CX‐5461 treatment also induced DNA damage in iKras PDAC cells, but this only occurred at the 1 μM dose, and irrespective of the Kras^G12D^ status (Fig EV5H and I). Consistent with the proposed role of nucleolar stress in this process, the 1 μM dose also caused the disruption of nucleoli, as evidenced by the leakage of Ncl into the nucleoplasm, which was not observed at lower CX‐5461 doses (Fig 6A). Critically, low nanomolar doses of CX‐5461 still abrogated the impact of Kras^G12D^ on the proliferation of iKras PDAC cells (Fig 6D and E), suggesting that inhibition of rRNA synthesis is the critical target of CX‐5461 in the context of Kras^G12D^‐driven cell proliferation.

Next, we evaluated the impact of CX‐5461 on the proteome of iKras PDAC cells. Similar to Kras^G12D^ removal, nanomolar dose treatment of CX‐5461 induced a strong decrease in the levels of Ribosome and rRNA processing protein categories, without affecting protein categories involved in the DNA damage response (Fig EV5J, Datasets EV13 and EV14). Moreover, when combined with Kras^G12D^ induction, this CX‐5461 treatment could suppress the Kras^G12D^‐induced accumulation of RPs (Fig 6F). These results suggest that nanomolar doses of CX‐5461 primarily inhibit the iKras PDAC cell proliferation via inhibition of Kras^G12D^‐driven ribosome biogenesis, with significant DNA damage occurring only at higher doses.

Finally, we assessed whether pharmacological inhibition of ribosome biogenesis by CX‐5461 could inhibit Kras^G12D^‐induced tumor growth, *in vivo*. To mimic therapeutic settings, orthotopic xenograft tumors were first established in Dox‐fed mice for 4 days, before the animals were subjected to treatment by oral administration of CX‐5461 for 10 days, or left untreated for the same period as control. Magnetic resonance imaging (MRI) was used to monitor disease progression for the duration of the experiment. MRI imaging revealed that while tumors grew rapidly in untreated mice, tumor growth was halted in response to CX‐5461 treatment (Fig 6G and H). End‐point analysis of the pancreatic tissues also revealed a significant inhibition of tumor growth in CX‐5461‐treated mice, with histological analysis showing a strong reduction in the proportion of the malignant component in comparison with untreated animals (Fig 6I–K). Immunohistochemistry (IHC) analysis revealed no evidence of DNA damage in the tumors, suggesting that the *in vivo* CX‐5461 dose used in our study (50 mg/kg) is comparable to the low‐dose treatments *in vitro* that do not induce DNA damage (Fig EV5K). Together, these results demonstrate that pharmacological inhibition of ribosome biogenesis, the critical downstream target of the Kras‐Ncl axis, could be used as a therapeutic strategy for inhibiting PDAC growth.

## Discussion

As central modulators of post‐transcriptional regulation, many RBPs have been shown to play key roles in cancer development and progression (Kang *et al*, 2020). Direct alteration of RBPs by mutation is relatively rare in cancer (Gebauer *et al*, 2021), so a key question concerns the mechanistic link between mutations in cancer driver genes and the resulting dysregulation of RBPs. In this study, we used an inducible mouse model of PDAC, combined with a whole‐transcriptome quantitative RIC approach, to unbiasedly assess changes in the RBPome in response to induction of oncogenic RAS signaling. Our results reveal a drastic rewiring of the RBPome upon Kras^G12D^ induction, with many conventional RBPs showing an increase in their association with RNA, while nonconventional RBPs exhibit decreased association. This switch occurs downstream of ERK1/2 and is achieved not only through modulation of the expression of RBPs but also their RNA‐binding activity. In particular, we reveal a network of nuclear RBPs that include Ncl, whose RNA‐binding activity increases upon Kras^G12D^ induction. Several of these RBPs undergo phosphorylation downstream of ERK1/2, and in the case of Ncl, we demonstrate that these phosphorylations act to enhance the RNA‐binding activity of Ncl. On the contrary, little phosphorylation changes were observed within nonconventional RBPs whose RNA‐binding activity was reduced upon Kras^G12D^ induction, suggesting that other mechanisms must be at play in regulating their activity. It is tempting to speculate that other types of post‐translational modifications may be involved in modulating the activity of these RBPs, but further work will be necessary to define the mechanisms of regulation, as well as the functional significance of these RBPs in the context of oncogenic RAS signaling. Nevertheless, our study demonstrates that the RNA‐binding of many conventional and nonconventional RBPs is highly dynamic and subject to regulation by oncogenic signaling. Crucially, in case of Ncl, we showed that its phosphorylation downstream of ERK1/2 was mediated by CK2, a serine/threonine kinase that has been implicated in ribosome biogenesis (Sailer *et al*, 2022). Despite harboring a constitutively active kinase domain (Sarno *et al*, 2002), previous studies have shown that a number of kinase signaling pathways such as ERK, SRC, AKT, and PKC can modulate the activity of CK2 by phosphorylating it (Donella‐Deana *et al*, 2003; Ji *et al*, 2009; Nguyen le & Mitchell, 2013). Accordingly, we revealed that upon Kras^G12D^ induction, ERK1/2 phosphorylated CK2 on T360/S362, leading to its enhanced activity and Ncl phosphorylation.

Ribosome biogenesis is a highly coordinated cellular process, which involves stepwise synthesis, processing, modification, and assembly of rRNA and RPs into mature ribosomal subunits (Pelletier *et al*, 2018). Ncl is known to play a key role in several steps of ribosome biogenesis, from promotion of pre‐rRNA synthesis (Roger *et al*, 2003; Cong *et al*, 2012), to mediating the first steps of pre‐rRNA processing (Ginisty *et al*, 1998), as well as loading of ribosomal proteins onto pre‐rRNA (Bouvet *et al*, 1998). The central RRM domains of Ncl, which mediate its binding to RNA, have been shown to be important for enhancing nascent pre‐rRNA expression (Storck *et al*, 2009), suggesting that this enhancement must be at least in part achieved post‐transcriptionally. In line with these findings, our results reveal that oncogenic RAS signaling activates the RNA‐binding activity of Ncl through promoting CK2‐mediated phosphorylation of Ncl in its N‐terminal region, leading to enhancement of pre‐rRNA expression, promotion of ribosome biogenesis, and increased protein synthesis. It is not yet clear how Ncl could act to upregulate pre‐rRNA expression post‐transcriptionally, but structural studies suggest that Ncl may act as an RNA chaperone to mediate the proper folding of pre‐rRNA (Allain *et al*, 2000). Interestingly, recent data suggest that such nascent RNA folding could be crucial for enhancing RNA polymerase‐I elongation rate by inhibiting polymerase backtracking (Turowski *et al*, 2020), thus providing a possible mechanism for post‐transcriptional enhancement of pre‐rRNA synthesis via Ncl's binding to pre‐rRNA. Importantly, we revealed that while the expression of the phospho‐mimicking Ncl mutant could rescue nascent pre‐rRNA expression in the absence of RAS oncogene, downstream processing of pre‐rRNA was blocked, indicating that the activity of other RAS‐driven factors is likely to be crucial for mediating rRNA processing. Accordingly, our qRIC analysis revealed that in addition to Ncl, the RNA‐binding activity of several other ribosome biogenesis factors was induced upon oncogenic RAS signaling. Whether any of these factors could be playing a role in mediating other key steps of ribosome biogenesis downstream of RAS remains to be determined. Nevertheless, our findings reveal that phosphorylation of Ncl downstream of RAS plays a key role in mediating rRNA synthesis, which is the crucial initiating step in the process of ribosome biogenesis.

Our iCLIP analysis of WT and the phospho‐mutants of Ncl suggests that the sites of Ncl interaction with pre‐rRNA are not affected by RAS‐dependent phosphorylation. Although it is possible that our iCLIP has not captured the complete repertoire of Ncl binding sites, for instance due to competition between ectopic myc‐tagged and endogenous Ncl, the absence of any significant differences between the mutants suggests that Ncl phosphorylation must be boosting its affinity without changing its RNA‐binding specificity. This is in agreement with the notion that the central RRM domains of Ncl are the primary drivers of specific binding to RNA (Allain *et al*, 2000). The N‐terminal region of Ncl is intrinsically disordered, consisted of repetitive acidic and basic‐rich stretches that create alternating charged blocks. Such repeats are common among several nucleolar proteins and often contain CK2 phosphorylation sites within their acidic stretches that further enhance their charge blockiness when phosphorylated. Intriguingly, a recent report has revealed that charge blockiness can regulate the condensation propensities of two other nucleolar proteins, NPM1 and Ki‐67 (Yamazaki *et al*, 2022). CK2 phosphorylation of Ncl may therefore act by boosting its condensation propensity, thus promoting weak multivalent interactions with pre‐rRNAs in a nonspecific manner. It remains to be determined whether a change in the condensation propensity of Ncl can indeed be triggered by its RAS‐dependent phosphorylation, and whether this change underlies the enhancement in its affinity for RNA, without a change in its binding specificity.

Hyperactive ribosome biogenesis is a common feature of a wide variety of human cancers, playing a pivotal role in sustaining the growth and proliferation of cancer cells (Pelletier *et al*, 2018). Pre‐rRNA synthesis is considered to be the rate‐limiting step in the process of human ribosome biogenesis (Lam *et al*, 2007). However, mechanisms by which cancer cells upregulate pre‐rRNA production are only beginning to be characterized (Hannan *et al*, 2003; Zhao *et al*, 2003; Arabi *et al*, 2005; Delloye‐Bourgeois *et al*, 2012; Justilien *et al*, 2017; Prakash *et al*, 2019). Here, we reveal that through phospho‐regulation of Ncl, oncogenic RAS signaling enhances pre‐rRNA expressionand ribosome biogenesis. This upregulation is crucial for mediating PDAC cell proliferation and tumorigenesis. In contrast, depletion of Ncl, or pharmacological inhibition of pre‐rRNA synthesis by CX‐5461, results in abrogation of Kras^G12D^‐induced ribosome biogenesis and protein synthesis, leading to inhibition of PDAC cell proliferation and tumor growth. It is not exactly clear how enhanced ribosome biogenesis can promote malignancy, but an attractive model postulates that an increase in the protein synthetic capacity of the cells may disproportionately enhance the translation of mRNAs that are inherently poorly translated, many of which encode for key mediators of cell proliferation (Robichaud *et al*, 2019). Importantly, in agreement with a previous study (Prakash *et al*, 2019), we confirm that when used at low nanomolar doses, the antiproliferative effects of CX‐5461 are mediated by inhibition of ribosome biogenesis, and not DNA damage induction, which can be caused at higher doses in our model. Based on these results, targeting ribosome biogenesis, either alone or in combination with other targeted therapies, appears to be a promising therapeutic avenue against RAS‐driven cancers. CX‐5461 has shown promise in early clinical trials (Hilton *et al*, 2018; Khot *et al*, 2019), and while our results suggest that its use could be therapeutically beneficial against PDAC, extra care must be taken in choosing the treatment dosage if DNA damage‐related side effects are to be avoided. Alternatively, more specific inhibitors of rRNA synthesis may become available in the near future. Based on our findings, therapeutic effectiveness of such inhibitors against RAS‐driven cancers warrants further investigation. In addition, direct inhibition of nucleolar Ncl, or its phospho‐regulation downstream of RAS, could be another attractive strategy for targeting RAS‐driven tumors.

## Materials and Methods

### Reagents and Tools table

**Table:** 

Reagent or resource	Manufacturer	Reference
**RT–qPCR primers**		
ITS1 fwd 5′‐CTCCCCGTCTTGTGTGTGTCCTCGCCG‐3′	Merck	Custom oligo
ITS1 rev 5′‐CCACCCCTTCTCTCACCTCACTCCAGACACCT‐3′	Merck	Custom oligo
Actb fwd 5′‐CGCCACCAGTTCGCCATGGA‐3′	Merck	Custom oligo
Actb rev 5′‐TACAGCCCGGGGAGCATCGT‐3′	Merck	Custom oligo
**iCLIP 3**′ **RNA adapter primer**		
(/5Phos/AG ATC GGA AGA GCG GTT CAG AAA AAA AAA AAA/iAzideN/AA AAA AAA AAA A/3Bio/)	Integrated DNA Technologies	Custom oligo
**iCLIP Reverse Transcription barcoded primers (5′‐3′)**		
/5Phos/ WWW GTGGA NNNN AGATCGGAAGAGCGTCGTGAT /iSp18/ GGATCC /iSp18/ TACTGAACCGC	Integrated DNA Technologies	Custom oligo
/5Phos/ WWW TCCGG NNNN AGATCGGAAGAGCGTCGTGAT /iSp18/ GGATCC /iSp18/ TACTGAACCGC	Integrated DNA Technologies	Custom oligo
/5Phos/ WWW TGCCT NNNN AGATCGGAAGAGCGTCGTGAT /iSp18/ GGATCC /iSp18/ TACTGAACCGC	Integrated DNA Technologies	Custom oligo
/5Phos/ WWW TATTC NNNN AGATCGGAAGAGCGTCGTGAT /iSp18/ GGATCC /iSp18/ TACTGAACCGC	Integrated DNA Technologies	Custom oligo
/5Phos/ WWW TTAAA NNNN AGATCGGAAGAGCGTCGTGAT /iSp18/ GGATCC /iSp18/ TACTGAACCGC	Integrated DNA Technologies	Custom oligo
/5Phos/ WWW AAATG NNNN AGATCGGAAGAGCGTCGTGAT /iSp18/ GGATCC /iSp18/ TACTGAACCGC	Integrated DNA Technologies	Custom oligo
/5Phos/ WWW AAGGT NNNN AGATCGGAAGAGCGTCGTGAT /iSp18/ GGATCC /iSp18/ TACTGAACCGC	Integrated DNA Technologies	Custom oligo
/5Phos/ WWW AATAC NNNN AGATCGGAAGAGCGTCGTGAT /iSp18/ GGATCC /iSp18/ TACTGAACCGC	Integrated DNA Technologies	Custom oligo
/5Phos/ WWW ACGCA NNNN AGATCGGAAGAGCGTCGTGAT /iSp18/ GGATCC /iSp18/ TACTGAACCGC	Integrated DNA Technologies	Custom oligo
/5Phos/ WWW ACTTG NNNN AGATCGGAAGAGCGTCGTGAT /iSp18/ GGATCC /iSp18/ TACTGAACCGC	Integrated DNA Technologies	Custom oligo
/5Phos/ WWW AGAGC NNNN AGATCGGAAGAGCGTCGTGAT /iSp18/ GGATCC /iSp18/ TACTGAACCGC	Integrated DNA Technologies	Custom oligo
/5Phos/ WWW AGTCT NNNN AGATCGGAAGAGCGTCGTGAT /iSp18/ GGATCC /iSp18/ TACTGAACCGC	Integrated DNA Technologies	Custom oligo
**siRNA oligos**		
ON‐TARGETplus Nontargeting pool	Dharmacon	D‐001810‐10‐05
ON‐TARGETplus Ncl ORF	Dharmacon	J‐059054‐09
5′‐GCAAAUUCCUAUACAUCUA‐3′		
ON‐TARGETplus Ncl ORF	Dharmacon	J‐059054‐12
5′‐UGGGAAAAGUAAAGGGAUU‐3′		
ON‐TARGETplus Ncl 3′UTR	Dharmacon	CTM‐706612
5′‐GGACAUUCCAAGACAGUAAUU‐3′		
**Primary antibodies**		
Anti‐GAPDH	Novus Biologicals	NB300‐221
Anti‐Nucleolin	Abcam	ab22758
Anti‐Ras G12D (Mutant Specific) (D8H7)	Cell Signaling	14429S
Anti‐p44/42 MAPK (Erk1/2) (137F5)	Cell Signaling	4695S
Anti‐Phospho‐p44/42 MAPK (Thr202/Tyr204) (p‐Erk1/2) (E10)	Cell Signaling	9106S
Anti‐CK2 phospho‐Substrate [(pS/pT)DXE] mAb mix	Cell Signaling	8738S
Anti‐Fibrillarin (C13C3)	Cell Signaling	2639
Anti‐Phospho‐Histone H2A.X (20E3)	Cell Signaling	9718S
Anti‐Myc tag (9B11)	Cell Signaling	2276S
Anti‐Brdu	Merck	B2531‐100UL
Anti‐NPM1	Fisher Scientific	10202223
Anti‐CK2α1 (CSNK2A1) (pSer362), (pThr360) antibody	Gmbh	ABIN1870086
Anti‐CK2α Antibody	Cell Signaling	2656S
Anti‐Puromycin (12D10) antibody	Millipore	MABE343
**Secondary antibodies**		
Rabbit IgG HRP linked	GE Healthcare	NA934
Mouse IgG HRP linked	GE Healthcare	NA931
Cy3‐conjugated Donkey Anti‐Mouse IgG (H + L)	Jackson ImmunoResearch	715‐165‐150
Cy5‐conjugated Donkey Anti‐Rabbit IgG (H + L)	Jackson ImmunoResearch	711‐175‐152
Alexa Fluor 488‐conjugated Donkey Anti‐Mouse IgG (H + L)	Jackson ImmunoResearch	715‐545‐150
Alexa Fluor 647‐conjugated Donkey Anti‐Rabbit IgG (H + L)	Jackson ImmunoResearch	711‐605‐152
DyLight 549‐conjugated Donkey Anti‐Mouse IgG (H + L)	Jackson ImmunoResearch	715‐505‐151
**Chemicals**		
Hoechst 33258	Thermo Fisher	H3569
NuPAGE LDS Sample Buffer	Thermo Fisher	NP0008
Pierce ECL Plus Western Blotting Substrate	Thermo Fisher	32132
Luminata Crescendo Western HRP substrate	Fisher Scientific	10776189
5‐Fluorouridine	Fisher Scientific	15494529
Crystal violet	Merck	C6158
DTT	VWR	M109
Iodoacetamide	VWR	786–228
Urea	Merck	U1250—5KG
Trypsin	Merck	T6567‐1MG
TRIzol reagent	Fisher Scientific	12034977
CX‐5461	Cambridge Biosciences	HY‐13323‐50MG
Silmitasertib	Cambridge Biosciences	2459‐5
Trametinib	Cambridge Biosciences	CAY16292‐25 mg
SUPERase‐In RNase Inhibitor	Thermo Fisher	AM2694
Dox Hyclate	Merck	D9891‐10G
Chloroform	SLS	372978‐100ML
TRIETHYLAMMONIUM BICARBONATE (TEAB)	Fisher Scientific	15215753
Sodium dodecyl sulfate	Merck	75746‐1kg
Magnesium chloride	Severn Biotech Ltd.	20‐xxxx‐01
RNAseA, T1 mix	Thermo Fisher	EN0551
Acetone	Merck	34850‐2.5L
Ammonium Bicarbonate	Merck	A6141‐500G
Trifluoroacetic acid (TFA)	Thermo Fisher	85183
Acetic Acid	Honeywell	33209‐2.5L
Acetonitrile	J.T.Baker	9012
Tris–HCl pH 8.8	Severn Biotech Ltd.	20‐7900‐01
Methanol	Fisher Scientific	M/4056/17
Xylene: 97%	Fisher Scientific	10784001
IMS (Methylated spirit industrial 74 O.P.)	Fisher Scientific	11482874
Surgipath Hematoxylin Gill III	Leica Biosystems	3801542E
Surgipath Eosin Y: alcoholic solution	Leica Biosystems	3801601E
1% Acid Alcohol: Surgipath differentiating solution	Leica Biosystems	3803650
Formaldehyde solution	Merck	F8775‐500ML
Nuclease‐Free Water	Fisher Scientific	10526945
Protein G dynabeads	Thermo Fisher	100.02
RNase I	Thermo Fisher	EN0602
Turbo DNase	Thermo Fisher	AM2238
PNK	NEB	M0201L
FastAP thermosensitive alkaline phosphatase	Thermo Fisher	EF0654
RNasin Plus	Promega	N2611
T4 RNA ligase I	NEB	M0204L
NEBuffer 2	NEB	B7002S
5′ deadenylase	NEB	M0331S
RecJF endonuclease	NEB	M0264S
NuPAGE 4 to 12%, Bis‐tris, 1.0 mm, mini protein gel, 10‐well	Thermo Fisher	NP0321BOX
NuPAGE MOPS SDS running buffer	Thermo Fisher	NP0001
Protran nitrocellulose membrane, pore size 0.45 μm	Whatman	Z613630
NuPAGE transfer buffer	Thermo Fisher	NP0006
Proteinase K	Thermo Fisher	25530‐049
Phenol:chloroform:isoamyl alcohol	Merck	P3803
2 ml Phase lock gel heavy tube	VWR	713‐2536
Glycoblue	Ambion	9510
Superscript IV reverse transcriptase kit	Thermo Fisher	18090010
Exonuclease I	NEB	M0293S
Agencourt AMPure XP beads	Beckman Coulter	A63880
CircLigase II ssDNA ligase kit	Epicentre	CL9021K
Phusion HF master mix	Thermo Fisher	F531S
Novex TBE gel, 6%, 10 well	Thermo Fisher	EC6265BOX
SYRB green I 10,000×	Thermo Fisher	S7563
Costar SpinX column	Corning Inc.	8161
10 mm diameter Grade GF/D binder‐free glass fiber microfiber filter paper circle disc	Whatman	1823010
Agilent D1000 High sensitivity ScreenTape	Agilent	5067‐5584
Agilent D1000 High sensitivity reagents	Agilent	5067‐5584
Qubit dsDNA HS assay kit	Thermo Fisher	Q32851
**Tissue Culture reagents**		
iBiDi u‐Slide 18 Well flat, ibiTreat, Tissue Culture Treated, Sterile	Thistle Scientific	81826
Corning Tissue Culture Treated plates and dishes	Corning Inc.	Various products
Collagen‐I (Bovine, pepsinized)	CellSystems GmbH	5005‐100ML
Gibco Fetal Bovine Serum	Thermo Fisher	10437028
Gibco™ DMEM w/High Glucose	Fisher Scientific	41966‐029
Trypsin/EDTA solution	Fisher Scientific	R‐001‐100
Lipofectamine 2000 Transfection Reagent‐1.5 ml	Thermo Fisher	11668019
Lipofectamine™ RNAiMAX Transfection Reagent	Thermo Fisher	13778150
Opti‐MEM I Reduced Serum Medium‐100 ml	Thermo Fisher	31985062
Gibco DMEM w/High Glucose and w/o Glutamine, Lysine and Arginine (For SILAC)	Fisher Scientific	12817552
L‐Arginine	Merck	A6969‐25G
L‐Lysine	Merck	L8662‐25G
Heavy L‐Arginine [U‐13C6, U‐15N4]	Cambridge Isotopes	CNLM‐539‐H‐0.5
Heavy L‐Lysine [U‐13C6, U‐15N2]	Cambridge Isotopes	CNLM‐291‐H‐0.5
L‐Proline	Merck	P0380‐100G
Gibco Dialyzed Fetal Bovine Serum	Thermo Fisher	11520646
**Commercial kits and reagent sets**		
TMT6plex™ Isobaric Label Reagent Set	Thermo Fisher	90061
TMT10plex™ Isobaric Label Reagent Set	Thermo Fisher	90110
Pierce High pH Reversed‐Phase Peptide Fractionation Kit	Life Technologies	84868
Titansphere TiO phospho‐peptide enrichment kit	GL Sciences	5010‐21308
CellTiter‐Glo 3D Cell Viability Assay Viability Assay	Promega	G9682
Pierce™ BCA Protein Assay Kit	Thermo Fisher	23225
Brilliant II SYBR® Green QRT–PCR	Agilent Technologies	600825
MycoAlert™ PLUS Mycoplasma Detection Kit	Lonza	LT07‐705
**Plasmids**		
Myc‐Ncl (WT) PRK5‐DEST	Generated in this study	
Myc‐Ncl (S4A) PRK5‐DEST	Generated in this study	
Myc‐Ncl (S4D) PRK5‐DEST	Generated in this study	
**Software and algorithms**		
Maxquant	Max Planck Institute	https://www.biochem.mpg.de/5111795/maxquant
Perseus	Max Planck Institute	https://www.biochem.mpg.de/5111810/perseus
ImageJ	NIH	https://imagej.nih.gov/ij/
Prism	Graphpad	https://www.graphpad.com/scientific‐software/prism/
**Other**		
Vivacon 500, 30,000 MWCO Hydrosart	Sartorius	VN01H22
Crl:CD1‐Foxn1nu mice	Charles River UK	Strain Code 086
PK20 EMPORE OCTADECYL C18 47MM &	Merck	66883‐U
Phos‐tag™ SuperSep™ 7.5% acrylamide precast gel	Alpha Labs	192‐18001
Immobilon‐P 26.5 × 3.75 m PVDF (0.45 μm)	Millipore	IPVH00010

### Methods and Protocols

#### Cloning

WT and mutant Myc‐Ncl expression plasmids were generated by Gateway cloning of custom synthesized murine Ncl donor vectors (GeneArt) into the Myc‐pRK5‐DEST vector (Mardakheh *et al*, 2009).

#### Cell culture and transfections

Mouse iKras PDAC model was originally generated by Prof. Ronald DePinho's Laboratory (Ying *et al*, 2012). The iKras PDAC cells isolated from this model were a gift from Dr Christopher Tape. Cells were grown in DMEM supplemented with 10% FBS, 1% penicillin/streptomycin, and doxycycline (Dox) (1 μg/ml). To remove Kras^G12D^ expression, the same media without Dox was used. Cells were grown in humidified incubator at 37°C with 5% CO_2_ and routinely checked to be mycoplasma‐free by MycoAlert Plus mycoplasma detection kit. Collagen‐I matrix gels were prepared as described previously (Dermit *et al*, 2020) and used for assessment of cell growth in 3D. For siRNA‐mediated depletions, 10,000 cells/cm^2^ were seeded on standard TC‐treated polystyrene plates and transfected the next day using Lipofectamine RNAiMAX (Thermo), according to the manufacturer's instructions. A final siRNA concentration of 20 nM was used per condition. For efficient Ncl depletion, cells were double‐ or triple‐transfected at 48‐h intervals. For DNA transfections, 25,000 cells/cm^2^ were seeded on standard TC‐treated polystyrene plates and transfected the next day using Lipofectamine 2000 (Thermo), according to the manufacturer's instructions. A final DNA amount of 250 ng/cm^2^ was used per condition, and cells were reseeded the next day for downstream analyses.

#### Colony formation and viability assays

Colony formation assay was performed by seeding cells at 500 cells per well on a 6‐well plate, with or without Dox (1 μg/ml) for 5–7 days, with the media being replenished every 3 days. Cells were subsequently fixed with 4% formaldehyde on ice for 30 min in the dark, followed by staining with 0.5% crystal violet staining solution (0.5% w/v, 20% MeOH) for 10 min at RT. Plates were then imaged on an Amersham Imager 600, and ImageJ was used to quantify the crystal violet staining densities. For this purpose, images were first converted to 8‐bit gray scale, recalibrated using the *Uncalibrated OD* function, and Integrated Density of then measured and quantified for each well. For viability assay, cells were seeded at 20,000 cells per well of a 24‐well plate (containing 0.5 ml/well of Collagen‐I gel for 3D growth assays), with or without Dox (1 μg/ml) for 48 h. 3D CellTiter Glo reagent (Promega), diluted 1:4 in PBS, was then used to quantify cell viability in each well, according to the manufacturer's instructions. Luminescence was measured on a BMG Plate‐reader and analyzed using GraphPad PRISM.

#### Immunofluorescence (IF)

IF was carried out in 18 Well Flat μ‐Slides from iBidi. 2,000 cells were seeded in each well and grown for 48 h in the presence or absence of Dox (1 μg/ml), before the indicated treatments. Cells were washed with PBS and fixed in fixation buffer (4% Formaldehyde in PBS) for 15 min. The fixed cells were then permeabilized with permeabilization buffer (0.5% Triton‐X100 in PBS) for 10 min before 3× washes with PBS. Cells were then incubated with blocking buffer (4% BSA in PBS) for 30 min, before incubation with the indicated primary antibodies (diluted in blocking buffer) for 1 h at room temperature (RT). This was followed by 3× PBS washes and incubation with fluorophore‐conjugated secondary antibodies (diluted in blocking buffer) for another hour at RT in the dark. Slides were then washed again 3× with PBS and imaged on a Zeiss LSM 880 confocal microscope using a 63× oil immersion lens. FUrd pulse labeling was done according to Percipalle & Louvet (2012), with some modifications. Briefly, cells were pulsed for 30 min with 2 mM FUrd before fixation. RNAse‐free reagents were used for preparation of fixation, permeabilization, and blocking buffers, and the blocking buffer was supplemented with SUPERase‐In RNase Inhibitor (Thermo) at 1:500 dilution, to inhibit RNA degradation. To visualize FUrd incorporation, a monoclonal antibody raised against BrdU that also detects FUrd (B2531, Merck) was used.

#### Image analysis

All images were processed and analyzed using ImageJ. For analysis of nucleolar FUrd incorporation, a mask of the nucleoli was first generated using the fibrillarin or Ncl channels as nucleoli markers. For this purpose, the *Despeckle* function followed by *Smoothing* of the edges was first performed on the Fibrillarin or Ncl channels. Channel images were then converted to *Binary*, and the *Find Edges* function was used to create the nucleolar mask. Each binary nucleolus was subsequently selected as a *region of interest* (*ROI*) and used for quantification of the Integrated Density from the FUrd channel. For DNA damage analysis, Hoechst staining was used to generate a binary mask of the nucleus. For this purpose, the Hoechst channel image was first converted to *Binary*, and the *Find Edges* function was subsequently performed to create a nuclear mask. Next, each nucleus was selected as an *ROI*, and the Integrated Density of the pH2AX channel was then quantified within each nucleus.

#### Western blotting

For whole cell lysis, cells were lysed by direct addition of 2% SDS, 100 mM Tris/HCl pH 7.5 and sonicated with a sonicator bath (Bioruptor Pico—Rm 343) for 15 cycles. Sample concentration was adjusted with a Pierce BCA Protein Assay Kit (Thermo) before the addition of NuPAGE LDS Sample Buffer (Thermo) with reducing agent and boiling at 95°C for 10 min. After separation on a NuPage 4–12% Bis/Tris protein gel (Thermo), proteins were transferred to an Immobilon‐P membrane (Millipore) using a standard wet transfer device. Primary antibodies were diluted in 5%BSA, PBS and incubated on the membranes at 4°C overnight followed by incubation with anti‐mouse or rabbit HRP‐conjugated secondary antibodies at RT for 1 h. Membranes were then probed with Pierce ECL Plus HRP‐detection reagent followed by imaging on an Amersham Imager 600. Immunoblots (IBs) were quantified using ImageJ.

#### Phos‐tag SDS PAGE analysis

To analyze phosphorylation changes in Ncl by Phos‐tag SDS–PAGE, ectopically expressed myc‐tagged Ncl was first purified from iKras PDAC cells by immunoprecipitation. Briefly, empty vector control or Myc‐Ncl transfected iKras PDAC cells from 10 cm dishes were lysed in 1 ml of IP lysis buffer (50 mM Tris–HCl pH 7.4, 100 mM NaCl, 1% Igepal CA‐630, 0.1% SDS, 0.5% sodium deoxycholate, supplemented with phosphatase and protease inhibitor cocktails (Roche)), sonicated with a sonicator bath (Bioruptor Pico—Rm 343) for 15 cycles, and cleared by centrifugation (12,000 *g*, 10 min). The protein concentration of the cleared lysate was then measured and balanced, before immunoprecipitation with 4 μg of anti‐Myc‐tag antibody (Cell Signaling) per sample, preconjugated to protein G Dynabeads (Thermo). The beads were washed three times with 1 ml of lysis buffer, and the purified proteins were eluted by boiling the beads in 60 μl 2% SDS, 100 mM Tris/HCl pH 7.5, 0.1 M DTT for 5 min. The eluates were then subjected to Phos‐tag gel electrophoresis using Phos‐tag™ SuperSep™ 7.5% acrylamide precast gels, according to the manufacturer's instructions, before immunoblotting with anti‐Myc antibody to visualize the migration of Myc‐Ncl through the gels.

#### RT–qPCR

Total RNA was isolated using TRIzol reagent (Fisher), as per the manufacturer's instructions. RT–qPCR was performed using Brilliant II SYBR® Green one‐step kit (Agilent) on an ABI 7500 Real‐Time PCR system (Applied Biosystems). The 2^−ΔΔCT^ method was used for relative quantification of RNA expression levels, according to Rao *et al* (2013). β‐actin (Actb) mRNA was also quantified and used as an internal control for normalizations. All primers used for RT–qPCR analyses are listed in the Key Resources Table.

#### iCLIP

The iCLIP method was performed as previously described in preprint: Lee *et al* (2021), with some modifications. Briefly, triplicates of mock‐transfected or WT Myc‐Ncl, S4A Myc‐Ncl, S4D Myc‐Ncl transfected iKras PDAC cells were seeded onto 10 cm dishes (2 million per dish) and allowed to grow for 48 h in the presence of Dox (1 μg/ml), before being irradiated once on ice with 150 mJ/cm^2^ UV light (254 nm) in PBS, using a Hoefer Scientific UV Crosslinker. Cells were then lysed in lysis buffer (50 mM Tris–HCl pH 7.4, 100 mM NaCl, 1% Igepal CA‐630, 0.1% SDS, 0.5% sodium deoxycholate, supplemented with protease inhibitors), cleared, and diluted to a protein concentration of 1 mg/ml. RNA was then digested with 0.2 U/ml of RNase I. Myc‐tagged Ncl was then immunoprecipitated with 4 μg anti‐Myc‐tag antibody (Cell Signaling), preconjugated to protein G Dynabeads (Thermo). The RNAs were labeled at the 3′ end using an adapter (/5Phos/AG ATC GGA AGA GCG GTT CAG AAA AAA AAA AAA/iAzideN/AA AAA AAA AAA A/3Bio/) conjugated to an infrared dye to allow the visualization of the complexes on a gel. After the SDS–PAGE and the transfer onto nitrocellulose membrane, the region corresponding to 140–200 kDa protein‐RNA‐cross‐linked complexes was excised to isolate the associated RNAs. Isolated RNAs were reverse transcribed using primers containing experimental barcodes unique to each sample (see Key Resources Table for the primer sequences). The cDNAs were then PCR amplified, gel extracted, and equal amounts of each amplified library were then combined together into a single mixed pool, before sequencing on an Illumina NextSeq 500, producing 150‐nt single‐end reads. For iCLIP data analysis, the reads were trimmed and demultiplexed using Ultraplex and aligned using STAR (Dobin *et al*, 2013; Wilkins *et al*, 2021). Reads were first mapped to a genome containing the ribosomal DNA repeat and other short ncRNA sequences from GENCODE vM22, before being mapped to the mm10 genome using GENCODE vM22 annotation. PCR duplicates were collapsed using UMI‐tools (Smith *et al*, 2017). The cross‐link position was defined as the nucleotide upstream of the 5′ end of the read. For analysis of cross‐linking to rRNA, cross‐linking signal was normalized to the total number of rRNA cross‐links per sample. Proportional cross‐link density was defined as the normalized cross‐links per nucleotide for each rRNA subtype.

#### Orthogonal organic phase separation

Orthogonal organic phase separation was carried out as described in Queiroz *et al* (2019), with some modifications. Briefly, 10^6^ heavy or light labeled iKras PDAC cells were seeded onto 10 cm dishes and grown for 48 h without Dox, before the addition of Dox (1 μg/ml) to one label, while leaving the other label unchanged. Cells were incubated for another 24 h before being washed with ice‐cold PBS and irradiated on ice with 400 mJ/cm^2^ of UV‐C (254 nm), using a Hoefer Scientific UV Crosslinker. Cells were then lysed by direct addition of TRIzol reagent (Thermo) to each dish (1 ml). After scraping the cells in TRIzol, the lysate from heavy and light SILAC labels was combined and homogenized through pipetting before incubating at RT for 5 min in order to dissociate non‐cross‐linked proteins. 200 μl of chloroform per 1 ml of TRIZol was then added to the mix, followed by a second homogenization through vortexing. Phase separation was achieved by centrifugation at 12,000 *g* for 15 min at 4°C. After removing the organic and the aqueous phases, the interface was further purified for three additional times by redissolving in TRIZol and repeating chloroform phase separation. Any residual TRIZol was then removed by washing the interface two times with Methanol. To extract the RNA‐bound proteins, the interface was solubilized in 200 μl of RBP buffer (1% SDS, 100 mM TEAB, 1 mM MgCl_2_) for 20 min at 95°C. The RNA component was digested by the addition of 4 μg of RNase A/T1 Mix (Thermo) for 3 h at 37°C, followed by the addition of another 4 μg of RNase A/T1 Mix and incubation at 37°C overnight. The organic phase was then collected after performing an additional TRIZol/chloroform phase separation. Acetone precipitation was then performed on the collected organic phases to precipitate the purified proteins. To extract the protein‐bound RNAs, the interface was resuspended in 200 μl of PBR buffer (1%SDS, 30 mM Tris–HCl (pH 8), 10 mM EDTA), and the protein component was digested by the addition of 18 U of RNA‐grade Proteinase K (Thermo) for 2 h at 50°C. The aqueous phase was then collected after performing an additional TRIZol/chloroform phase separation. Isopropanol precipitation, followed by an ethanol (75%) wash, was then performed on the aqueous phase to precipitate the purified RNA. The purified RNA was resuspended in nuclease‐free water and analyzed by Capillary Electrophoresis on a TapeStation 4200 instrument, using Agilent High Sensitivity RNA ScreenTape assays, according to the manufacturers' protocol.

#### SILAC labeling

Cells were SILAC labeled by being passaged for at least six doublings in lysine‐ and arginine‐free DMEM, supplemented with 10% dialyzed FBS, 1% P/S, 600 mg/l Proline, in the presence of 100 mg/l of either light Arginine and Lysine (for “light” media), or heavy Arginine [U‐13C6, U‐15N4] and Lysine [U‐13C6, U‐15N2] (for “heavy” media). Successful label incorporation was checked prior to experiments by calculating the percentage of heavy or light labeled peptides from tryptic digests of each cell population and was ensured to be > 95%. Experiments were always performed in duplicates, with reciprocal SILAC labeling.

#### Mass spectrometry sample preparation and data acquisition

For OOPS, acetone‐precipitated proteins were subjected to in‐solution digestion. Briefly, proteins were recovered in 200 μl 2 M Urea, 50 mM Ammonium Bicarbonate (ABC) and reduced by adding DTT to a final concentration of 10 mM. After 30 min of incubation at RT, samples were alkylated by adding 55 mM iodoacetamide and incubation for another 30 min at RT in the dark. Trypsin digestion was then performed using 2 μg of trypsin/sample. The next day, samples were desalted using the Stage Tip procedure (Rappsilber *et al*, 2003) and recovered in 0.1% TFA, 0.5% Acetic Acid, 2% Acetonitrile (A* buffer) for MS analysis. For total and phospho‐proteome analyses, lysates, prepared in 2–4% SDS, 100 mM Tris/HCl pH 7.5, were sonicated with a sonicator bath (Bioruptor Pico—Rm 343) for 10 cycles and reduced with the addition of 100 mM DTT and boiling at 95°C for 10 min. For SILAC samples, filter‐aided sample preparation (FASP; Wisniewski *et al*, 2009) was performed, as described previously (Dermit *et al*, 2020). For TMT samples, isobraric filter‐aided sample preparation (iFASP; McDowell *et al*, 2013) was performed. Briefly, 25 μg (for total proteomics) or 100 μg (for phospho‐proteomics) of each total lysate was reduced with 50 mM Bond‐Breaker TCEP Solution (Thermo) by boiling at 95°C for 10 min. Reduced samples were then diluted in UA buffer (8 M urea, 100 mM Tris–HCl pH 8.8) and transferred to Vivacon 500 Hydrosart filters with a molecular cutoff of 30 kDa, before being concentrated by centrifugation at 14,000 *g* for 20 min. Samples were then washed once with UA buffer through buffer addition to the filter top and concentration, before alkylation with 55 mM iodoacetamide in UA buffer at RT for 30 min in the dark. Samples were then washed three additional times with the UA buffer, before three washes with 100 mM TEAB to reduce the urea concentration. Samples were then trypsin digested overnight at 37°C in a 600 rpm shaking thermomixer, by adding 100 μl of 100 mM TEAB supplemented with 25 ng Trypsin per 1 μg of input protein. Next day, TMT 6plex or 10plex label reagents were thawed and dissolved in acetonitrile. Each Sample was then supplemented with 8 μg of TMT label per 1 μg of input protein and incubated for 1 h at 25°C, followed by quenching with 5% hydroxylamine for 30 min at 25°C. Peptides were then eluted by centrifugation at 14,000 g. Two additional elutions were then performed by adding 40 μl of TEAB and centrifugation, plus a final elution with 40 μl of 30% acetonitrile. After combining all individually labeled eluates into one, the pooled mixture was dried with a vacuum concentrator. For SILAC and TMT total proteomics analyses, samples were fractionated into seven fractions using Pierce™ High pH reverse‐phase fractionation kit, according to the manufacturer's instructions. Fractions were then dried with vacuum centrifugation before LC–MS/MS analysis. For phospho‐proteomics, samples were subjected to TiO phospho‐peptide enrichment using GL Sciences TiO enrichment kit, according to the manufacturer's instructions. LC–MS/MS analysis was performed on a Q Exactive‐plus Orbitrap mass spectrometer coupled with a nanoflow ultimate 3000 RSL nano HPLC platform (Thermo Fisher). Dried peptide mixtures were resuspended in A* buffer. For total proteomics analysis, equivalent of ~1 μg of protein was injected into the nanoflow HPLC. For OOPS and phospho‐proteomics analysis, ~90% of the total peptide mixture was injected. Samples were resolved at flow rate of 250 nl/min on an Easy‐Spray 50 cm × 75 μm RSLC C18 column (Thermo Fisher). Each run consisted of a 123 min gradient of 3% to 35% of Buffer B (0.1% FA in Acetonitrile) against Buffer A (0.1% FA in LC–MS gradient water), and separated samples were infused into the MS by electrospray ionization (ESI). Spray voltage was set at 1.95 kV, and capillary temperature was set to 255°C. MS was operated in data‐dependent positive mode, with 1 MS scan followed by 15 MS2 scans (top 15 methods). Full‐scan survey spectra (m/z 375–1,500) were acquired with a 70,000 resolution for MS scans and 17,500 for the MS2 scans. For TMT10plex samples, MS2 scans were acquired with 35,000 resolution. A 30‐s dynamic exclusion was applied.

#### Mass spectrometry data analysis

MaxQuant (version 1.6.3.3) was used for all mass spectrometry search and quantifications (Tyanova *et al*, 2016a). Raw data files were searched against a FASTA file of the Mus musculus proteome, extracted from Uniprot (2016). Enzyme specificity was set to “Trypsin,” allowing up to two missed cleavages. False discovery rates (FDR) were calculated using a reverse database search approach and were set at 1%. Default MaxQuant parameters were used with some adjustments: For TMT experiments, “reporter ion MS2” type option was selected with a reporter mass tolerance of 0.003 Da. For SILAC experiments, “Match between runs” and the “Re‐quantify” options were enabled. A minimum ratio count of 2 was also used for protein identifications. All downstream data analyses, such as data filtering, log transformation, ratio calculation, data normalization, *t*‐test analysis, category annotation, 1D & 2D annotation enrichment analysis, Fisher's exact test analysis, and data visualizations, were performed in Perseus software (version 1.6.2.3) (Tyanova *et al*, 2016b). For one‐sample *t*‐test analyses, *P*‐value correction via Benjamini–Hochberg FDR calculation, with a cutoff of < 0.05 was used (Tyanova *et al*, 2016b). For two‐sample *t*‐test analyses, a permutation‐based FDR calculation with an S0 of 0.1, and an FDR cutoff of < 0.05 was used (Tyanova *et al*, 2016b). Category enrichment analyses were performed using Fisher's exact test. 1D and 2D annotation enrichment analyses were performed using an adapted Wilcoxon Mann–Whitney test. *P*‐value correction using Benjamini–Hochberg FDR calculation with a cutoff of < 0.02 was applied to both types of enrichment analyses (Tyanova *et al*, 2016b).

#### Mouse orthotopic xenograft assay

All xenograft experiments were performed under Home Office UK Project License (PP9448177), protocol 3, after internal review board approval. For orthotopic establishment of PDAC tumors, 5 × 10^5^ iKras PDAC cells, suspended in 20 μl of 50% Matrigel (BD Biosciences)/Hanks buffered saline solution, were injected into the pancreas of NCr nude mice. Anesthetic machine with Isoflurane for induction and duration of the procedure was used during the surgery. As analgesic, animals were given Vetergesic (buprenorphine) subcutaneous into scruff before the surgery, later in the day, and the next morning. All animals were observed and examined for any abnormalities, and body weight was measured daily. To maintain Kras^G12D^ expression, animals were fed with water supplemented with 2 g/l of Dox and 20 g/l of Sucrose. CX‐5461, resuspended in 50 mM sodium phosphate solution (pH 4.5) at 10 mg/ml, was administered via daily oral gavage, at a final dose of 50 mg/kg. *In vivo* tumor imaging was performed using a fast 3 min T2 weighted MRI scan on a Bruker ICON 1T MRI system instrument, at the indicated time points. An anesthetic rig was used to immobilize the mice by administering isoflurane into an induction box and keeping them unconscious on the MRI bed for imaging.

#### Tissue staining

All tissue stainings were performed by the BCI Histophatology core facility. Hematoxylin & Eosin (H&E) staining of formalin fixed paraffin embedded tissue sections was performed using the Leica Autostainer XL (V2.01). For Immunohistochemistry (IHC), a Ventana Discovery XT instrument was used for the deparaffinization, heat‐induced epitope retrieval, and blocking steps. Ph2AX antibody was initially tested on cisplatin‐treated and untreated iKras PDAC cells embedded in agarose, and an optimal dilution of 1:400 was determined for the detection of DNA damage cells by IHC staining. Primary and secondary OmniMap HRP‐conjugated antibodies were used in 100 μl volumes, for 60 and 16 min, respectively. Slides were then stained by applying one drop of DAB CM and One Drop H_2_O_2_ CM and incubating for 8 min, then applying one drop of Copper CM and incubating for 5 min. Slides were then counterstained with Hematoxylin, and post‐counterstaining with Bluing reagent was performed, before washing with warm water with detergent, and dehydrating in graded ethanol and xylene. Slides were then covered by glass coverslips, attached with permanent mounting media.

#### Statistical analysis

Statistical analyses of the proteomics data were performed using Perseus (version 1.6.2.3) (Tyanova *et al*, 2016b), as described previously. All other statistical analyses were performed using GraphPad PRISM (version 9). For direct comparisons against set a control, unpaired Student's *t*‐test was used. For comparisons of multiple conditions, one‐way or two‐way ANOVA was used, as described in each figure legend. Standard deviation (SD) was used for all error bars.

## Author contributions

**Muhammad S Azman:** Data curation; formal analysis; validation; investigation; visualization; methodology. **Emilie L Alard:** Data curation; formal analysis; validation; investigation; visualization; methodology. **Martin Dodel:** Data curation; formal analysis; validation; investigation; visualization; methodology. **Federica Capraro:** Validation; investigation; methodology. **Rupert Faraway:** Formal analysis; visualization. **Maria Dermit:** Investigation; methodology. **Wanling Fan:** Investigation. **Alina Chakraborty:** Investigation. **Jernej Ule:** Formal analysis; supervision. **Faraz K Mardakheh:** Conceptualization; formal analysis; supervision; funding acquisition; validation; writing – original draft; project administration; writing – review and editing.

## Disclosure and competing interests statement

The authors declare that they have no conflict of interest.

## Supporting information



Expanded View Figures PDF

Dataset EV1

Dataset EV2

Dataset EV3

Dataset EV4

Dataset EV5

Dataset EV6

Dataset EV7

Dataset EV8

Dataset EV9

Dataset EV10

Dataset EV11

Dataset EV12

Dataset EV13

Dataset EV14

PDF+

Source Data for Figure 2

Source Data for Figure 3

Source Data for Figure 4

Source Data for Figure 5

Source Data for Figure 6

## Data Availability

All mass spectrometry raw files and their associated MaxQuant output files were deposited on ProteomeXchange Consortium (Vizcaino *et al*, 2014) via the PRIDE partner repository (http://www.ebi.ac.uk/pride/archive/), under the accession numbers PXD030825 (http://proteomecentral.proteomexchange.org/cgi/GetDataset?ID=PXD030825), PXD030893 (http://proteomecentral.proteomexchange.org/cgi/GetDataset?ID=PXD030893), PXD038338 (http://proteomecentral.proteomexchange.org/cgi/GetDataset?ID=PXD038338), PXD038439 (http://proteomecentral.proteomexchange.org/cgi/GetDataset?ID=PXD038439), PXD038440 (http://proteomecentral.proteomexchange.org/cgi/GetDataset?ID=PXD038440), PXD038441 (http://proteomecentral.proteomexchange.org/cgi/GetDataset?ID=PXD038441), and PXD038496 (http://proteomecentral.proteomexchange.org/cgi/GetDataset?ID=PXD038496). All iCLIP FASTQ files were deposited to the ArrayExpress database (http://www.ebi.ac.uk/arrayexpress), under the accession number E‐MTAB‐12481 (http://www.ebi.ac.uk/arrayexpress/experiments/E‐MTAB‐12481).
