# Supplementary material for: An ERK1/2‐driven RNA‐binding switch in nucleolin drives ribosome biogenesis and pancreatic tumorigenesis downstream of RAS oncogene
Source: EMBO J. 2023 Apr 11;42(11):e110902. doi: 10.15252/embj.2022110902 (PMC10233377; doi:10.15252/embj.2022110902)
Supplement: Supplementary file 1 — Expanded View Figures PDF [file EMBJ-42-e110902-s001.pdf]

# Expanded View Figures

**Figure EV1. Kras$^{G12D}$ reshapes the RBpome of PDAC cells.**

A Dox removal results in loss of Kras$^{G12D}$ expression and ERK activity in iKras PDAC cells. Cells were grown in the presence or absence of Dox for the indicated amounts of time, before being subjected to lysis and immunoblotting (IB) with the indicated antibodies.

B Quantification of phospho / total Erk1/2 ratio values from (A), as a measure of Erk1/2 kinase activity. A total of three independent biological replicate experiments were quantified. Error bars depict SD (*$P < 0.05$—calculated from unpaired *t*-test).

C Addition of Dox to Dox-withdrawn cells results in induction of Kras$^{G12D}$ expression and ERK activity in iKras PDAC cells. Cells were grown in the absence of Dox for 48 h, before its addition for the indicated amounts of time. Cells were then subjected to lysis and immunoblotting with the indicated antibodies.

D Quantification of phospho / total Erk1/2 ratio values from (C), as a measure of Erk1/2 kinase activity. A total of three independent biological replicate time course experiments were quantified (****$P < 0.0001$—calculated from one-way ANOVA).

E OOPS-mediated enrichment of proteins in the interface is dependent on UV-C cross-linking. IKras PDAC cells were treated with or without UV-C cross-linking, before lysis in TRIzol and OOPS analysis as in (Queiroz *et al*, 2019). Interface proteins were then extracted and subjected to mass spectrometry analysis. A total of two biological replicates per condition were analyzed, and the total number of proteins identified in both replicates for each condition were plotted. Enrichment of proteins in the interface was boosted by > 400% upon UV-C cross-linking.

F OOPS specifically enriches RNA-binding proteins in the interface, following UV-C cross-linking. Fisher's exact test analysis of enriched protein categories in the interface of UV-C cross-linked samples from (E) (FDR < 0.02). Each data point represents a category from Gene Ontology (GO) and Kyoto Encyclopedia of Genes & Genome (KEGG) databases, with functionally similar categories highlighted with the same colors. Conventional as well as nonconventional RBPs are significantly enriched in the interface of UV-C cross-linked samples.

G Volcano plot of changes in the RBPome following Kras$^{G12D}$ induction. RBPome changes were quantified, as described in Fig 1A, from six independent biological replicate qRIC experiments (Dataset EV1), using a one-sample *t*-test analysis. Seventy-four proteins were upregulated in the RNA-bound fraction (red), while 109 showed a significant decrease (blue) (FDR < 0.05).

H Fisher's exact test analysis of protein categories that are over-represented among the upregulated RBPome (FDR < 0.02). Each data point represents a category from GO and KEGG databases, with functionally similar categories highlighted with the same colors (Dataset EV2).

I Fisher's exact test analysis of protein categories that are over-represented among the downregulated RBPome (FDR < 0.02). Each data point represents a category from GO and KEGG databases, with functionally similar categories highlighted with the same colors (Dataset EV3).

J Trametinib inhibits Kras$^{G12D}$-induced ERK activity in iKras PDAC cells. Cells were grown in the absence of Dox for 48 h, before its addition to the indicated cells for 24 h, with or without 10 or 50 nM Trametinib. Cells were then subjected to lysis and immunoblotting with the indicated antibodies.

K Quantification of phospho / total Erk1/2 ratio values from (J), as a measure of Erk1/2 kinase activity. A total of three independent biological replicate experiments were quantified. Error bars depict SD (*$P < 0.05$; n.s.: not significant—calculated from unpaired *t*-test).

L Volcano plot of Kras$^{G12D}$-driven changes in the RBPome in the presence of Trametinib (10 nM). RBPome changes in the presence of Trametinib were quantified from four independent qRIC experiments, using a one-sample *t*-test analysis (FDR < 0.05), with significantly increased (red) or decreased (blue) proteins from (G) highlighted on the plot (Dataset EV4).

M Analysis of the Kras$^{G12D}$-induced change in the RNA-binding activity of endogenous Ncl. IKras PDAC cells were grown in the absence of Dox for 48 h, before its addition to the indicated cells for a further 24 h to induce Kras$^{G12D}$ expression. Cells were then subjected to OOPS in order to isolate the interface (RNA-bound proteins), or whole cell lysis (total lysate), followed by immunoblotting with anti-Ncl antibody. For comparison, Gapdh, which also binds RNA but does not show a change in its RNA-binding activity (Dataset EV1), was also blotted for. In parallel, a fraction of each interface or total lysate sample was subjected to RNA extraction, which was resolved and quantified by capillary electrophoresis (CE) as loading control.

N Quantification of normalized RNA-bound to total Ncl ratio values from (M), as a measure its RNA-binding activity. A total of three biological replicate experiments were quantified. Error bars depict SD (*$P < 0.05$—calculated from unpaired *t*-test).

 © 2023 The Authors

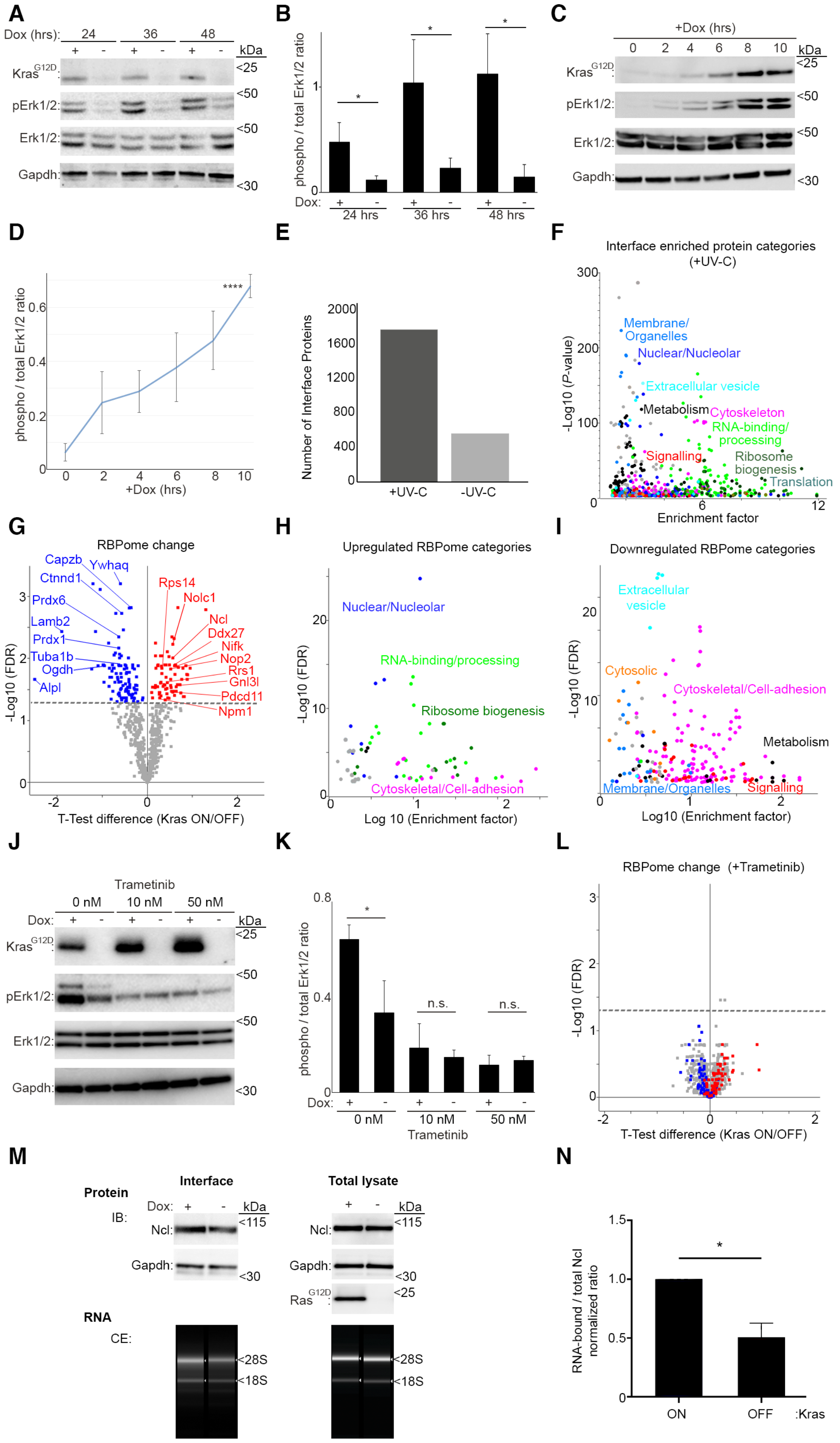
A
Dox (hrs): 24 36 48
kDa
KrasG12D: <25
pErk1/2: <50
Erk1/2: <50
Gapdh: <30
B
phospho / total Erk1/2 ratio
Dox:
24 hrs 36 hrs 48 hrs
C
+Dox (hrs)
0 2 4 6 8 10
kDa
KrasG12D <25
pErk1/2: <50
Erk1/2: <50
Gapdh: <30
D
phospho / total Erk1/2 ratio
+Dox (hrs)
E
Number of Interface Proteins
+UV-C -UV-C
F
Interface enriched protein categories (+UV-C)
Membrane/ Organelles
Nuclear/Nucleolar
Extracellular vesicle
Metabolism
Cytoskeleton
RNA-binding/ processing
Signalling
Ribosome biogenesis
Translation
-Log10 (P-value)
Enrichment factor
G
RBPome change
Capzb Ywhaq Ctnnd1 Prdx6 Lamb2 Prdx1 Tuba1b Ogdh Alpl
Rps14 Nolc1 Ncl Ddx27 Nifk Nop2 Rrs1 Gnl3l Pdcd11 Npm1
-Log10 (FDR)
T-Test difference (Kras ON/OFF)
H
Upregulated RBPome categories
Nuclear/Nucleolar
RNA-binding/processing
Ribosome biogenesis
Cytoskeletal/Cell-adhesion
-Log10 (FDR)
Log 10 (Enrichment factor)
I
Downregulated RBPome categories
Extracellular vesicle
Cytosolic
Cytoskeletal/Cell-adhesion
Metabolism
Membrane/Organelles
Signalling
-Log10 (FDR)
Log10 (Enrichment factor)
J
Trametinib
0 nM 10 nM 50 nM
Dox:
kDa
KrasG12D <25
pErk1/2: <50
Erk1/2: <50
Gapdh: <30
K
phospho / total Erk1/2 ratio
n.s. n.s.
Dox:
0 nM 10 nM 50 nM
Trametinib
L
RBPome change (+Trametinib)
-Log10 (FDR)
T-Test difference (Kras ON/OFF)
M
Protein
Interface
Total lysate
IB:
Dox:
kDa
Ncl: <115
Gapdh: <30
RasG12D: <25
RNA
CE:
<28S
<18S
N
RNA-bound / total Ncl normalized ratio
ON OFF :Kras

**Figure EV1.**

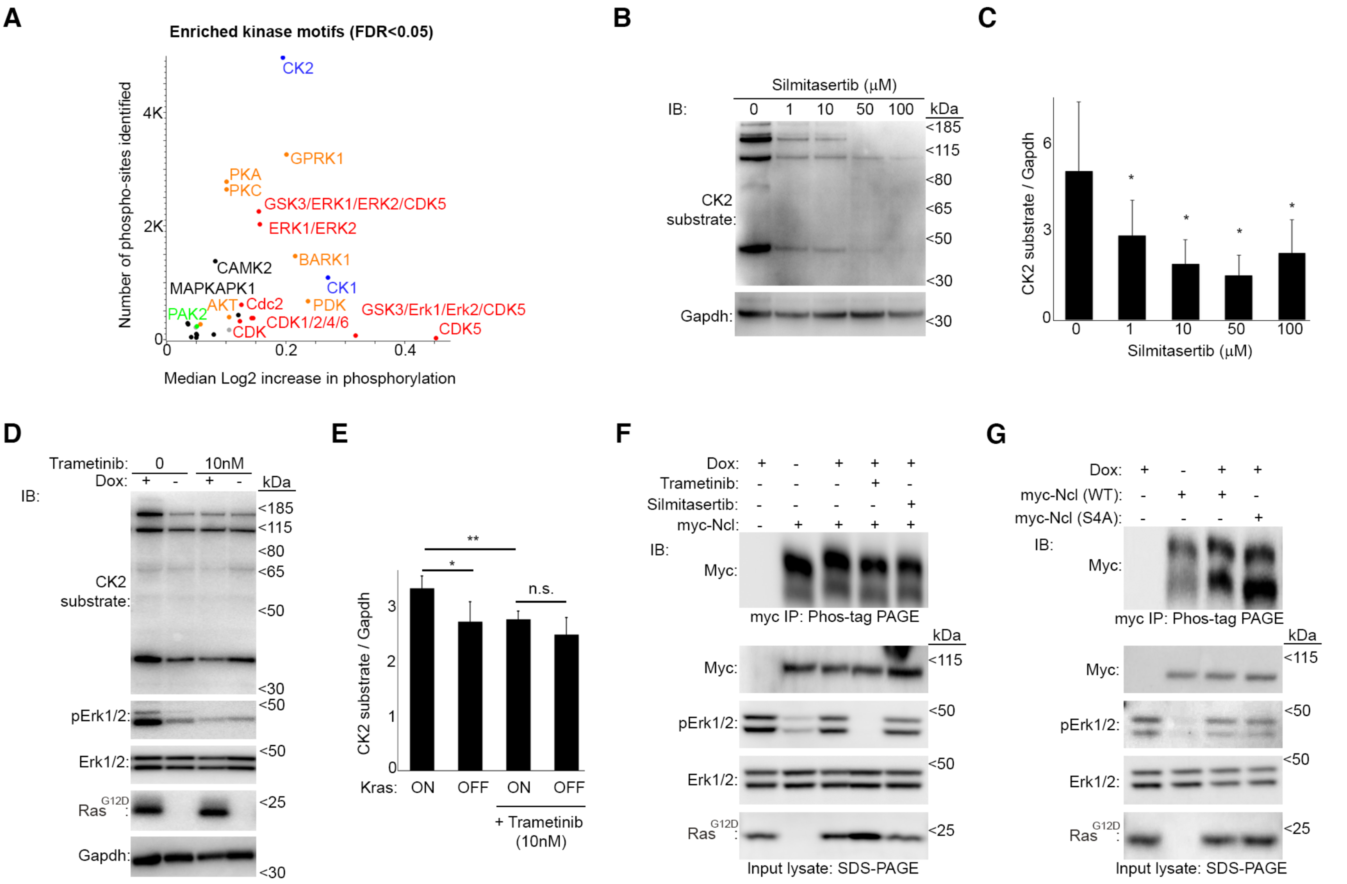


**Figure EV2. Kras$^{G12D}$ activates CK2 to phosphorylate Ncl.**

A Motif analysis of phosphorylation changes in iKras PDAC cells in response to Kras$^{G12D}$ induction. Phospho to total proteomic changes from Fig 2A were annotated for different kinase target linear phospho-motifs in Perseus (Tyanova *et al*, 2016b), and subjected to 1D annotation enrichment test (FDR < 0.02). Kras$^{G12D}$-induced median shift in the phosphorylation intensity was plotted against the total number of the sites for each indicated motif (Dataset EV9). Colors indicate the major kinase family groups (red: Proline-directed kinases; blue: Casein kinases; orange: AGC kinases; black: $Ca^{2+}$/Calmodulin-dependent kinases; green: STE kinases). In addition to the ERK1/2 substrate motif, motifs for substrates of several other kinases such as CDKs, CK1, CK2, and GPRK/β-ARK kinases were significantly enriched among the Kras$^{G12D}$-induced phosphorylations.

B A CK2 phospho-substrate antibody mix can be used as an indicator of CK2 activity. IKras PDAC cells grown in the presence of Dox were treated overnight with the indicated concentrations of Silmitasertib, a specific CK2 inhibitor (Chon *et al*, 2015), before being subjected to lysis and immunoblotting with the indicated antibodies.

C Quantification of normalized CK2 phospho-substrate levels from (B), as a measure of CK2 kinase activity. A total of three independent biological replicate experiments were quantified. Error bars depict SD. Significance was calculated relative to untreated control (*$P$ < 0.05—calculated from unpaired $t$-test).

D Kras$^{G12D}$ induction enhances CK2 activity in an Erk1/2-dependent manner. IKras PDAC cells were grown in the absence of Dox for 48 h, before its addition to the indicated cells, with or without Trametinib (10 nM), for a further 24 h. Cells were then lysed and analyzed by immunoblotting with the indicated antibodies.

E Quantification of normalized CK2 phospho-substrate levels from (D), as a measure of CK2 kinase activity. A total of three independent biological replicate experiments were quantified. Error bars depict SD (**$P$ < 0.01; *$P$ < 0.05; n.s.: not significant—calculated from unpaired $t$-test).

F Kras$^{G12D}$ induction slows the migration of myc-Ncl through the Phos-tag gel in an Erk1/2- and CK2-dependent manner. Myc-Ncl transfected iKras PDAC cells were grown in the absence of Dox for 48 h, before its addition to the indicated cells for a further 24 h, with or without Trametinib (10 nM) or Silmitasertib (10 μM). Cells were lysed and subjected to immunoprecipitation with anti-Myc tag antibody, and the immunoprecipitates were resolved by Phos-tag SDS–PAGE, followed by immunoblotting with the anti-Myc antibody. In parallel, input lysates were resolved using standard SDS–PAGE, and immunoblotted with the indicated antibodies. The blots are representative results from three independent biological replicate experiments.

G Kras$^{G12D}$-induced retardation of myc-Ncl migration through the Phos-tag gel is dependent on S28, S34, S40, and S41. Control, wild-type (WT), and phospho-defective (S4A) mutant Myc-Ncl transfected iKras PDAC cells were grown in the absence of Dox for 48 h, before its addition to the indicated cells for 24 h. Cells were lysed and subjected to immunoprecipitation with anti-Myc tag antibody, and the immunoprecipitates were resolved by Phos-tag SDS–PAGE, followed by immunoblotting with the anti-Myc antibody. In parallel, input lysates were resolved using standard SDS–PAGE, and immunoblotted with the indicated antibodies. The blots are representative results from two independent biological replicate experiments.

 © 2023 The Authors

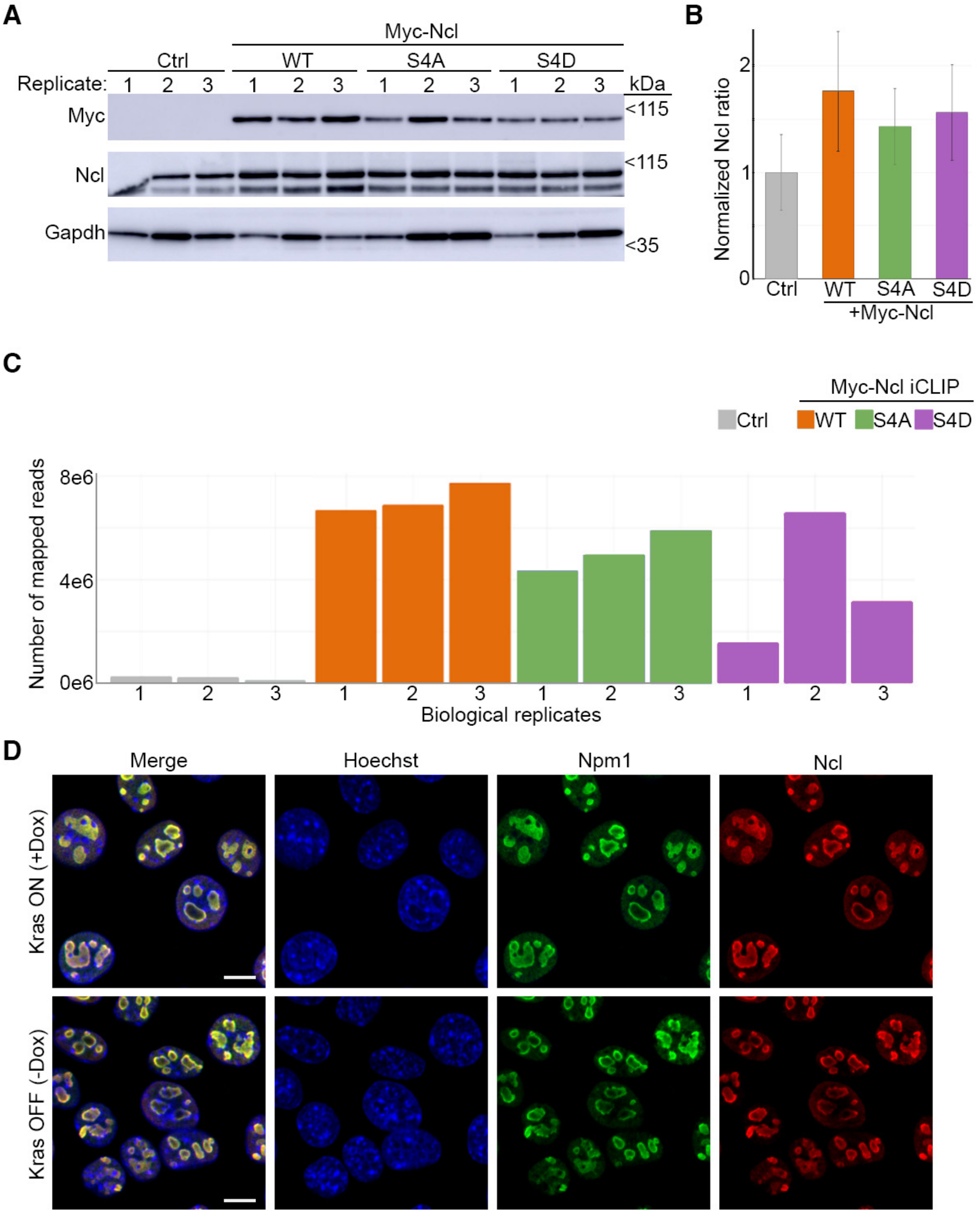


**Figure EV3. Ncl iCLIP quality-control and localization analysis.**

A Assessment of the expression levels of endogenous and ectopic WT, S4A, and S4D Ncl, in iKras PDAC cells that were subjected to iCLIP analysis. IKras PDAC cells were transfected with constructs encoding WT, S4A, and S4D Myc-Ncl, or mock-transfected as negative control, before being seeded and grown for 48 h. Cells were then UV-C irradiated, lysed, and subjected to iCLIP analysis. Aliquots of the iCLIP input lysates from three independent biological replicates were analyzed by immunoblotting with the indicated antibodies in parallel.

B Quantification of the relative normalized levels of Ncl antibody signal in the Ctrl vs. Myc-Ncl expressing cells from (A). Myc-Ncl transfected cells exhibit total Ncl levels that are around 50% more than those of the Ctrl cells. A total of three independent biological replicates per condition were analyzed.

C Ncl-bound RNAs are specifically identified in Myc-Ncl iCLIP experiments. Plot of the total number of mapped reads in each replicate of Ctrl vs. Myc-Ncl iCLIP sequencing results. Few reads were identified in the iCLIP sequencing results of Ctrl, as opposed to Myc-Ncl expressing cells.

D Endogenous Ncl is exclusively localized to the Nucleolus of iKras PDAC cells, irrespective of Kras$^{G12D}$ expression. Cells were grown in the absence of Dox for 48 h, before its addition to the indicated cells for 24 h. Dox-treated and untreated cells were subsequently fixed and immunostained with an anti-Ncl antibody (red), an anti-Npm1 antibody as a Nucleolar marker (green), and Hoechst (blue) as the Nuclear stain, followed by confocal microscopy analysis. Scale bar = 10 μm.

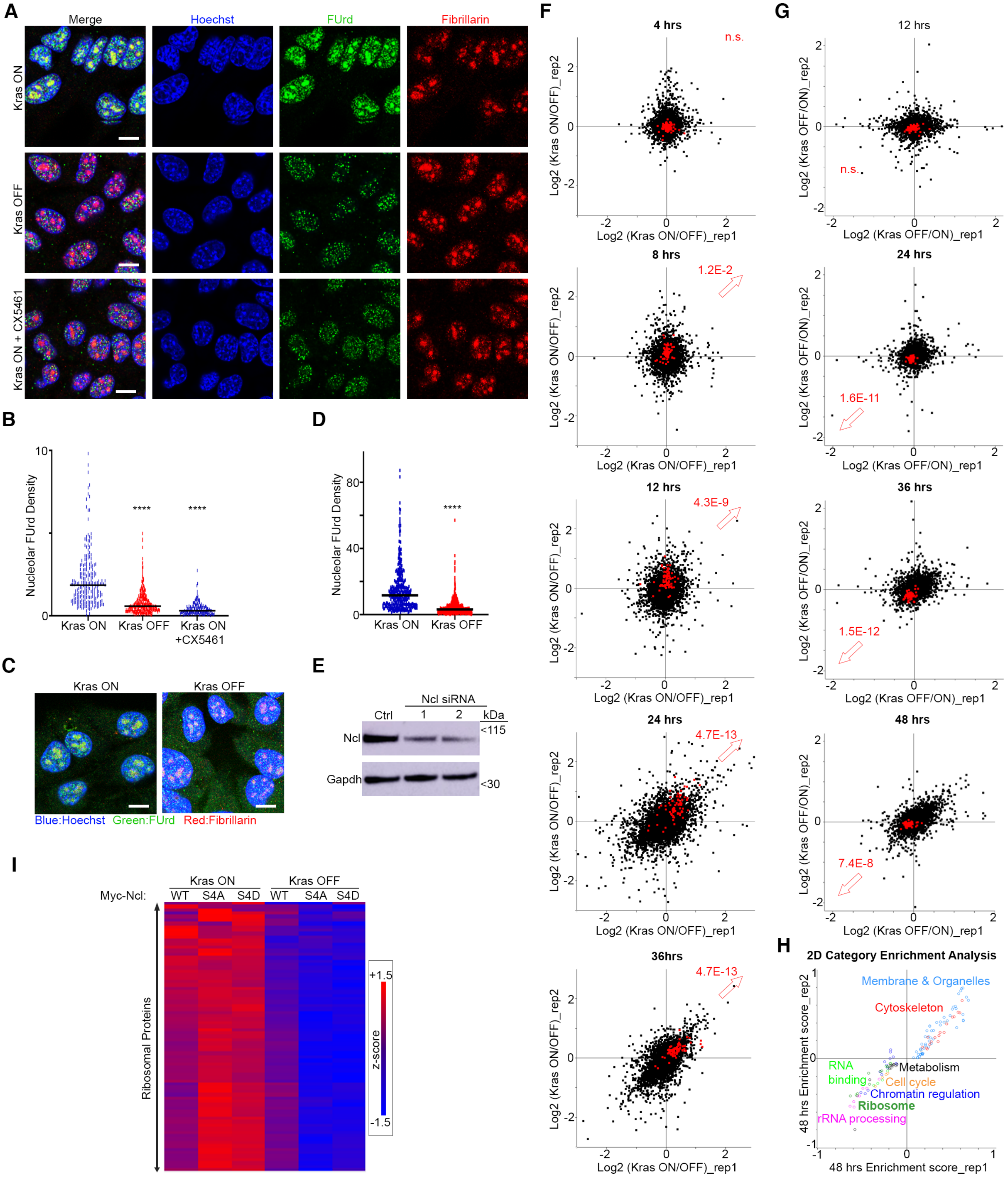


**Figure EV4.**

 © 2023 The Authors

**Figure EV4. Kras$^{G12D}$ promotes rRNA synthesis and ribosome biogenesis.**

A Induction of Kras$^{G12D}$ expression triggers nascent pre-rRNA synthesis in iKras PDAC cells. Cells were grown for 48 h in the absence of Dox. Kras$^{G12D}$ expression was then induced in the indicated cells (Kras ON) by the addition of Dox for a further 24 h. The rRNA polymerase-I inhibitor (CX-5461) was added to the indicated cells for 30 min, before all conditions were subjected to pulse labeling with FUrd to visualize RNA synthesis. Cells were then fixed and immunostained with anti-FUrd antibody (green) to visualize nascent RNA, along with anti-Fibrillarin (Fbl) antibody as a Nucleolar marker (red), and Hoechst (blue) as the Nuclear stain, followed by confocal microscopy analysis. Kras$^{G12D}$ induction results in accumulation of nascent RNA in the Nucleoli of iKras PDAC cells, in an RNA polymerase-I-dependent manner. Scale bar = 10 μm.

B Quantification of Nucleolar FUrd levels from (A). FUrd fluorescence densities in single nucleoli were quantified from 157 to 289 individual cells per condition, combined from two independent biological replicate experiments. Significance was calculated relative to the Kras ON condition (****$P < 0.0001$—calculated from unpaired *t*-test).

C Removal of Kras$^{G12D}$ expression results in loss of nascent pre-rRNA synthesis in iKras PDAC cells. Cells were grown for 48 h in the presence or absence of Dox, before pulse labeling with FUrd to visualize RNA synthesis. Cells were fixed and immunostained with anti-FUrd antibody (green) to visualize nascent RNA, along with anti-Fibrillarin (Fbl) antibody as a Nucleolar marker (red), and Hoechst (blue) as the Nuclear stain, followed by confocal microscopy analysis. Loss of Kras$^{G12D}$ expression results in abrogation of nascent RNA accumulation in the Nucleoli. Scale bar = 10 μm.

D Quantification of Nucleolar FUrd levels from (C). FUrd fluorescence densities in single nucleoli were quantified from 147 to 196 individual cells per condition, combined from two independent biological replicate experiments (****$P < 0.0001$—calculated from unpaired *t*-test).

E Validation of siRNA-mediated depletion of Ncl in iKras PDAC cells. Cells were transfected with a nontargeting control siRNA, or two independent siRNAs against Ncl, before being lysed and analyzed by immunoblotting with the indicated antibodies.

F Induction of Kras$^{G12}$ expression results in accumulation of ribosomal proteins (RPs). IKras PDAC cells were grown in the absence of Dox for 48 h, before its addition to the cells for the indicated amounts of time (Kras ON), or leaving the cells untreated for the same period as control (Kras OFF). Cells were subsequently lysed and subjected to TMT-mediated quantitative proteomics (Dataset EV10). Log2 of Kras ON/Kras OFF protein ratio values from two biological replicate experiments were plotted for each time point, with the ratio values of RPs marked in red. Benjamini–Hochberg-corrected *P*-values of the increase in RPs ratio values are reported on each graph (n.s.: not significant).

G Loss of Kras$^{G12}$ expression results in depletion of RPs. IKras PDAC cells were seeded and grown in the presence (Kras ON) or absence (Kras OFF) of Dox for the indicated amounts of time, before lysis and TMT-mediated quantitative proteomics (Dataset EV11). Log2 of Kras ON/Kras OFF protein ratio values from two biological replicate experiments were plotted for each time point, with the ratio values of RPs marked in red. Benjamini–Hochberg-corrected *P*-values of the decrease in RPs ratio values are reported on each graph (n.s.: not significant).

H 2D-annotation enrichment analysis of the 48-h time point data from (G). Each data point represents a functional category from GO and KEGG databases, with similar categories highlighted with the same colors (Dataset EV12). After loss of Kras$^{G12}$ for 48 h, protein categories related to Ribosome and rRNA processing exhibit significant downregulation, while those related to cytoskeleton and membranous organelles show upregulation (FDR < 0.02).

I TMT quantitative analysis of RP levels in WT, S4A, and S4D Myc-Ncl expressing iKras PDAC cells, in the presence or absence of Kras$^{G12D}$. Vectors encoding Myc-tagged WT, S4A, and S4D Ncl were transiently transfected into iKras PDAC cells, before reseeding and growing the cells for 48 h in the presence or absence of Dox. Cells were then lysed and analyzed by TMT-mediated quantitative mass spectrometry. *Z*-scores of TMT intensity changes for all the identified RPs across the different conditions were plotted as a heat map (red → increase; blue → decrease).

© 2023 The Authors 

**Figure EV5. CX-5461 does not induce DNA damage at low nanomolar doses, irrespective of Kras$^{G12D}$-induced cell proliferation.**

A Kras$^{G12D}$ enhances iKras PDAC cell proliferation in 2D cell culture. IKras PDAC cells were seeded and subjected to clonogenic assay for 7 days, in the presence (Kras ON) or absence (Kras OFF) of Dox. Colonies were visualized by Crystal Violet staining.

B Quantification of Crystal Violet staining levels from (A). A total of 12 biological replicate experiments were quantified. Error bars depict SD (****$P < 0.0001$—calculated from unpaired *t*-test).

C Kras$^{G12D}$ enhances iKras PDAC cell proliferation in 3D cell culture. IKras PDAC cells were seeded onto 3D Collagen-I gels, with (Kras ON) or without (Kras OFF) Dox, and allowed to grow for 48 h. Cells were subsequently imaged live by phase contrast microscopy. Scale bar = 200 μm.

D Analysis of the percentage of viable cells in 3D cultures of (C). Cells were subjected to luminescence-based viability assay by CellTiter-Glo to quantify the relative percentage of viable cells. A total of six biological replicate experiments were quantified. Error bars depict SD (****$P < 0.0001$—calculated from unpaired *t*-test).

E Analysis of the impact of ectopic expression of Ncl or its phospho-mutants on iKras PDAC cell proliferation. IKras PDAC cells were seeded with (Kras ON) or without (Kras OFF) Dox and transfected the next day with myc-tagged wild-type (WT), phospho-defective (S4A), or phospho-mimicking (S4D) mutants of Ncl, along with an empty vector negative control (Ctrl). Percentage of change in the number of viable cells relative to Ctrl was then quantified by CellTiter-Glo assay 48-h post-transfection. A total of four biological replicate experiments were quantified. Error bars depict SD. Significance was calculated relative to each corresponding control (Kras ON or OFF) condition (****$P < 0.0001$; ***$P < 0.001$; **$P < 0.01$; n.s.: not significant—calculated from unpaired *t*-test).

F Kaplan–Meier overall survival analysis of nude mice orthotopically engrafted with iKras PDAC cells (Cohort size = 7). Mice were Dox-fed throughout the analysis. Arrow marks the day of the first mortality event.

G Analysis of Ncl expression in iKras PDAC cells that were used for orthotopic xenograft studies in Fig 5E–G. IKras PDAC cells were transfected with Ctrl and Ncl siRNAs, before injection into the pancreas of nude mice for orthotopic analysis. In parallel, a fraction of the cells from each siRNA treatment were lysed and analyzed by immunoblotting with the indicated antibodies.

H Dose–response analysis of CX-5461 impact on DNA damage. CX-5461 induces DNA damage only at the highest tested dose (1,000 nM). IKras PDAC cells were grown in the absence of Dox for 48 h. Cells were subsequently treated for 24 h with the indicated concentrations of CX-5461, or Cisplatin as positive control, with or without co-addition of Dox to induce Kras expression. Treated cells were then fixed and immunostained with anti-pH2AX (Ser 139) antibody which marks DNA damage foci (red), and Hoechst as a Nuclear stain (blue), followed by confocal microscopy analysis. Scale bar = 10 μm.

I Quantification of nuclear pH2AX signal intensity from (A). Fluorescence density of pH2AX in single nucleoli was quantified from 237 to 570 individual cells per condition, combined from two independent biological replicate experiments. Significance was calculated relative to each corresponding untreated control (Kras ON or OFF) condition (****$P < 0.0001$; n.s.: not significant—calculated from unpaired *t*-test).

J CX-5461 treatment impact on the proteome of iKras PDAC cells mimics Kras$^{G12D}$ removal. IKras PDAC cells grown in the presence of Dox were treated with or without CX-5461 (100 nM) for 48 h, before being lysed and analyzed by TMT-mediated quantitative proteomics (Dataset EV13). CX-5461-induced changes from two independent biological replicate experiments were then subjected to 2D-annotation enrichment analysis. Each data point represents a functional category from GO and KEGG databases, with similar categories highlighted with the same colors (Dataset EV14). Similar to the impact of Kras$^{G12D}$ removal (Fig EV4H), protein categories related to Ribosome and rRNA processing exhibit significant downregulation following 48 h of CX-5461 treatment, while those related to cytoskeleton and membranous organelles show upregulation (FDR < 0.02). No significant change in protein categories related to DNA damage response was detected.

K IHC analysis of tumors from Fig 6I with anti-pH2AX (Ser 139) antibody. No pH2AX signal, indicative of DNA damage, was detectable in tumors from either the control or CX-5461 (50 mg/kg) treated mice. Scale bar = 50 μm.

 © 2023 The Authors

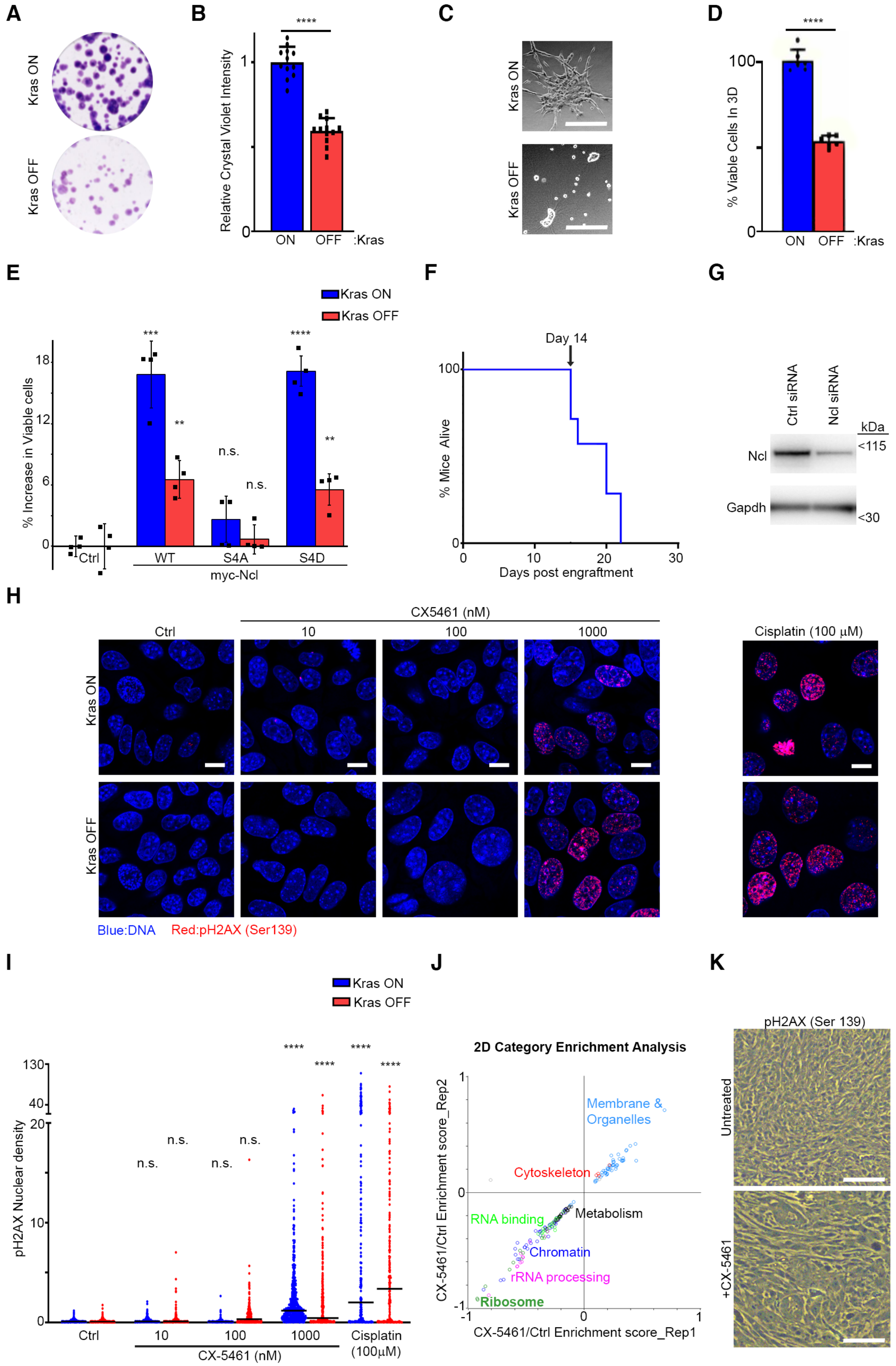
A
Kras ON
Kras OFF
B
Relative Crystal Violet Intensity
ON OFF :Kras
C
Kras ON
Kras OFF
D
% Viable Cells In 3D
ON OFF :Kras
E
Kras ON
Kras OFF
% Increase in Viable cells
Ctrl
WT
S4A
S4D
myc-Ncl
n.s.
F
Day 14
% Mice Alive
Days post engraftment
G
Ctrl siRNA
Ncl siRNA
kDa
Ncl
Gapdh
H
CX5461 (nM)
Ctrl
10
100
1000
Cisplatin (100 μM)
Kras ON
Kras OFF
Blue:DNA
Red:pH2AX (Ser139)
I
Kras ON
Kras OFF
pH2AX Nuclear density
n.s.
Ctrl
CX-5461 (nM)
Cisplatin (100μM)
J
2D Category Enrichment Analysis
CX-5461/Ctrl Enrichment score_Rep2
CX-5461/Ctrl Enrichment score_Rep1
Membrane & Organelles
Cytoskeleton
Metabolism
RNA binding
Chromatin
rRNA processing
Ribosome
K
pH2AX (Ser 139)
Untreated
+CX-5461

**Figure EV5.**
