# Supplementary figures and images for: An ERK1/2‐driven RNA‐binding switch in nucleolin drives ribosome biogenesis and pancreatic tumorigenesis downstream of RAS oncogene
Source: EMBO J. 2023 Apr 11;42(11):e110902. doi: 10.15252/embj.2022110902 (PMC10233377; doi:10.15252/embj.2022110902)

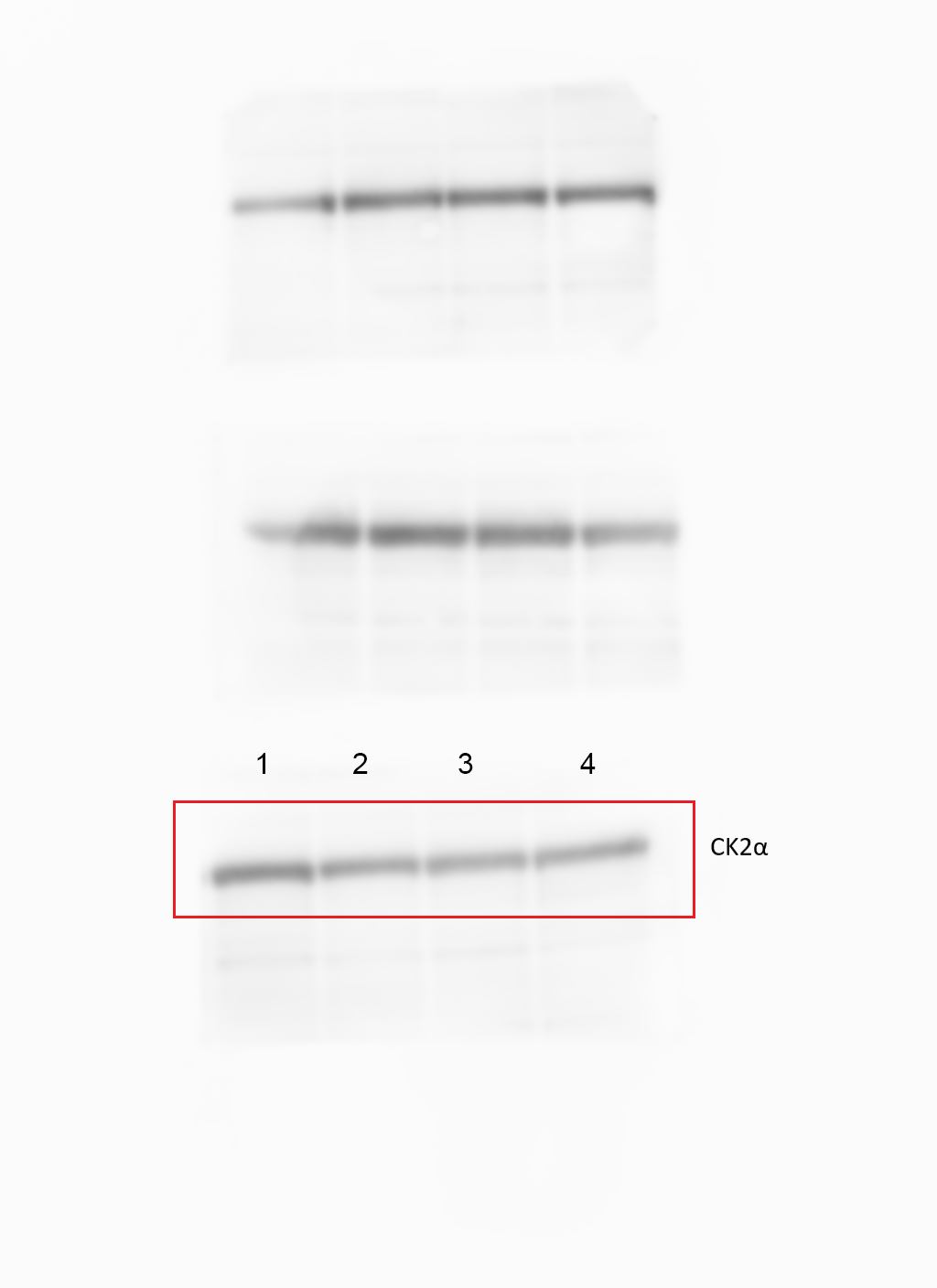

Supplement: Supplementary file 17 — Source Data for Figure 2 [file EMBJ-42-e110902-s002.zip › Figure 2/2E/CK2a_channel1_chemiluminescence.tif]

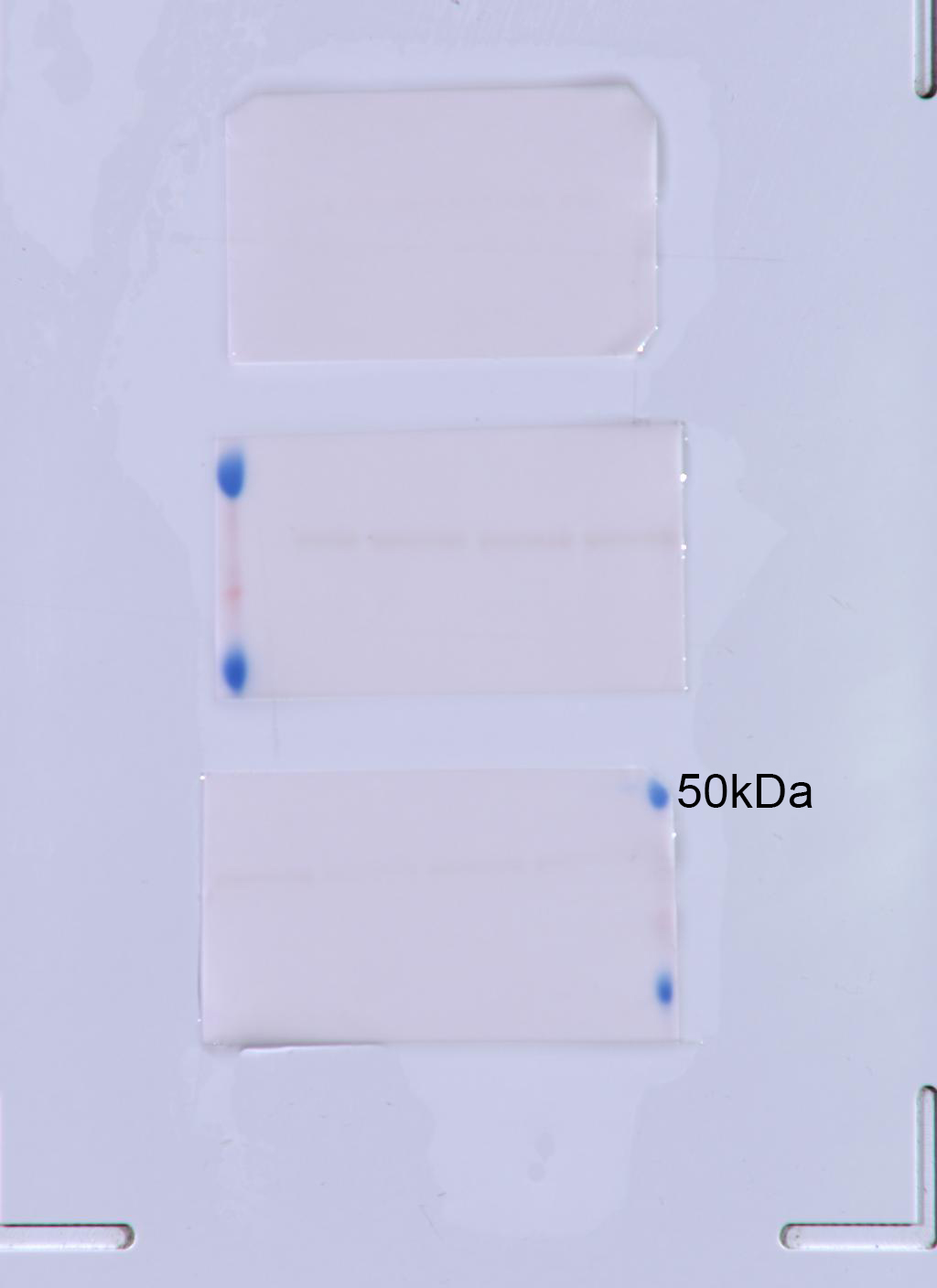

Supplement: Supplementary file 17 — Source Data for Figure 2 [file EMBJ-42-e110902-s002.zip › Figure 2/2E/CK2a_channel2_markers.jpg]

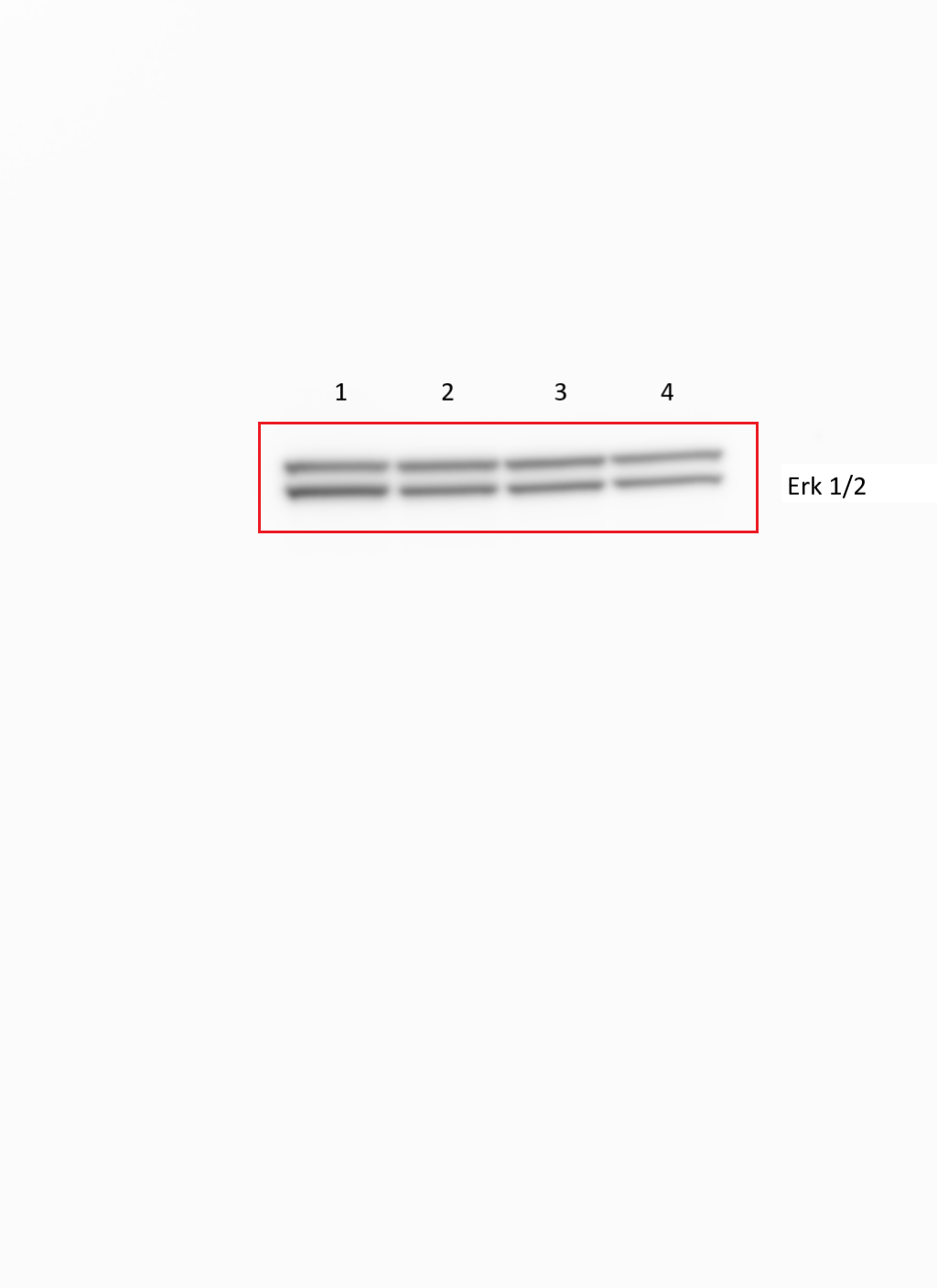

Supplement: Supplementary file 17 — Source Data for Figure 2 [file EMBJ-42-e110902-s002.zip › Figure 2/2E/Erk_channel1_chemiluminescence.tif]

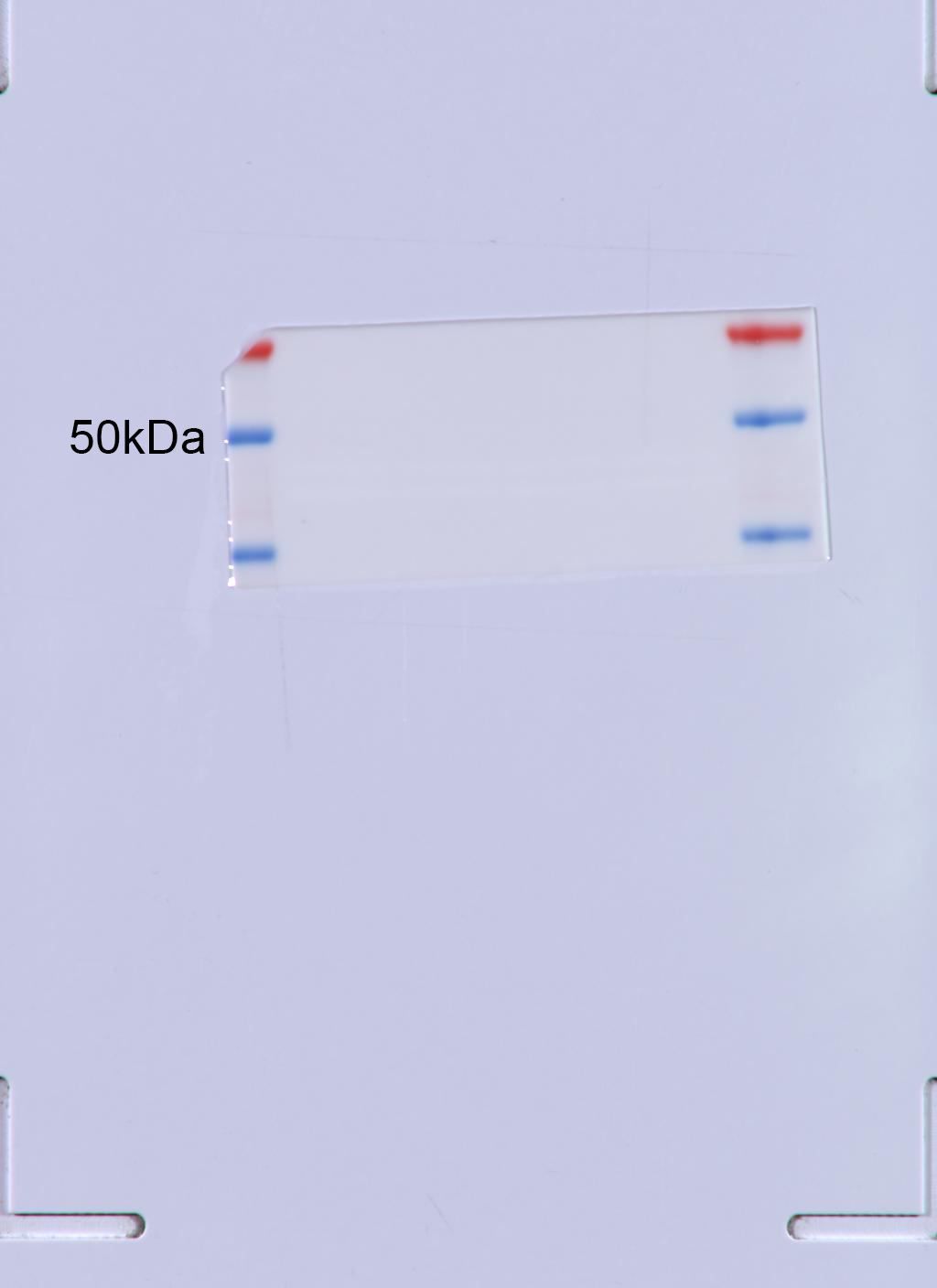

Supplement: Supplementary file 17 — Source Data for Figure 2 [file EMBJ-42-e110902-s002.zip › Figure 2/2E/Erk_channel2_markers.jpg]

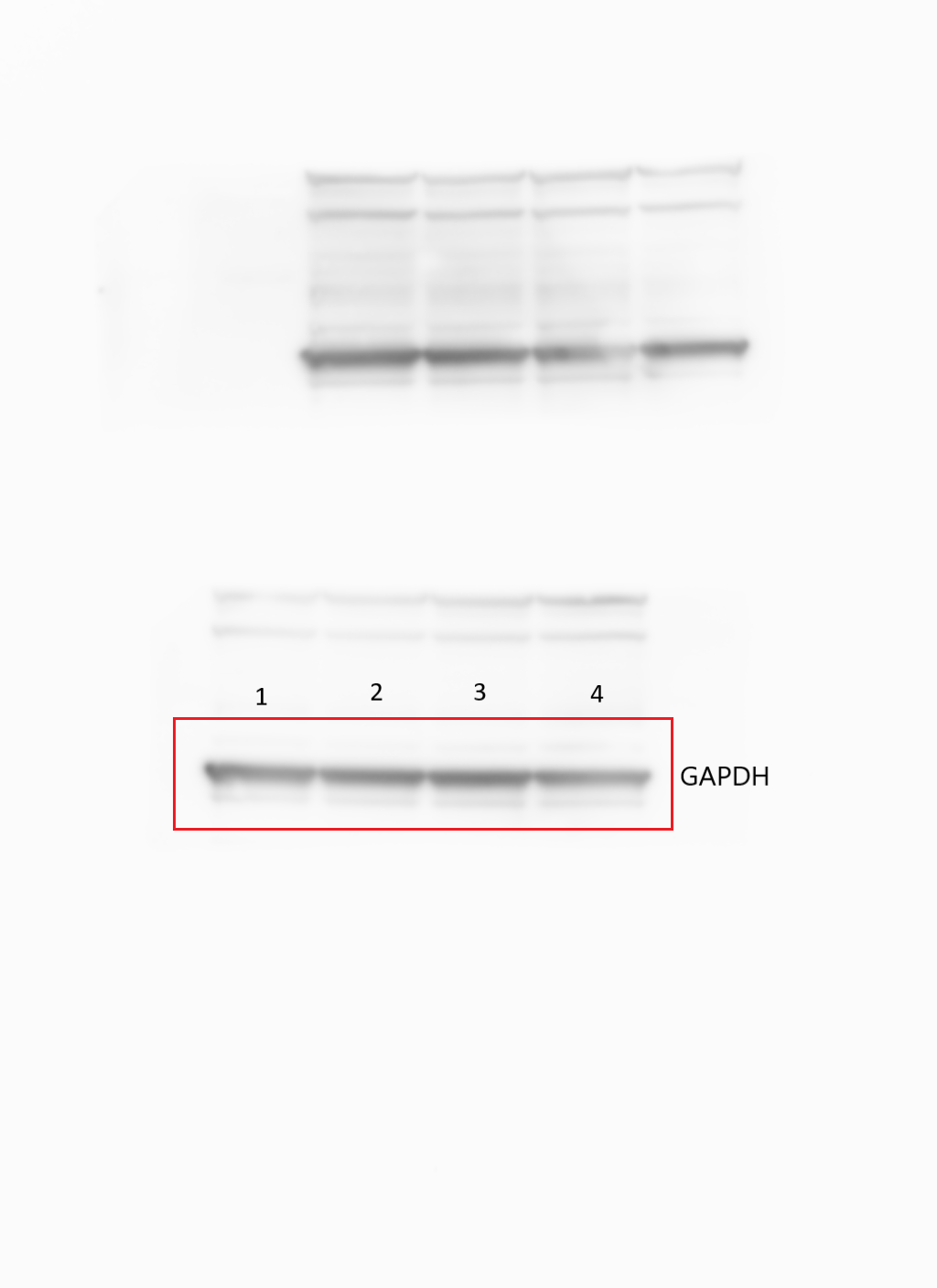

Supplement: Supplementary file 17 — Source Data for Figure 2 [file EMBJ-42-e110902-s002.zip › Figure 2/2E/Gapdh_channel1_chemiluminescence.tif]

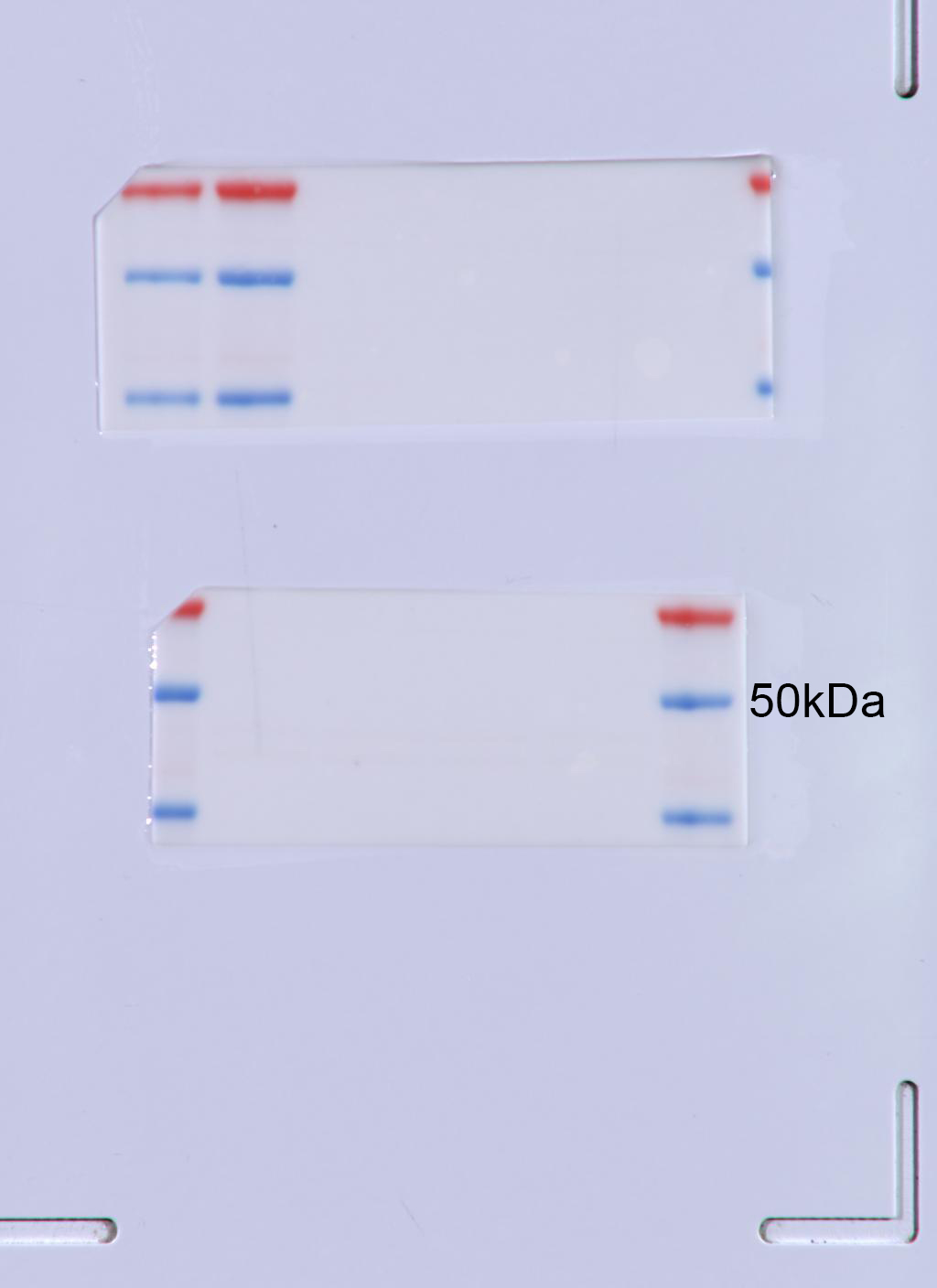

Supplement: Supplementary file 17 — Source Data for Figure 2 [file EMBJ-42-e110902-s002.zip › Figure 2/2E/Gapdh_channel2_markers.jpg]

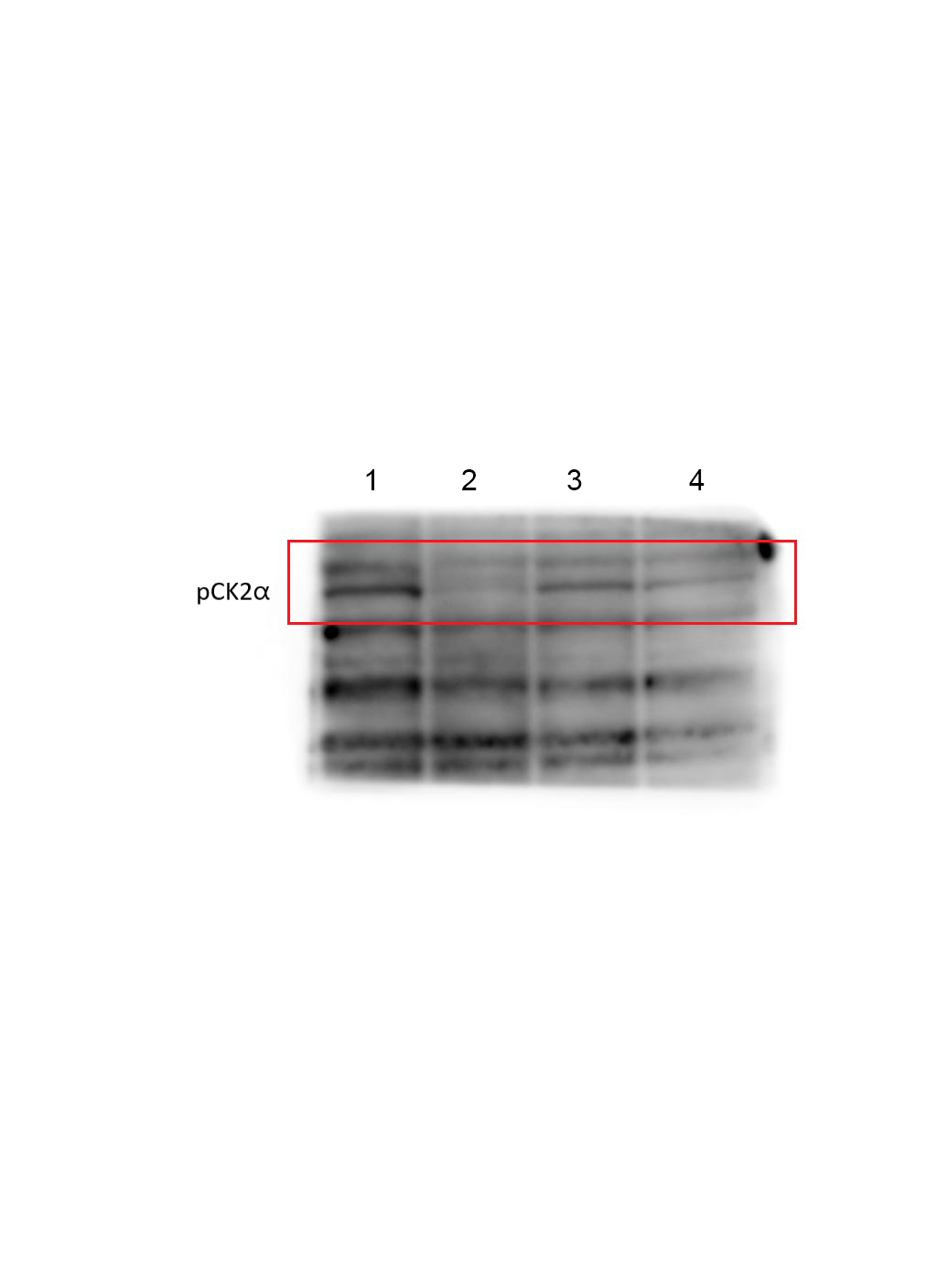

Supplement: Supplementary file 17 — Source Data for Figure 2 [file EMBJ-42-e110902-s002.zip › Figure 2/2E/pCK2_channel1_chemiluminescence.tif]

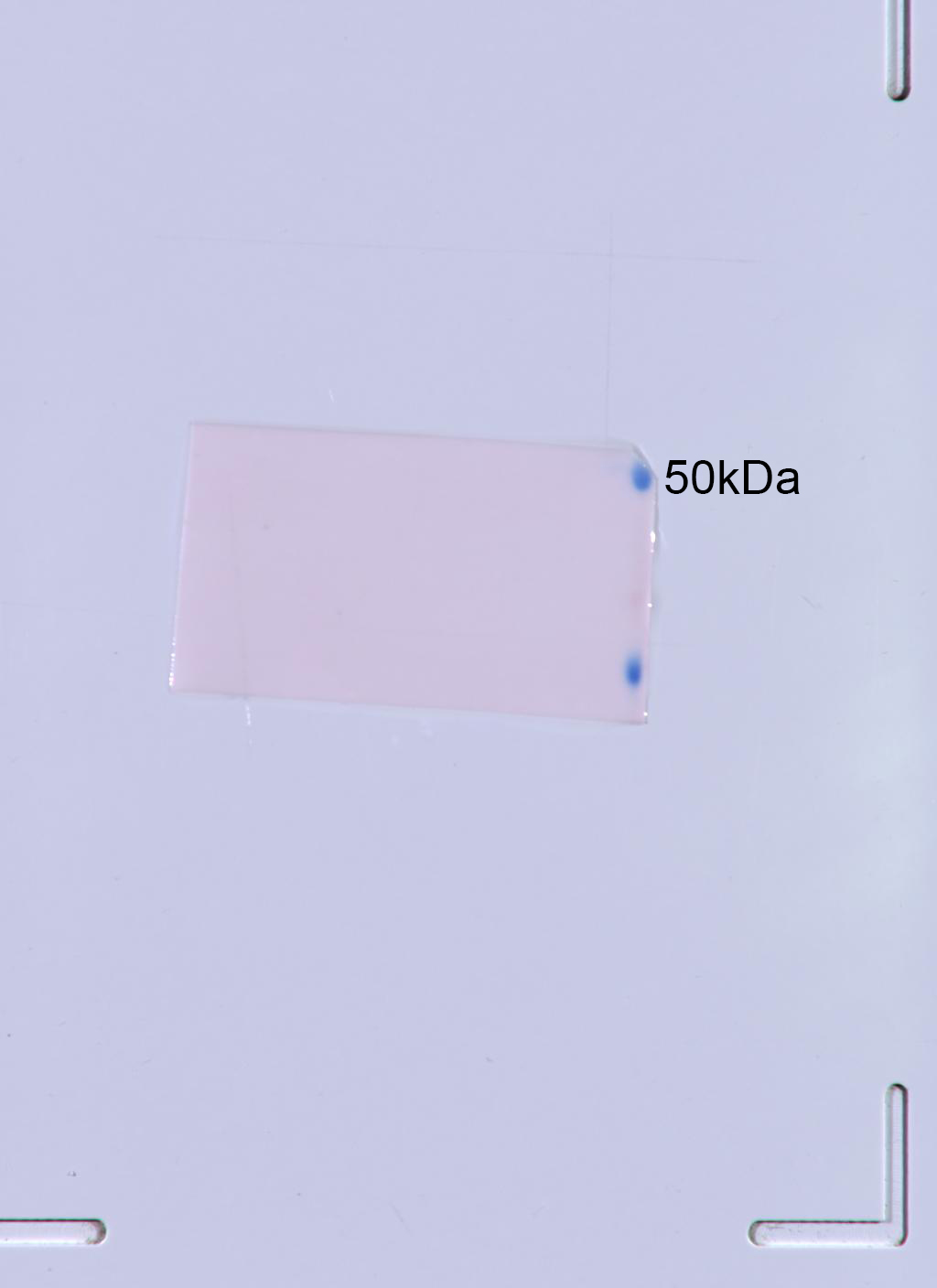

Supplement: Supplementary file 17 — Source Data for Figure 2 [file EMBJ-42-e110902-s002.zip › Figure 2/2E/pCK2_channel2_markers.jpg]

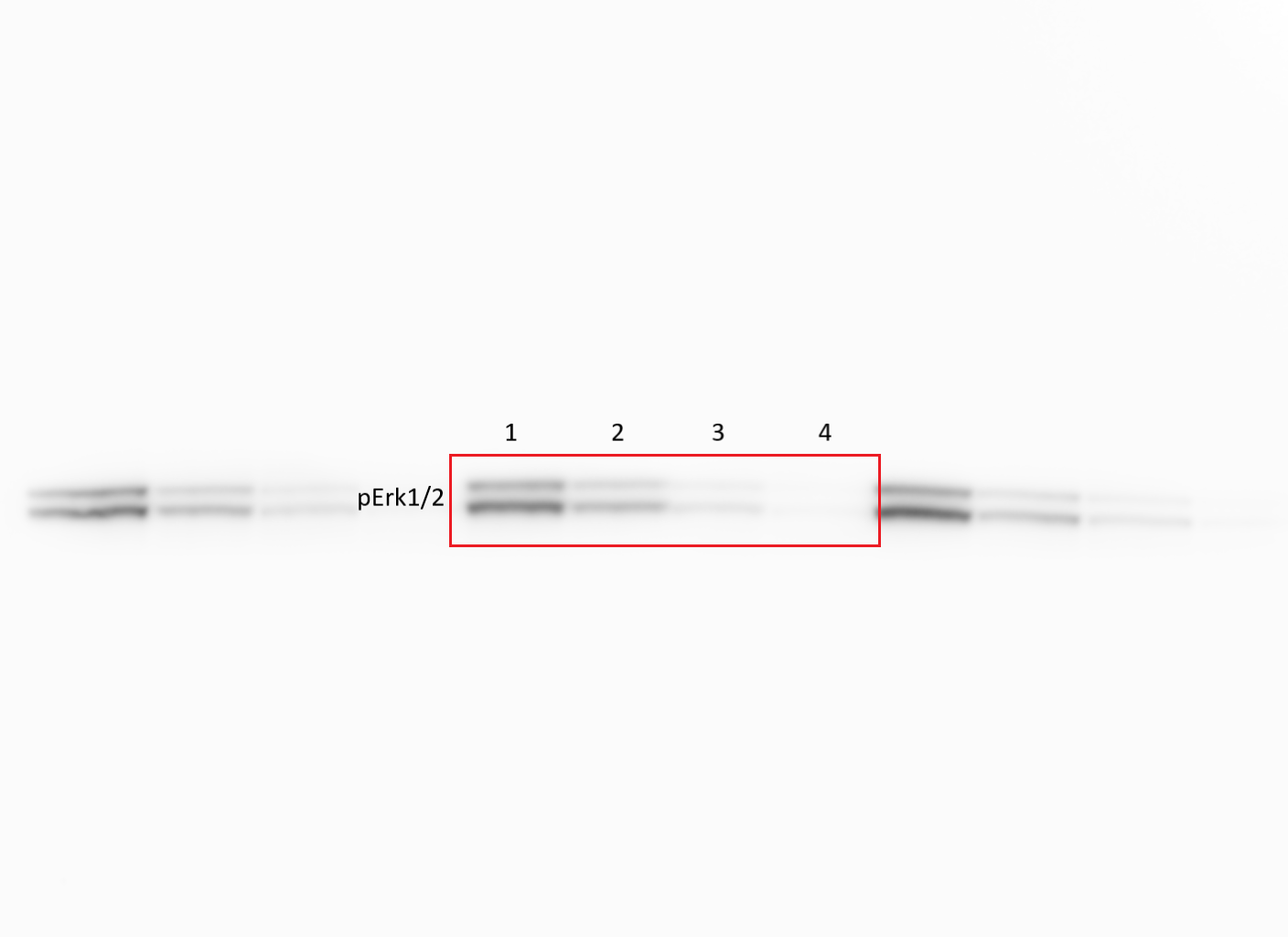

Supplement: Supplementary file 17 — Source Data for Figure 2 [file EMBJ-42-e110902-s002.zip › Figure 2/2E/pErk_channel1_chemiluminescence.tif]

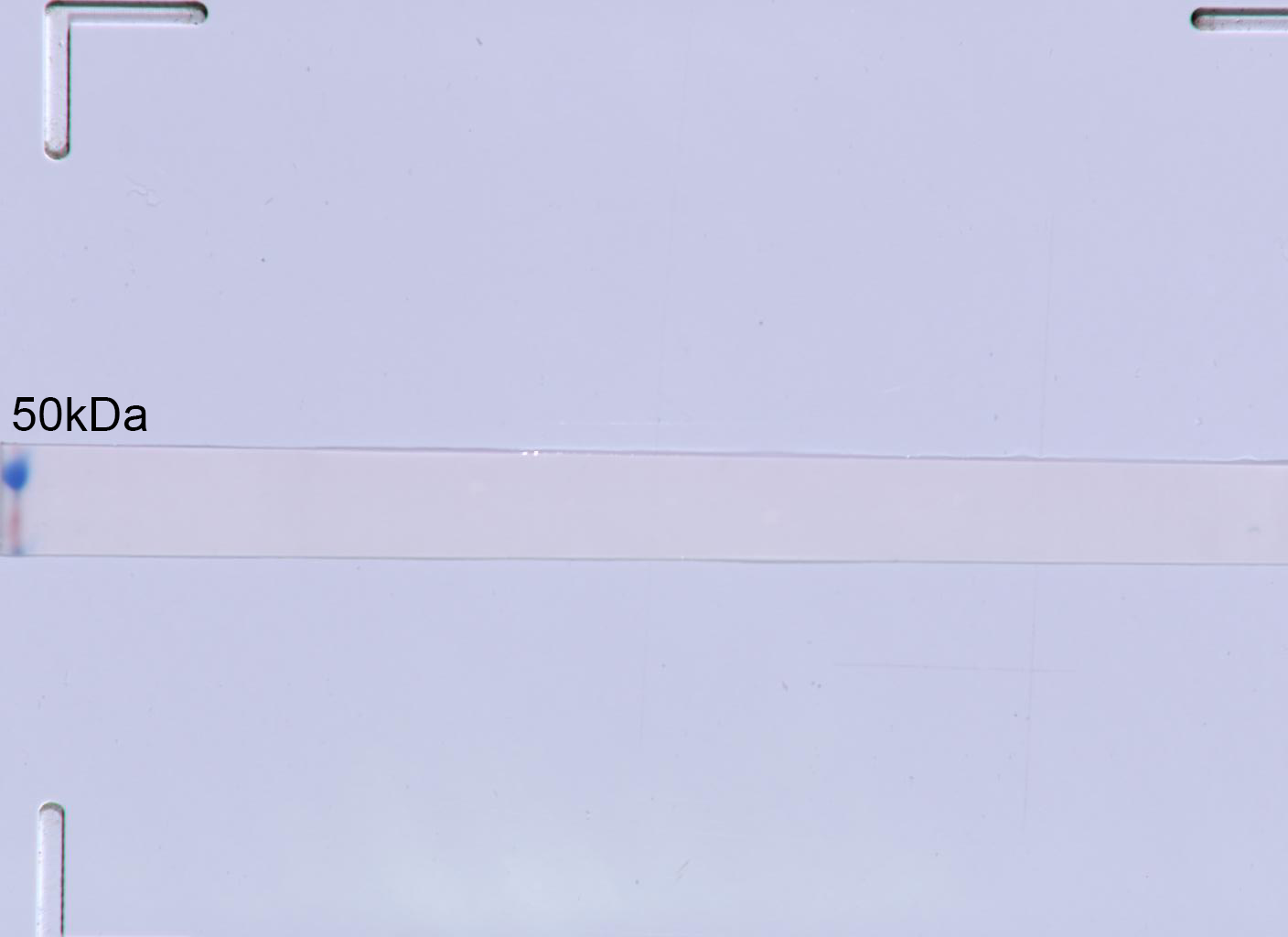

Supplement: Supplementary file 17 — Source Data for Figure 2 [file EMBJ-42-e110902-s002.zip › Figure 2/2E/pErk_channel2_markers.jpg]

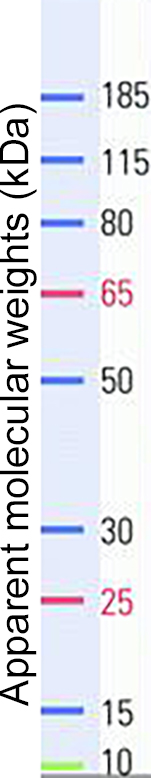

Supplement: Supplementary file 17 — Source Data for Figure 2 [file EMBJ-42-e110902-s002.zip › Figure 2/2E/protein ladder size guide.jpg]

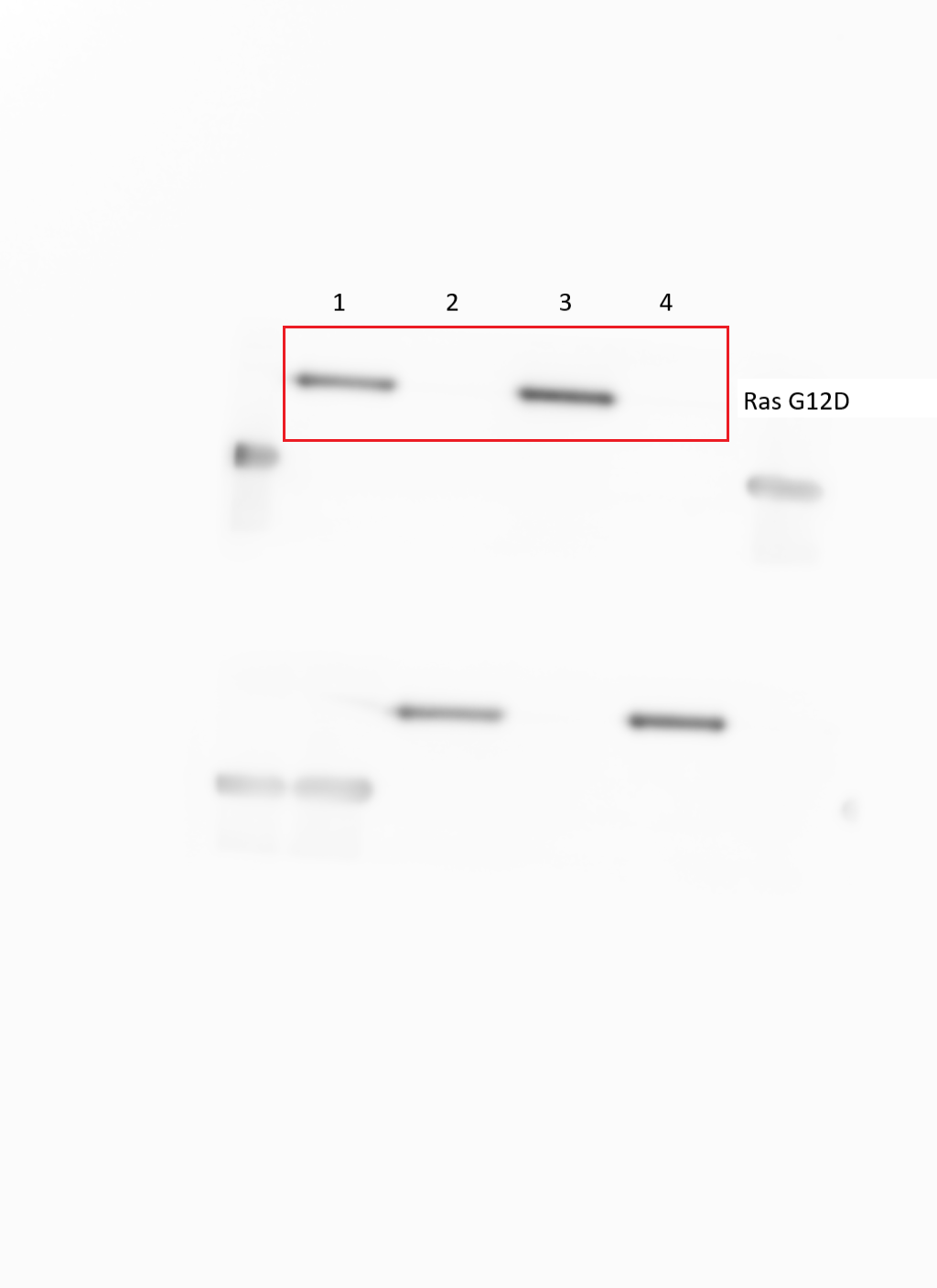

Supplement: Supplementary file 17 — Source Data for Figure 2 [file EMBJ-42-e110902-s002.zip › Figure 2/2E/RasG12D_channel1_chemiluminescence.tif]

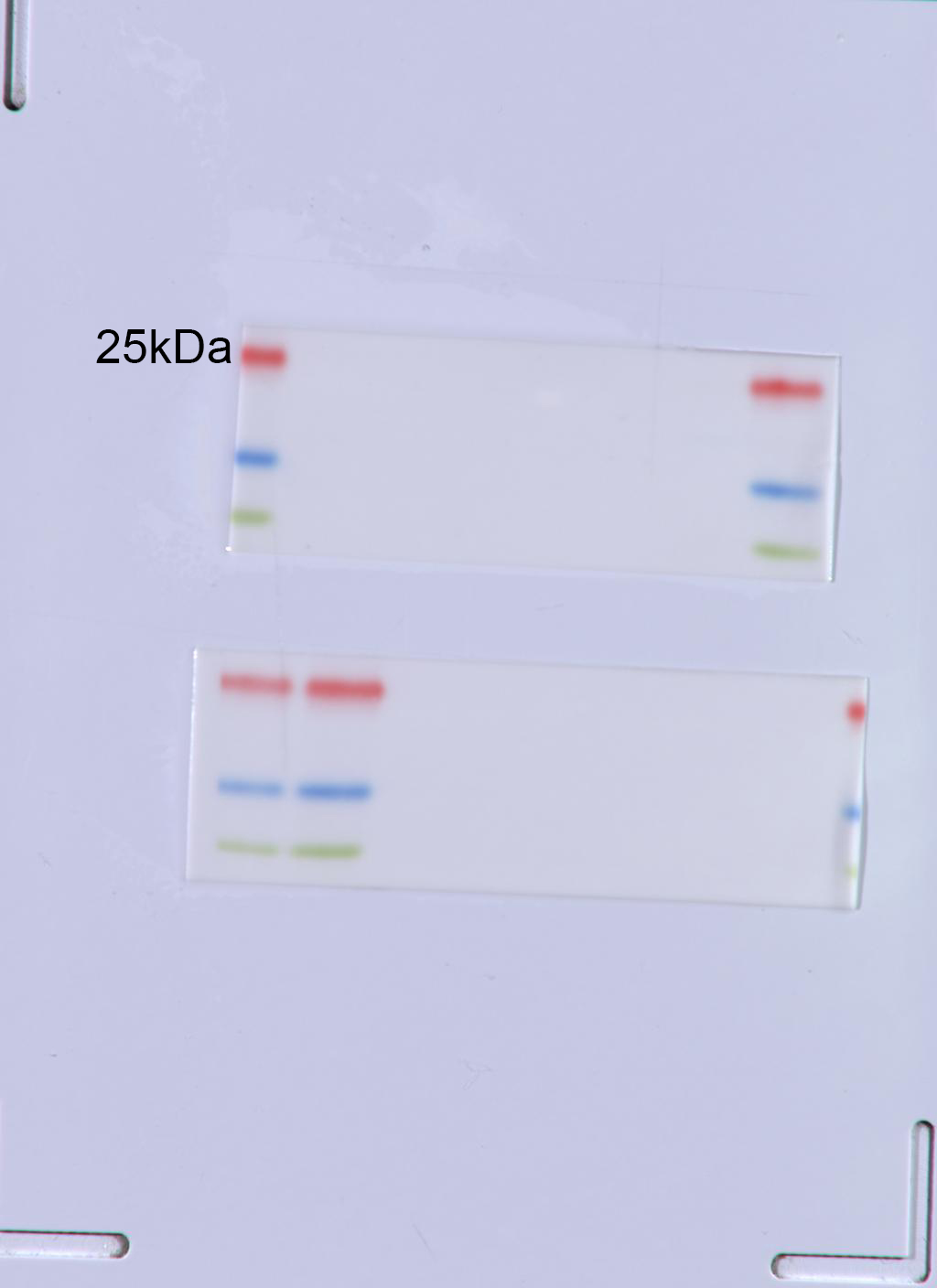

Supplement: Supplementary file 17 — Source Data for Figure 2 [file EMBJ-42-e110902-s002.zip › Figure 2/2E/RasG12D_channel2_markers.jpg]

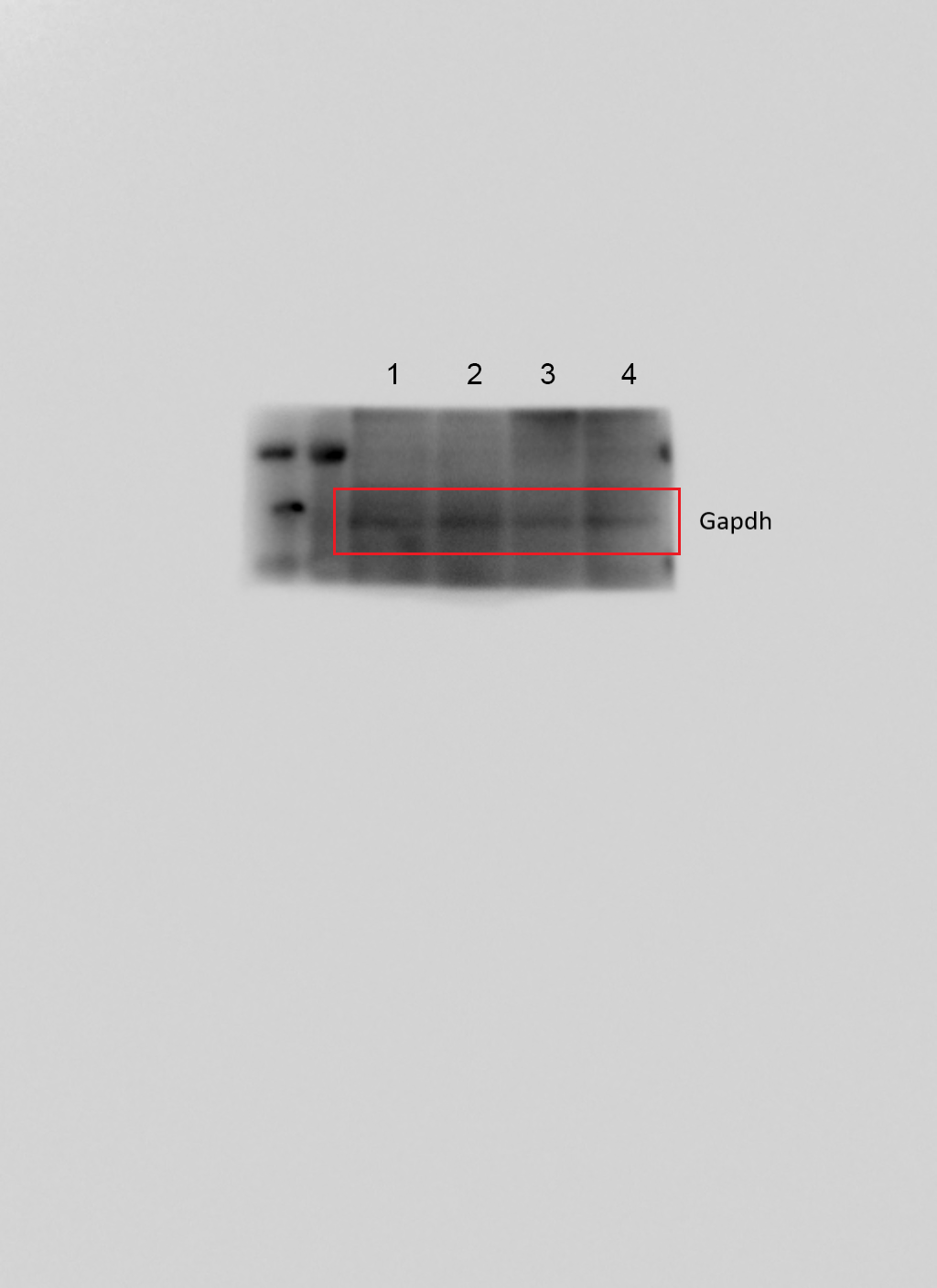

Supplement: Supplementary file 17 — Source Data for Figure 2 [file EMBJ-42-e110902-s002.zip › Figure 2/2G/Interface_Gapdh_channel1_chemiluminescence.tif]

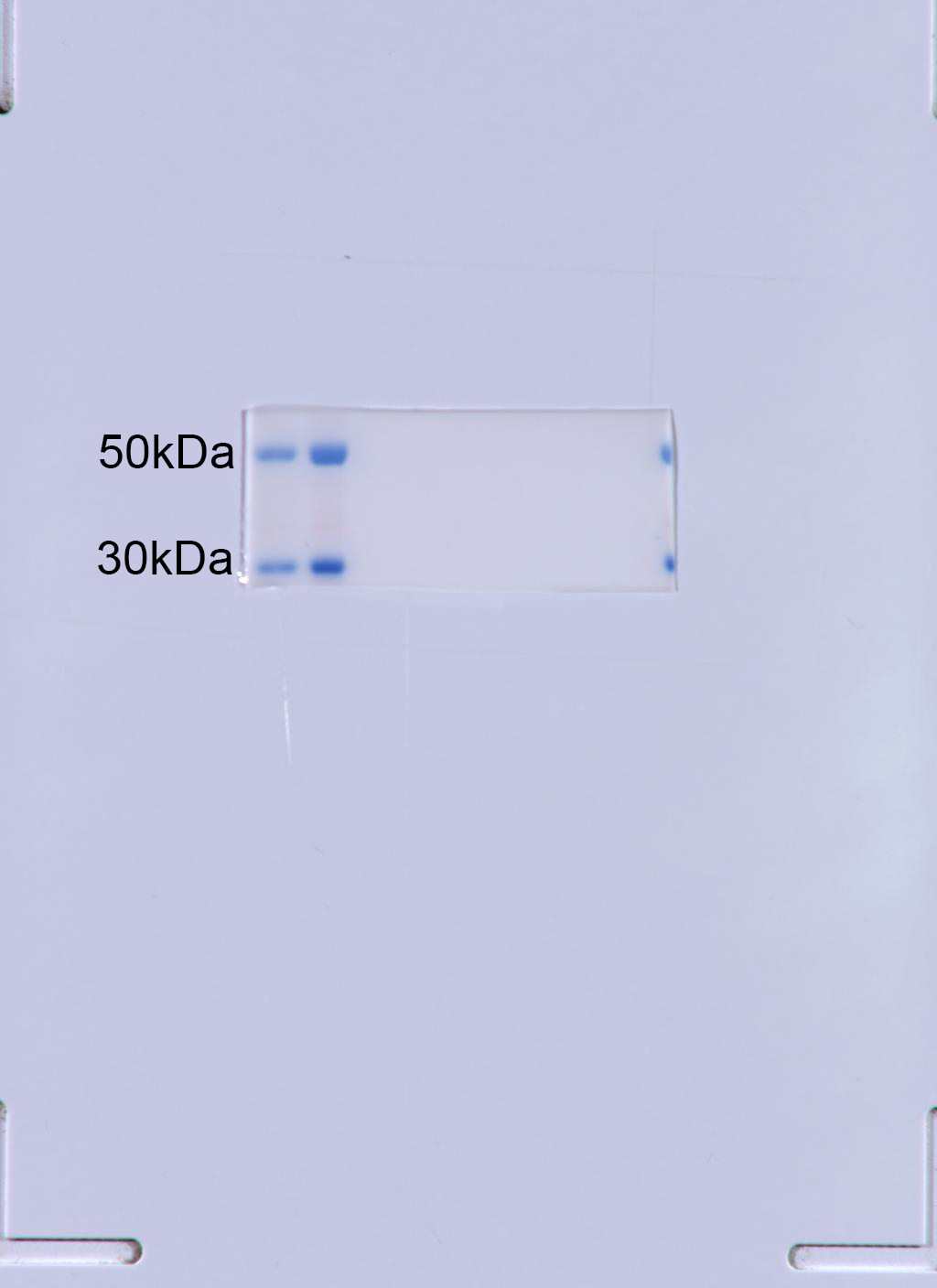

Supplement: Supplementary file 17 — Source Data for Figure 2 [file EMBJ-42-e110902-s002.zip › Figure 2/2G/Interface_Gapdh_channel2_markers.jpg]

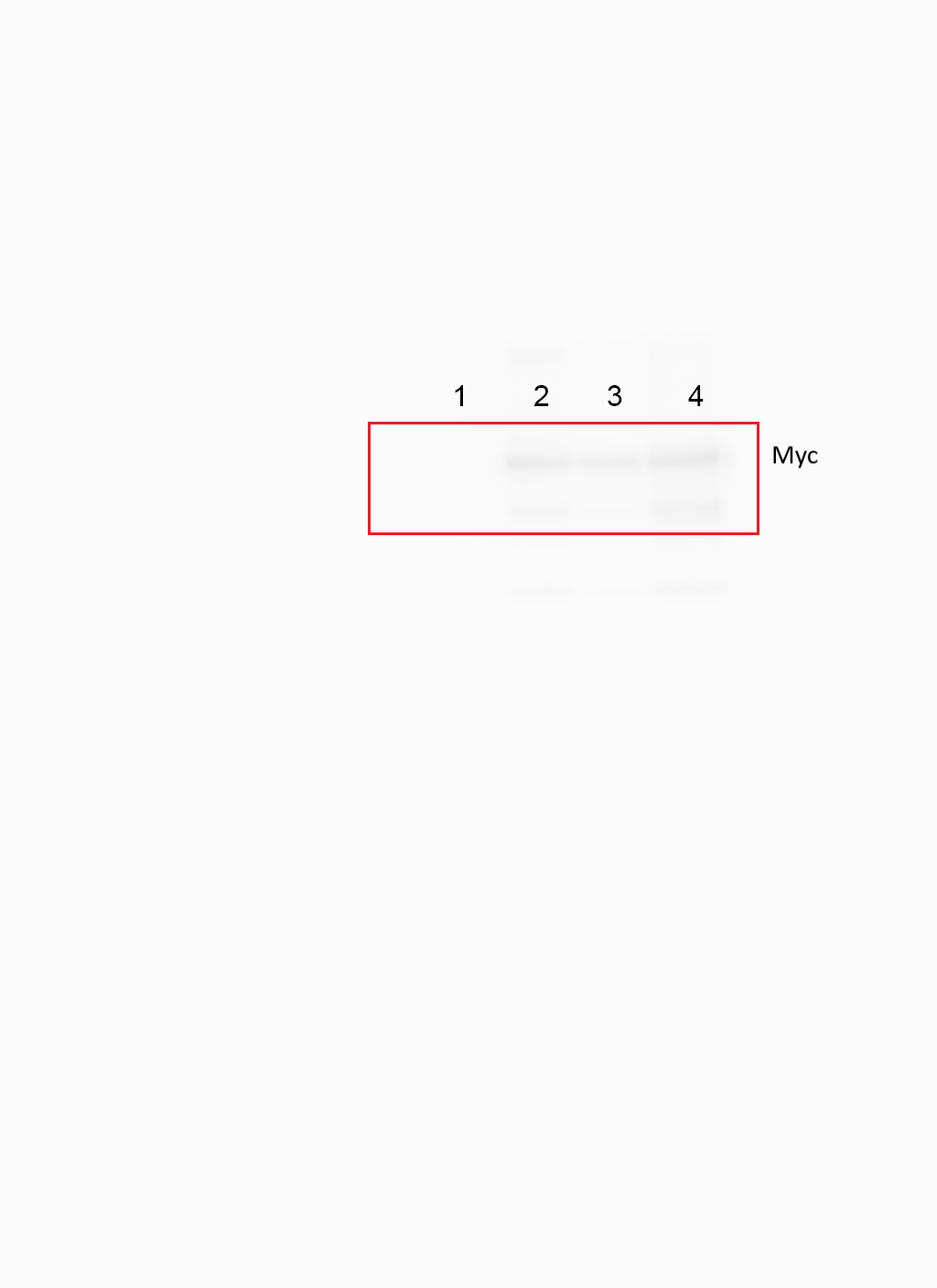

Supplement: Supplementary file 17 — Source Data for Figure 2 [file EMBJ-42-e110902-s002.zip › Figure 2/2G/Interface_Myc_channel1_chemiluminescence.tif]

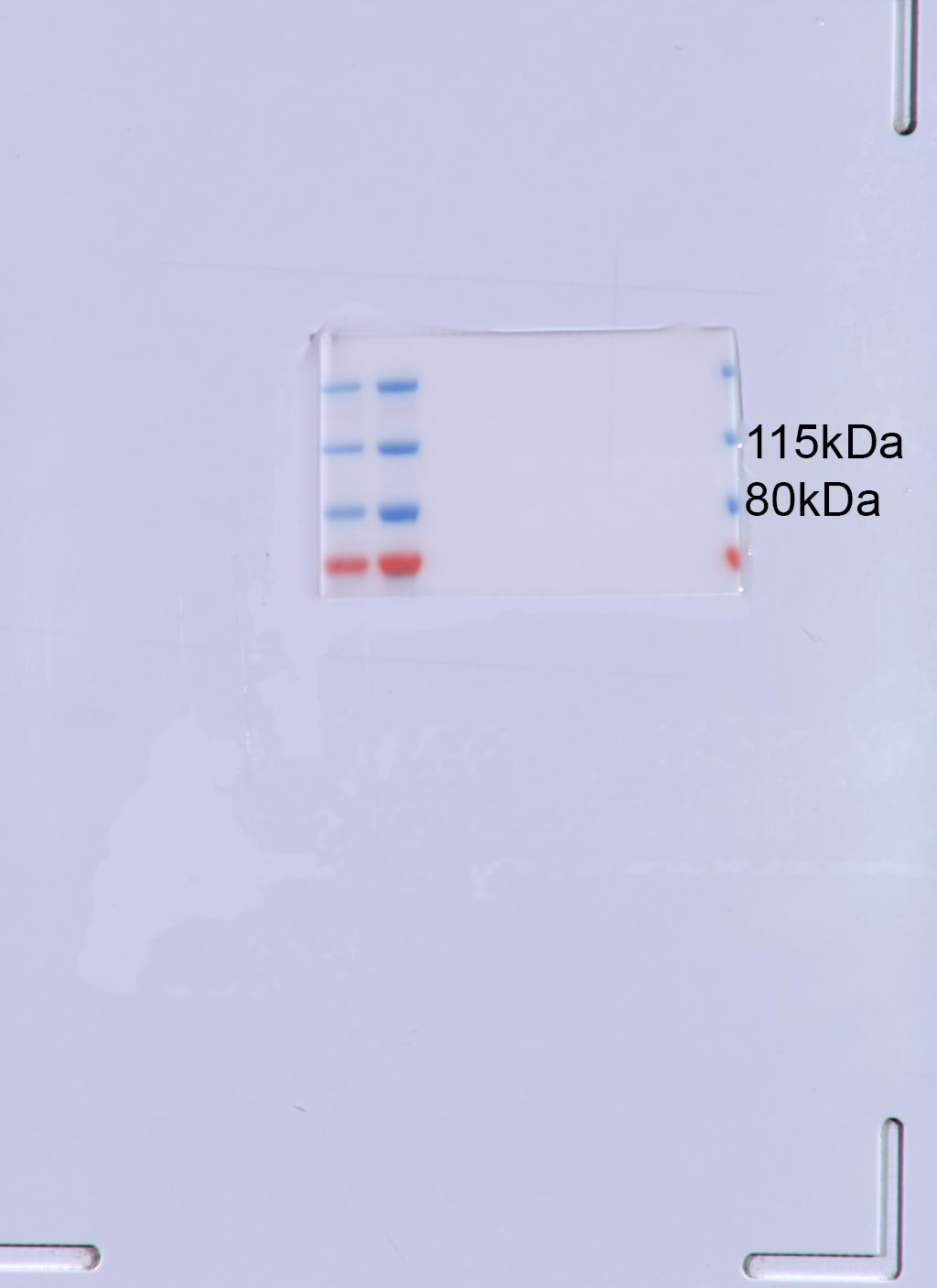

Supplement: Supplementary file 17 — Source Data for Figure 2 [file EMBJ-42-e110902-s002.zip › Figure 2/2G/Interface_Myc_channel2_markers.jpg]

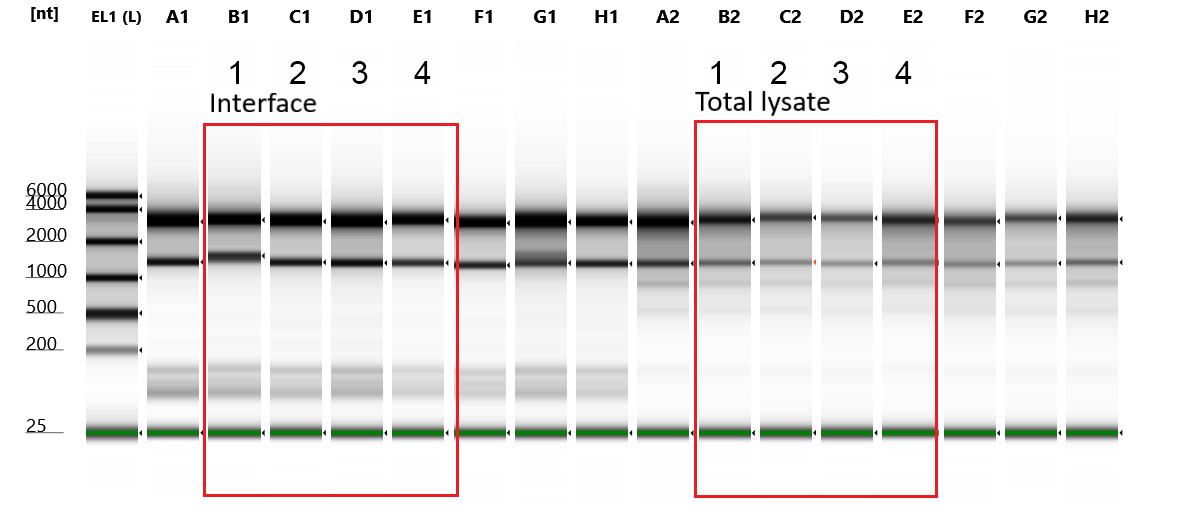

Supplement: Supplementary file 17 — Source Data for Figure 2 [file EMBJ-42-e110902-s002.zip › Figure 2/2G/RNA Tape - Interface and total lysate.tif]

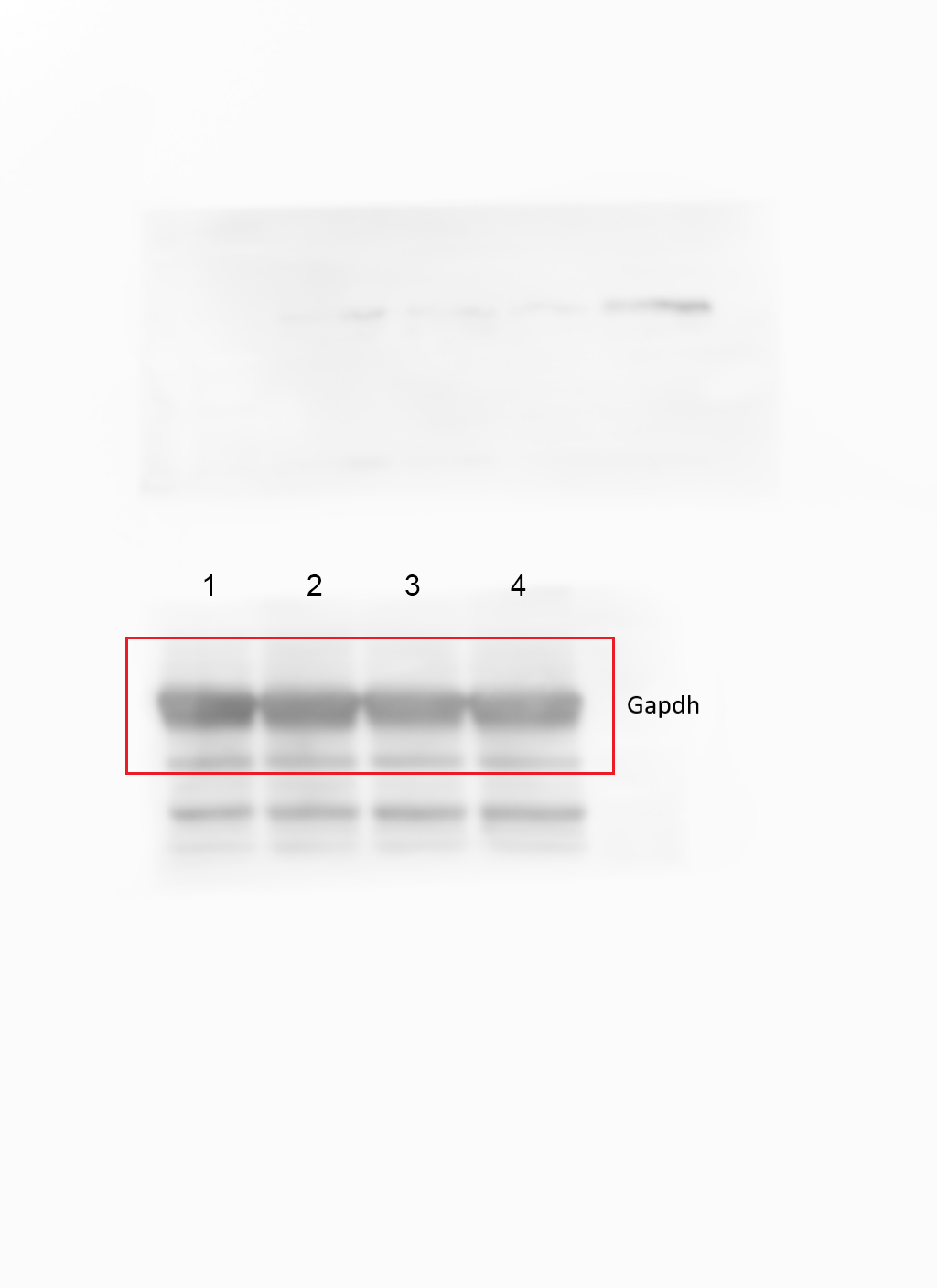

Supplement: Supplementary file 17 — Source Data for Figure 2 [file EMBJ-42-e110902-s002.zip › Figure 2/2G/Total Lysate_Gapdh_channel1_chemiluminescence.tif]

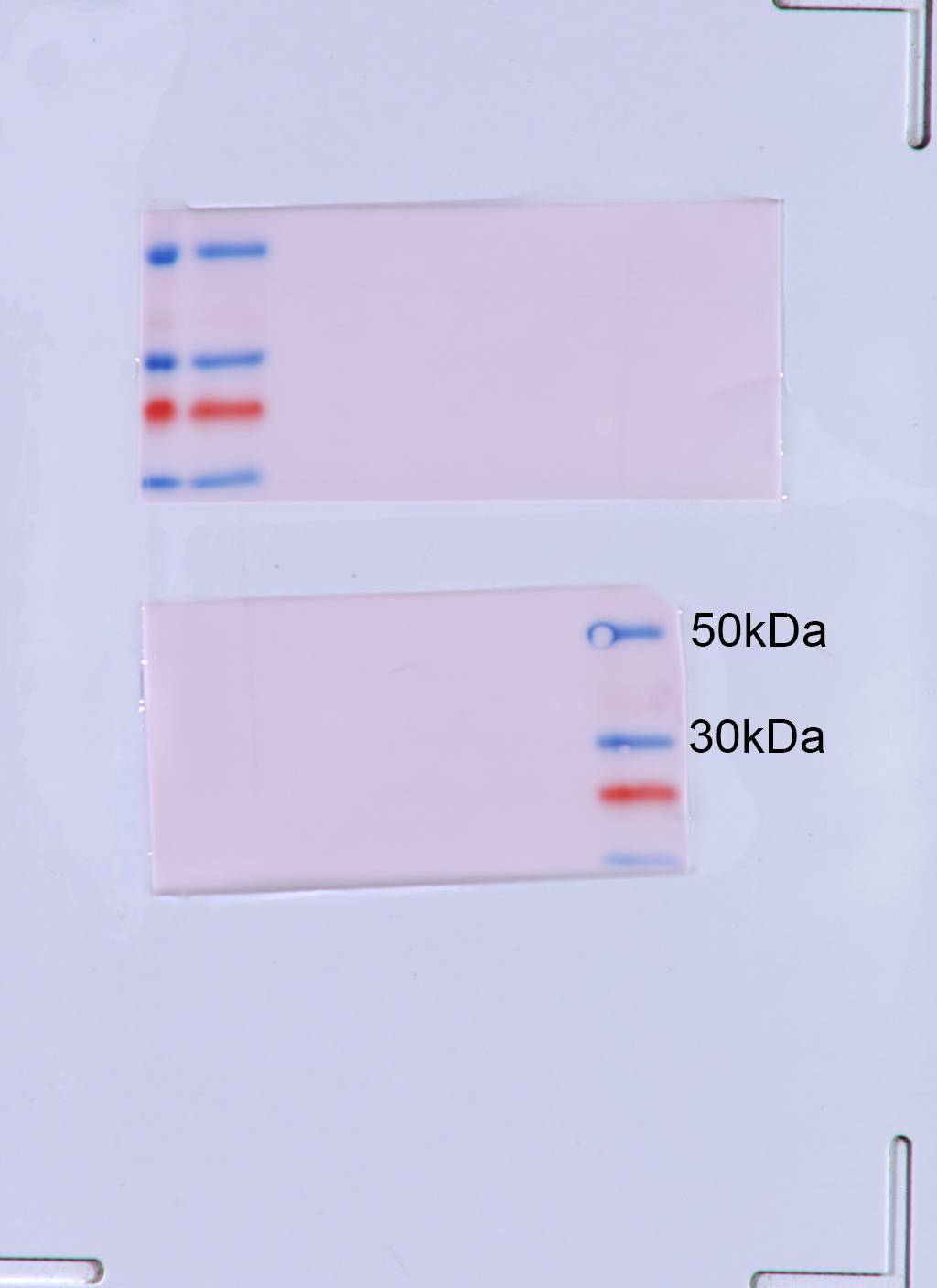

Supplement: Supplementary file 17 — Source Data for Figure 2 [file EMBJ-42-e110902-s002.zip › Figure 2/2G/Total Lysate_Gapdh_channel2_markers.jpg]

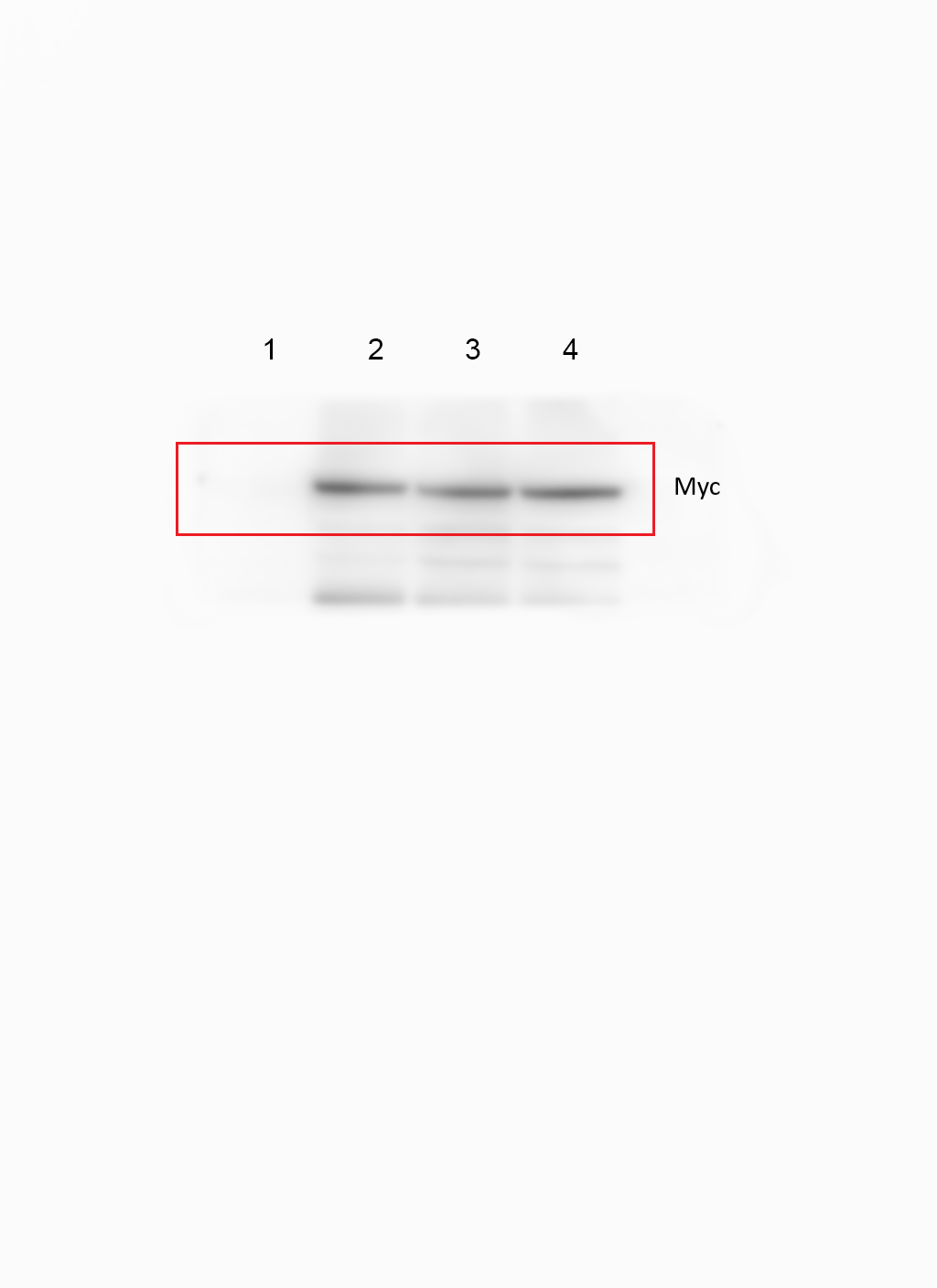

Supplement: Supplementary file 17 — Source Data for Figure 2 [file EMBJ-42-e110902-s002.zip › Figure 2/2G/Total Lysate_Myc_channel1_chemiluminescence.tif]

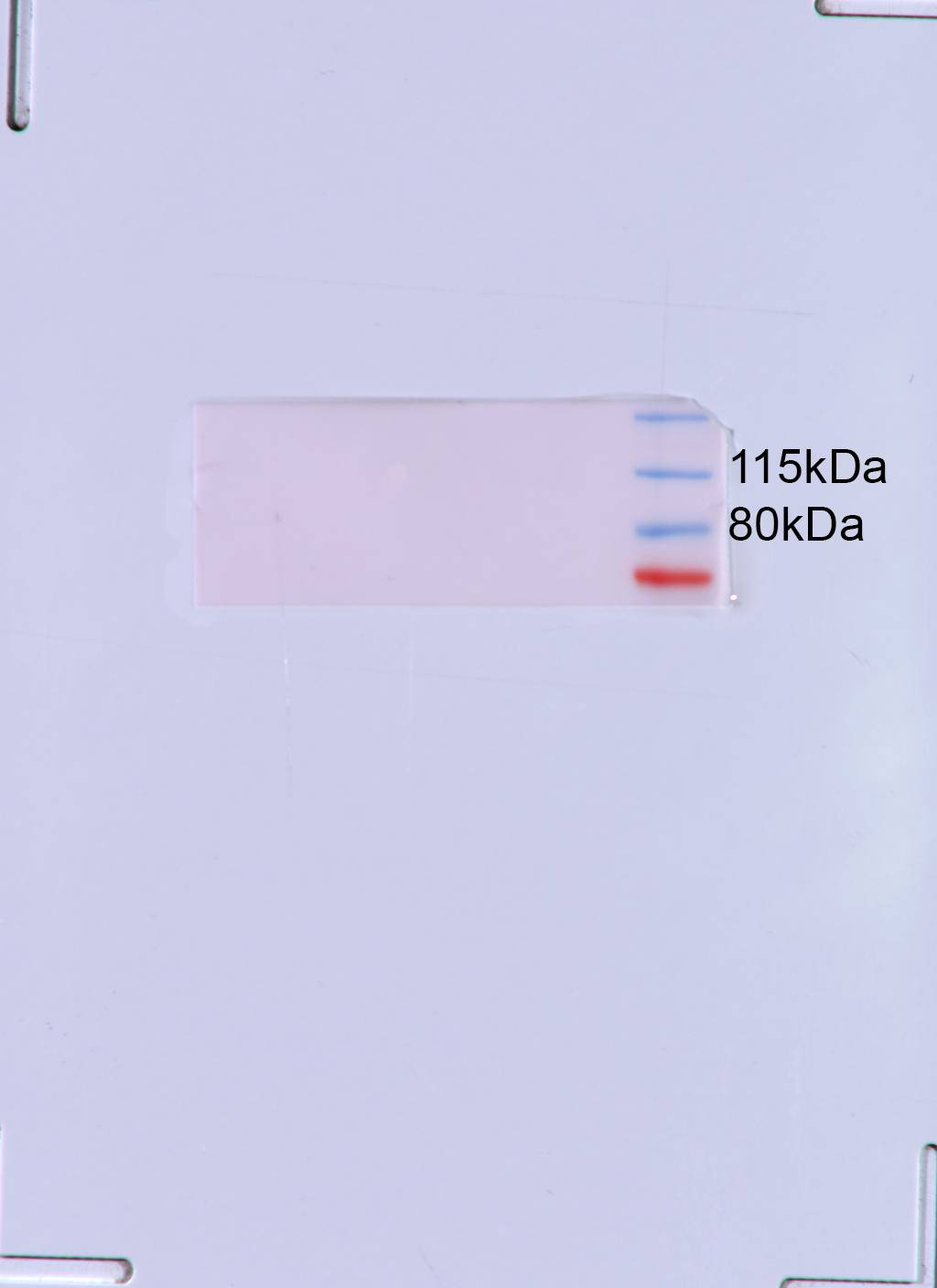

Supplement: Supplementary file 17 — Source Data for Figure 2 [file EMBJ-42-e110902-s002.zip › Figure 2/2G/Total Lysate_Myc_channel2_markers.jpg]

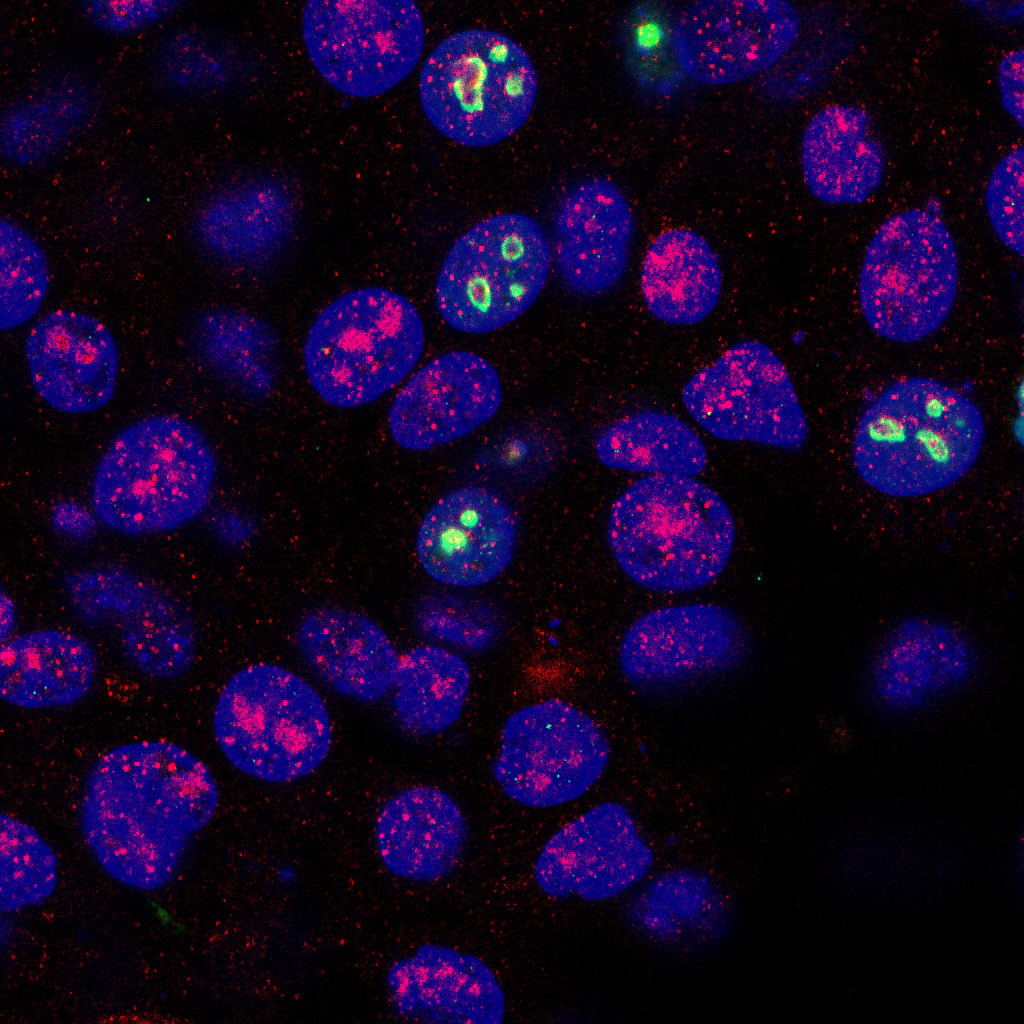

Supplement: Supplementary file 18 — Source Data for Figure 3 [file EMBJ-42-e110902-s005.zip › Figure 3/3B/Myc-Ncl S4A.tif]

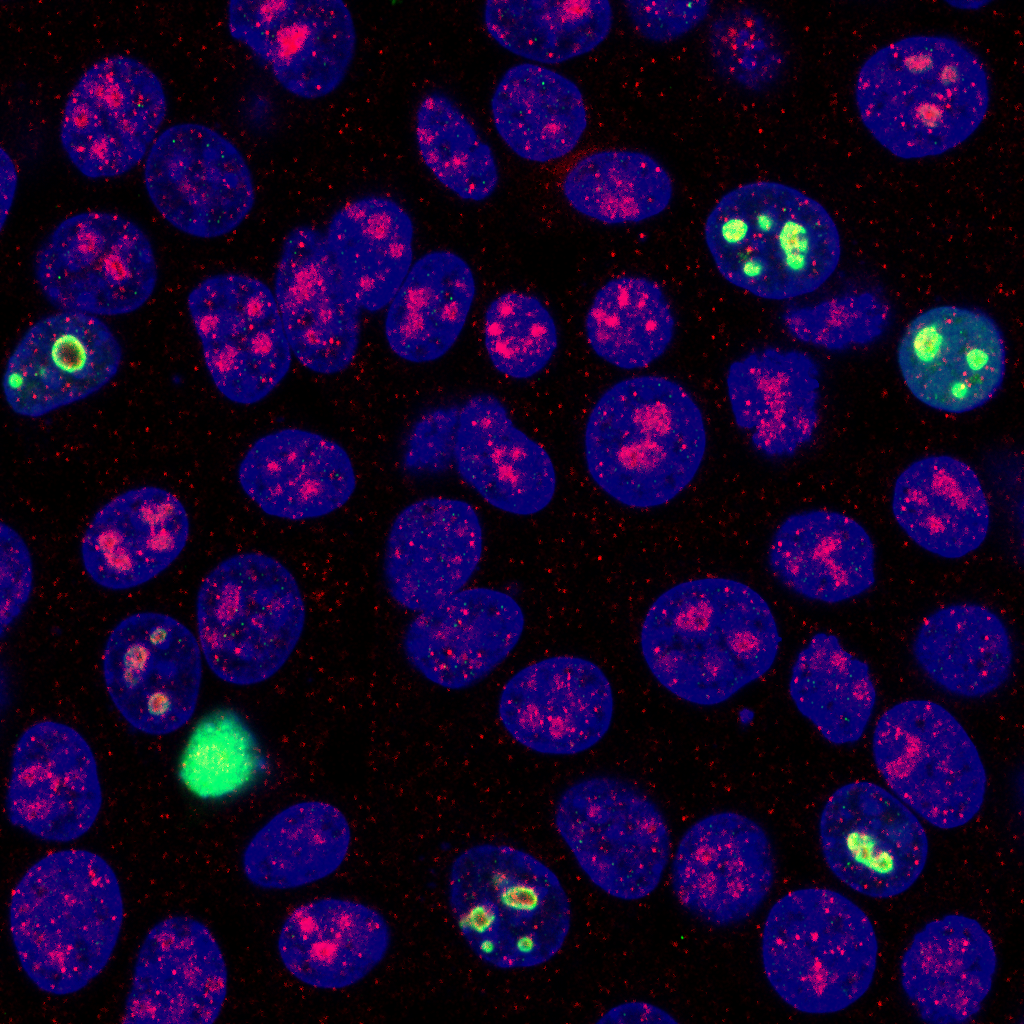

Supplement: Supplementary file 18 — Source Data for Figure 3 [file EMBJ-42-e110902-s005.zip › Figure 3/3B/Myc-Ncl S4D.tif]

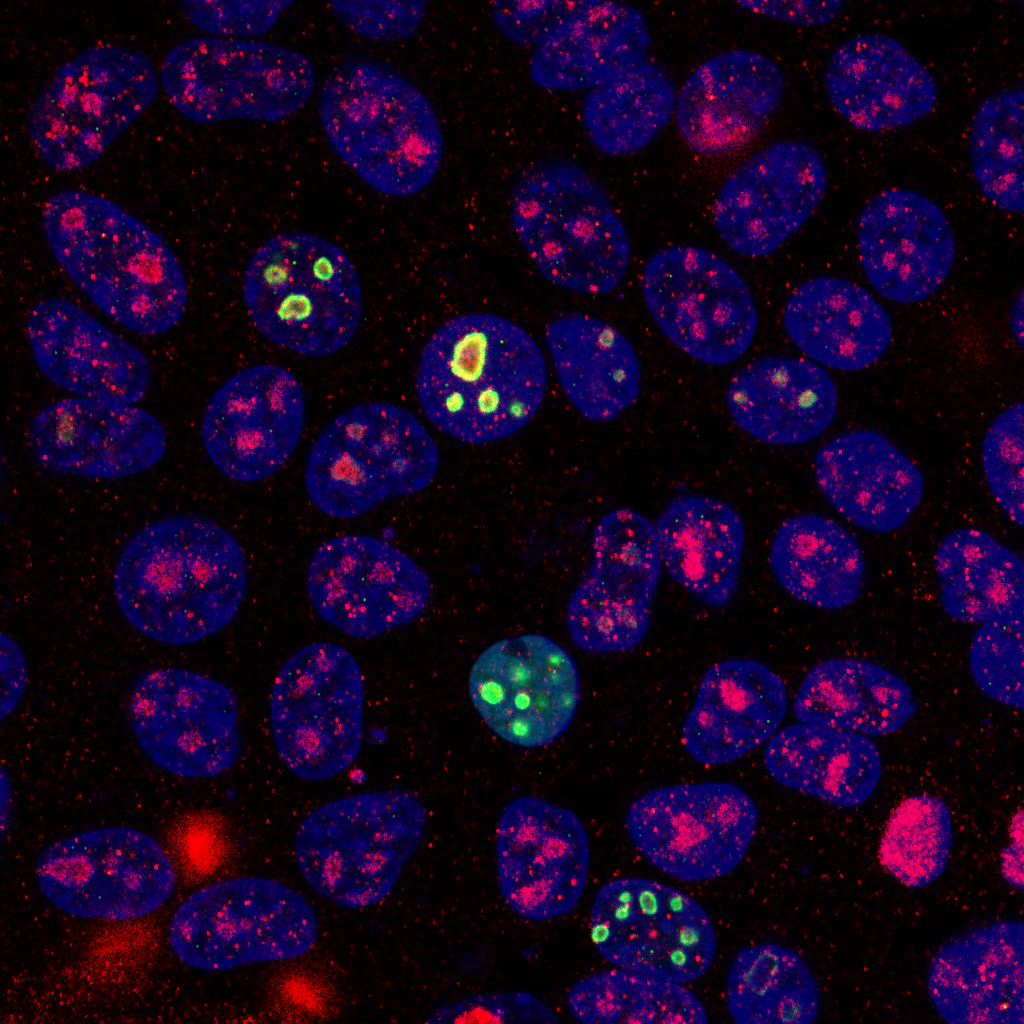

Supplement: Supplementary file 18 — Source Data for Figure 3 [file EMBJ-42-e110902-s005.zip › Figure 3/3B/Myc-Ncl WT.tif]

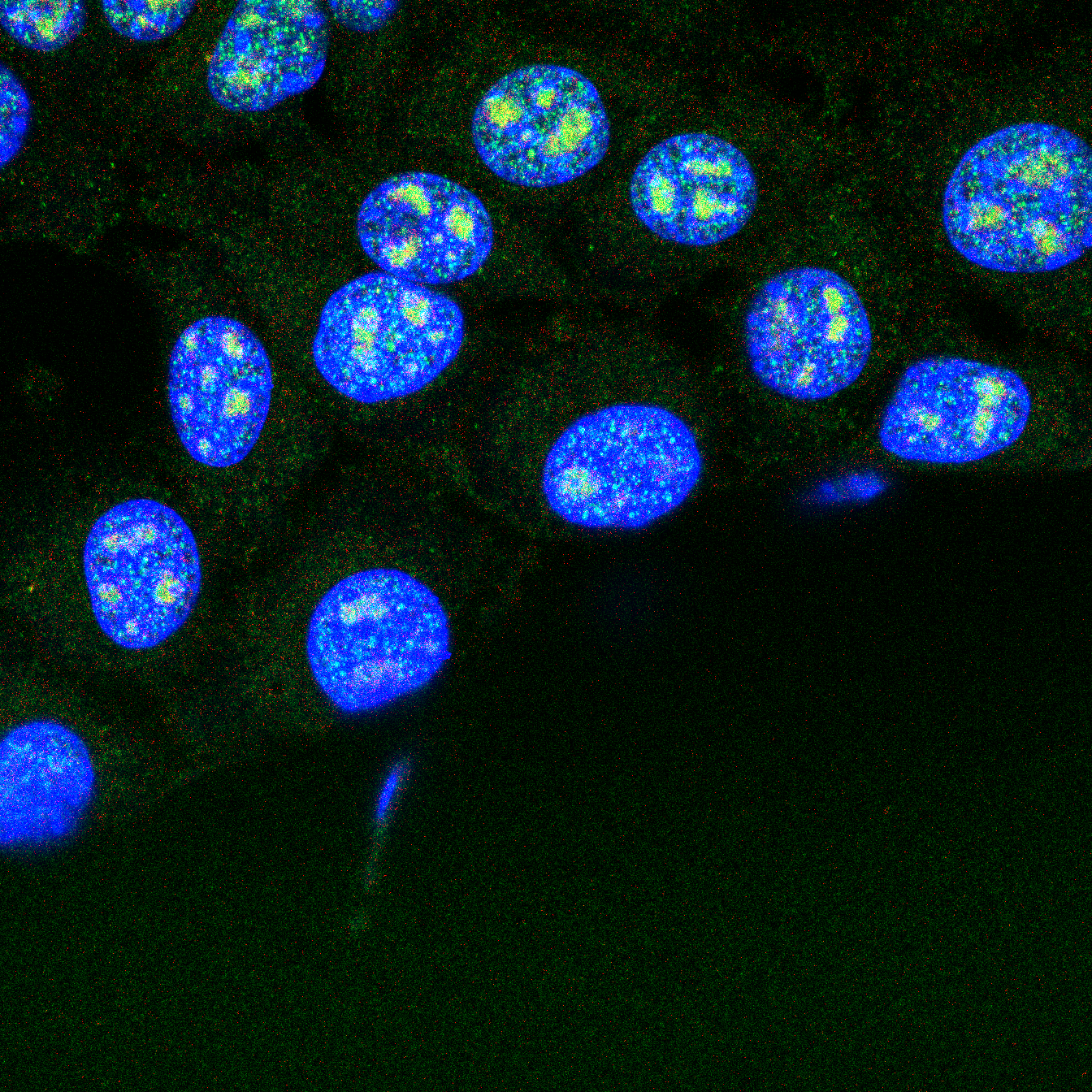

Supplement: Supplementary file 19 — Source Data for Figure 4 [file EMBJ-42-e110902-s010.zip › Figure 4/4A/KRASinactivation_Control siRNA_DOX.tif]

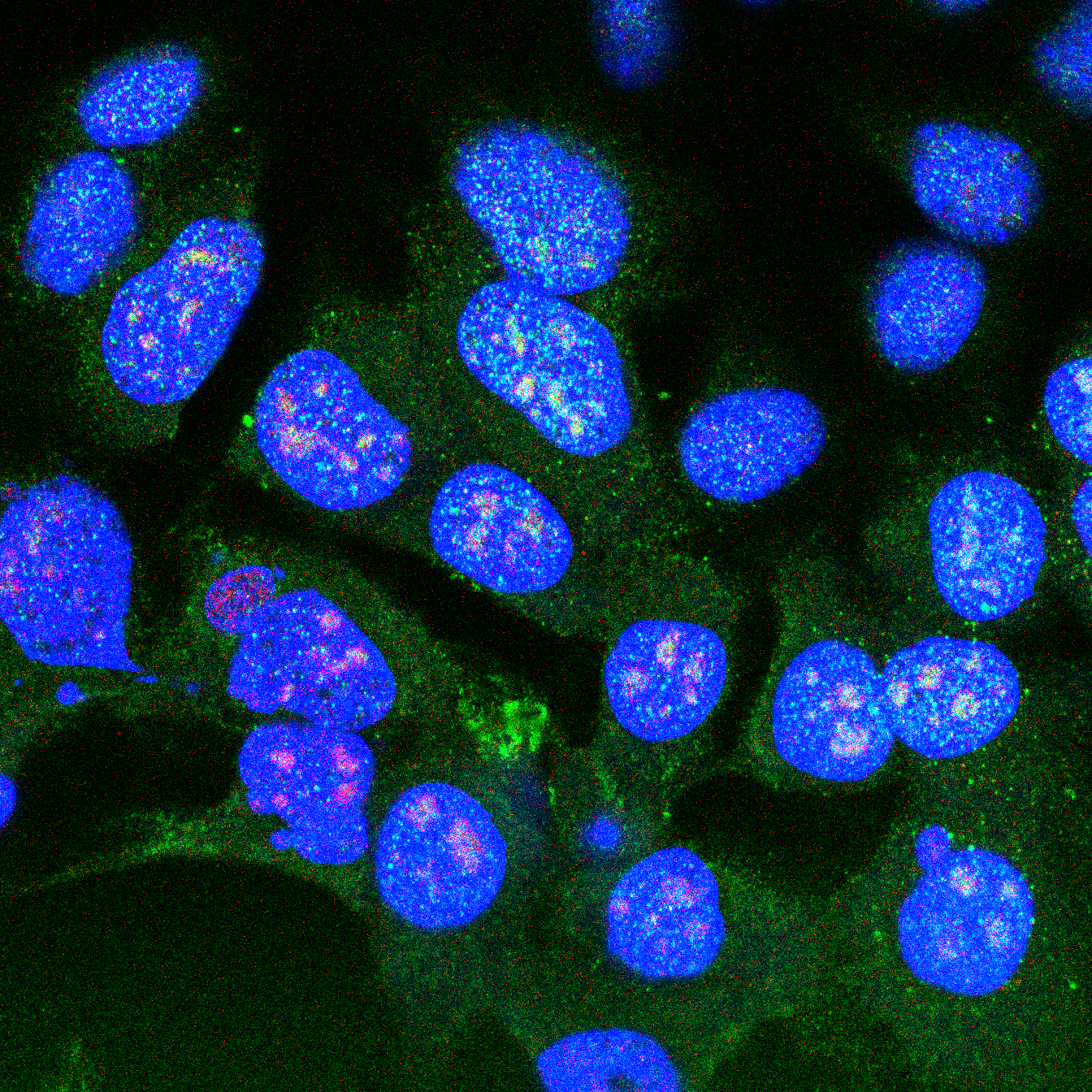

Supplement: Supplementary file 19 — Source Data for Figure 4 [file EMBJ-42-e110902-s010.zip › Figure 4/4A/KRASinactivation_Control siRNA_NO-DOX.tif]

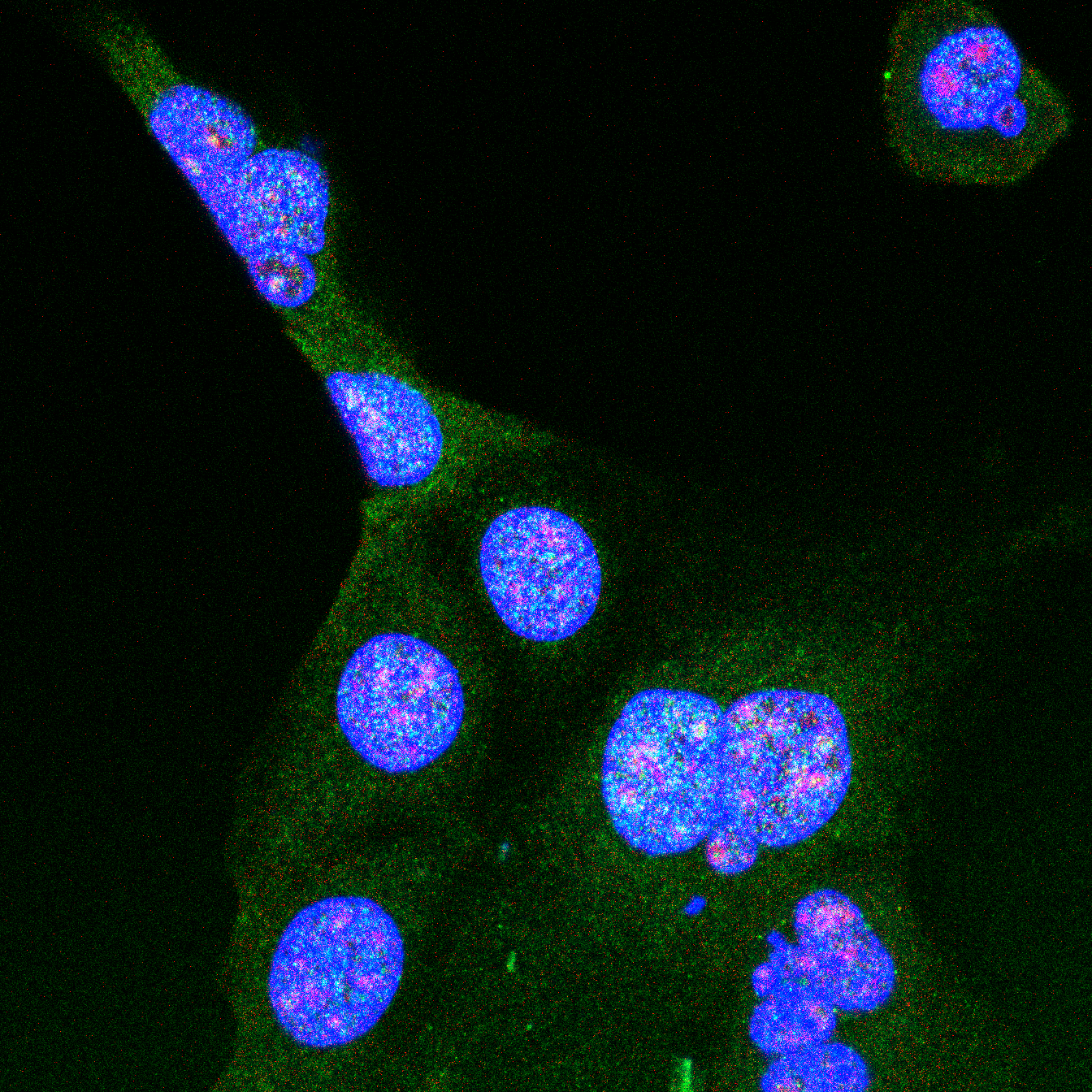

Supplement: Supplementary file 19 — Source Data for Figure 4 [file EMBJ-42-e110902-s010.zip › Figure 4/4A/KRASinactivation_Ncl siRNA1_DOX.tif]

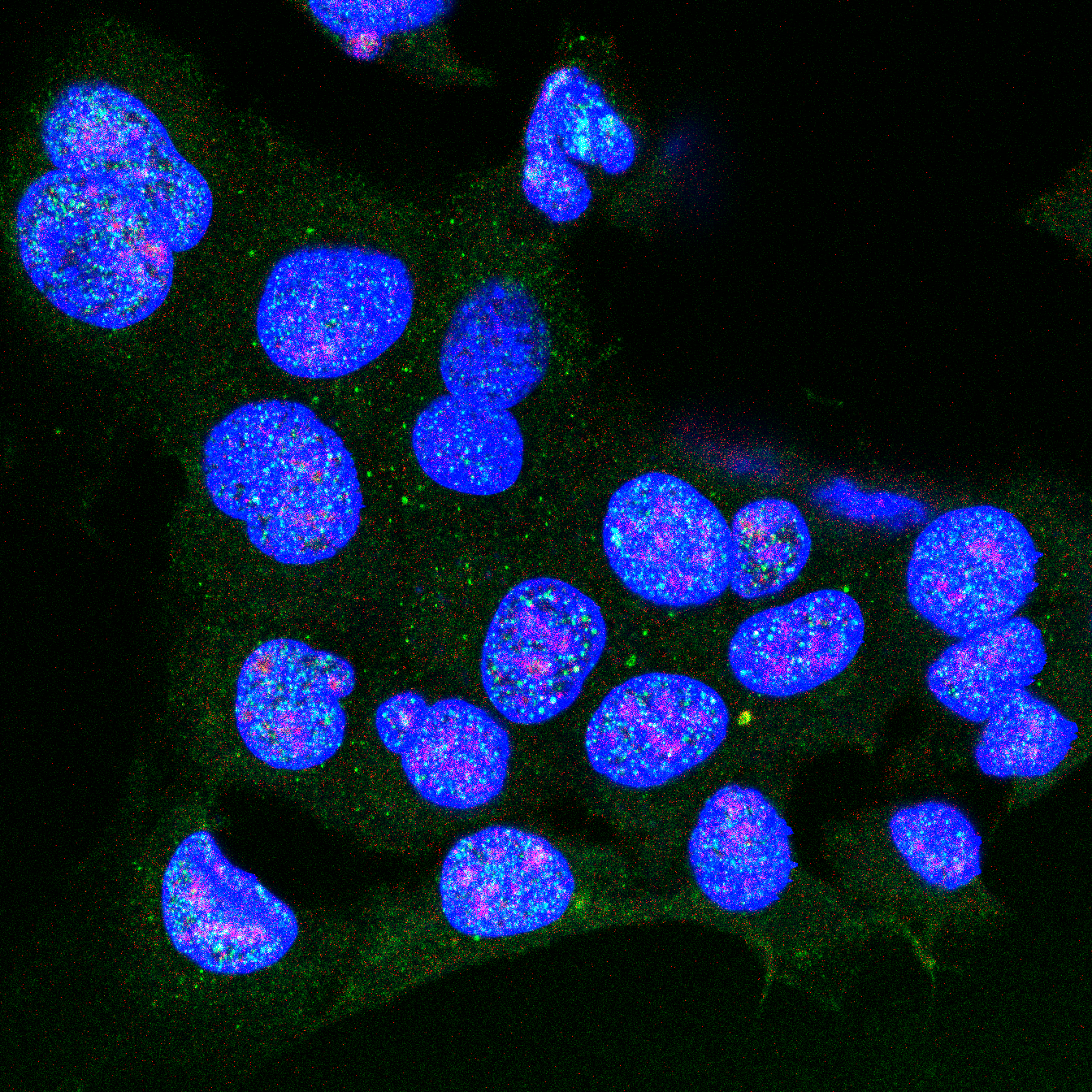

Supplement: Supplementary file 19 — Source Data for Figure 4 [file EMBJ-42-e110902-s010.zip › Figure 4/4A/KRASinactivation_Ncl siRNA1_NO-DOX.tif]

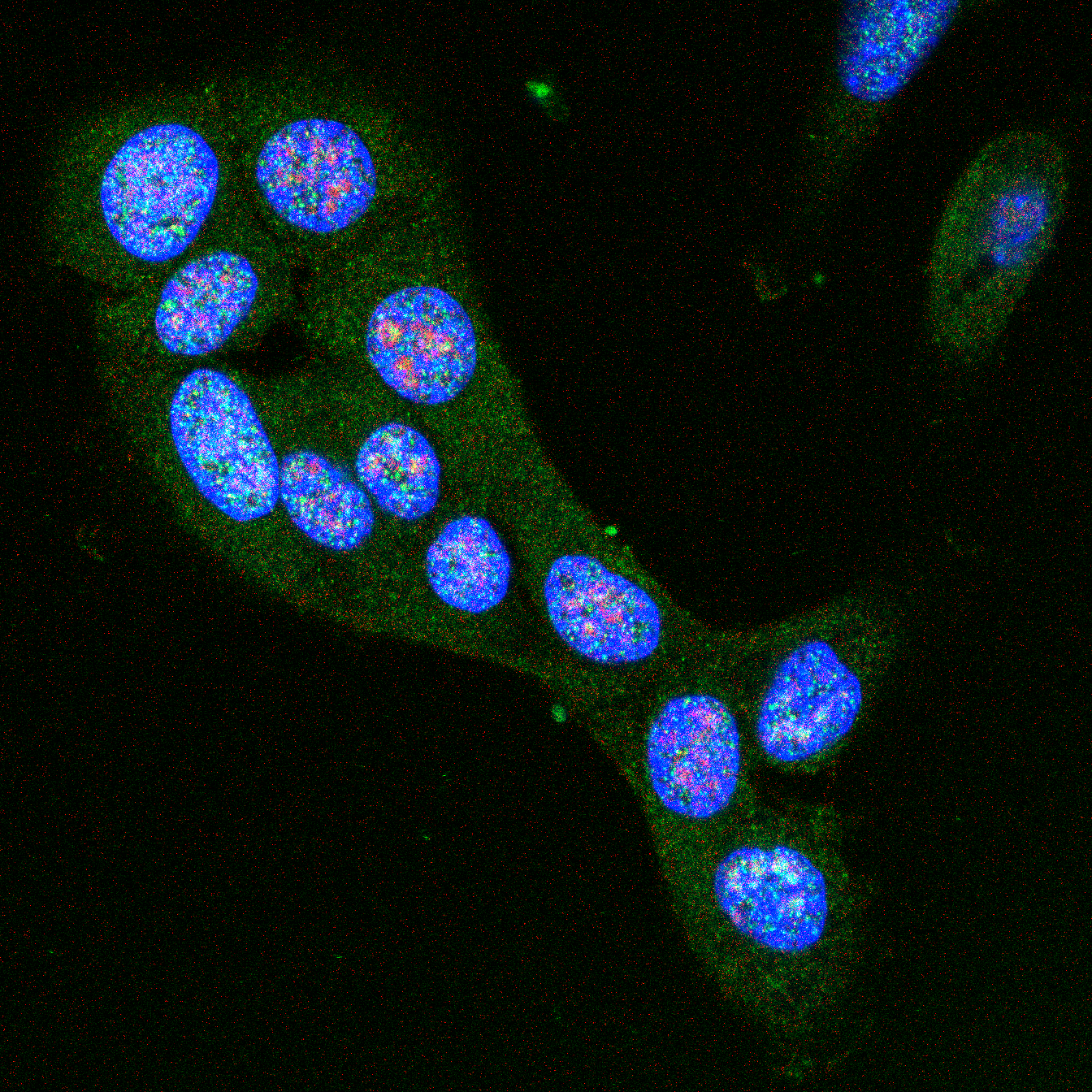

Supplement: Supplementary file 19 — Source Data for Figure 4 [file EMBJ-42-e110902-s010.zip › Figure 4/4A/KRASinactivation_Ncl siRNA2_DOX.tif]

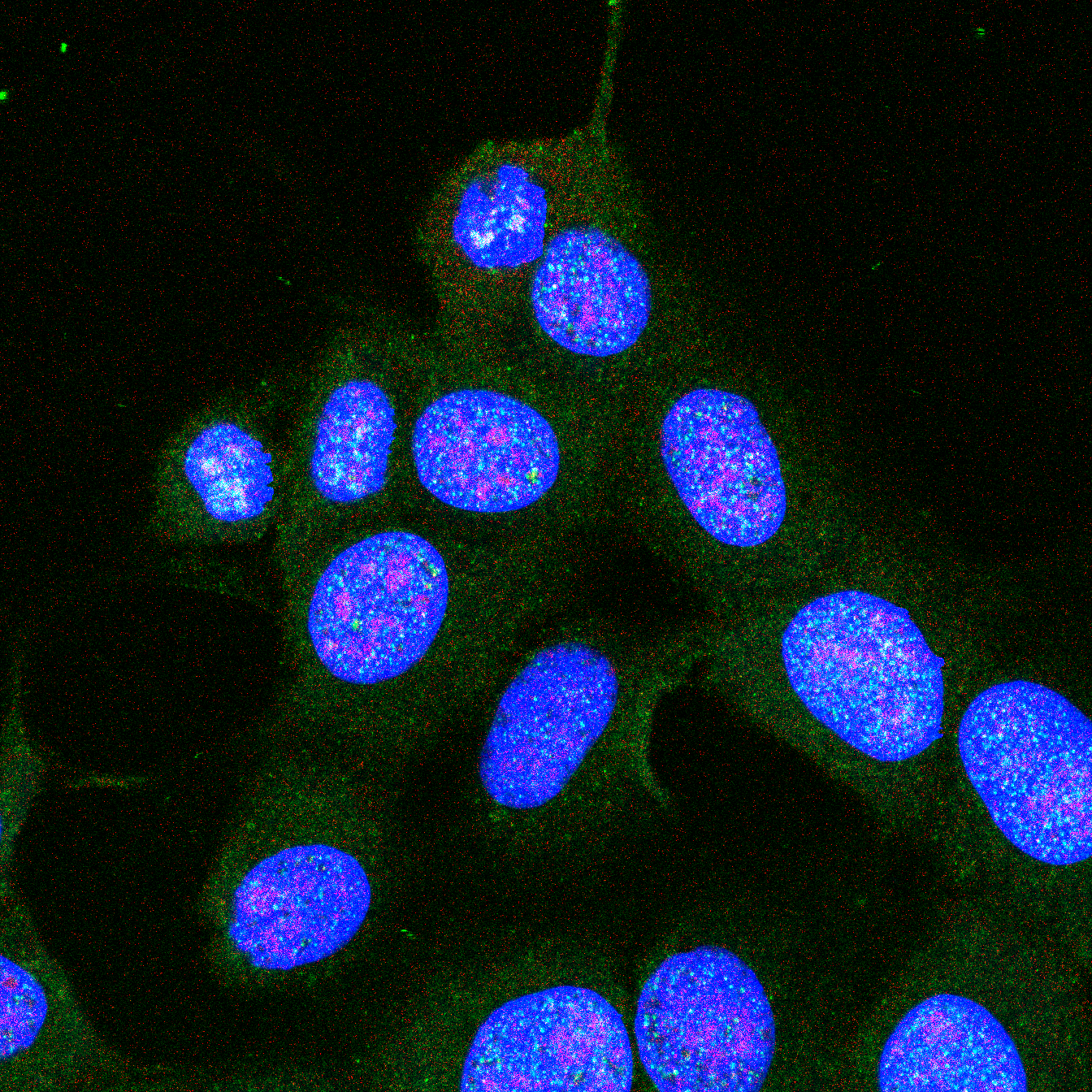

Supplement: Supplementary file 19 — Source Data for Figure 4 [file EMBJ-42-e110902-s010.zip › Figure 4/4A/KRASinactivation_Ncl siRNA2_NO-DOX.tif]

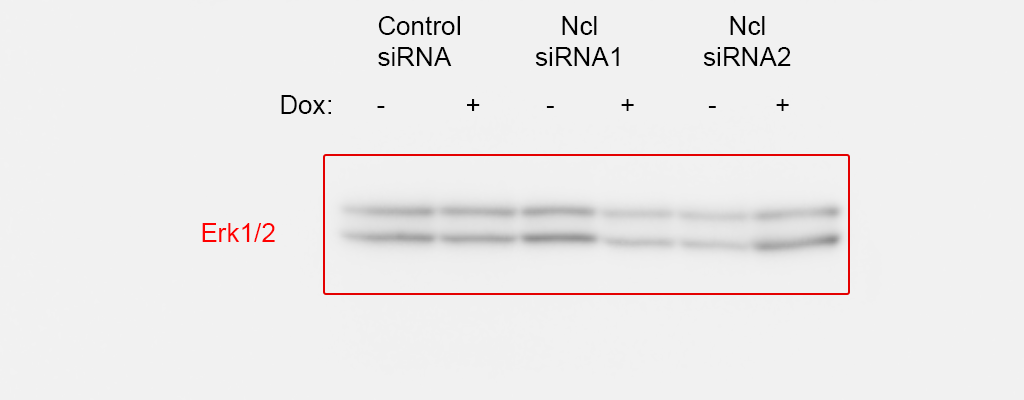

Supplement: Supplementary file 19 — Source Data for Figure 4 [file EMBJ-42-e110902-s010.zip › Figure 4/4E/Erk-channel1-chemiluminescence.tif]

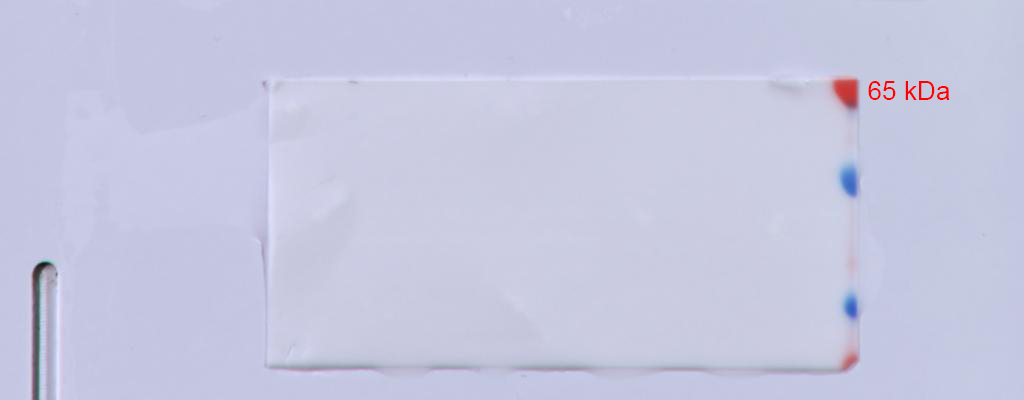

Supplement: Supplementary file 19 — Source Data for Figure 4 [file EMBJ-42-e110902-s010.zip › Figure 4/4E/Erk-channel2-Markers.jpg]

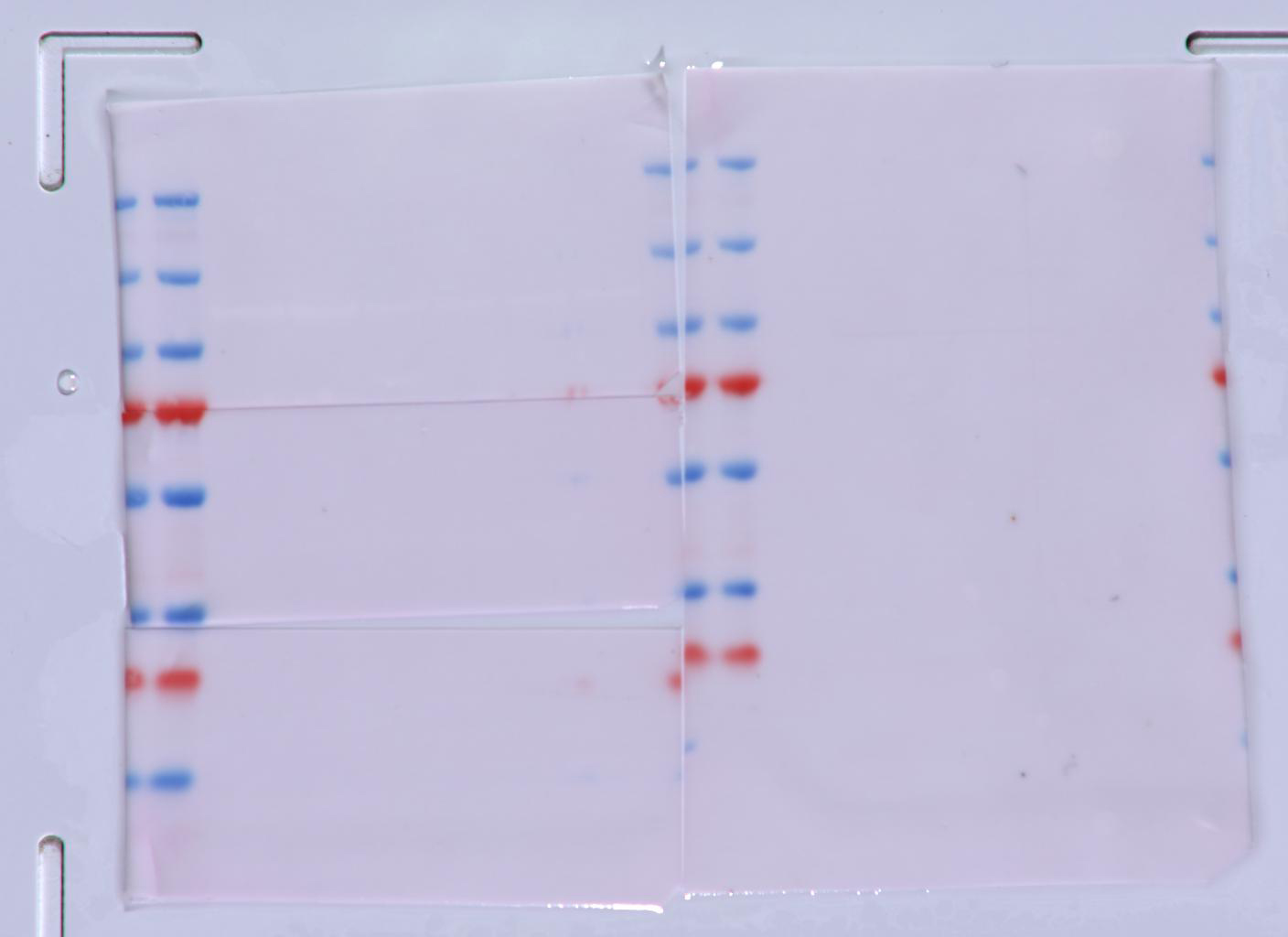

Supplement: Supplementary file 19 — Source Data for Figure 4 [file EMBJ-42-e110902-s010.zip › Figure 4/4E/Ncl_pERK_RASG12D-channel2-Markers.jpg]

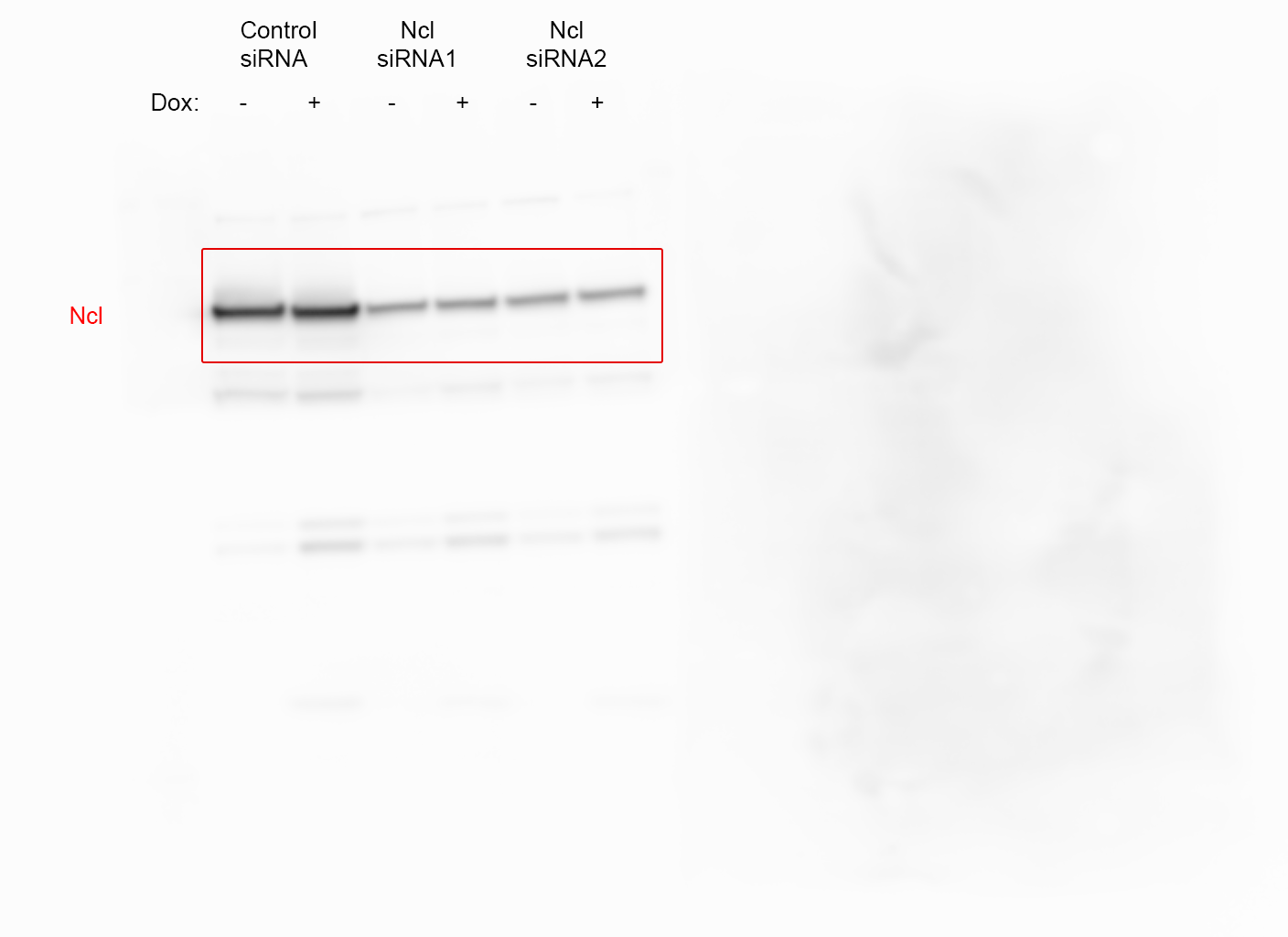

Supplement: Supplementary file 19 — Source Data for Figure 4 [file EMBJ-42-e110902-s010.zip › Figure 4/4E/Ncl-channel1-chemiluminescence.tif]

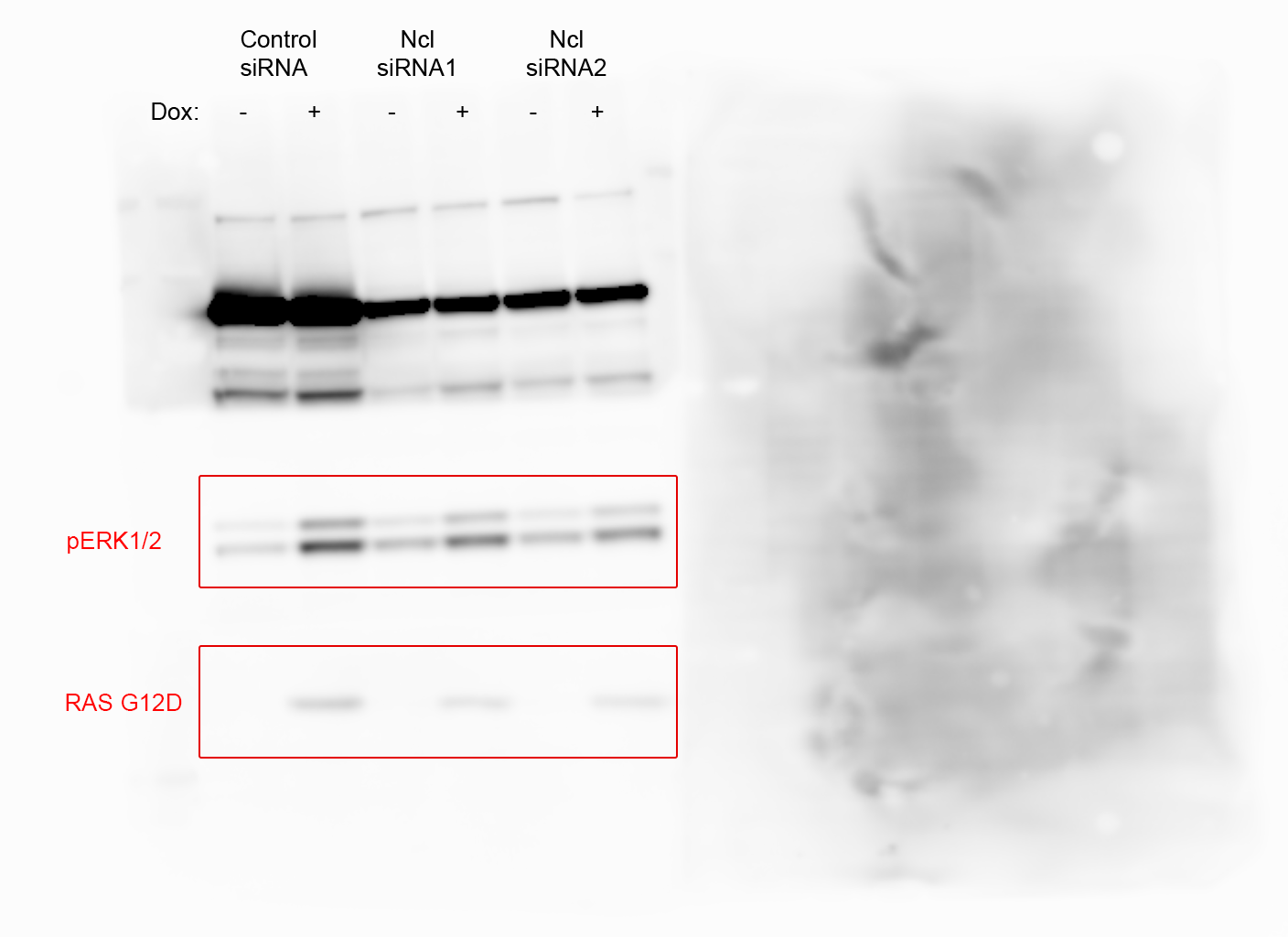

Supplement: Supplementary file 19 — Source Data for Figure 4 [file EMBJ-42-e110902-s010.zip › Figure 4/4E/pErk_RasG12D-channel1-chemiluminescence.tif]

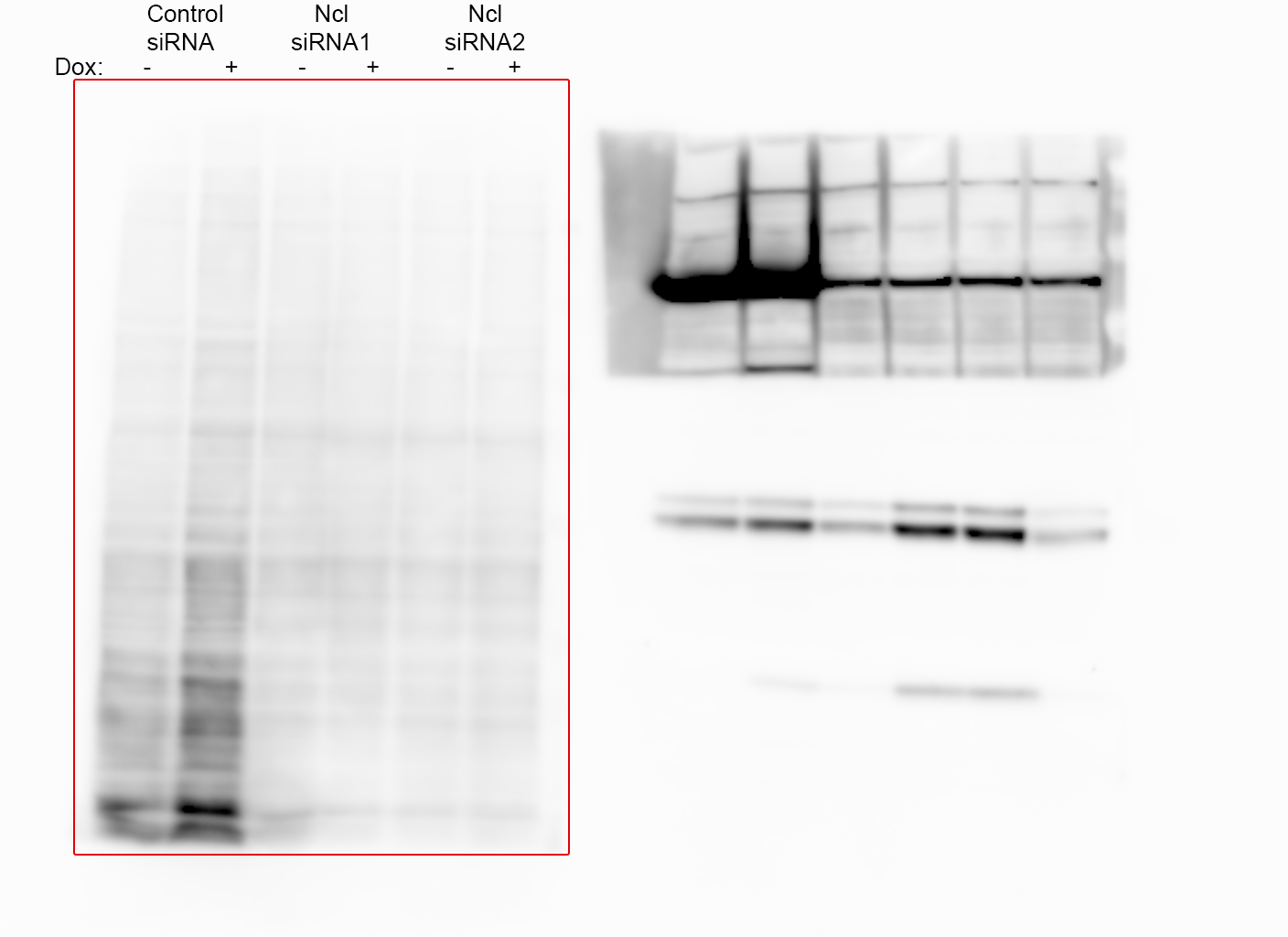

Supplement: Supplementary file 19 — Source Data for Figure 4 [file EMBJ-42-e110902-s010.zip › Figure 4/4E/Puromycin-channel1-chemiluminescence.tif]

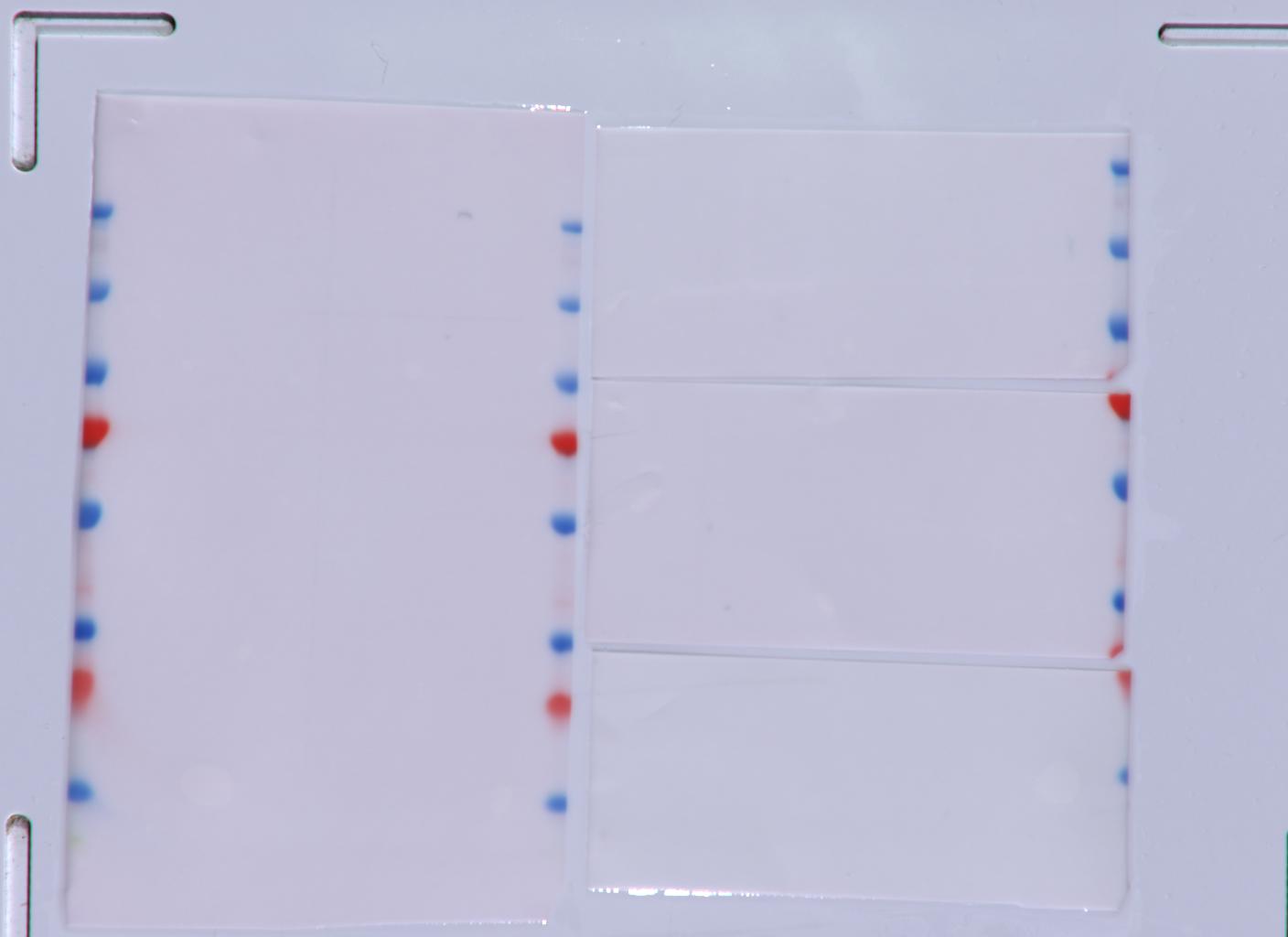

Supplement: Supplementary file 19 — Source Data for Figure 4 [file EMBJ-42-e110902-s010.zip › Figure 4/4E/Puromycin-channel2-markers.jpg]

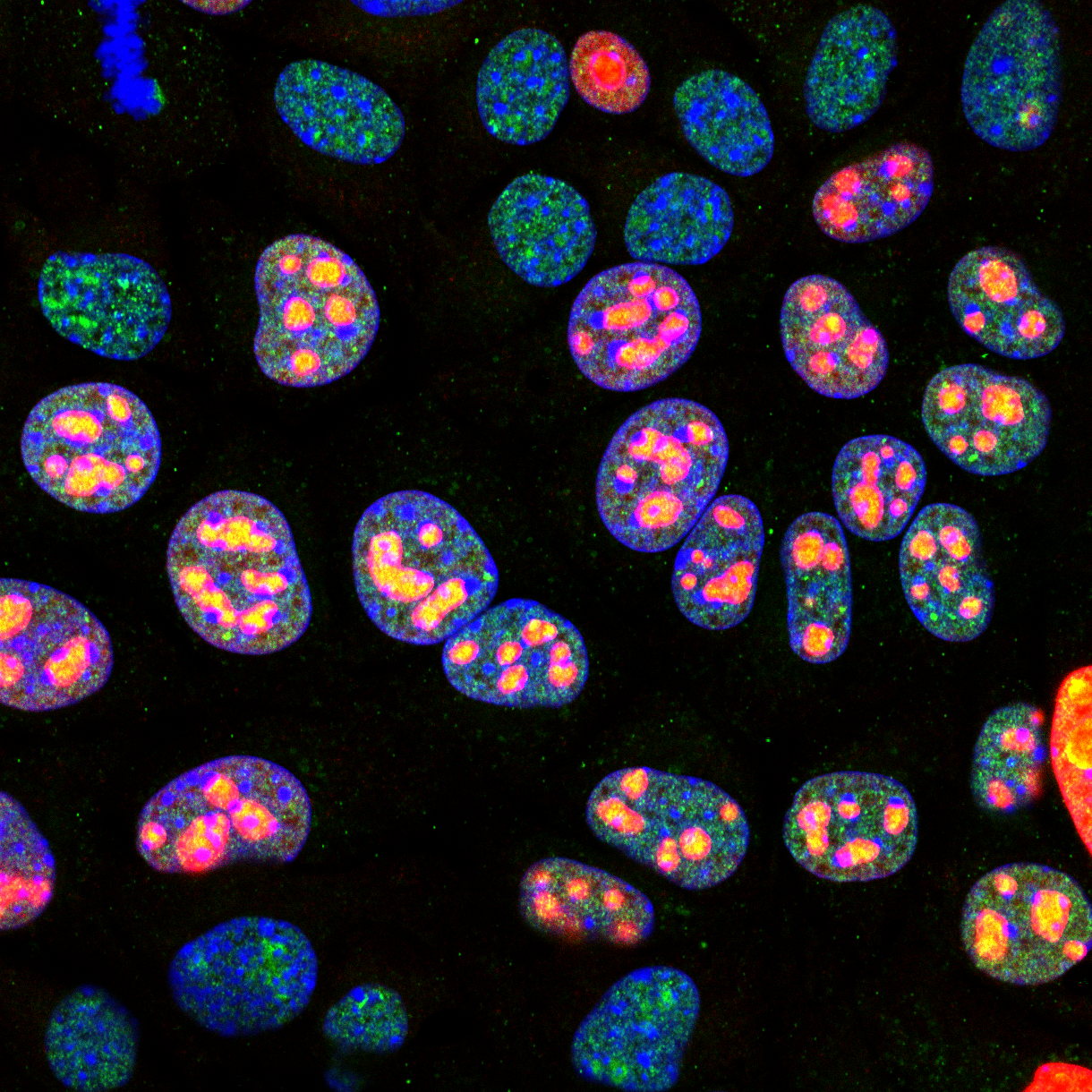

Supplement: Supplementary file 19 — Source Data for Figure 4 [file EMBJ-42-e110902-s010.zip › Figure 4/4G/MycNcl_Mutants_S4A_DOX.tif]

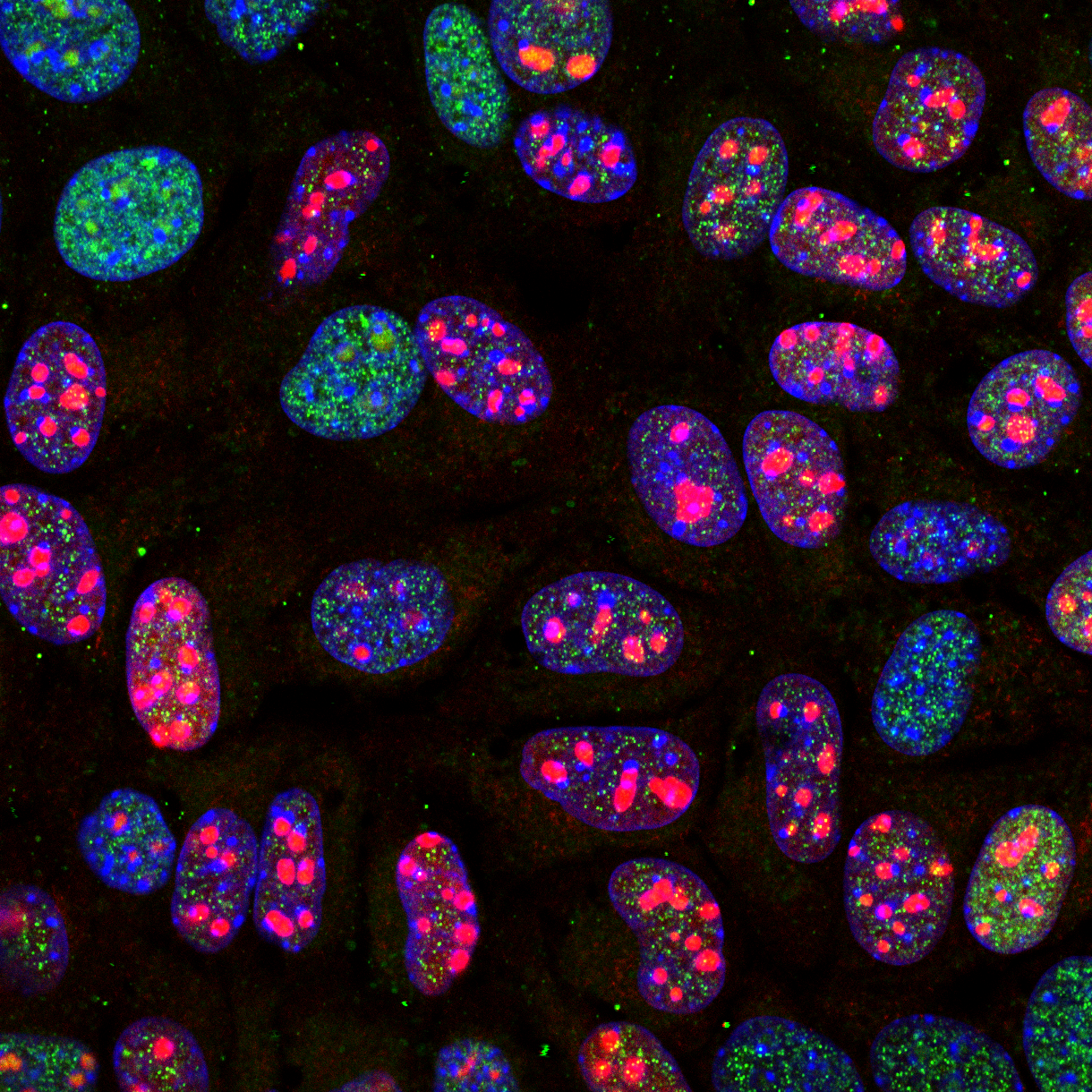

Supplement: Supplementary file 19 — Source Data for Figure 4 [file EMBJ-42-e110902-s010.zip › Figure 4/4G/MycNcl_Mutants_S4A_NO DOX.tif]

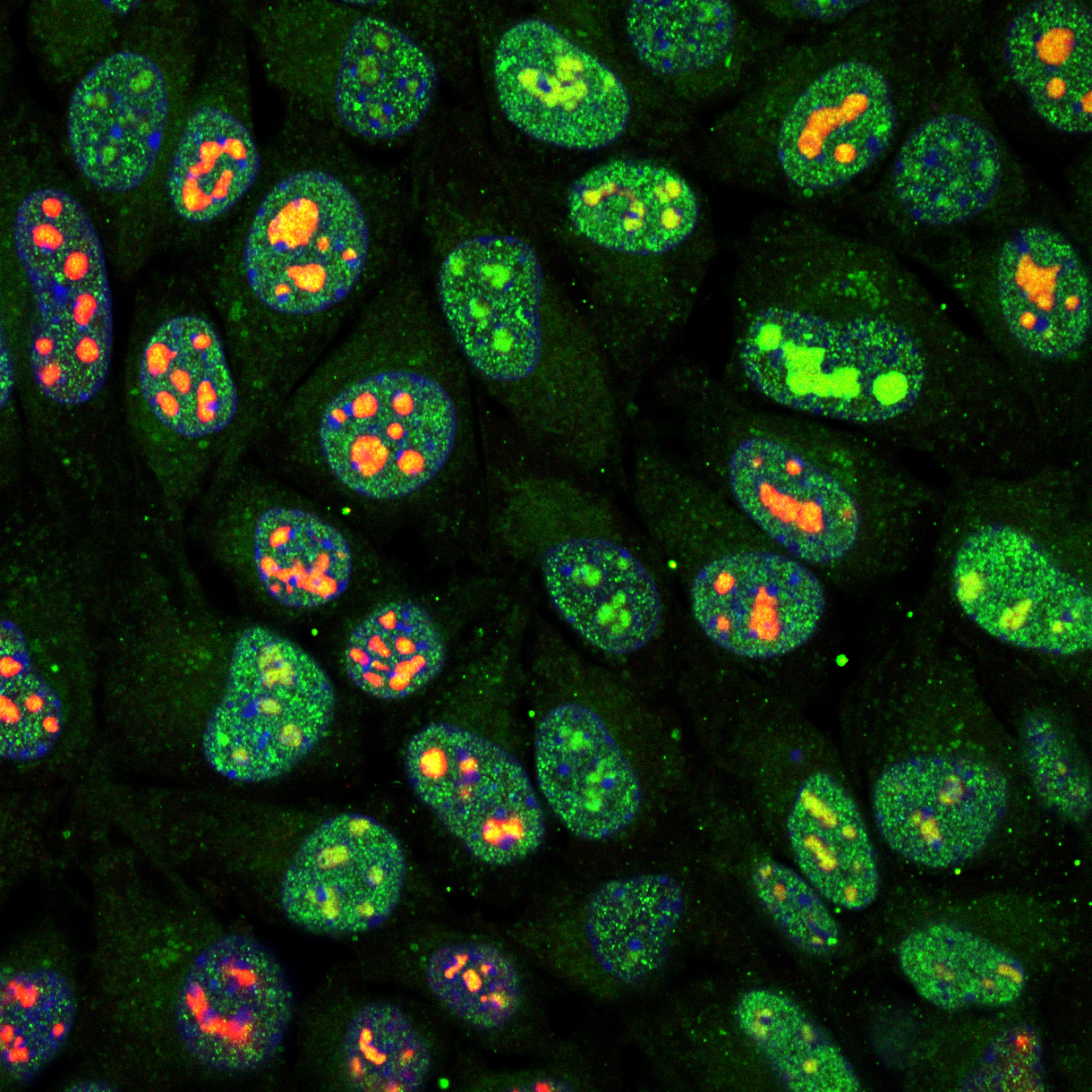

Supplement: Supplementary file 19 — Source Data for Figure 4 [file EMBJ-42-e110902-s010.zip › Figure 4/4G/MycNcl_Mutants_S4D_DOX.tif]

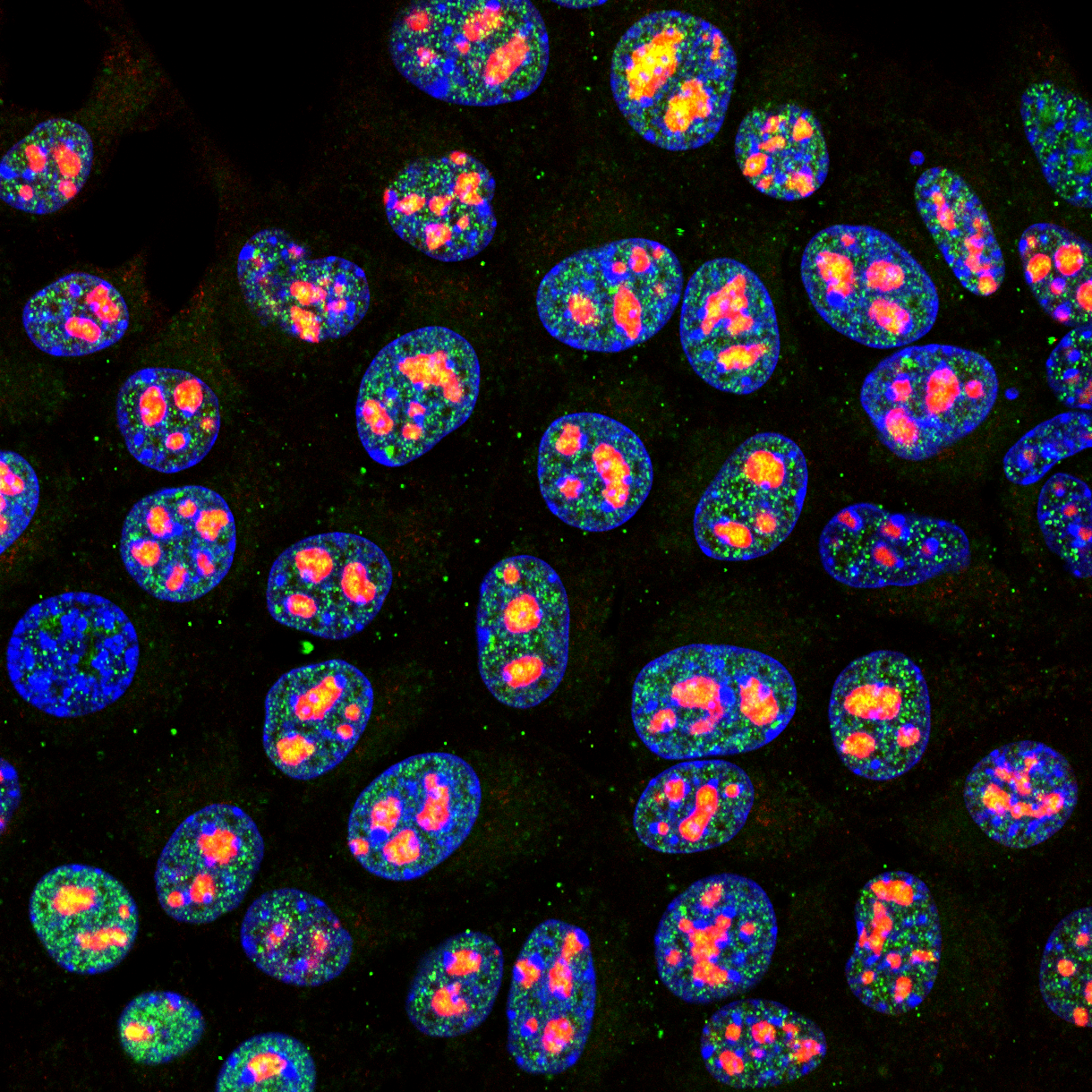

Supplement: Supplementary file 19 — Source Data for Figure 4 [file EMBJ-42-e110902-s010.zip › Figure 4/4G/MycNcl_Mutants_S4D_NO DOX.tif]

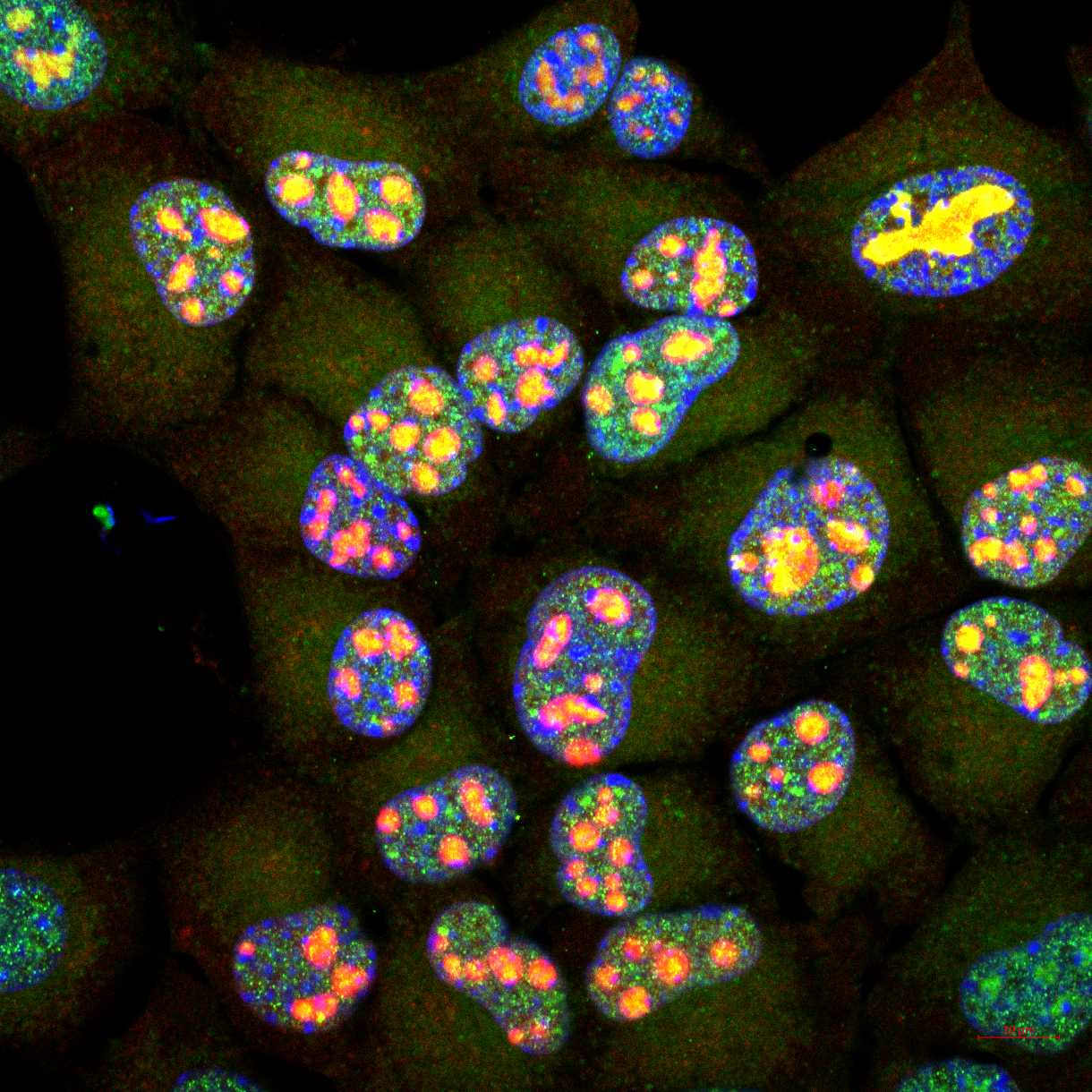

Supplement: Supplementary file 19 — Source Data for Figure 4 [file EMBJ-42-e110902-s010.zip › Figure 4/4G/MycNcl_Mutants_WT_DOX.tif]

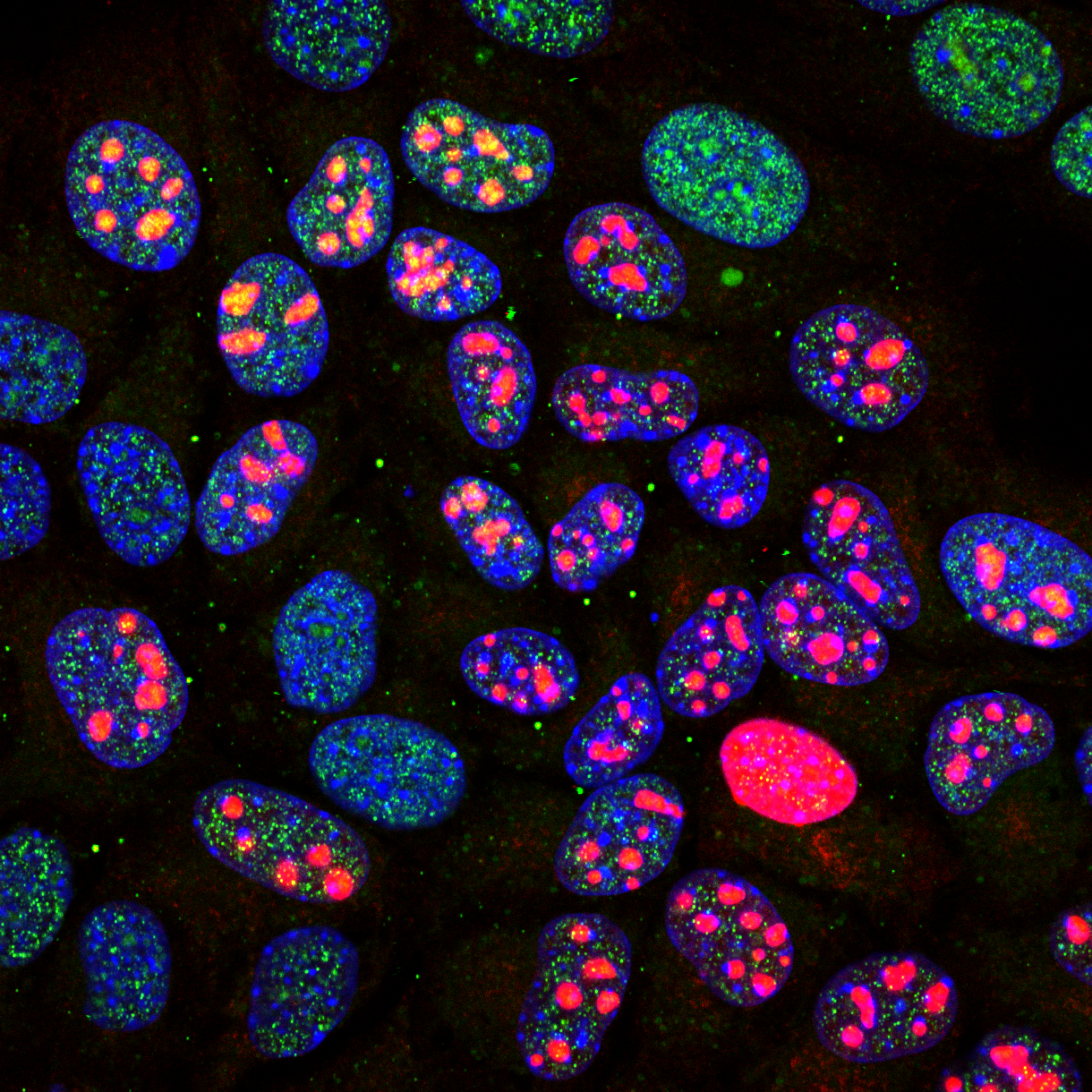

Supplement: Supplementary file 19 — Source Data for Figure 4 [file EMBJ-42-e110902-s010.zip › Figure 4/4G/MycNcl_Mutants_WT_NO DOX.tif]

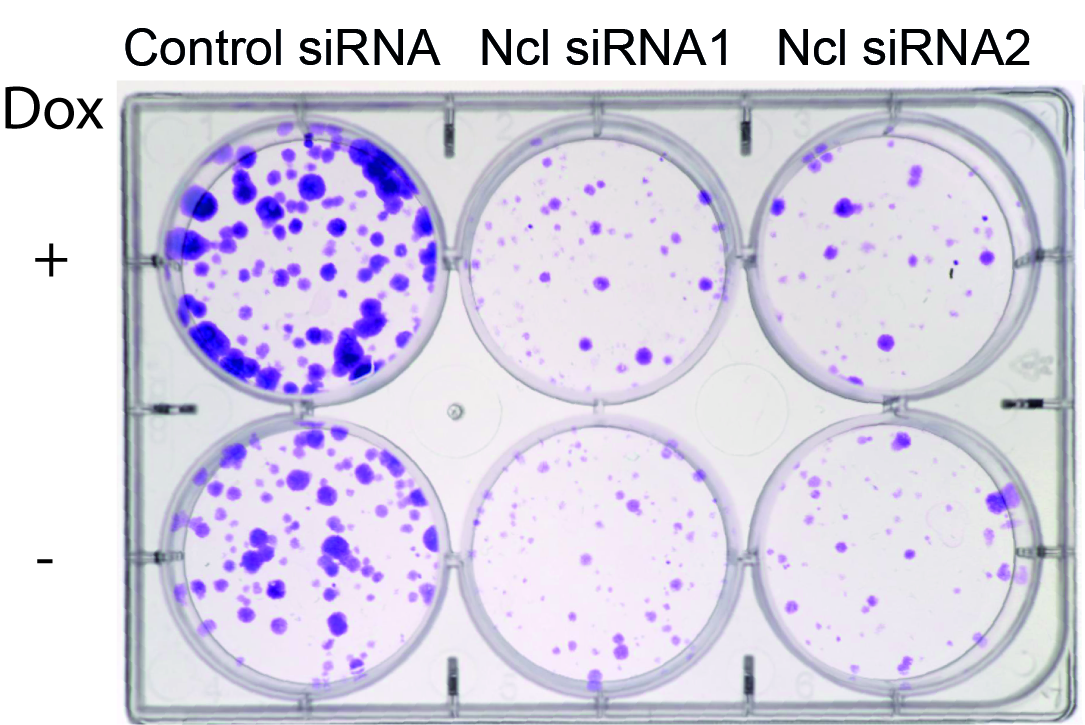

Supplement: Supplementary file 20 — Source Data for Figure 5 [file EMBJ-42-e110902-s022.zip › Figure 5/5A/Colony_Formation_Ncl_KD_2D_vs_3D_Colour_ copy.tif]

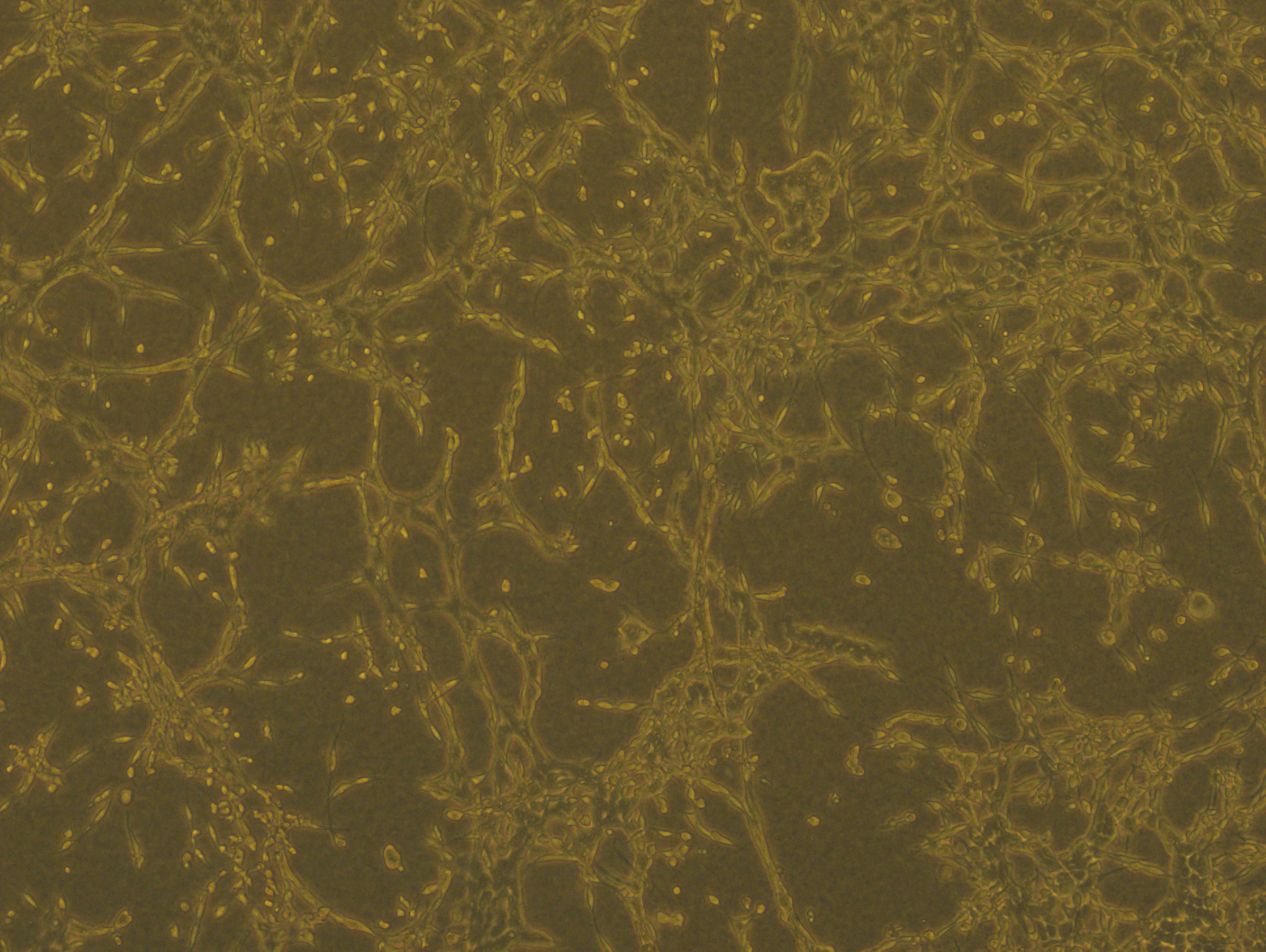

Supplement: Supplementary file 20 — Source Data for Figure 5 [file EMBJ-42-e110902-s022.zip › Figure 5/5C/Control siRNA_DOX.tif]

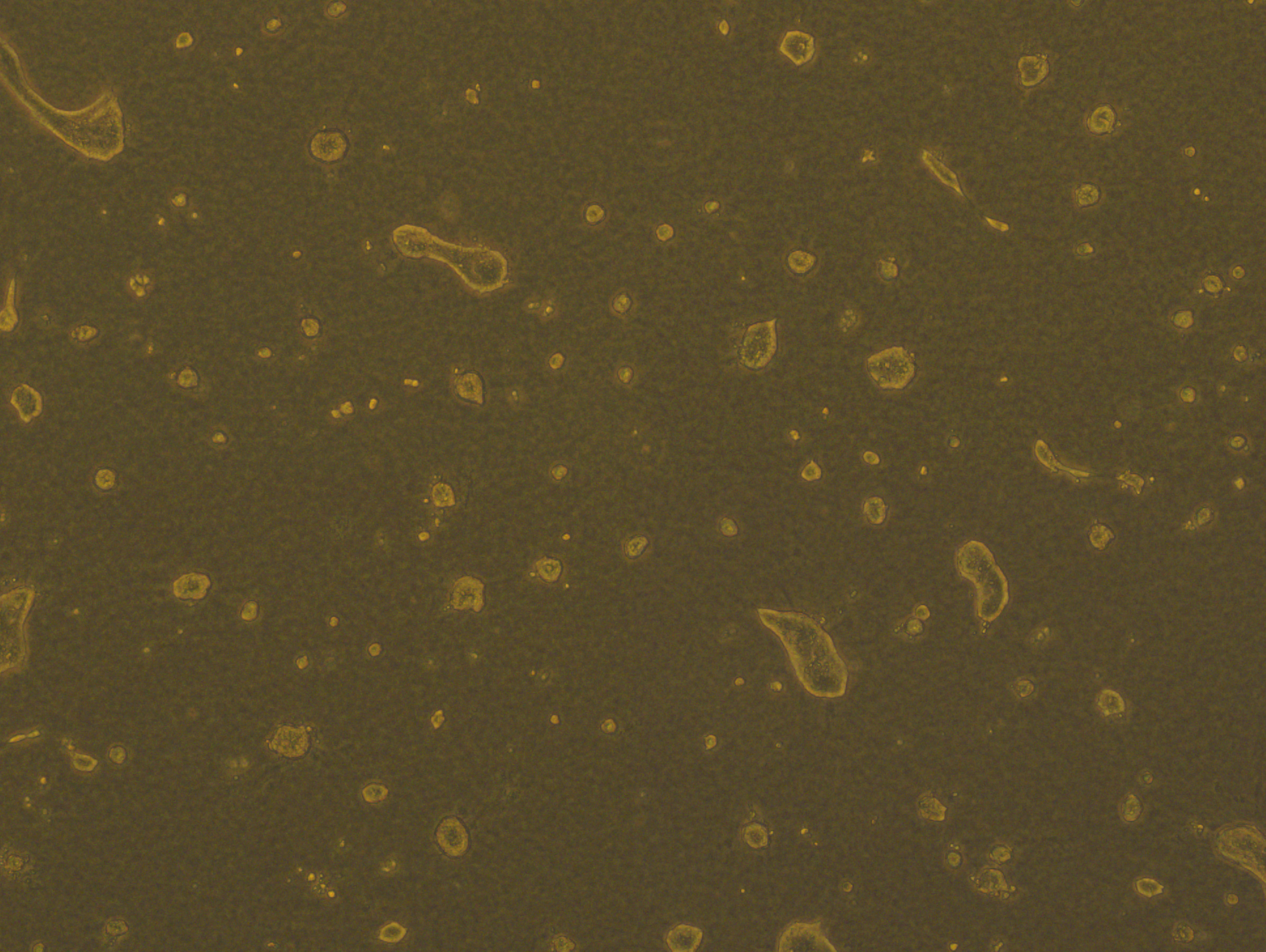

Supplement: Supplementary file 20 — Source Data for Figure 5 [file EMBJ-42-e110902-s022.zip › Figure 5/5C/Control siRNA_NODOX.tif]

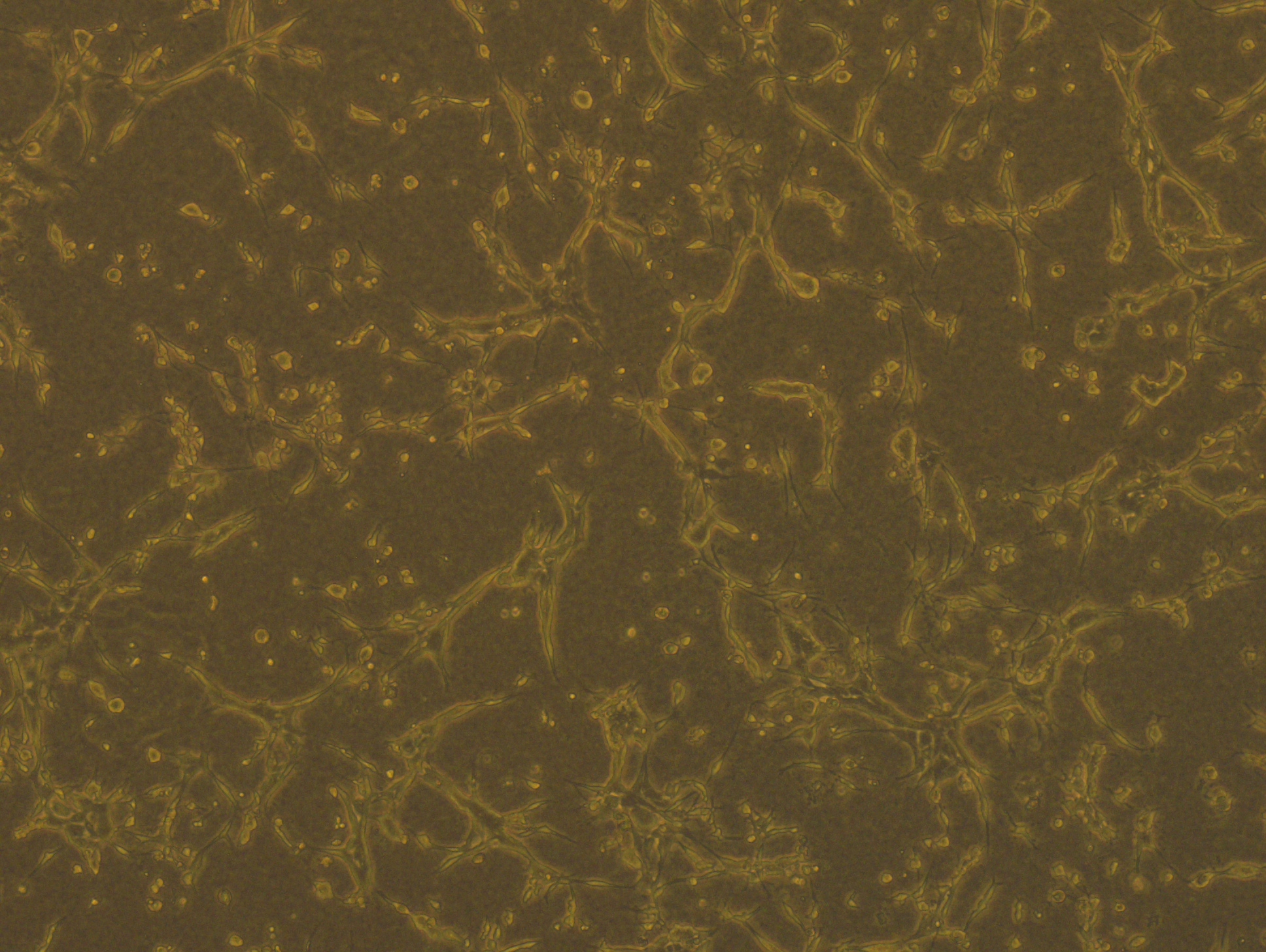

Supplement: Supplementary file 20 — Source Data for Figure 5 [file EMBJ-42-e110902-s022.zip › Figure 5/5C/Ncl siRNA1_DOX.tif]

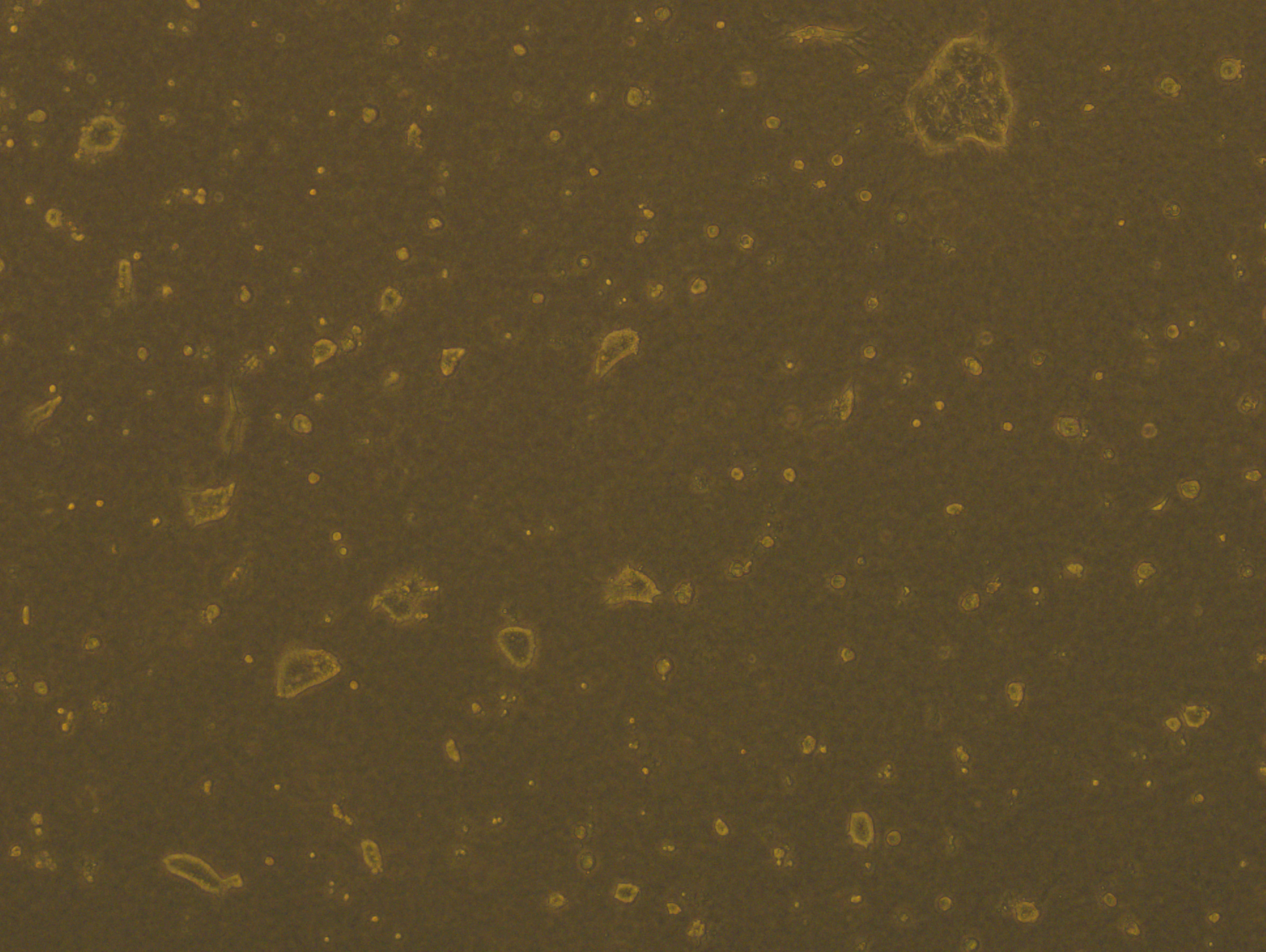

Supplement: Supplementary file 20 — Source Data for Figure 5 [file EMBJ-42-e110902-s022.zip › Figure 5/5C/Ncl siRNA1_NODOX.tif]

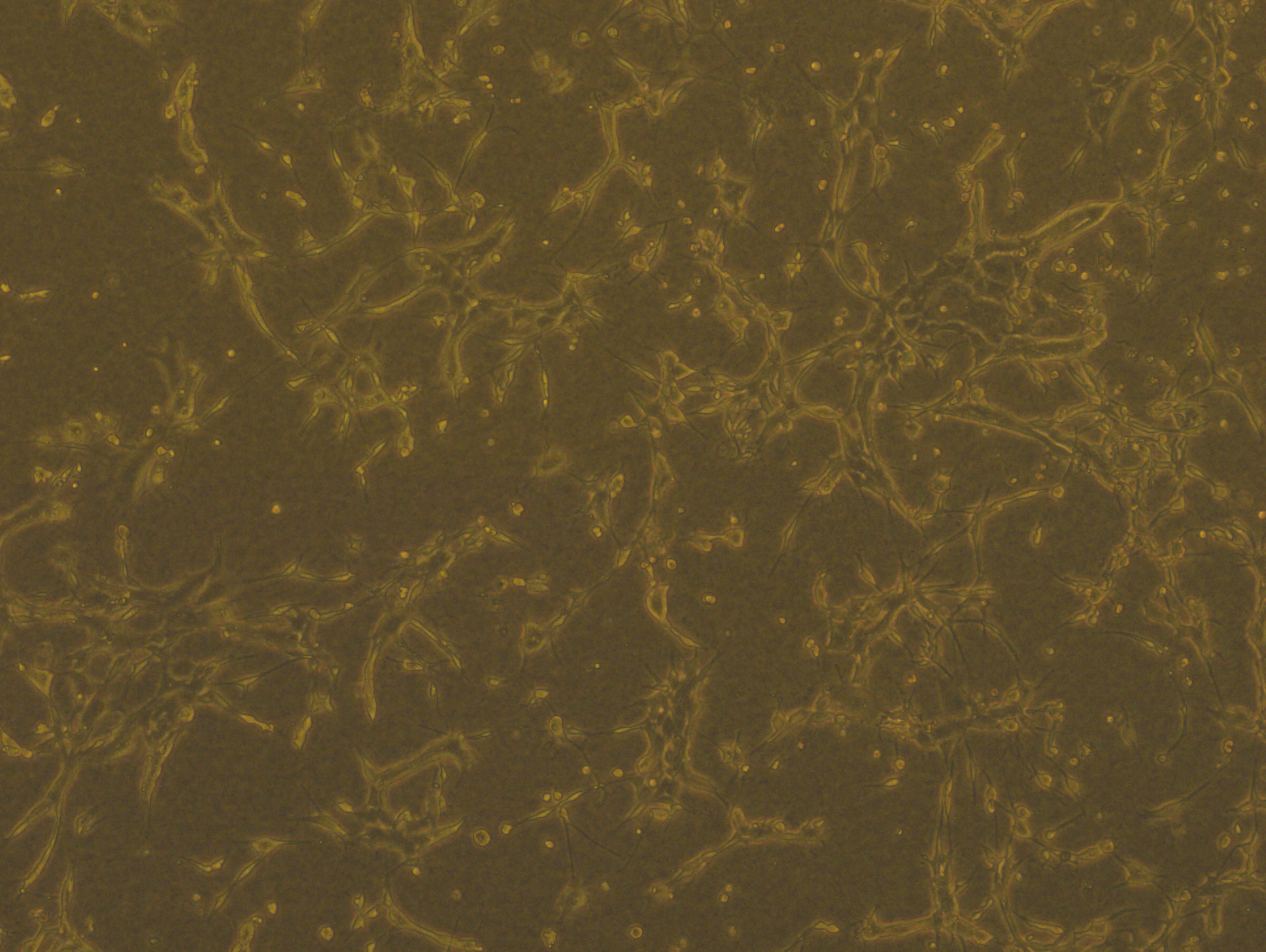

Supplement: Supplementary file 20 — Source Data for Figure 5 [file EMBJ-42-e110902-s022.zip › Figure 5/5C/Ncl siRNA2_DOX.tif]

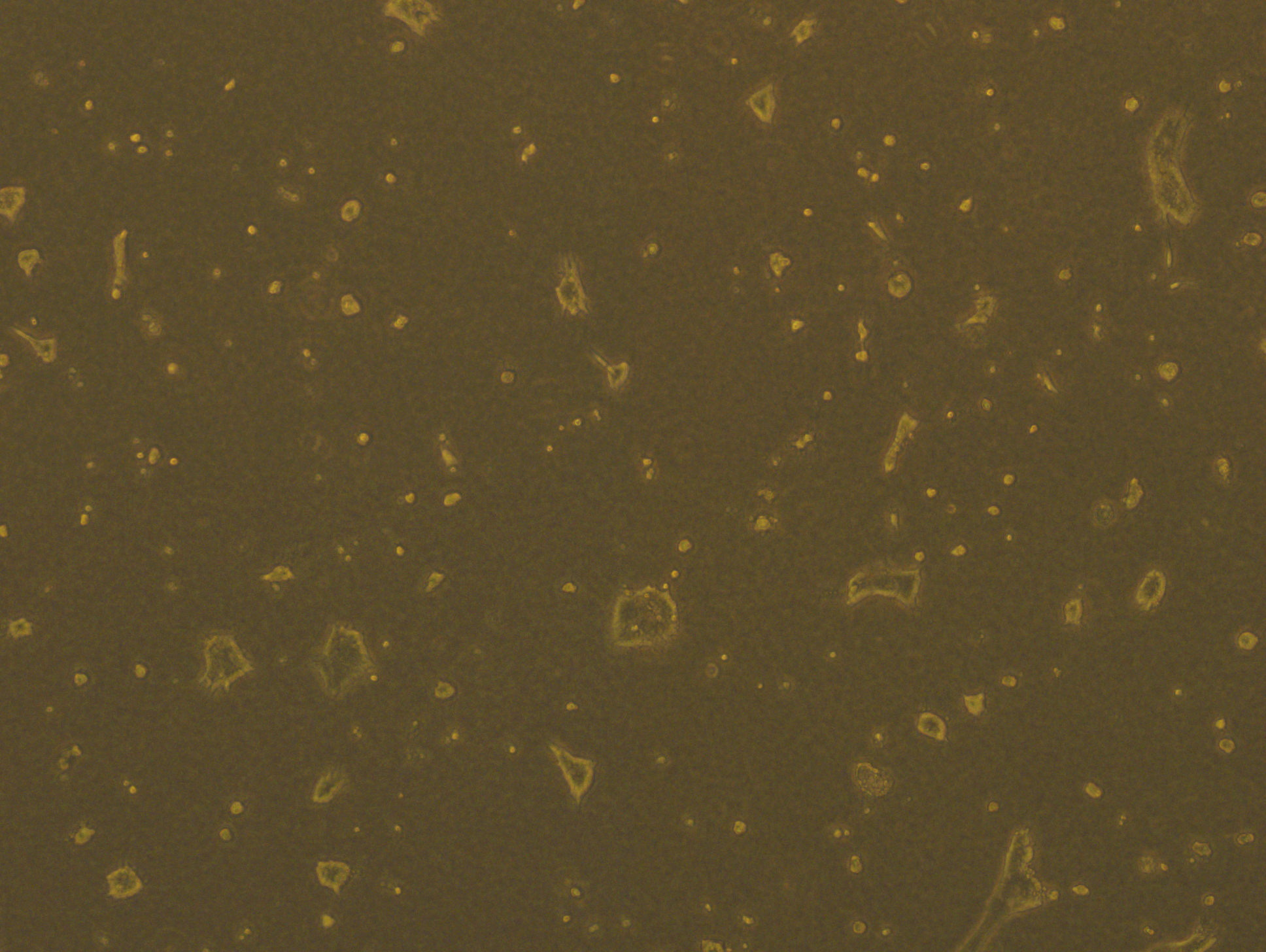

Supplement: Supplementary file 20 — Source Data for Figure 5 [file EMBJ-42-e110902-s022.zip › Figure 5/5C/Ncl siRNA2_NODOX.tif]

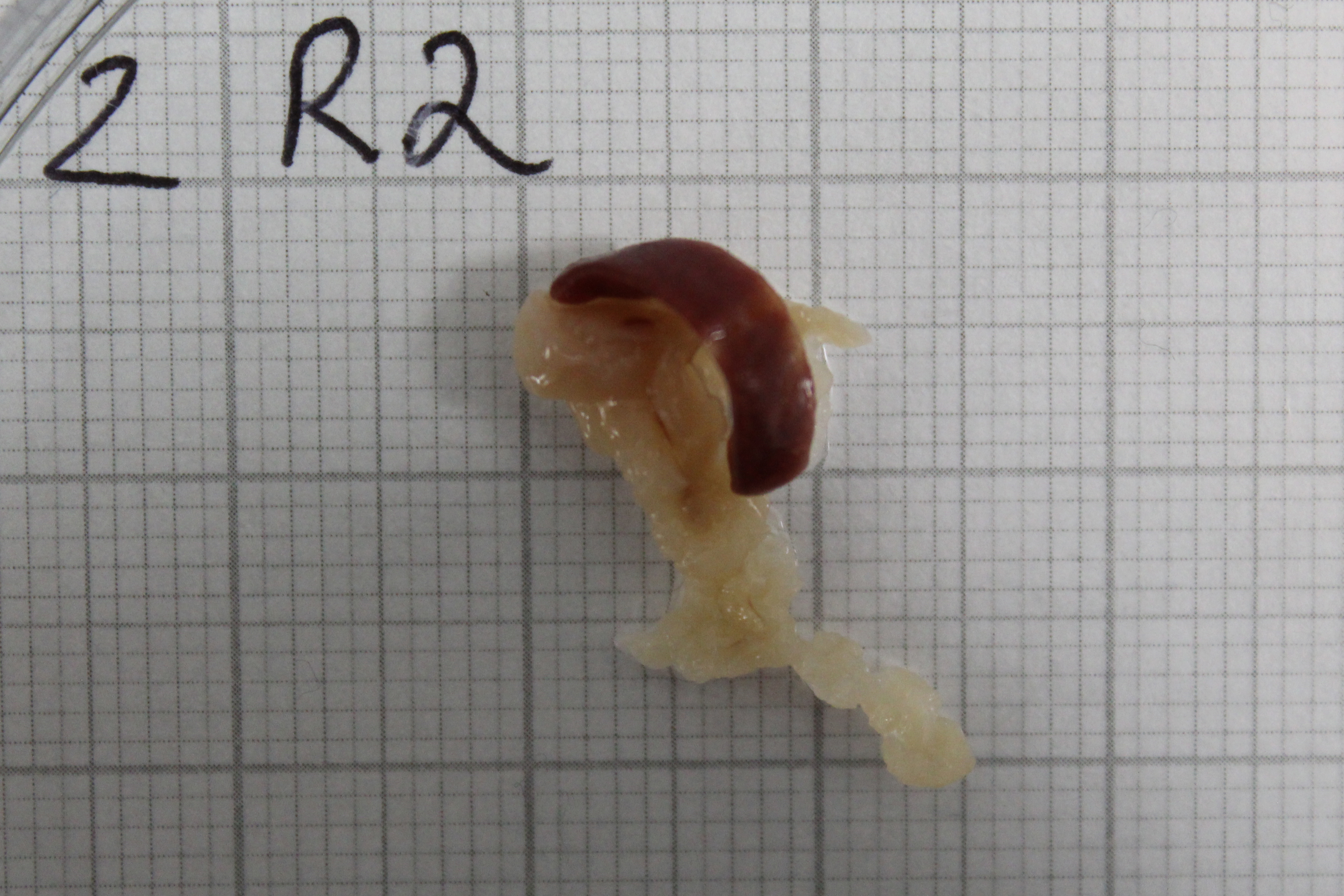

Supplement: Supplementary file 20 — Source Data for Figure 5 [file EMBJ-42-e110902-s022.zip › Figure 5/5E/Control siRNA_KRAS OFF.JPG]

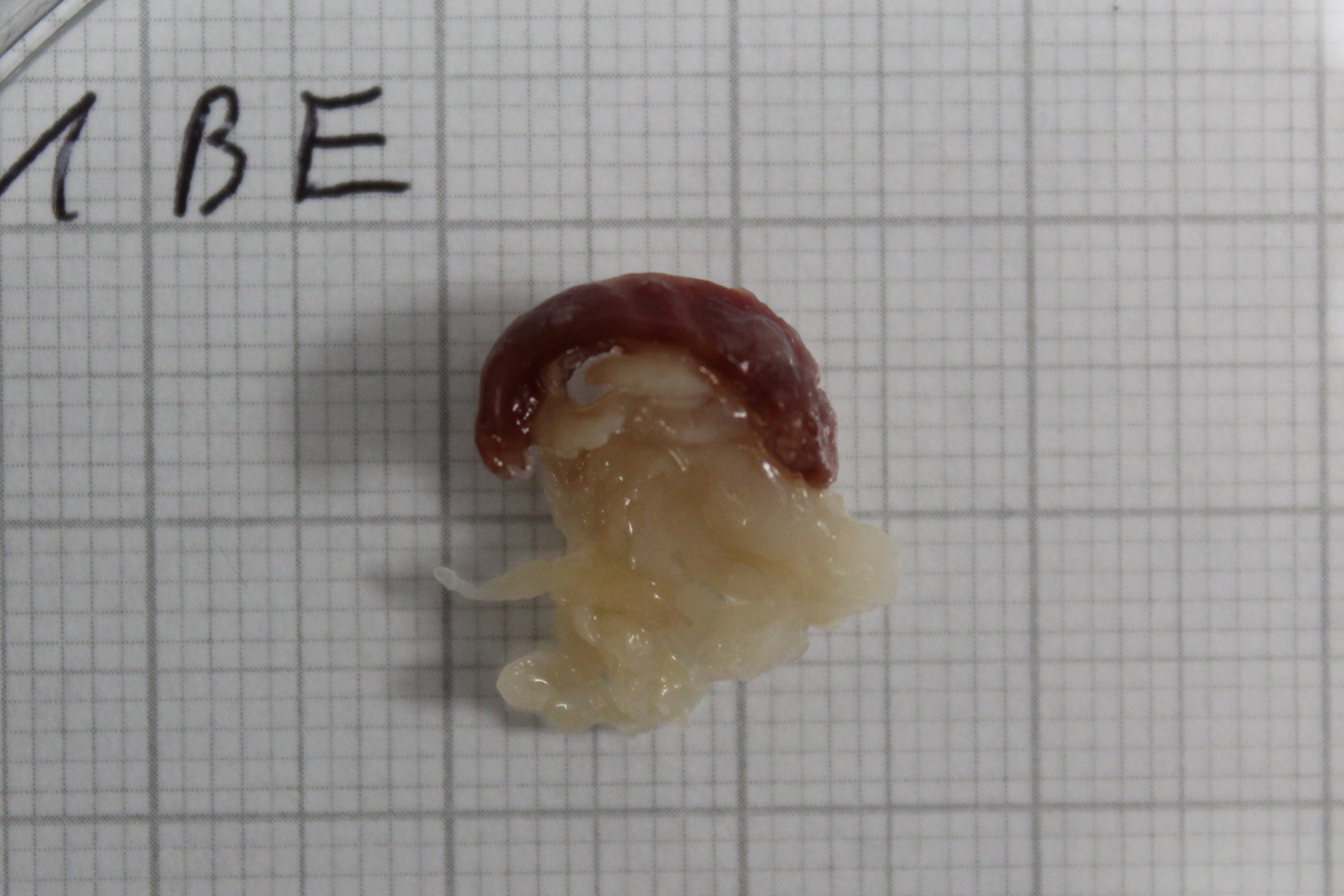

Supplement: Supplementary file 20 — Source Data for Figure 5 [file EMBJ-42-e110902-s022.zip › Figure 5/5E/Control siRNA_KRAS ON.JPG]

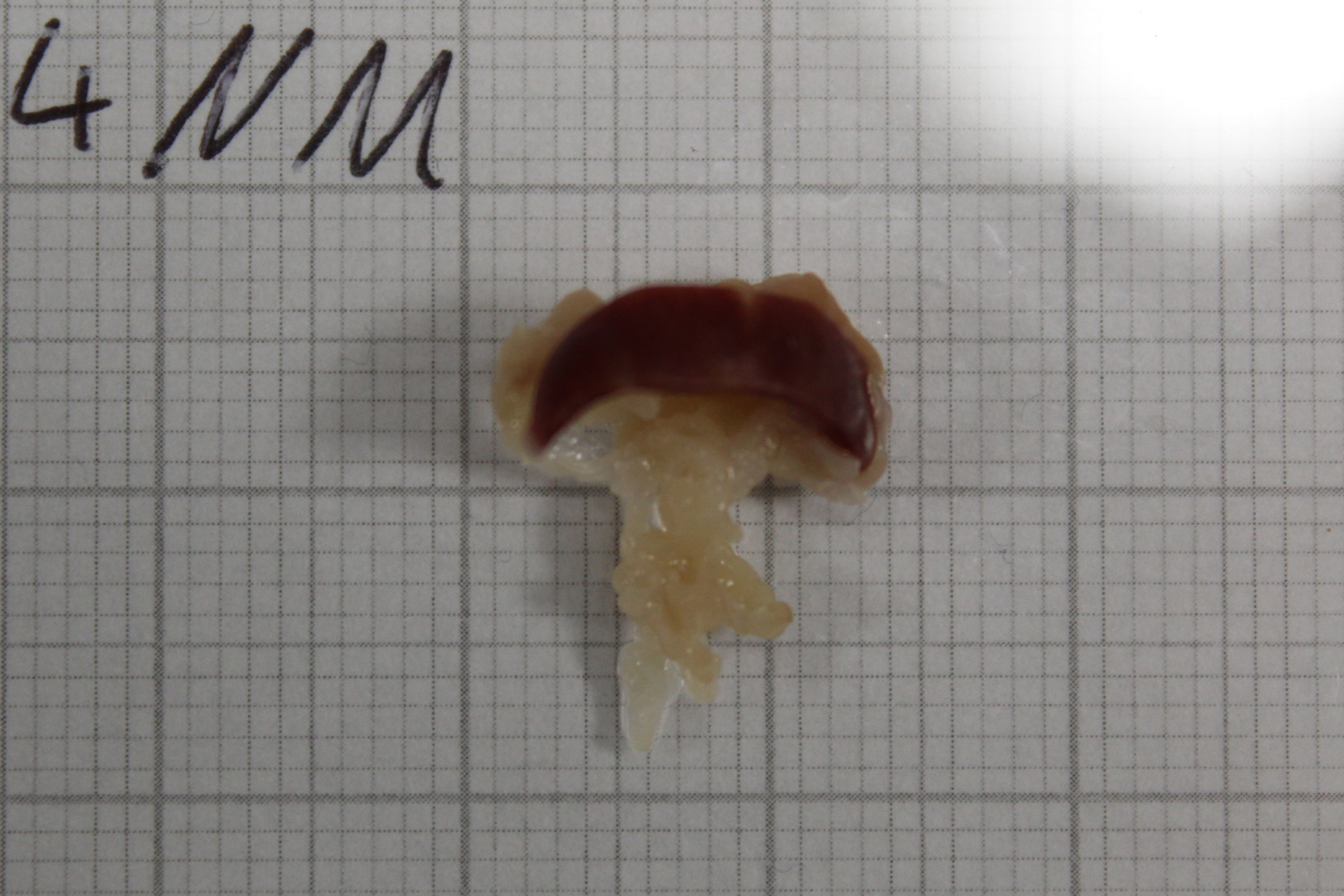

Supplement: Supplementary file 20 — Source Data for Figure 5 [file EMBJ-42-e110902-s022.zip › Figure 5/5E/Ncl siRNA_KRAS OFF.JPG]

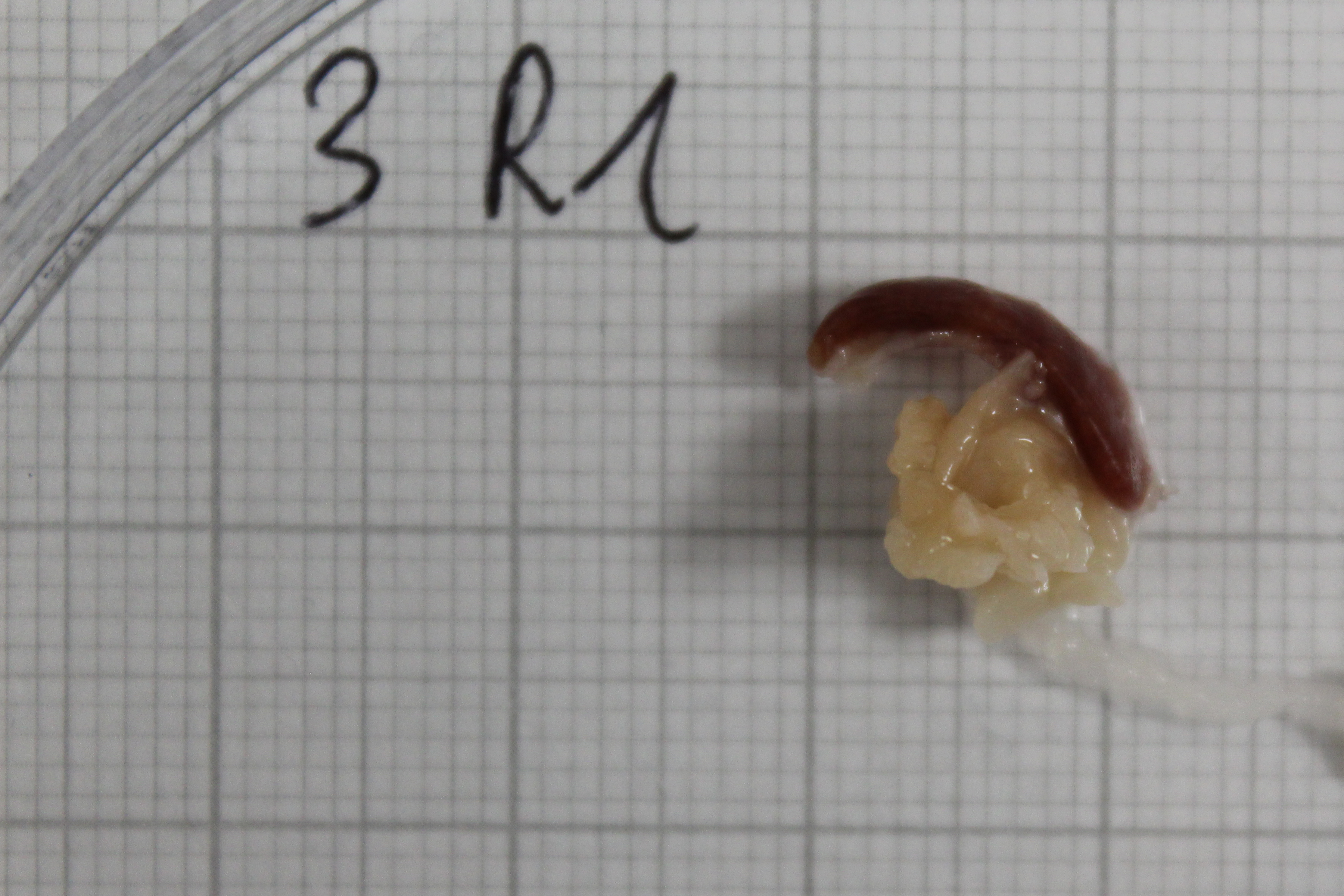

Supplement: Supplementary file 20 — Source Data for Figure 5 [file EMBJ-42-e110902-s022.zip › Figure 5/5E/Ncl siRNA_KRAS ON.JPG]

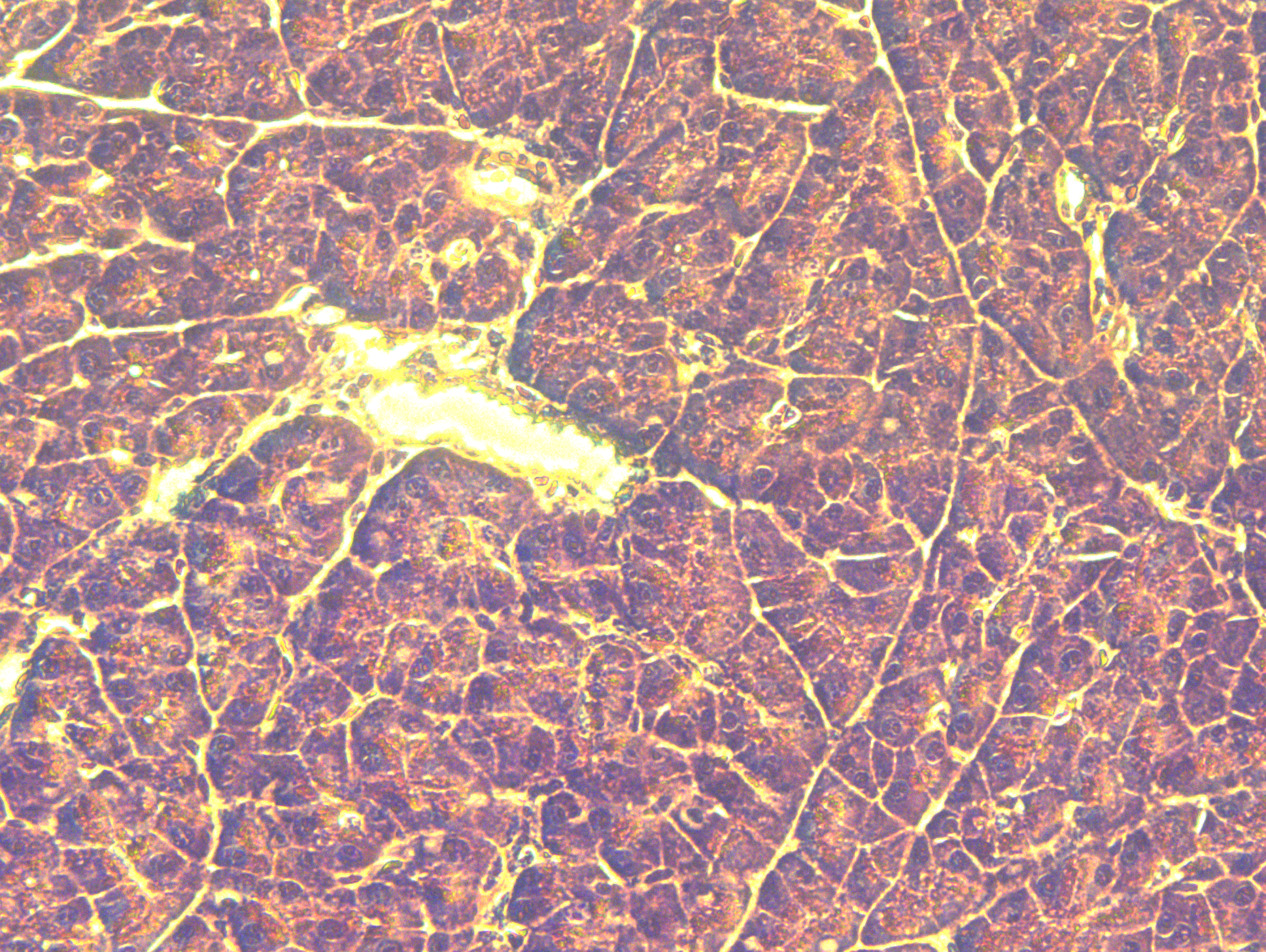

Supplement: Supplementary file 20 — Source Data for Figure 5 [file EMBJ-42-e110902-s022.zip › Figure 5/5G/Control siRNA - KRAS OFF.tif]

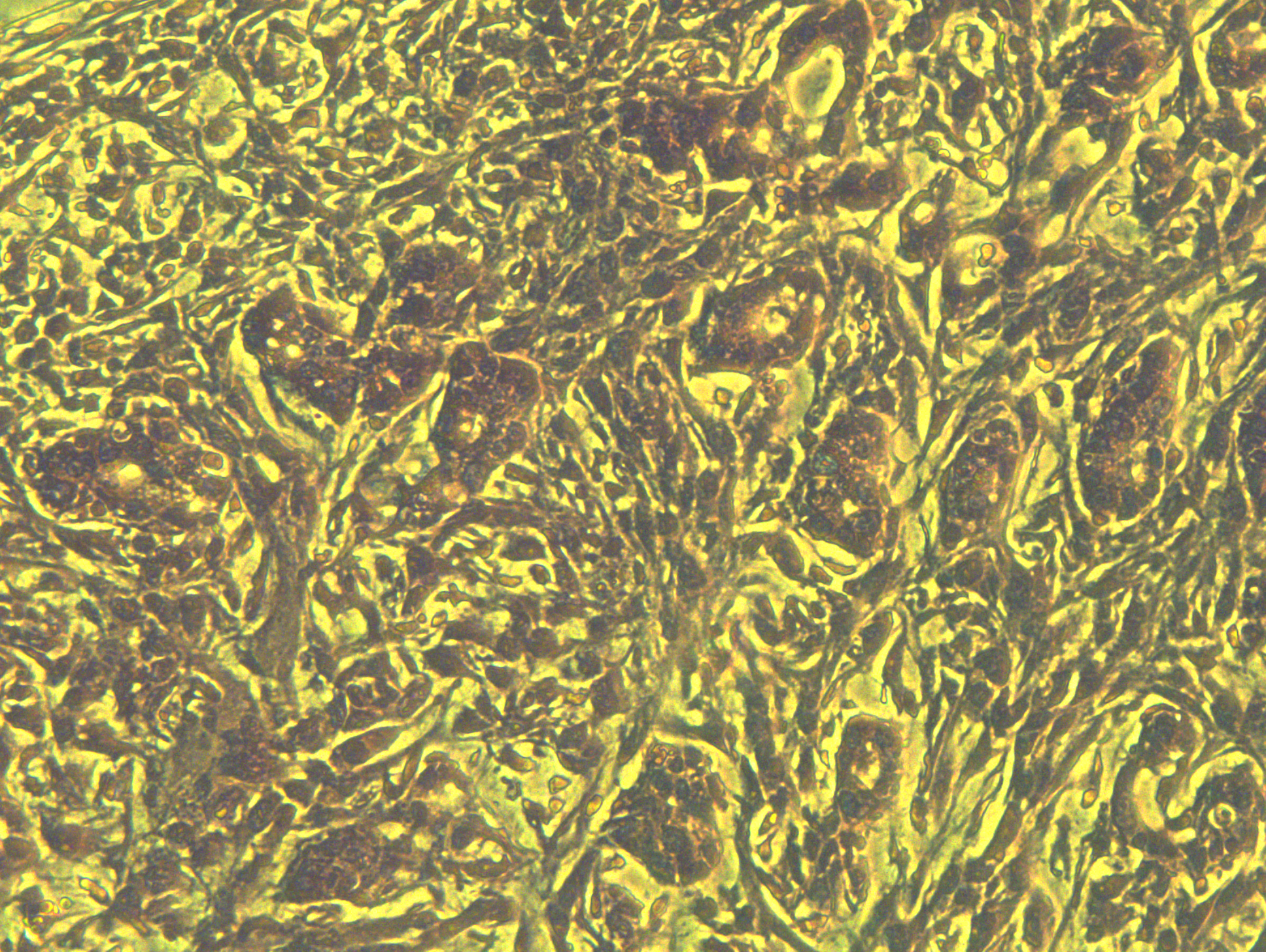

Supplement: Supplementary file 20 — Source Data for Figure 5 [file EMBJ-42-e110902-s022.zip › Figure 5/5G/Control siRNA - KRAS ON.tif]

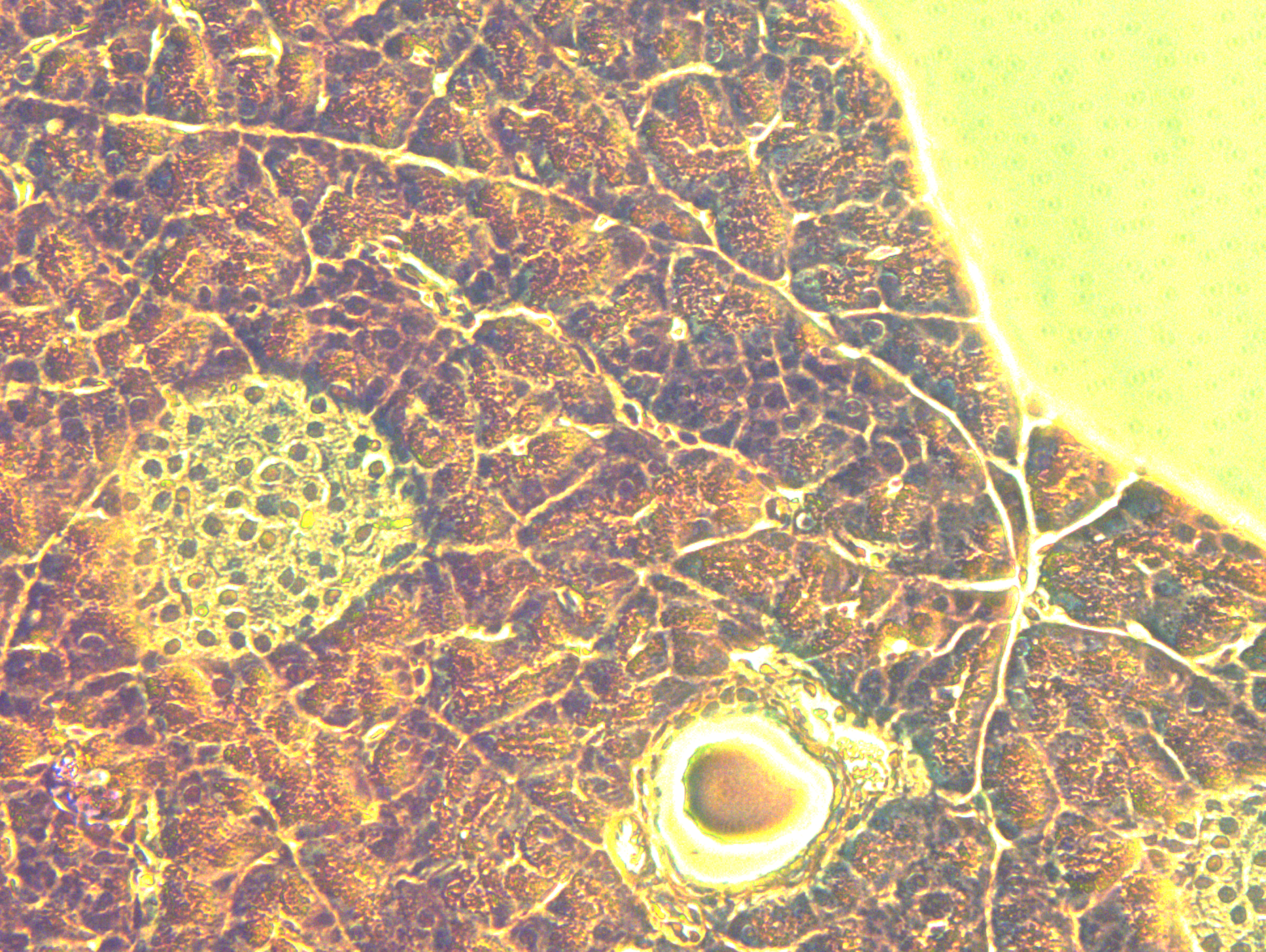

Supplement: Supplementary file 20 — Source Data for Figure 5 [file EMBJ-42-e110902-s022.zip › Figure 5/5G/Ncl siRNA - KRAS OFF.tif]

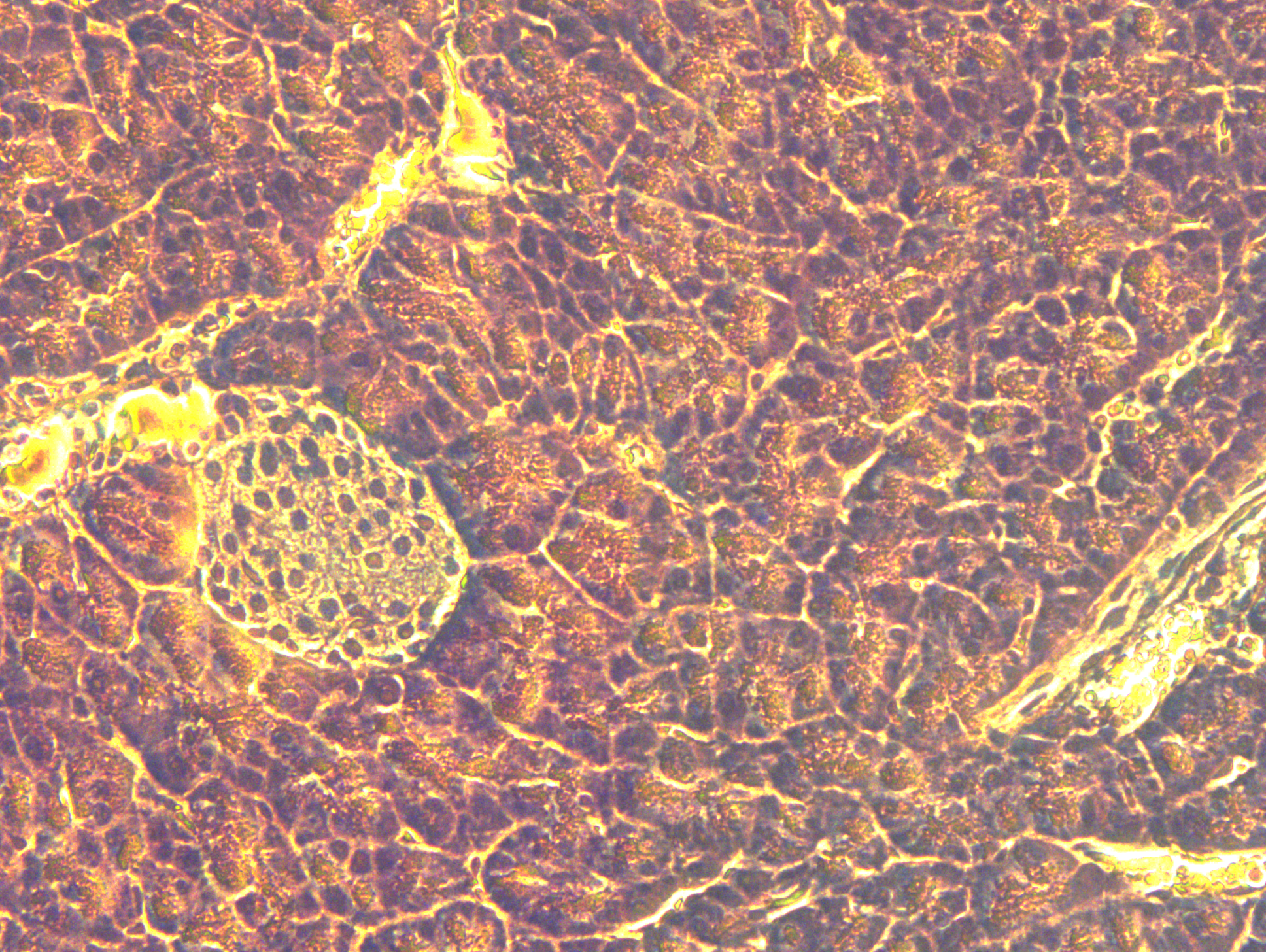

Supplement: Supplementary file 20 — Source Data for Figure 5 [file EMBJ-42-e110902-s022.zip › Figure 5/5G/Ncl siRNA - KRAS ON.tif]

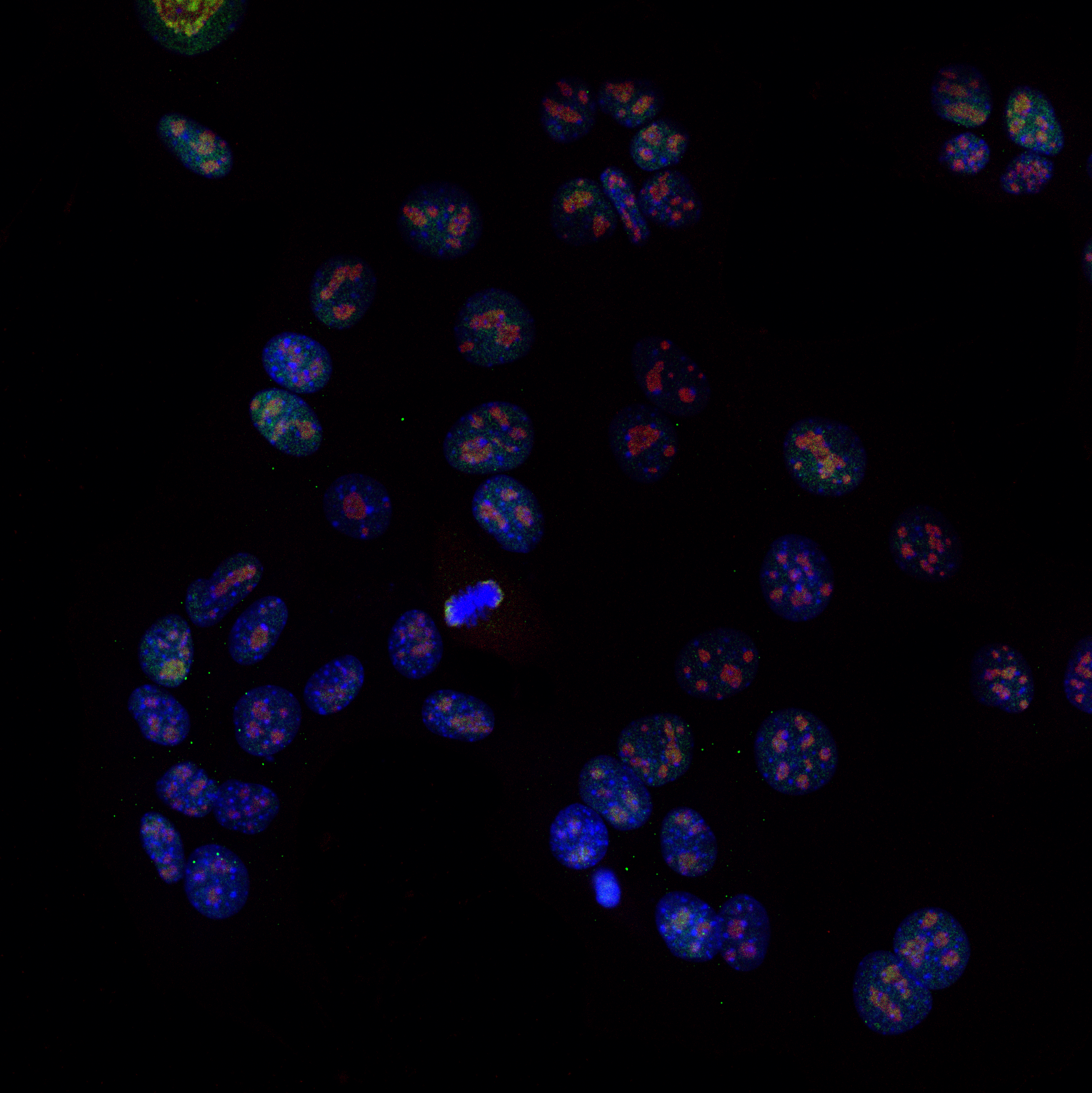

Supplement: Supplementary file 21 — Source Data for Figure 6 [file EMBJ-42-e110902-s018.zip › Figure 6/6A/KRAS OFF_0nM CX5461.tif]

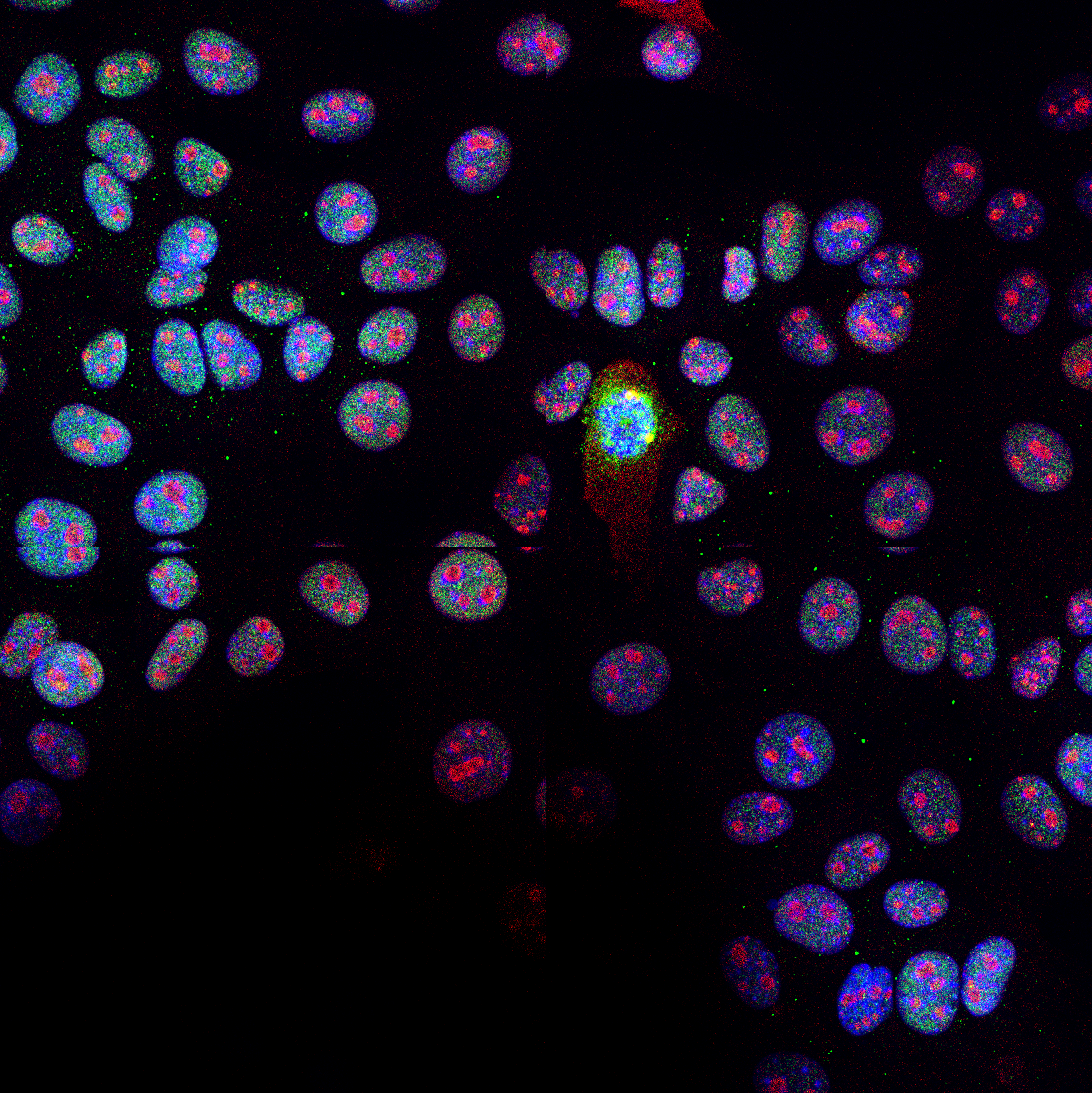

Supplement: Supplementary file 21 — Source Data for Figure 6 [file EMBJ-42-e110902-s018.zip › Figure 6/6A/KRAS OFF_1000nM CX5461.tif]

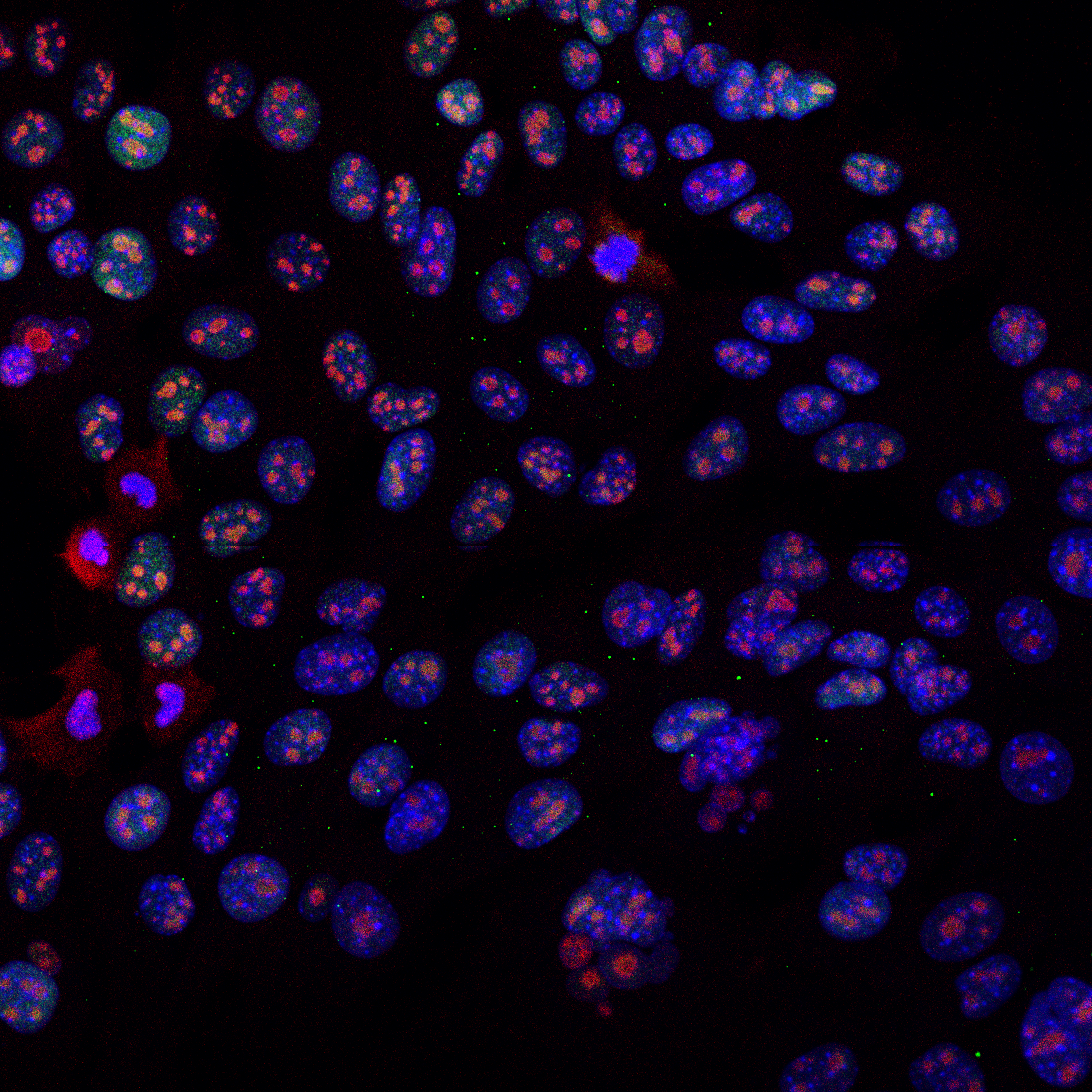

Supplement: Supplementary file 21 — Source Data for Figure 6 [file EMBJ-42-e110902-s018.zip › Figure 6/6A/KRAS OFF_100nM CX5461.tif]

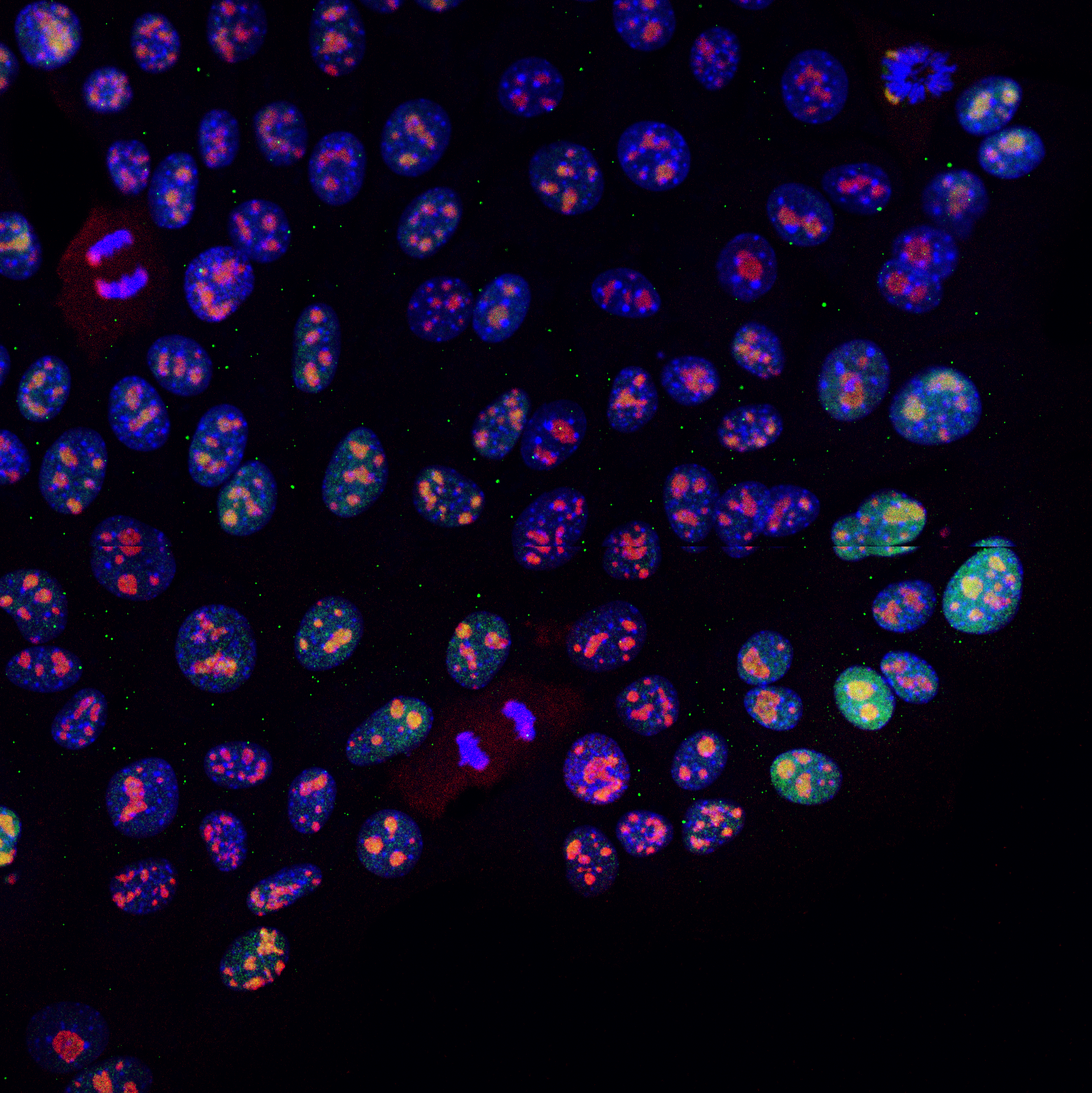

Supplement: Supplementary file 21 — Source Data for Figure 6 [file EMBJ-42-e110902-s018.zip › Figure 6/6A/KRAS OFF_10nM CX5461.tif]

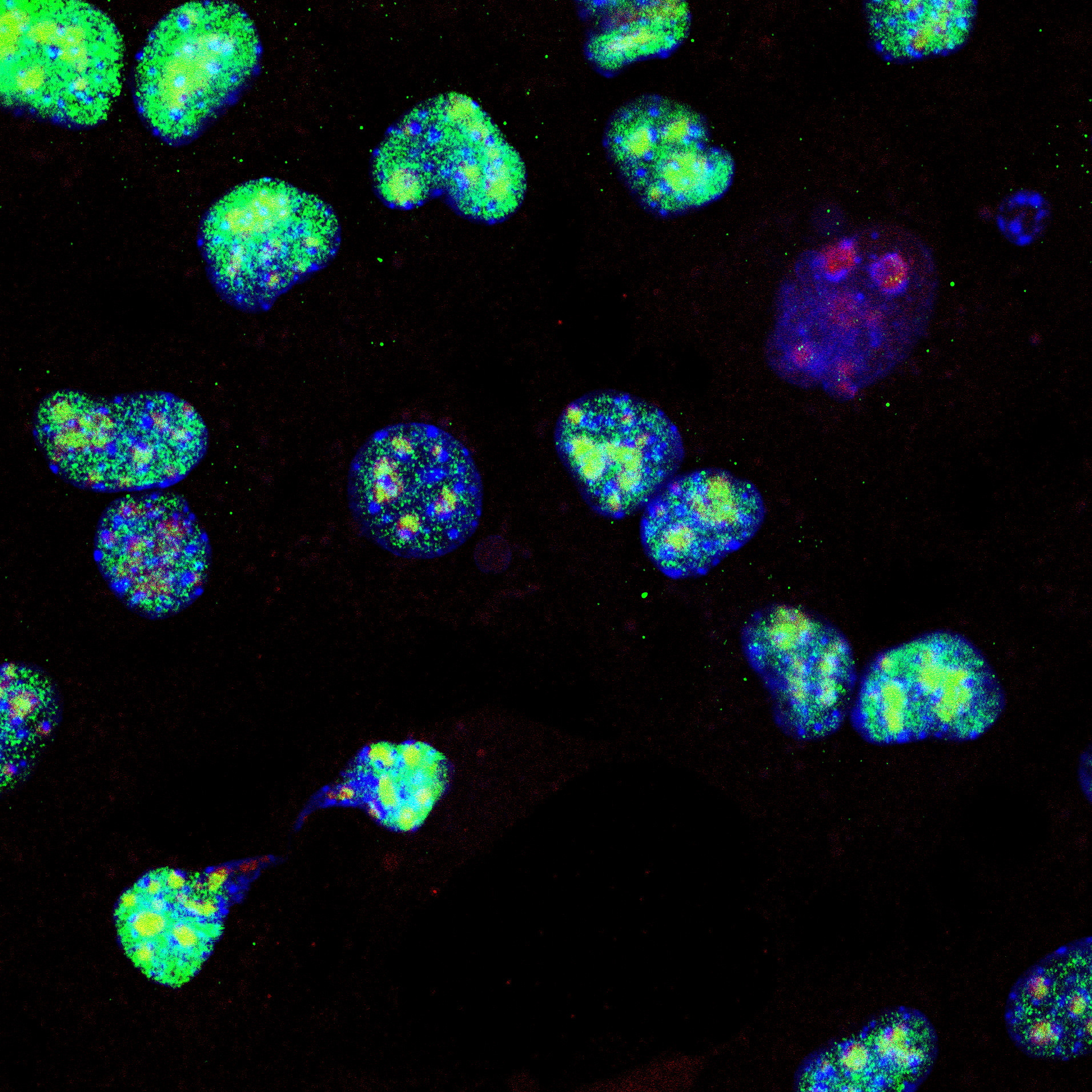

Supplement: Supplementary file 21 — Source Data for Figure 6 [file EMBJ-42-e110902-s018.zip › Figure 6/6A/KRAS ON_0nM CX5461.tif]

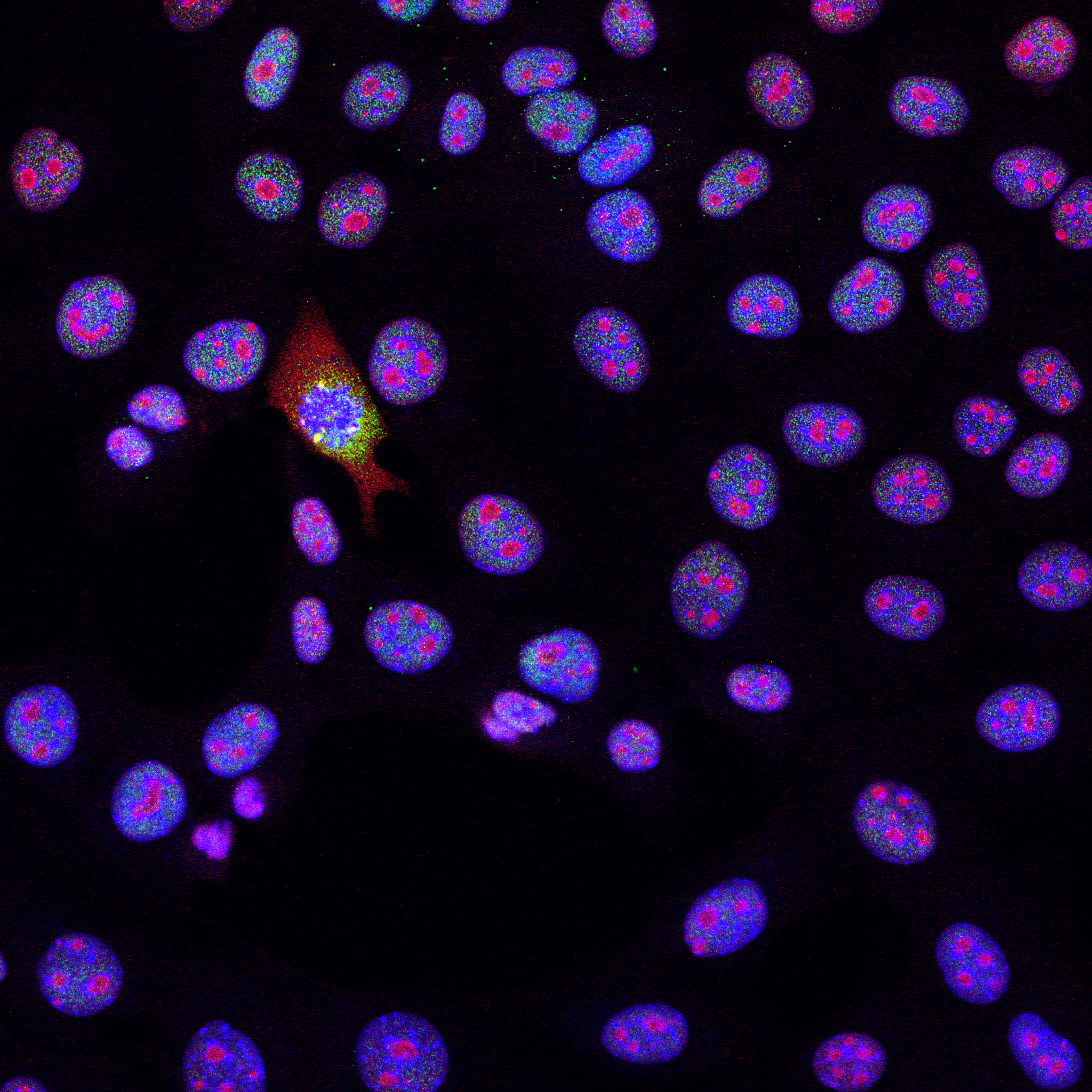

Supplement: Supplementary file 21 — Source Data for Figure 6 [file EMBJ-42-e110902-s018.zip › Figure 6/6A/KRAS ON_1000nM CX5461.tif]

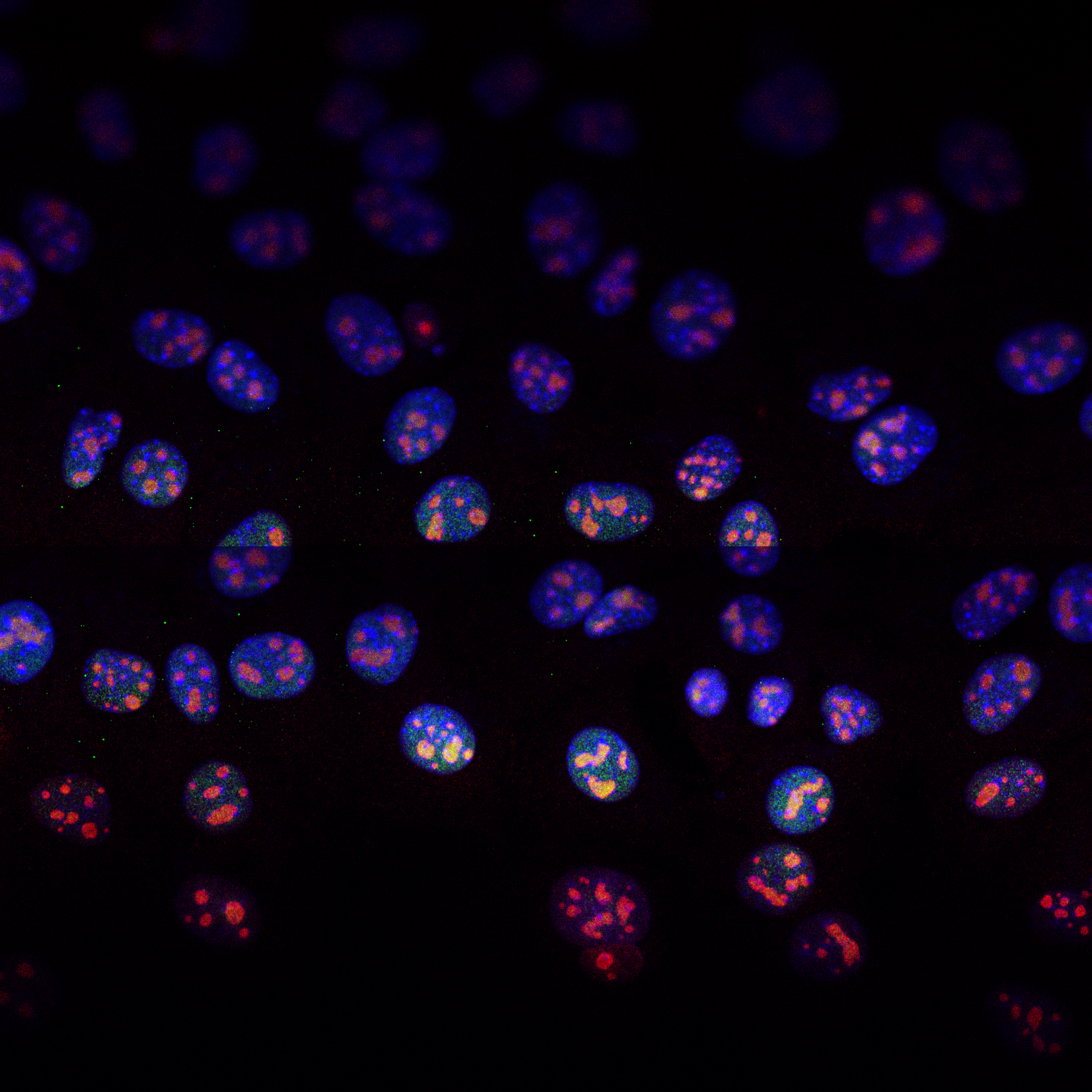

Supplement: Supplementary file 21 — Source Data for Figure 6 [file EMBJ-42-e110902-s018.zip › Figure 6/6A/KRAS ON_100nM CX5461.tif]

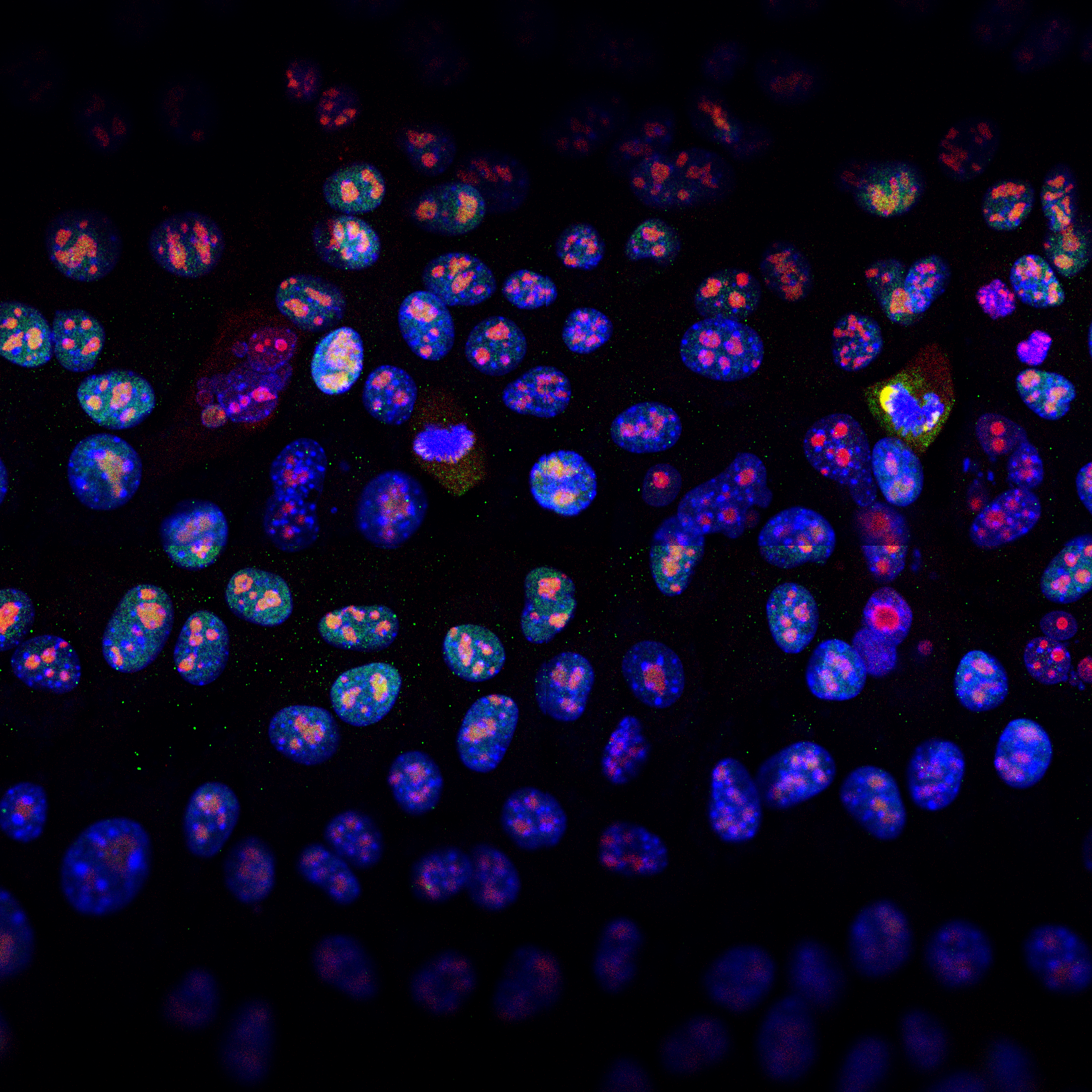

Supplement: Supplementary file 21 — Source Data for Figure 6 [file EMBJ-42-e110902-s018.zip › Figure 6/6A/KRAS ON_10nM CX5461.tif]

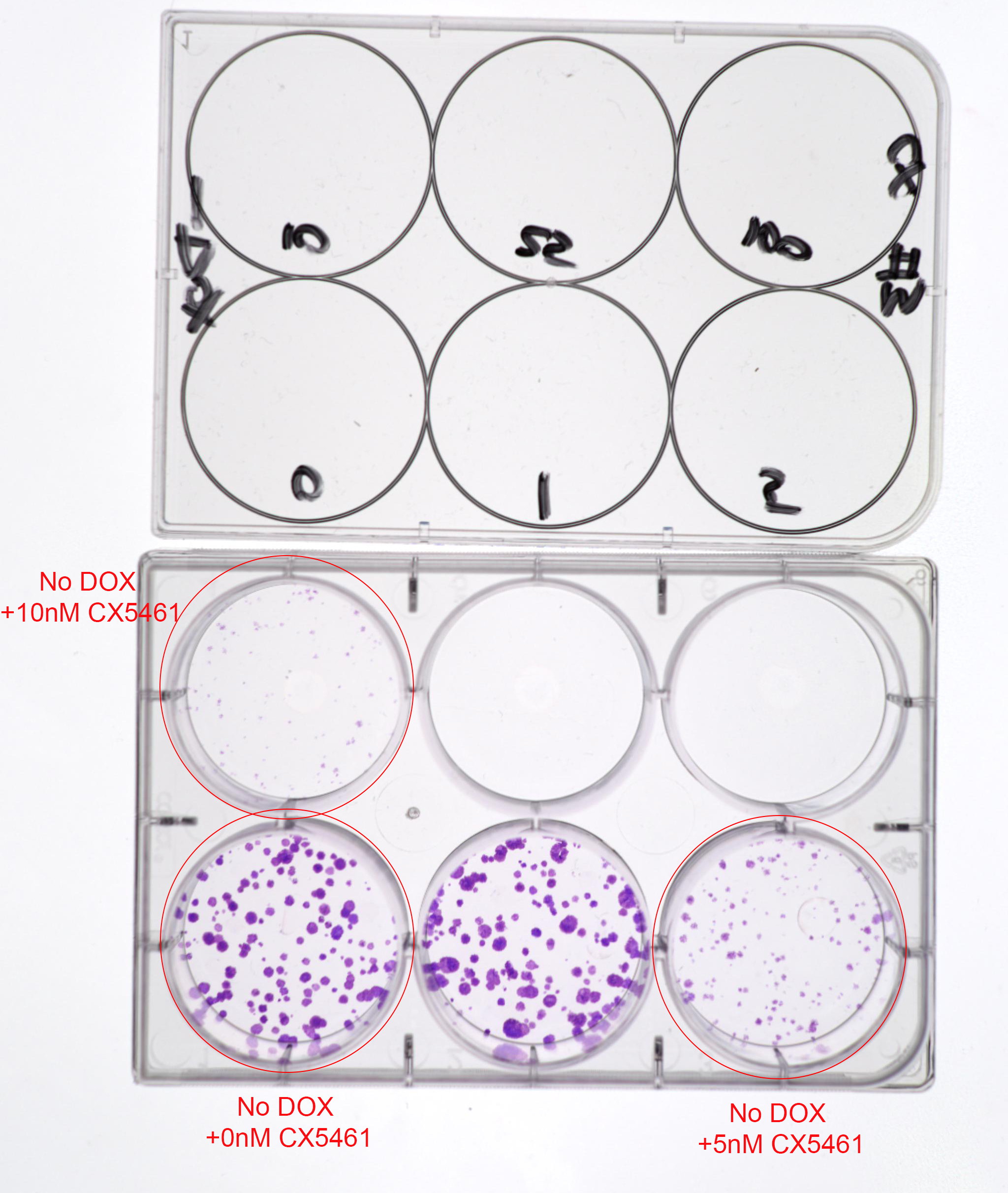

Supplement: Supplementary file 21 — Source Data for Figure 6 [file EMBJ-42-e110902-s018.zip › Figure 6/6D/KRAS OFF_0nM_5nM_10nM CX5461.jpg]

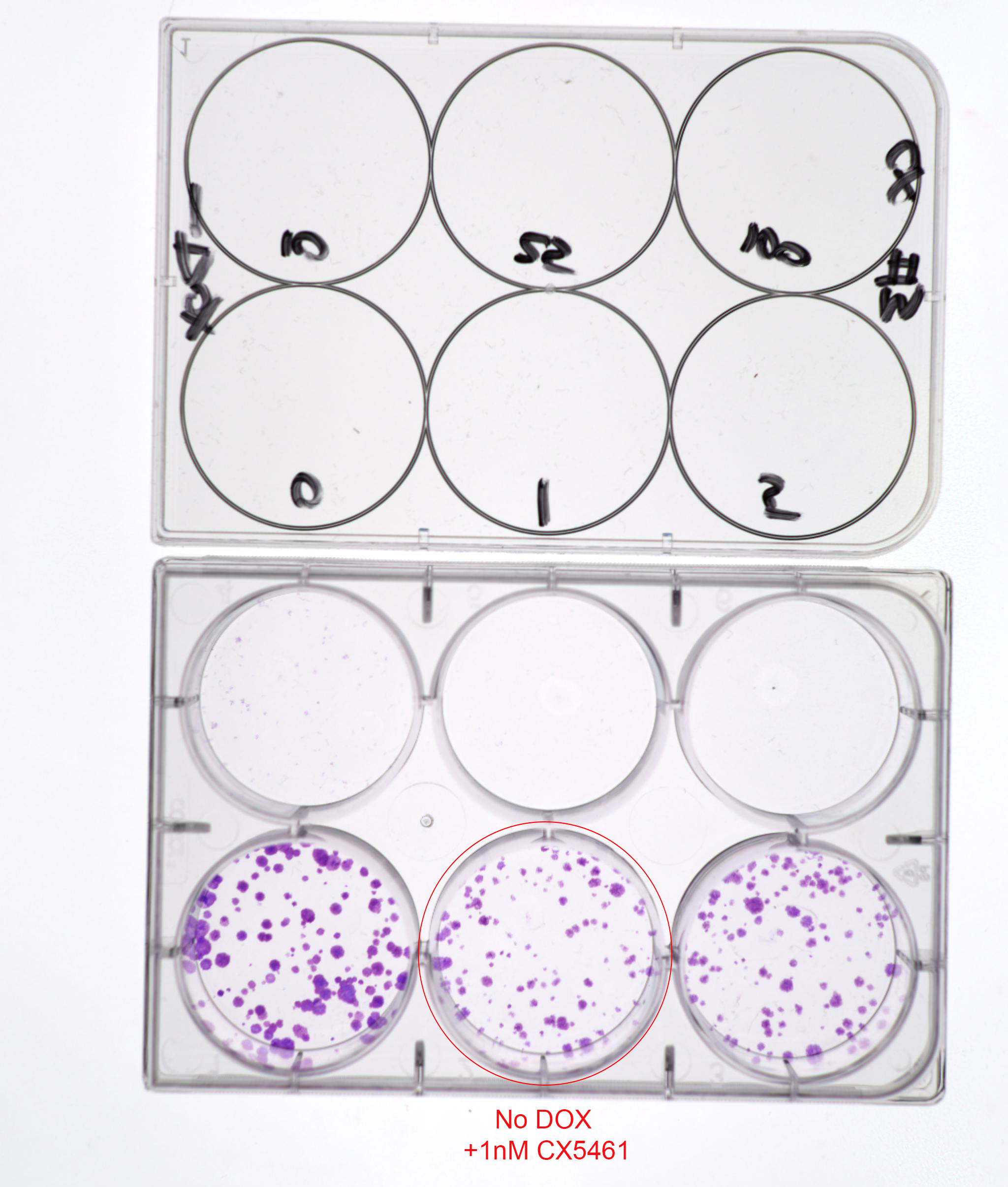

Supplement: Supplementary file 21 — Source Data for Figure 6 [file EMBJ-42-e110902-s018.zip › Figure 6/6D/KRAS OFF_1nM CX5461.jpg]

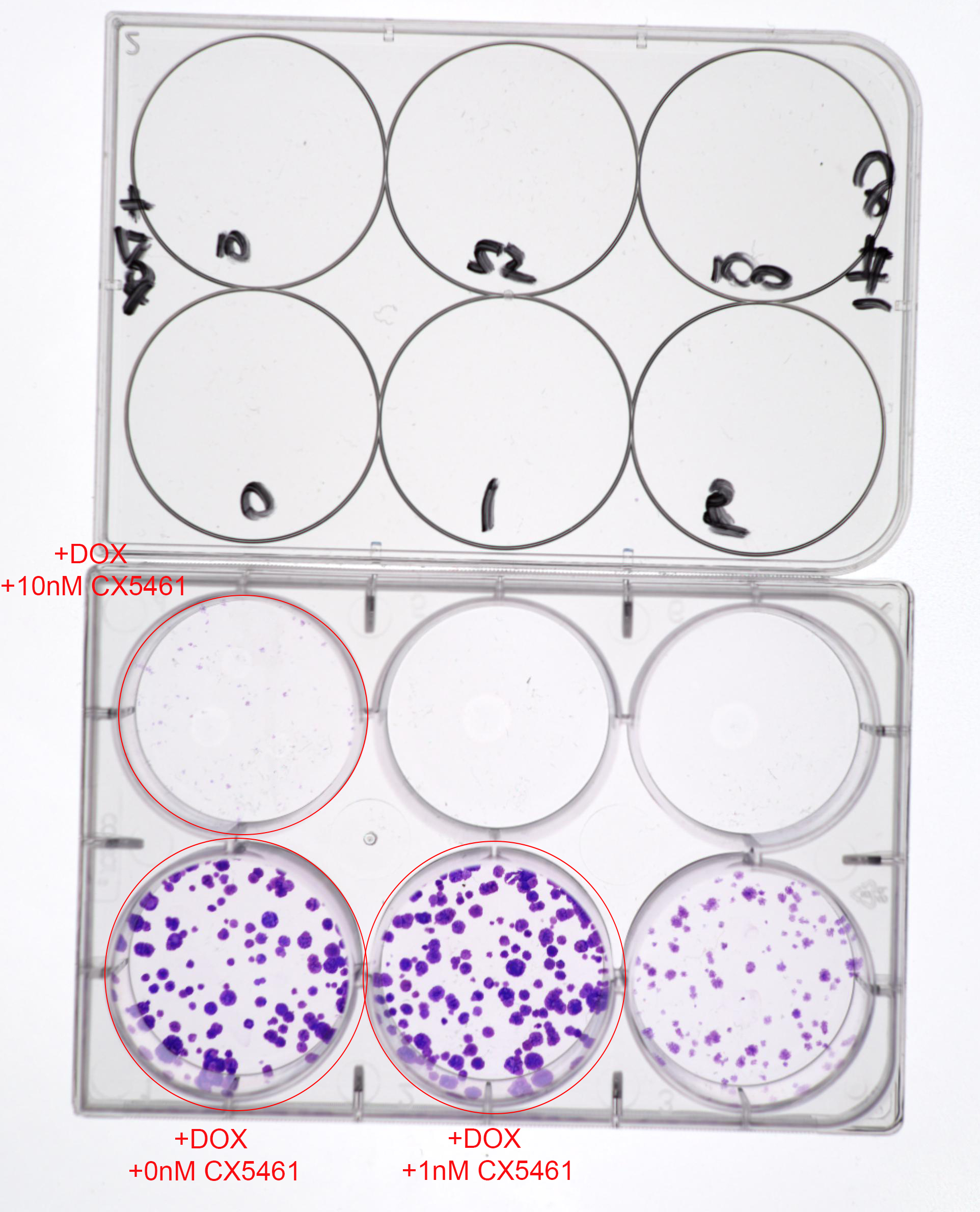

Supplement: Supplementary file 21 — Source Data for Figure 6 [file EMBJ-42-e110902-s018.zip › Figure 6/6D/KRAS ON_0nM_1nM_10nM CX5461.jpg]

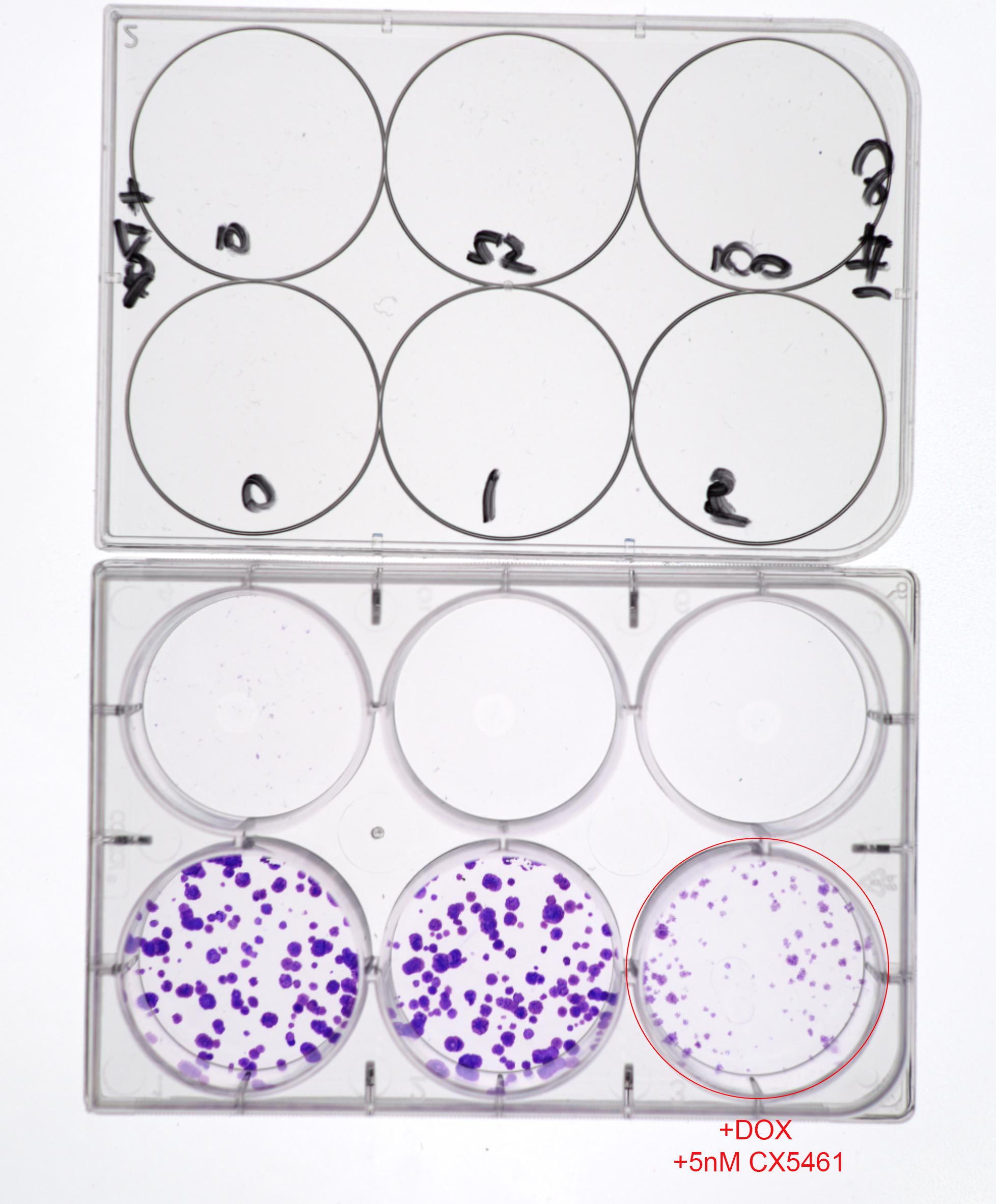

Supplement: Supplementary file 21 — Source Data for Figure 6 [file EMBJ-42-e110902-s018.zip › Figure 6/6D/KRAS ON_5nM CX5461.jpg]

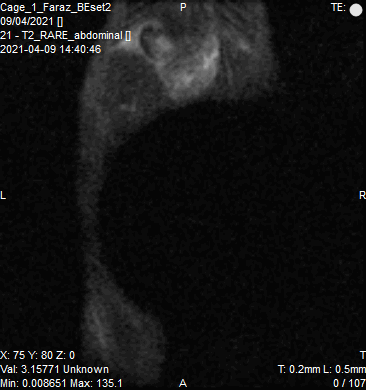

Supplement: Supplementary file 21 — Source Data for Figure 6 [file EMBJ-42-e110902-s018.zip › Figure 6/6G/20210409_CX-5461 treated_day 3_ROI.gif]

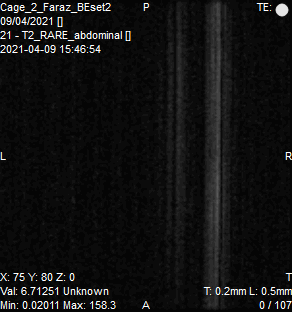

Supplement: Supplementary file 21 — Source Data for Figure 6 [file EMBJ-42-e110902-s018.zip › Figure 6/6G/20210409_Untreated_day 3_ROI.gif]

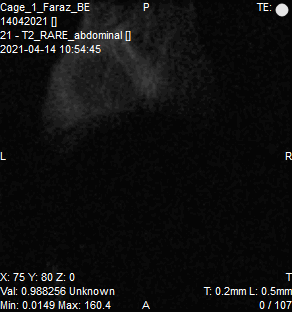

Supplement: Supplementary file 21 — Source Data for Figure 6 [file EMBJ-42-e110902-s018.zip › Figure 6/6G/20210414_CX-5461 treated_day 8_ROI.gif]

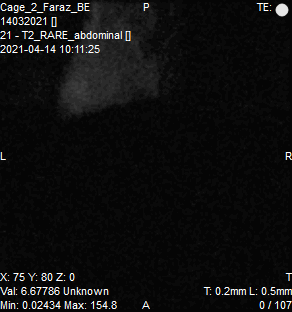

Supplement: Supplementary file 21 — Source Data for Figure 6 [file EMBJ-42-e110902-s018.zip › Figure 6/6G/20210414_Untreated_day 8_ROI.gif]

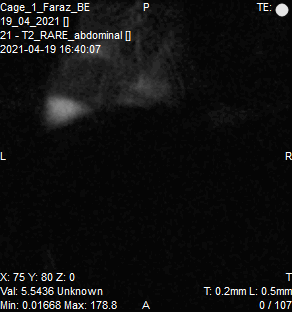

Supplement: Supplementary file 21 — Source Data for Figure 6 [file EMBJ-42-e110902-s018.zip › Figure 6/6G/20210419_CX-5461 treated_day 13_ROI.gif]

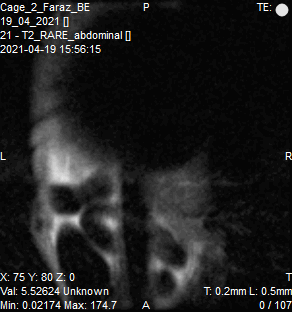

Supplement: Supplementary file 21 — Source Data for Figure 6 [file EMBJ-42-e110902-s018.zip › Figure 6/6G/20210419_Untreated_day 13_ROI.gif]

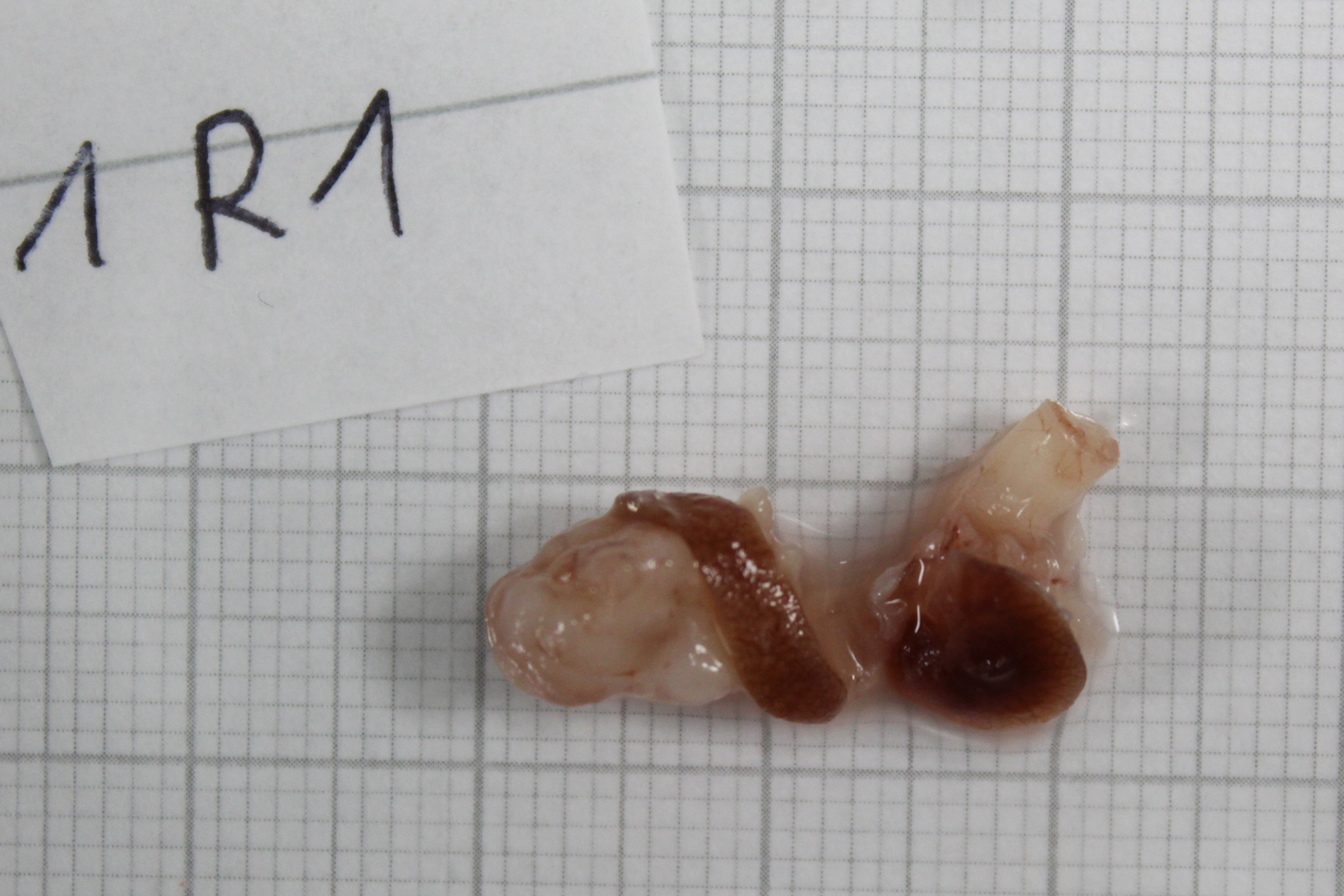

Supplement: Supplementary file 21 — Source Data for Figure 6 [file EMBJ-42-e110902-s018.zip › Figure 6/6I/CX-5461 treated.JPG]

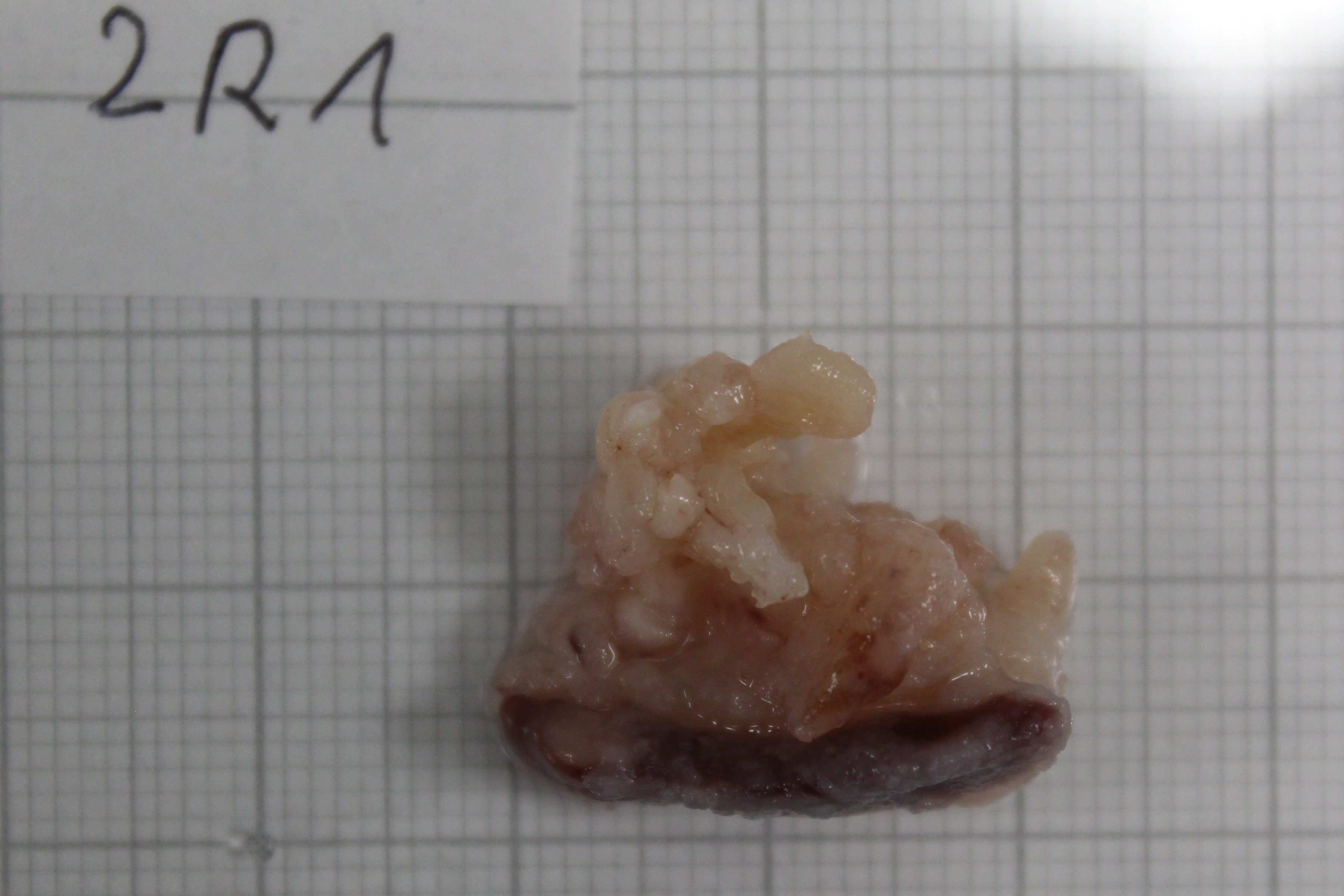

Supplement: Supplementary file 21 — Source Data for Figure 6 [file EMBJ-42-e110902-s018.zip › Figure 6/6I/Untreated.JPG]

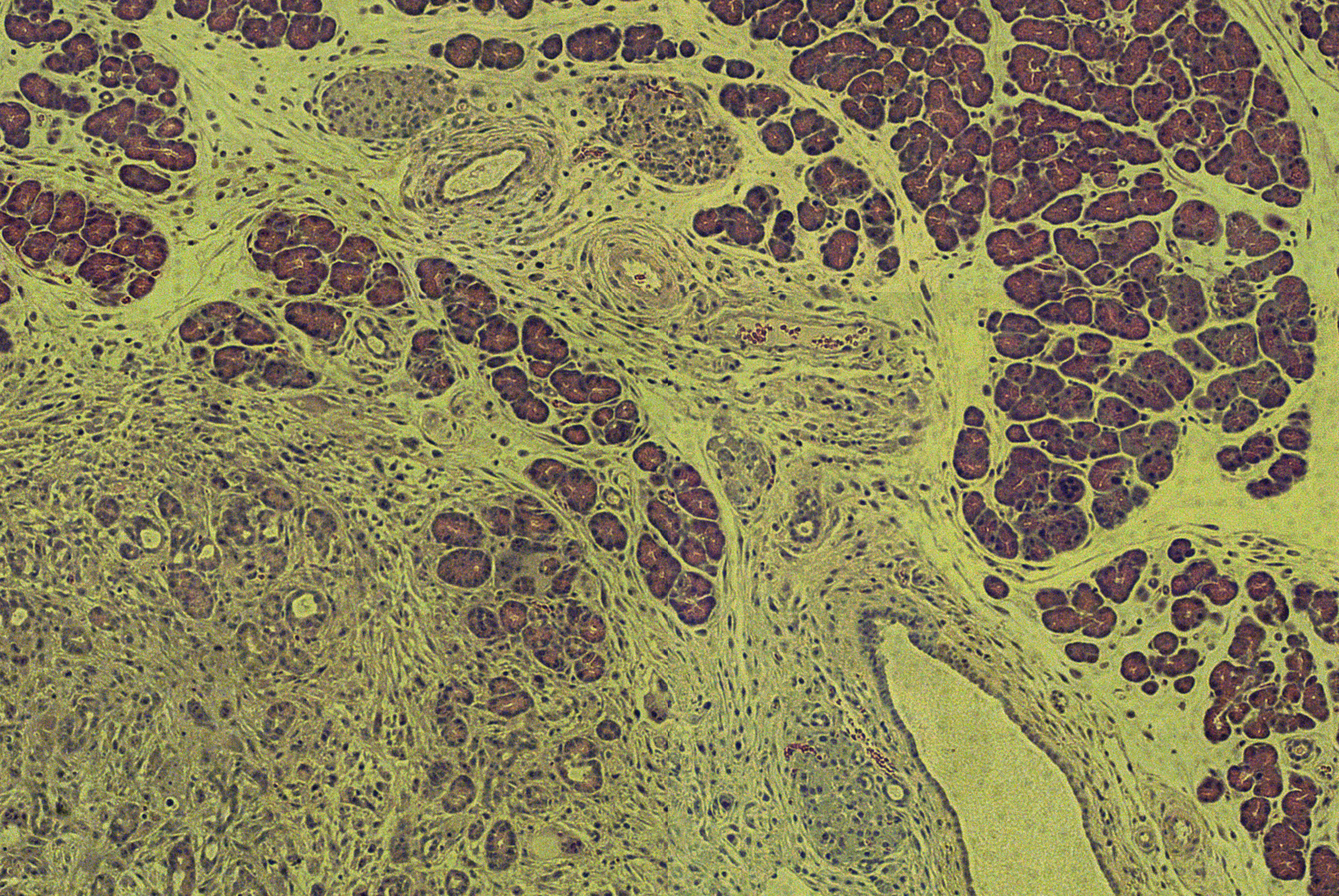

Supplement: Supplementary file 21 — Source Data for Figure 6 [file EMBJ-42-e110902-s018.zip › Figure 6/6K/CX-5461_tiled image.tif]

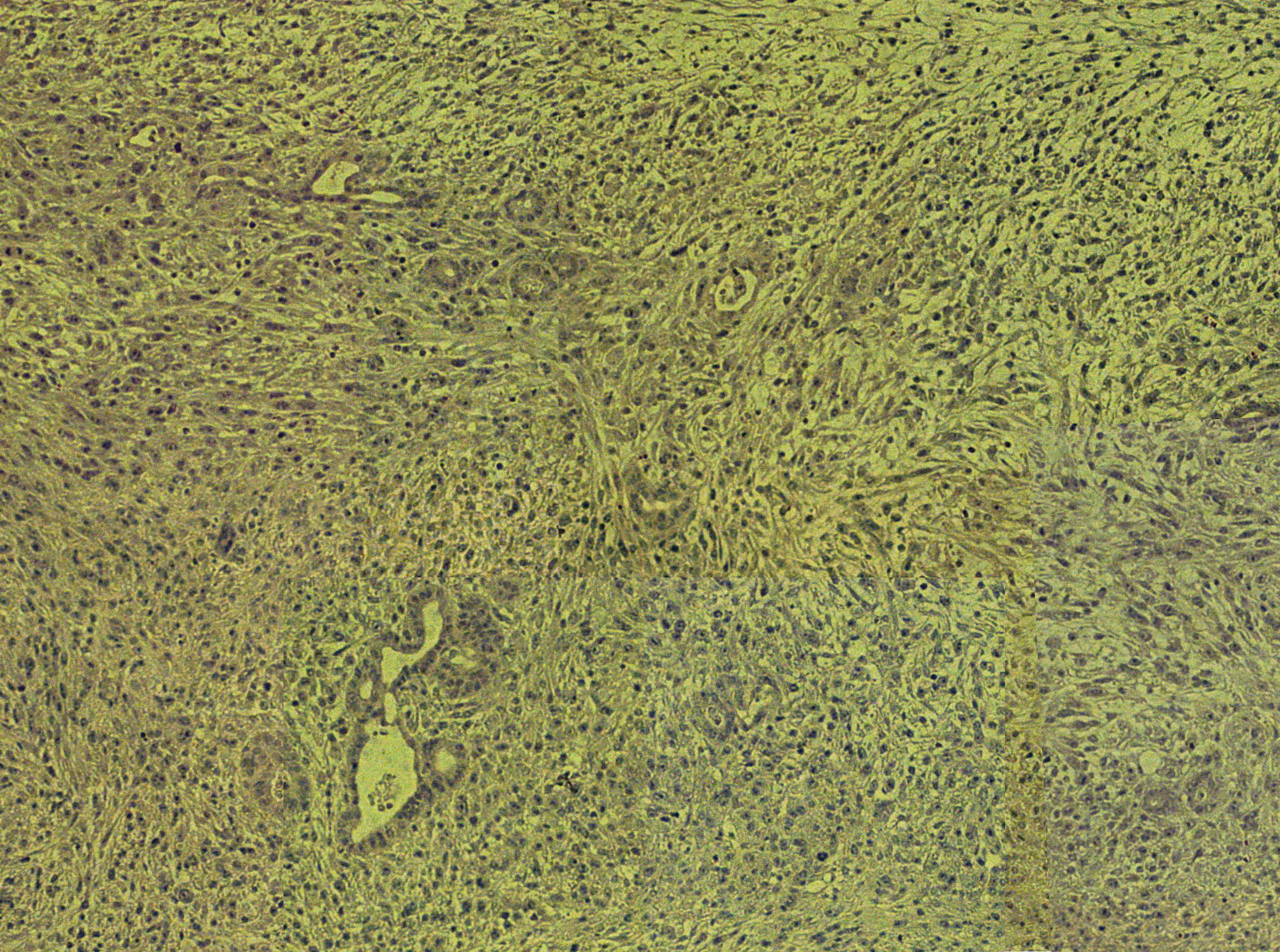

Supplement: Supplementary file 21 — Source Data for Figure 6 [file EMBJ-42-e110902-s018.zip › Figure 6/6K/Untreated_tiled image.tif]
