# Supplementary material for: An ERK1/2‐driven RNA‐binding switch in nucleolin drives ribosome biogenesis and pancreatic tumorigenesis downstream of RAS oncogene
Source: EMBO J. 2023 Apr 11;42(11):e110902. doi: 10.15252/embj.2022110902 (PMC10233377; doi:10.15252/embj.2022110902)
Supplement: Supplementary file 22 [file EMBJ-42-e110902-s008.pdf]

THE EMBO JOURNAL

An ERK1/2-driven RNA-binding switch in nucleolin drives ribosome biogenesis and pancreatic tumorigenesis downstream of RAS oncogene

Muhammad Azman, Emilie Alard, Martin Dodel, Federica Capraro, Rupert Faraway, Maria Dermit, Wanling Fan, Alina Chakraborty, Jernej Ule, and Faraz Mardakheh
DOI: 10.15252/embj.2022110902

Corresponding author: Faraz Mardakheh (f.mardakheh@qmul.ac.uk)

Review Timeline:

Submission Date:	10th Feb 22
Editorial Decision:	29th Mar 22
Revision Received:	1st Dec 22
Editorial Decision:	12th Jan 23
Revision Received:	30th Jan 23
Editorial Decision:	11th Feb 23
Revision Received:	14th Feb 23
Accepted:	12th Mar 23

Editor: Stefanie Boehm / Daniel Klimmeck

Transaction Report:

(Note: With the exception of the correction of typographical or spelling errors that could be a source of ambiguity, letters and reports are not edited. Depending on transfer agreements, referee reports obtained elsewhere may or may not be included in this compilation. Referee reports are anonymous unless the Referee chooses to sign their reports.)

Dr. Faraz K Mardakheh
Barts Cancer Institute, Queen Mary University of London
Centre for Cancer Cell and Molecular Biology
Charterhouse square
London EC1M 6BQ
United Kingdom

28th Mar 2022

Re: EMBOJ-2022-110902
An RNA-binding switch drives ribosome biogenesis and tumorigenesis downstream of RAS oncogene

Dear Dr. Mardakheh,

Thank you for submitting your manuscript for consideration by The EMBO Journal. We have now received two referee reports on your study, which are included below for your information. I have discussed the these reports and the manuscript with other members of the editorial team. However, taking all into consideration, we have decided that we unfortunately cannot offer to invite a revision for EMBO Journal at this time.

As you will see, both reviewers appreciate the proposed model where KRas-G12D and Erk1/2-induced Nucleolin (Ncl) phosphorylation alters rRNA synthesis and ribosome biogenesis. However, at this stage, we overall find that additional experimental data to support this model would be required, as referee #2 in particular points out. Specifically, the phosphorylation of Ncl and its functional effects would need to be assessed in further mechanistic detail (ref #1- point 3, 4; ref #2- point 6, 7, 8, 9). Thus, despite the in general more positive assessment of referee #1, in our view, addressing these major issues regarding the mechanism, as well as the additional concerns on controls and statistics, would likely go beyond the extent of the single round of revision journal policy allows for and potentially include experiments with unknown outcome. Therefore we have concluded that we cannot offer further steps towards publication here at this time.

However as mentioned, we recognize the potential interest of the findings to the field, and would be open to reconsider a revised version of the study if you are able to fully resolve all concerns of the referees in the future. Please however note that in such a case, we again assess novelty and advance at the timepoint of resubmission and may involve alternative or additional referees if needed. Yet, we also realize that this may not be an option, and we encourage you to consider a transfer your study to our not-for-profit open-access sister journal, Life Science Alliance (LSA) for publication of the current manuscript pending a less extensive revision.

We shared your manuscript and the accompanying reviews with LSA Executive Editor, Eric Sawey, who is interested in these findings, and would like to invite further consideration of this manuscript at LSA pending the following revisions: - Address Reviewer 2's Points # 1, 3, 4, 8, 9, 11, 12, 13. We understand that such a revision might need to be re-reviewed, in which case, Dr. Sawey will walk the Reviewers through our transfer process. We encourage you to use the link below to transfer your manuscript to LSA. You do not need to revise the manuscript before transferring it to LSA. Once you transfer, Dr. Sawey will email you an invitation to revise and resubmit, listing the same revision requests as mentioned above. Please feel free to reach out at e.sawey@life-science-alliance.org if you have any questions about the LSA journal, the transfer process or the revisions requested.

I am sorry that I cannot be more positive on this occasion, but hope that you will nonetheless find the comments of our reviewers helpful and consider the option of a transfer. Thank you again for giving us the opportunity to consider your manuscript.

Kind regards,

Stefanie Boehm

Stefanie Boehm
Editor
The EMBO Journal

****

Referee #1:

This is an interesting manuscript that unravels a new function for Ras oncogenic activity in reprograming the RNA interactome during tumor development. Specifically, the authors employed several cutting-edge techniques such as Orthogonal Organic Phase Separation (OOPS) and quantitative RNA Interactome Capture (qRIC) to show a significant increase in the RNA-bound levels of 73 proteins. The authors further showed that Erk1/2 signaling downstream of Ras is responsible to phosphorylate the Nucleolin protein (Ncl), an event that is important to augment the interaction between Ncl and rRNA precursors. Ncl phosphorylation also enhances rRNA synthesis and ribosome biogenesis. Importantly the authors presented very interesting data on the role of Ncl in supporting pancreatic cell proliferation and tumor development. Finally the authors showed that pharmacological inhibition of ribosome biogenesis, by employing the CX-5461 compound, functionally mimics the Ncl decrease. This is a comprehensive and important work that makes a significant contribution to the field by unveiling new mechanisms by which oncogenic Ras directs a post-transcriptional circuitry to sustain one of the deadliest cancers. I have small comments that may help to strengthen the conclusions presented in this manuscript.

-The authors should test if the Ncl mutants behave differently when overexpressed in cells where the endogenous Ncl has been depleted. In this scenario, the authors may observe different results in the experiments presented in Fig3. At least they should comment that this is a possible caveat of why they don't observe any difference in rRNA binding between Wt and Ncl mutants.

-It would be interesting to assess whether the Ras-Ncl axis also increases protein synthesis in cells or in vivo.

-The authors should investigate the phosphorylation status of Ncl during tumor development and whether Ncl phospo mutants impact tumor growth or at least cancer cell growth.

-The authors should also discuss/speculate how downstream of the Ras-Ncl axis the translation of specific mRNA networks may impact tumor development.

Referee #2:

In this original article, the authors set out to address the changes in RNA binding proteins upon modulation of KRAS activity in a model of PDAC. Importantly, the manuscript addresses the molecular mechanisms as well as the therapeutic implications of their findings.
Overall, the study is very well structured and the reasoning behind the experiments flows well. However, we find that there are some major issues and relatively limited novelty. The target protein the authors focus on, Nucleolin, is a known RNA binding protein, with known function in regulating ribosomal RNAs transcription, biogenesis, and general ribosomal biogenesis. All these processes are essential for tumour growth.
The novelty would lie in the mechanism proposed: that KRAS activates ribosomal biogenesis increasing Nucleolin RNA binding activity, and that this happens via CK2. However, the manuscript is not structured around this, the experiments are not strong enough to support the claims and causality is not properly explored. Therefore, I would recommend to reject the article, while still providing some suggestions to the authors to strengthen their claims for another journal.

1. The title should clearly indicate that the described mechanism is in pancreatic cancer.

2. It seems that the authors want to bring importance to the fact that downstream KRAS activation there is a change in RNA binding activity, rather than RBPs abundance. Therefore, it is suggest to move into supplementary the panels 1B to 1F, which will help to bring more clarity to the story.

3. All of the proteomics data is plotted in figures showing -log10 p-values and t-test differences. In the figure legends, sometimes thresholds are indicated with p-values, sometimes with FDR. The method sections does not describe any p-value adjustments done downstream of conditions comparisons - the only FDR described seems to be applying to the peptide mapping. However, a high throughput proteomic output would definitely require a p-value adjusting.
At present, it is unclear whether adjustments have not been done, or if the labelling of the axes on graphs Fig 1B, 1E, 1G, 2A, 2C requires correcting. In either cases, please correct.

In terms of thresholds being used, it is unclear for figure 2A and 2C why they are different in the figures (black lines) but in the legend they are mentioned to be the same FDR 0.05. On dataset S8, the FDR is not provided. If one uses the same p-value and difference thresholds in the two conditions (-/+ Trametinib), there are positions differentially phosphorylated upon KRAS induction even in presence of Trametinib.

Regarding adjusted p-values and FDRs for the proteomics datasets in the excel supplementary file, they should all be included - at present the information is given as -log10- p-value (S1). The rest of the datasets mention -log P value (please add the base of the -log, I can assume 10, but would be good to have it) this applies to S4, S5, S8. Additionally please consider using -log10 p value also for the CX-5461 enrichment analyses - at present it is being given as p-value.

4. A lot of the emphasis is put on how inhibition of MAPK signalling arm abrogates the RNA binding activity changes and/or changes in the RBPome downstream KRAS activation. However, fig 1E and 2C (once the same threshold as 2A is applied) show that there are still differences present when cells are treated with Trametinib. One or two sentences could be spent in the discussion to mention that the PI3K arm of signalling downstream KRAS might also be playing a role in regulating some functions of the RBPome.

5. In the Phospho-motif analysis in fig S2, together with ERK1, also DUPS1 is highly enriched. What is potentially the explanation that DUSP1 motives are increased in sites which undergo enhanced phosphorylation?

6. A validation of Nucleolin phosphorylation downstream of ERK is required. If no antibody is available, an approach such as Phos Tag gel could be employed. These experiments should ideally be done in conditions which also support the fact that the phosphorylation is mediated by CK2.

7. pCK2 substrate blots and CK2 activity downstream ERK1/2. The article you cite describes CK2 activation via ERK signalling via a specific phosphorylation (ERK2 docking groove and phosphorylates CK2α primarily at T360/S362). We were wondering if the phosphorylation is present also in your own dataset or perhaps other CK2 phosphorylations are present.
Are total levels of CK2 changing? Total CK2 should be blotted for the samples shown in Fig S2 B and D to help clarify the mechanism.

8. Considering the centrality of CK2 in the mechanism that regulates Nucleolin activity, some space in the discussion should be devoted to it. How is CK2 regulated? Is it possible that what gets regulated downstream ERK is a phosphatase rather than a kinase?

9. Figure 2E and F seem to us problematic. First of all it is not clear if this analysis was done in a condition of KRAS ON or OFF - this might affect our comment in 9.c.

a. You are extracting from the same sample the interphase and the organic phase. The total amount of a protein in your sample of origin is a sum of the two. Therefore, the total amount of Ncl S4D is higher than the others.
This should be tackled by doing a blot which has an input portion, the interphase, and the organic phase all together. Additionally, the blot of endogenous Nucleolin in input, interphase, and organic phase in KRAS ON and KRAS OFF conditions would further support this mechanism.

b. It is highly unclear as to why in the interphase there are two bands, and only one in the organic.

c. This figure is the central one in proving your novel mechanism: to prove that phosphorylated Ncl increases its RNA binding capabilities. In fig 4G you show that expression of S4D Ncl in absence of KRAS activation increases unprocessed pre rRNA. Likewise, in figure 4C, with KRAS on there is more unprocessed pre-rRNA. These data both point at the fact that what happens is not that Ncl RNA binding activity is increased, but at the fact that there is simply more pre-rRNA and therefore Ncl binds "more" to it. But the real regulation happens at the RNA expression level, to which Ncl binding is just a consequence.
To tackle this issue, I think you could take advantage of the tagged constructs you have already generated. An IP of the three Ncl versions using equal RNA input material, rather than equal protein input would help to clarify if there is actually an increase in binding activity regardless of how much RNA is present.

10. Ncl KD tends to have an effect across all the phenotypes and molecular mechanisms tested both in KRAS ON and KRAS OFF conditions (eg comparing KRAS OFF Ctrl vs the two siRNAs rather than KRAS ON vs KRAS OFF). The only exception to this seems to be in vivo (Fig 5E and 5F)

11. Figures 6A, 6B, 6C, S6A, and S6B are addressing the responses of cells to CX-5461 - however these experiments have all been performed only in KRAS ON conditions. Please do consider that KRAS OFF cells are suffering heavily from CX-5461 treatment, possibly more than KRAS ON, at the lowest concentration. Therefore, it would be important to show the same effects are present (for rRNA/nucleolar phenotypes) or absent (DNA damage) also in KRAS OFF conditions.

12. Dataset S12 is an enrichment based on proteomics data not shown. Ideally, as for all the other figures you have shown with treatments, the dataset should be included in the supplementary tables. This is particularly important as you have generated a heatmap on that data (Fig 6F).

13. Minor points:

a. Figure S6: please check legend and panel numbering, they are not matching
b. Please consider using a different colour scheme for the WT/S4A/S4D data as red and blue are already being heavily used by KRAS ON and OFF and it can get confusing. In particular since you have used blue for KRAS ON(which should correspond to phosphorylated Ncl) and then again used it for the phospho-defective Ncl.

*** As a service to authors, The EMBO Journal offers the possibility to directly transfer declined manuscripts to another EMBO Press title (EMBO Reports, EMBO Molecular Medicine, Molecular Systems Biology) or to the open access journal Life Science Alliance launched in partnership between EMBO Press, Rockefeller University Press and Cold Spring Harbor Laboratory Press. The full manuscript (including reviewer comments, where applicable and if chosen) will be automatically forwarded to the receiving journal, to allow for fast handling and a prompt decision on your manuscript. For more details of this service, and to transfer your manuscript to another EMBO title please follow this link:
Link Not Available

We would like to thank both reviewers for their insightful and constructive comments, which have strengthened and improved our manuscript. Here we have provided a comprehensive point-by-point response to their concerns and suggestions. In addition, to assist with the inspection of our revised manuscript, we have marked the changes made to the original version in red (see revised manuscript).

Referee #1:

This is an interesting manuscript that unravels a new function for Ras oncogenic activity in reprograming the RNA interactome during tumor development. Specifically, the authors employed several cutting-edge techniques such as Orthogonal Organic Phase Separation (OOPS) and quantitative RNA Interactome Capture (qRIC) to show a significant increase in the RNA-bound levels of 73 proteins. The authors further showed that Erk1/2 signaling downstream of Ras is responsible to phosphorylate the Nucleolin protein (Ncl), an event that is important to augment the interaction between Ncl and rRNA precursors. Ncl phosphorylation also enhances rRNA synthesis and ribosome biogenesis. Importantly the authors presented very interesting data on the role of Ncl in supporting pancreatic cell proliferation and tumor development. Finally the authors showed that pharmacological inhibition of ribosome biogenesis, by employing the CX-5461 compound, functionally mimics the Ncl decrease. This is a comprehensive and important work that makes a significant contribution to the field by unveiling new mechanisms by which oncogenic Ras directs a post-transcriptional circuitry to sustain one of the deadliest cancers. I have small comments that may help to strengthen the conclusions presented in this manuscript.
>We are very glad to hear that the reviewer finds our work comprehensive and important. We have done our best to take on board their suggestions (see below).

-The authors should test if the Ncl mutants behave differently when overexpressed in cells where the endogenous Ncl has been depleted. In this scenario, the authors may observe different results in the experiments presented in Fig3. At least they should comment that this is a possible caveat of why they don't observe any difference in rRNA binding between Wt and Ncl mutants.
> Thank you for this suggestion. We now discuss this point in our revised discussion section (see revised manuscript lines 745-763). Specifically, we discuss that the presence of endogenous Ncl may be masking some rRNA binding sites and thereby making them less accessible to our ectopic myc-tagged Ncl proteins. This means that some of the Ncl binding sites may have not been revealed by our approach, and it is possible that a portion of such sites could be differentially impacted by phosphorylation. Having said that, since we performed in-depth sequencing of our iCLIP libraries (4-8 million reads per individual sample – see Figure S3C – as opposed to 1-2 million reads which is commonplace in most iCLIP studies), we expect the likelihood of completely missing some binding sites to be low. Especially as our ectopic Ncl variants are expressed at ~50% of the endogenous level (see Figure S3A & S3B), it is not highly likely that they can be completely outcompeted. Nonetheless, since we can’t rule out this possibility, it is definitely sensible to discuss it.

-It would be interesting to assess whether the Ras-Ncl axis also increases protein synthesis in cells or in vivo.
> Again, a very good suggestion. We have now added new data showing that KrasG12D does indeed enhance overall protein synthesis rates in iKras PDAC cells, and this enhancement is completely dependent on Ncl (See revised Figure 4E and 4F). Thus, the impact on translation mimics that of ribosome biogenesis.

-The authors should investigate the phosphorylation status of Ncl during tumor development and whether Ncl phospo mutants impact tumor growth or at least cancer cell growth.

> Since no phospho-specific antibodies against the 4 sites which we have identified are available, assessment of the phosphorylation change in tumour sections is not currently possible (although we have plans of raising antibodies against these sites in future). To assess the impact of Ncl phospho-mutants on tumour growth, we attempted to generate iKras PDAC cells stably expressing WT or the different phospho-mutants of Ncl, which would be suitable for orthotopic injection into the pancreas. However, despite numerous attempts, we failed to obtain stable cell-lines that maintained the expression of the phospho-defective (S4A) mutant. To us, this suggests that long-term expression of the phospho-defective mutant is likely having a negative impact on the proliferation/viability iKras cells.

We could, however, assess the impact of transiently overexpressing the different phospho-mutants on iKras cell proliferation (See revised Figure S5E). As it can be seen in these results, transient expression of WT and S4D mutant, but not the S4A mutant, significantly boosted tumour cell proliferation, *in vitro*. Interestingly, this effect was much more pronounced when oncogenic KRAS was present, in line with our results from revised Figure S4I, in which we showed that overexpression of WT or the phospho-mimicking mutant of Ncl alone cannot fully rescue ribosome biogenesis, highlighting the existence of other RAS-dependent processes that are important for ribosome biogenesis (see revised manuscript lines 488-490).

-The authors should also discuss/speculate how downstream of the Ras-Ncl axis the translation of specific mRNA networks may impact tumor development.
> Great suggestion! We have now added a few lines to our discussion on this subject (see revised manuscript lines 798-802).

Referee #2:

In this original article, the authors set out to address the changes in RNA binding proteins upon modulation of KRAS activity in a model of PDAC. Importantly, the manuscript addresses the molecular mechanisms as well as the therapeutic implications of their findings.

Overall, the study is very well structured and the reasoning behind the experiments flows well. However, we find that there are some major issues and relatively limited novelty. The target protein the authors focus on, Nucleolin, is a known RNA binding protein, with known function in regulating ribosomal RNAs transcription, biogenesis, and general ribosomal biogenesis. All these processes are essential for tumour growth.

The novelty would lie in the mechanism proposed: that KRAS activates ribosomal biogenesis increasing Nucleolin RNA binding activity, and that this happens via CK2.
> While it is true that our proposed mechanism of Ncl regulation downstream of RAS is one of the key novel aspects of our study, we disagree that this is our only major novel finding. We strongly believe that one of our major novel discoveries is revealing that the expression and activity of several RBPs are regulated by oncogenic RAS signalling. Traditionally, most oncogenic signalling pathways (e.g. RAS-MAPK) have been considered to primarily to control gene expression transcriptionally, through regulating the activity of different transcription factors (e.g. MYC, JUN, FOS, and ELK, in case of RAS-MAPK signalling). Our unbiased quantitative RNA Interactome Capture (qRIC) analysis, in an inducible KRAS cancer model, clearly shows that RAS-MAPK signalling also modulates the RBPome, thus demonstrating a key novel layer of regulation at the post-transcriptional level (Rebuttal Figure 1). To our knowledge, this is the first example of a qRIC study in the context of oncogenic signalling, and therefore constitutes a major original finding. In addition, we show that for some RBPs, their RNA

binding activity, and not expression levels, is regulated by RAS. This is another novel aspect of our study. Our phospho-proteomics analysis provides evidence that the regulation of several activated RBPs maybe achieved via phosphorylation. Through use of phospho-mutants, we prove this for Ncl, which was chosen for further in focus analysis because it was the most strongly phosphorylated RBP downstream of KRAS (Figure 2A), and is a well-known cancer-relevant RBP that has been previously implicated in various cancers including PDAC *(Abdelmohsen & Gorospe, 2012; Gilles et al, 2016)*.

However, the manuscript is not structured around this, the experiments are not strong enough to support the claims, and causality is not properly explored. Therefore, I would recommend to reject the article, while still providing some suggestions to the authors to strengthen their claims for another journal.
> As mentioned above, we strongly believe that RBPome reorganisation by RAS signalling is a major novel finding of our study. This is the main reason why the manuscript structured upon this finding, and then follows on to Ncl as a target which is further validated and studied. Regarding the suggestions provided, we indeed found them very helpful, and have taken them all on board in our revised manuscript (see the point-by-point response below). As a result, we believe that our manuscript is now much stronger, and hopefully acceptable for publication in EMBO Journal.

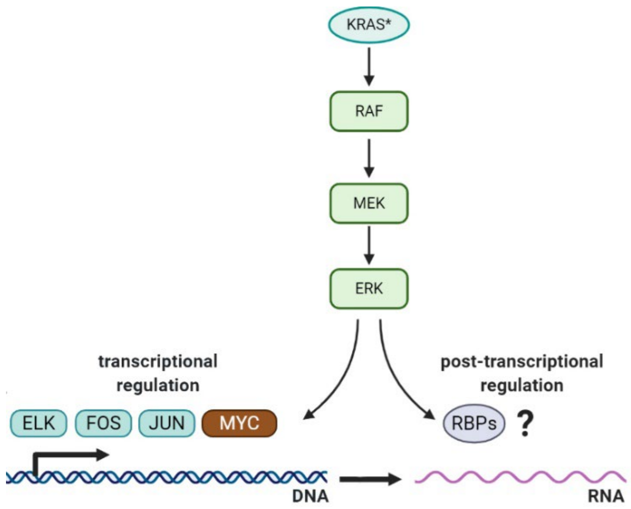

Rebuttal Figure 1: Unlike transcriptional regulation of gene expression, post-transcriptional regulation by oncogenic RAS signalling is not well understood. Our work is the first unbiased systematic study that shows RAS-MAPK signalling regulates the activity of several RBPs, unravelling a novel layer of post-transcriptional control at the level of RBP-RNA interactions.

1. The title should clearly indicate that the described mechanism is in pancreatic cancer.
> This is a fair point. We have now changed the title and also abstract accordingly.

2. It seems that the authors want to bring importance to the fact that downstream KRAS activation there is a change in RNA binding activity, rather than RBPs abundance. Therefore, it is suggest to move into supplementary the panels 1B to 1F, which will help to bring more clarity to the story.
> A very good suggestion. We have now done this.

3. All of the proteomics data is plotted in figures showing -log10 p-values and t-test differences. In the figure legends, sometimes thresholds are indicated with p-values, sometimes with FDR. The method sections does not describe any p-value adjustments done downstream of conditions comparisons - the only FDR described seems to be applying to the peptide mapping. However, a high throughput proteomic output would definitely require a p-value adjusting.
At present, it is unclear whether adjustments have not been done, or if the labelling of the axes on graphs Fig 1B, 1E, 1G, 2A, 2C requires correcting. In either cases, please correct.

> We apologise for these inconsistencies. We have now made sure that all our proteomic outputs are subjected to p-value correction (previously this was done for all our experiments apart from the OOPS analyses, due to lower number of proteins detected in these experiments). In the revised manuscript, we now perform p-value correction by calculating a Benjamini-Hochberg FDR for all OOPS analyses. In addition, all our volcano plots are now plotted as -Log 10 of FDR vs. difference score (see revised Figures 1, S1, and 2).

Please note that as a result of these changes, the supplemental datasets, the category enrichments analyses, and the network analysis in Figure 1 and S1 had to be updated (see revised Figure 1, S1, and Datasets S1-S7). In addition, the list of RBPs marked on Figure 2 had to be updated (see Figure 2A & 2B). Finally, the materials and methods section on the MS data analysis was updated to better describe the statistical analyses applied to our MS experiments (see 'Mass spectrometry data analysis' in the revised Supplementary Information)

In terms of thresholds being used, it is unclear for figure 2A and 2C why they are different in the figures (black lines) but in the legend, they are mentioned to be the same FDR 0.05.
> A permutation based corrected p-value (FDR) of 0.05, with an S0 offset value of 0.1, was used as the cut-off in these experiments (Tyanova *et al*, 2016). As FDR is calculated for each experiment, this may correspond to a different un-corrected p-value (which was plotted as the y-axis in the original volcano plot). We appreciate that this may have caused some confusion (plotting uncorrected p-value, but using and the corrected value as cut-off). We have now rectified this issue, plotting the -Log 10 of FDR vs. the difference score for these volcano plots (see revised Figure 2A and 2C).

On dataset S8, the FDR is not provided. If one uses the same p-value and difference thresholds in the two conditions (-/+ Trametinib), there are positions differentially phosphorylated upon KRAS induction even in presence of Trametinib.
> Apologies for this mistake. We now have provided both the uncorrected p-value as well as the FDR in the dataset (see revised dataset S8). As mentioned above, we now use the FDR values as opposed to uncorrected p-values as Y-axes for these plots.

Regarding adjusted p-values and FDRs for the proteomics datasets in the excel supplementary file, they should all be included - at present the information is given as -log10- p-value (S1). The rest of the datasets mention -log P value (please add the base of the -log, I can assume 10, but would be good to have it) this applies to S4, S5, S8. Additionally please consider using -log10 p value also for the CX-5461 enrichment analyses - at present it is being given as p-value.
> We apologise for these inconsistencies in reporting the p-values and FDRs. We have now corrected all of these. The revised datasets now report both uncorrected p-value as well as the corrected FDR values (as -Log 10 transformations).

4. A lot of the emphasis is put on how inhibition of MAPK signalling arm abrogates the RNA binding activity changes and/or changes in the RBPome downstream KRAS activation. However, fig 1E and 2C (once the same threshold as 2A is applied) show that there are still differences present when cells are treated with Trametinib. One or two sentences could be spent in the discussion to mention that the PI3K arm of signalling downstream KRAS might also be playing a role in regulating some functions of the RBPome.
> Previous work by *Tape et al* looked extensively into deciphering the different contributions of MAPK vs. PI3K pathways to KRAS signalling in this specific model of PDAC (Tape *et al*, 2016). It was demonstrated that KRAS primarily activates MAPK, but not PI3K signalling, in a cell-autonomous manner, and PI3K can only become activated non-autonomously through cell-cell communication with cancer associated fibroblasts, when cells are grown as co-cultures (Tape *et al.*, 2016). These results agree with our phospho-proteomic analysis which show a complete dependence on MAPK (see

revised Figures 2C). In the revised manuscript, we now add a few words to our results section to discuss this point better (see revised manuscript lines 207-211).

5. In the Phospho-motif analysis in fig S2, together with ERK1, also DUPS1 is highly enriched. What is potentially the explanation that DUSP1 motives are increased in sites which undergo enhanced phosphorylation?

> We thank the reviewer for highlighting this point, which we have now rectified. This was down to the way the enrichment analysis was performed (binary enrichment, scoring only presence or absence of motifs in the significant selection group relative to background, leading to categories with very small number of sites returning a high enrichment score if they were only present in the selection group. In this case, DUSP1 target sites are only present on ERK1 and 2 kinases, but 100% of these sites are in the significant selection group, hence the high enrichment score). After this point was raised, we decided that a better way of assessing the activation of potential kinases downstream of KRAS based on our phospho-proteomics data (which is the primarily point of this analysis) would be a 1D Wilcoxon Mann-Whitney annotation enrichment test. This test takes into account the category size (number of phospho-sites per motif) as well as the degree of the shift (increase or decrease) for a specific motif in the whole dataset, and therefore is a much better indicator of whether potential substrate sites of a given kinase are all significantly increased as a result of oncogenic KRAS activation. This new enrichment analysis data has now replaced the old motif analysis (See revised Figure S2A). We also provide the actual output of enrichment analysis as an additional NEW supplemental dataset (see revised Dataset S9)

6. A validation of Nucleolin phosphorylation downstream of ERK is required. If no antibody is available, an approach such as Phos Tag gel could be employed. These experiments should ideally be done in conditions which also support the fact that the phosphorylation is mediated by CK2.

> This was a very good suggestion. Using Immunoprecipitation of myc-Ncl in combination with Phos-tag gel analysis, we could indeed show an upward shift in Ncl Phos-tag migration upon KRAS induction, suggestive of increased phosphorylation. This shift was abrogated upon inhibiting ERK1/2 or CK2 kinases (see revised Figure S2F), supporting the notion that RAS, via ERK and CK2, triggers the phosphorylation of Ncl.

7. pCK2 substrate blots and CK2 activity downstream ERK1/2. The article you cite describes CK2 activation via ERK signalling via a specific phosphorylation (ERK2 docking groove and phosphorylates CK2α primarily at T360/S362). We were wondering if the phosphorylation is present also in your own dataset or perhaps other CK2 phosphorylations are present.

> We did not find the specific peptide corresponding to this phosphorylation in our phospho-proteomic analysis, but this is likely due to the fact that the tryptic peptide encompassing the site is too long and therefore not optimal for detection by standard shotgun proteomics approaches (58 residues long). However, after the reviewer raised this point, we found out that a new commercial antibody against this doubly phosphorylated site was available. We have now used this antibody to show that CK2 is indeed phosphorylated on T360/S362 upon KRAS induction, and this phosphorylation is dependent on Erk1/2 activity (see revised Figure 2E & 2F).

Are total levels of CK2 changing? Total CK2 should be blotted for the samples shown in Fig S2 B and D to help clarify the mechanism.

> Great suggestion. We can indeed show that total CK2 does not change in response to KRAS induction or Trametinib treatment (see revised Figure 2E).

8. Considering the centrality of CK2 in the mechanism that regulates Nucleolin activity, some space in the discussion should be devoted to it. How is CK2 regulated? Is it possible that what gets regulated downstream ERK is a phosphatase rather than a kinase?

> Again, a very good suggestion. In light of the new data in Revised Figure 2 and S2, we have now added a few words on CK2 regulation to our discussion (see revised manuscript lines 707-714).

9. Figure 2E and F seem to us problematic. First of all it is not clear if this analysis was done in a condition of KRAS ON or OFF - this might affect our comment in 9.c.
> These experiments were done in the KRAS ON condition. This was very important to us, as we wanted to make sure that we were specifically focusing on the inherent RNA binding activity of these mutants and not their RNA substrate availability (We reasoned, since it is well-known that RAS-MAPK activates transcription, lack of RAS-MAPK activity could lead to the RNA substrates of Ncl being absent/depleted, thus interfering with our interpretation). In the revised manuscript, we have made sure that this is clearly stated both in the manuscript text (see revised manuscript lines 275-287), as well as within the Figure 2G legend.

Indeed, this point is very important regarding the 9.c comment. Please note that when we later on show that pre-rRNA is the main target of Ncl, our qPCR analysis also clearly shows that pre-rRNA levels <u>in presence of KRAS</u> are equal between all the mutants (see the blue bars in revised Figure 4I).

a. You are extracting from the same sample the interphase and the organic phase. The total amount of a protein in your sample of origin is a sum of the two. Therefore, the total amount of Ncl S4D is higher than the others. This should be tackled by doing a blot which has an input portion, the interphase, and the organic phase all together.
> Although this appears to be the case, we should highlight that the vast majority of cellular proteins go to the organic phase (>1mg of proteins in the organic phase vs. ~10ug in the interface for these experiments, which started from ~10 million cells). This is likely due to the efficiency of UV-C crosslinking (i.e. only a small percentage of the RNA-bound proteome getting crosslinked). As a result, the organic phase is basically acting identical to a total sample control. However, we appreciate that this can be confusing to our readers, so to avoid confusions we have now done these experiments with a total lysate as input control (see revised Figure 2G & 2H). As total and organic phase blots are basically identical, we believe there is no value in showing both (if anything, it has the potential to confuse the readers). Moreover, if total RNA is to be used for balancing the loadings (as the reviewer suggested in comment 9.c and is now done for these experiments), this can only be done for the total and interface fractions, in which RNA is also present, and not the organic phase.

Additionally, the blot of endogenous Nucleolin in input, interphase, and organic phase in KRAS ON and KRAS OFF conditions would further support this mechanism.
> An excellent suggestion. We have now done this, but have decided to show the results as part of Figure S1, since we feel they fit there better as per flow of our story (see new Figure S1M & S1N). As mentioned in the above comment, there is no further value in showing the organic phase, so these experiments are also done only with interface vs. total comparisons.

b. It is highly unclear as to why in the interphase there are two bands, and only one in the organic.
> Depending on the cell-type and context, Ncl has been shown to occasionally appear as two or more bands during SDS-PAGE analysis, with the largest most prominent band corresponding to the full-length protein resolving at just over the 100kDa mark. The cause of this is proposed to be due to the proteolytic cleavage of Ncl inside the cell through various proteases, but the exact triggers are not well characterised (Chen *et al*, 1991; Hsu *et al*, 2015; Tosoni *et al*, 2015). In iKras cells, we detect endogenous Ncl as a doublet but the higher band is far more prominent, although if the membranes are highly overexposed, we can start to see traces of the lower band too (See Rebuttal Figure 2).

Interestingly, we see this lower band more prominently when Ncl is overexpressed. However, it is when we look at the interface from overexpressing cells that the lower band becomes most prominent

(See revised Figure 2G – compare Interface and Lysate). This probably indicates a yet undiscovered interplay between the proteolytic cleavage of Ncl and regulation of its RNA binding activity. Despite this being a very interesting observation, we feel that it is not relevant to our story's focus. We have therefore chosen not to delve into this interplay in our manuscript. Instead, we simply show both band as they appear in our blots (but quantifications are always only performed on the higher band).

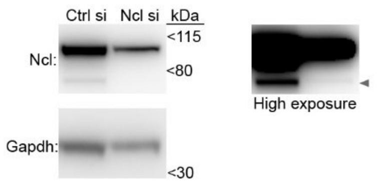

Rebuttal Figure 2: Ncl primarily resolves as a prominent band at ~100kDa during SDS-PAGE, with a secondary lower band at below 80kDa becoming more clearly at higher exposures. IKras1 cells grown in presence of doxycycline were treated with siRNAs against non-targeting Control (Ctrl si) or Ncl (Ncl si) for 72 hrs, prior to lysis, SDS-PAGE, and western blotting with an anti-Nucleolin antibody. Gapdh was also blotted as loading control. The grey triangle marks the lower molecular weight band of Ncl, which becomes visible more prominently after high exposure.

c. This figure is the central one in proving your novel mechanism: to prove that phosphorylated Ncl increases its RNA binding capabilities. In fig 4G you show that expression of S4D Ncl in absence of KRAS activation increases unprocessed pre rRNA. Likewise, in figure 4C, with KRAS on there is more unprocessed pre-rRNA. These data both point at the fact that what happens is not that Ncl RNA binding activity is increased, but at the fact that there is simply more pre-rRNA and therefore Ncl binds "more" to it. But the real regulation happens at the RNA expression level, to which Ncl binding is just a consequence.
> This was discussed earlier in response to point 9. As mentioned, to prove that phospho-mimicking Ncl has higher RNA binding activity, it was paramount to do these analyses with KRAS ON to make sure RNA expression changes are not the driver of these observations. As we highlighted above, please also note that in the condition of KRAS ON, there is no difference in pre-rRNA levels between different mutants (see Fig 4I), further supporting our claim that Ncl phosphorylation does indeed enhance its inherent RNA binding activity.

To tackle this issue, I think you could take advantage of the tagged constructs you have already generated. An IP of the three Ncl versions using equal RNA input material, rather than equal protein input would help to clarify if there is actually an increase in binding activity regardless of how much RNA is present.
>A very good suggestion to balance loadings based on equal RNA. Indeed, we could extract and measure total RNA levels from both total input lysates as well as the interface fractions, and normalise the loadings based on equal RNA amount. This is now done for the OOPS western experiments and the trend is similar as before, with the phospho-mimicking S4D mutant clearly showing a significant increase in its RNA binding activity (see revised Figure 2G and 2H). Interestingly, in these new experiments there is also a hint of a decrease (albeit just below the statistical significance) in the RNA binding activity of the phospho-defective S4A mutant, compared with WT. This is indeed expected, since a fraction of WT must be phosphorylated under KRAS ON conditions, while this cannot happen for the S4A mutant.

10. Ncl KD tends to have an effect across all the phenotypes and molecular mechanisms tested both in KRAS ON and KRAS OFF conditions (eg comparing KRAS OFF Ctrl vs the two siRNAs rather than KRAS ON vs KRAS OFF). The only exception to this seems to be in vivo (Fig 5E and 5F)
> This is correct. However, we would like to point out that these conditions are not directly comparable due to the treatment timings. KRAS OFF corresponds to 48hrs of dox removal (leading to a gradual loss of mutant Kras – See Figure S1A), while Ncl siRNAs are knockdowns done for several days (two rounds of siRNA transfections at 48hr intervals). Therefore, the most valid comparison in these experiments is KRAS ON vs. OFF, in different backgrounds (Ctrl vs. Ncl KD), as done by us.

11. Figures 6A, 6B, 6C, S6A, and S6B are addressing the responses of cells to CX-5461 - however these experiments have all been performed only in KRAS ON conditions. Please do consider that KRAS OFF cells are suffering heavily from CX-5461 treatment, possibly more than KRAS ON, at the lowest concentration. Therefore, it would be important to show the same effects are present (for rRNA/nucleolar phenotypes) or absent (DNA damage) also in KRAS OFF conditions.
> Great suggestion! We have now repeated these experiments in both KRAS ON and OFF conditions. Similar to Ncl depletion, CX-5461 can block KRAS-induced rRNA synthesis (see revised Figure 6A-C), but DNA damage is only seen at high doses, irrespective of the KRAS status (see revised Figure S6A-B).

We thank the reviewer for highlighting this point. Due to the interest on CX-5461 from a pre-clinical/clinical perspective, we believe it will be particularly useful to the community to see that the DNA damage induction by CX-5461 is strictly dose-dependent, does not occur at low concentrations of the drug that still inhibit rRNA synthesis, and is irrespective of the KRAS oncogene status.

12. Dataset S12 is an enrichment based on proteomics data not shown. Ideally, as for all the other figures you have shown with treatments, the dataset should be included in the supplementary tables.
> Apologies for missing this. We have now added this dataset to the supplementary tables (See revised Supplementary Dataset S13 and S14). Please also note that all the proteomics raw files and search results have now been uploaded to the PRIDE Proteomics repository, which for now are accessible via the below provided usernames and passwords, but will be made publically available upon the publication of the manuscript:

1- Project Name: Analysis of oncogenic Kras-induced changes in the RNA-bound proteome of PDAC cells
Project accession: PXD030825
Reviewer account details:
Username: reviewer_pxd030825@ebi.ac.uk
Password: FhoUuxCN

2- Project Name: Analysis of oncogenic KRAS impact on the phospho-proteome of PDAC cells. Project accession: PXD030893
Reviewer account details:
Username: reviewer_pxd030893@ebi.ac.uk
Password: hIbUXUES

3- Project Name: Quantitative timecourse proteomics analysis of oncogenic KRAS removal in mouse Pancreatic Ductal Adenocarcinoma Cells
Project accession: PXD038441
Reviewer account details:
Username: reviewer_pxd038441@ebi.ac.uk
Password: C6B0Su62

4- Project Name: Quantitative timecourse analysis of oncogenic KRAS expression impact on the proteme of pancreatic ductal adenocarcinoma cells.
Project accession: PXD038440
Reviewer account details:
Username: reviewer_pxd038440@ebi.ac.uk
Password: GMeKeiaL

5- Project Name: Analysis of Nucleolin impact on the KRAS-induced proteomic changes in mouse Pancreatic ductal adenocarcinoma cells.

Project accession: PXD038338
Reviewer account details:
Username: reviewer_pxd038338@ebi.ac.uk
Password: CtdcEcrt

6- Project Name: Quantitative analysis of the impact of CX-5461 on the proteome of Pancreatic ductal adenocarcinoma cells.
Project accession: PXD038439
Reviewer account details:
Username: reviewer_pxd038439@ebi.ac.uk
Password: iC9HrhZP

13. Minor points:

a. Figure S6: please check legend and panel numbering, they are not matching
>Thank you for bringing this to our attention. We have now corrected this.

b. Please consider using a different colour scheme for the WT/S4A/S4D data as red and blue are already being heavily used by KRAS ON and OFF and it can get confusing. In particular since you have used blue for KRAS ON (which should correspond to phosphorylated Ncl) and then again used it for the phospho-defective Ncl.
> A very good suggestion. We have now changed the mutant colour scheme to distinguish it from our KRAS ON-blue / KEAS OFF-red scheme.

Rebuttal References:

Abdelmohsen K, Gorospe M (2012) RNA-binding protein nucleolin in disease. *RNA Biol* 9: 799-808

Chen CM, Chiang SY, Yeh NH (1991) Increased stability of nucleolin in proliferating cells by inhibition of its self-cleaving activity. *J Biol Chem* 266: 7754-7758

Gilles ME, Maione F, Cossutta M, Carpentier G, Caruana L, Di Maria S, Houppe C, Destouches D, Shchors K, Prochasson C *et al* (2016) Nucleolin Targeting Impairs the Progression of Pancreatic Cancer and Promotes the Normalization of Tumor Vasculature. *Cancer research* 76: 7181-7193

Hsu TI, Lin SC, Lu PS, Chang WC, Hung CY, Yeh YM, Su WC, Liao PC, Hung JJ (2015) MMP7-mediated cleavage of nucleolin at Asp255 induces MMP9 expression to promote tumor malignancy. *Oncogene* 34: 826-837

Tape CJ, Ling S, Dimitriadi M, McMahon KM, Worboys JD, Leong HS, Norrie IC, Miller CJ, Poulogiannis G, Lauffenburger DA *et al* (2016) Oncogenic KRAS Regulates Tumor Cell Signaling via Stromal Reciprocation. *Cell* 165: 910-920

Tosoni E, Frasson I, Scalabrin M, Perrone R, Butovskaya E, Nadai M, Palu G, Fabris D, Richter SN (2015) Nucleolin stabilizes G-quadruplex structures folded by the LTR promoter and silences HIV-1 viral transcription. *Nucleic acids research* 43: 8884-8897

Tyanova S, Temu T, Sinitcyn P, Carlson A, Hein MY, Geiger T, Mann M, Cox J (2016) The Perseus computational platform for comprehensive analysis of (prote)omics data. *Nat Methods* 13: 731-740

Dr. Faraz K Mardakheh
Barts Cancer Institute, Queen Mary University of London
Centre for Cancer Cell and Molecular Biology
Charterhouse square
London EC1M 6BQ
United Kingdom

12th Jan 2023

Re: EMBOJ-2022-110902R-Q
An RNA-binding switch drives ribosome biogenesis and pancreatic tumorigenesis downstream of RAS oncogene

Dear Faraz,

Thank you for submitting a revised version of your manuscript EMBOJ-2022-110902, which we had previously rejected after review. As we had discussed, I contacted the initial referees, however, referee #2 unfortunately could not look at the revised manuscript. Therefore, I contacted two additional experts, who reviewed the manuscript together with the initial reports and your response. We have now received those comments, as well as the report from the initial referee #1 (please see below).

As you will see, while referee #1 finds that her/his concerns have been sufficiently addressed, referee #3 and referee #4 are not convinced that the critical issues raised during the initial review have been fully resolved, despite acknowledging that the manuscript has improved. As all referees indicate the interest to the field, we would be open to consider the study further for publication, if the remaining crucial issues are resolved. However, in our view, this involves further experiments in an exceptional second round of experimental revision. Here, it would be required that referee #3's concerns regarding an alternative explanation for the specificity of the observed effects (requirement for elevated ribosome biogenesis) and the nucleolin phosphorylation sites are resolved, either by the suggested experiment(s) or other suited approaches. Please be aware that we will not be able to proceed, if these issues are not resolved and there is no clear support from the referee(s). Given the amount of time and effort you have already spent revising the manuscript, I could thus understand if you would like to consider other options, and can offer to discuss with other EMBO Press journals in case you are interested in a potential transfer. Once you have looked through the comments in detail, please do not hesitate to contact us to discuss potential ways forward. In case you do decide to submit a revision, please remember to provide a detailed response to each of the comments and to adhere to the general formatting instructions below.

Thank you again for the opportunity to consider your work for publication.

Kind regards,
Stefanie

Stefanie Boehm
Editor
The EMBO Journal

***IMPORTANT NOTE: we now perform an initial quality control of all revised manuscripts before re-review. Your manuscript will FAIL this control and the handling will be DELAYED if the following APPLIES:

1) A data availability section is missing.
2) Your manuscript contains error bars based on n=2. Please use scatter blots showing the individual datapoints in these cases. The use of statistical tests needs to be justified.

When submitting your revised manuscript, please carefully review the instructions that follow below. Failure to include requested items will delay the evaluation of your revision.***

-

When submitting your revised manuscript, please carefully review the instructions below and include the following items:

1) A .docx formatted version of the manuscript text (including legends for main figures, EV figures and tables). Please make sure

that the changes are highlighted to be clearly visible.

2) Individual production quality figure files as .eps, .tif, .jpg (one file per figure).

3) A .docx formatted letter INCLUDING the reviewers' reports and your detailed point-by-point response to their comments. As part of the EMBO Press transparent editorial process, the point-by-point response is part of the Review Process File (RPF), which will be published alongside your paper.

4) A complete author checklist, which you can download from our author guidelines (https://wol-prod-cdn.literatumonline.com/pb-assets/embo-site/Author%20Checklist%20-%20EMBO%20J-1561436015657.xlsx). Please insert information in the checklist that is also reflected in the manuscript. The completed author checklist will also be part of the RPF.

5) All corresponding authors are required to supply an ORCID ID for their name upon submission of a revised manuscript.

6) Before submitting your revision, primary datasets produced in this study need to be deposited in an appropriate public database. Please remember to provide a reviewer password if the datasets are not yet public.

Please see the instructions below on how to format the Data Availability section (see https://www.embopress.org/page/journal/14602075/authorguide#datadeposition). Please note that the Data Availability Section is restricted to new primary data that are part of this study.

Data availability

The datasets (and computer code) produced in this study are available in the following databases:

[data type]: [name of the resource] [accession number/identifier/doi] ([URL or identifiers.org/DATABASE:ACCESSION])

*** Note - All links should resolve to a page where the data can be accessed. ***

7) Our journal encourages inclusion of *data citations in the reference list* to directly cite datasets that were re-used and obtained from public databases. Data citations in the article text are distinct from normal bibliographical citations and should directly link to the database records from which the data can be accessed. In the main text, data citations are formatted as follows: "Data ref: Smith et al, 2001" or "Data ref: NCBI Sequence Read Archive PRJNA342805, 2017". In the Reference list, data citations must be labeled with "[DATASET]". A data reference must provide the database name, accession number/identifiers and a resolvable link to the landing page from which the data can be accessed at the end of the reference. Further instructions are available at < https://www.embopress.org/page/journal/14602075/authorguide#referencesformat >.

8) We would also encourage you to include the source data for figure panels that show essential data. Numerical data can be provided as individual .xls or .csv files (including a tab describing the data). For 'blots' or microscopy, uncropped images should be submitted (using a zip archive or a single pdf per main figure if multiple images need to be supplied for one panel).

Please note that source data should be uploaded in the following format:
a) For main figures: please upload the source data as one zip file per figure and label it as source_data_figX.
b) For EV figures please compile individual (zip) files for each figure, then combine these and upload one zip file for all figures. Please label this as source_data_EVfigures.
c) For Appendix figures please also make one file per figure, combine all Appendix files into one final zip file for all figures and label it as source_data_Appendix.

Additional information on source data and instruction on how to label the files are available at < https://www.embopress.org/page/journal/14602075/authorguide#sourcedata >.

9) We replaced Supplementary Information with Expanded View (EV) Figures and Tables that are collapsible/expandable online (see examples in http://msb.embopress.org/content/11/6/812). A maximum of 5 EV Figures can be typeset. EV Figures should be cited as 'Figure EV1, Figure EV2" etc.. in the text and their respective legends should be included in the main text after the legends of regular figures.

- For the figures that you do NOT wish to display as Expanded View figures, they should be bundled together with their legends in a single PDF file called *Appendix*, which should start with a short Table of Content. Appendix figures should be referred to in the main text as: "Appendix Figure S1, Appendix Figure S2" etc. See detailed instructions regarding expanded view here: < https://www.embopress.org/page/journal/14602075/authorguide#expandedview>.

- Additional Tables/Datasets should be labeled and referred to as Table EV1, Dataset EV1, etc. Legends have to be provided in a separate tab in case of .xls files. Alternatively, the legend can be supplied as a separate text file (README) and zipped together with the Table/Dataset file.

10) When assembling figures, please refer to our figure preparation guideline in order to ensure proper formatting and readability in print as well as on screen:
https://wol-prod-cdn.literatumonline.com/pb-assets/embo-site/EMBOPress_Figure_Guidelines_061115-1561436025777.pdf

Please remember: Digital image enhancement is acceptable practice, as long as it accurately represents the original data and conforms to community standards. If a figure has been subjected to significant electronic manipulation, this must be noted in the figure legend or in the 'Materials and Methods' section. The editors reserve the right to request original versions of figures and the original images that were used to assemble the figure. Finally, we generally encourage uploading of numerical as well as gel/blot image source data; for details see: embopress.org/page/journal/14602075/authorguide#sourcedata

At EMBO Press, we ask authors to provide source data for the main manuscript figures. Our source data coordinator will contact you to discuss which figure panels we would need source data for and will also provide you with helpful tips on how to upload and organize the files.

Further information is available in our Guide For Authors:
https://www.embopress.org/page/journal/14602075/authorguide

****
Revision to The EMBO Journal should be submitted online within 90 days, unless an extension has been requested and approved by the editor; please click on the link below to submit the revision online before 12th Apr 2023:
Link Not Available

If you choose to alternatively have this study further considered by another EMBO Press publication, please use the following hyperlink to directly transfer the manuscript, optionally with inclusion of referee reports and identities:
Link Not Available

****

Referee #1:

The authors have satisfactorily addressed my comments, and this exciting manuscript is ready for publication.

Referee #3:

In general, I believe the responses of the authors are appropriate and that the manuscript is improved. However, I still share Referee 2's concerns around some key aspects of novelty and causality. I don't want to roll out the "more mechanism required" objection but I believe that additional work would be required to justify publication in EMBO J. In my opinion, the demonstration that RAS/ERK driven changes in the interaction of a network of RNA binding proteins and that this can result in altered ribosome biogenesis that can be targeted is sufficiently novel to be potentially of significant interest to the field. My concern is that there is little focus on understanding how this rewiring occurs, for example, how the translation of RBPs is regulated specifically? What are the specific effects of inhibiting ERK or CK2 on the RAS-induced interactome?

Instead, the investigators focus on one attractive but logical target, nucleolin. Instead, the investigators focus on one attractive but logical target, nucleolin. As commented on specifically below, the characterisation of the regulation of nucleolin by RAS/ERK/CK2 is mostly well investigated in the revised manuscript but with some limitations. However, the impact of this manuscript is limited by the fact that the use of nucleolin knockdown or CX-5461 treatment to demonstrate specificity of response could equally be explained by a simple requirement for each for elevated ribosome biogenesis rather than a role for increased binding of nucleolin upon RAS driven activation of CK2 dependent nucleolin phosphorylation.

To Comment specifically on the negative comments of Referee 2:
Referee 2 made several suggestions regarding data presentation and additional experiments that have in most part been addressed. However, the key point made by this reviewer questioned the novelty of the study being reliant on demonstrating Ras activation of ERK leads CK2-dependent phosphorylation of nucleolin and that the experiments addressing this are not sufficiently convincing. In my opinion, the authors have partially addressed this important concern. The revised manuscript now uses phosphor-specific antibodies to show RAS induces CK2 phosphorylation, but I think these experiments should be performed +/- imatinib to demonstrate the dependence on ERK. The referee suggested using a phosphor-tag gel to reveal evidence of nucleolin phosphorylation and the authors provide new data demonstrating that gel retardation of MYC-tagged

nucleolin in response to RAS was abrogated by ERK and CK2 inhibitors. This is a key experiment but would have been more convincing if the authors demonstrated phosphorylation on the relevant nucleolin sites. As they are proficient in phosphopeptide anaylsis, this should be straight forward on immunoprecipitated, exogenously expressed protein.

Referee #4:

This is a very interesting and novel study. While it is true that a number of individual RNA binding proteins (RBPs) have increased expression in certain cancers, as far as I am aware, this is only the second study that has been carried out which examines how the global RBPome is changed in tumorigenesis. Moreover, this research differs from prior work, which examined the RBPs in a metastatic melanoma, and this is the first time that the role of signalling to RBPome, in this case via Ras, has been examined at a cell wide level. The data very clearly show that nucleolin is a key target of Ras (amongst other interesting RBPs), and that moreover, via iCLP, that rRNA production and ribosome biogenesis is upregulated in a nucleolin-dependent manner. This is important since the role of ribosome biogenesis in cancer development is somewhat under-appreciated. This final point is very much strengthened by the animal data to show that PDAC dependency can be targeted by inhibition of ribosome biogenesis with CX-5461. The additional experiments carried out in response the original reviewers' comments have improved the manuscript and I have no specific comments to make about the data.

Referee #1:

The authors have satisfactorily addressed my comments, and this exciting manuscript is ready for publication.
> We thank the reviewer for their comments and suggestions, which made our manuscript significantly improved.

Referee #3:

In general, I believe the responses of the authors are appropriate and that the manuscript is improved. However, I still share Referee 2's concerns around some key aspects of novelty and causality. I don't want to roll out the "more mechanism required" objection but I believe that additional work would be required to justify publication in EMBO J. In my opinion, the demonstration that RAS/ERK driven changes in the interaction of a network of RNA binding proteins and that this can result in altered ribosome biogenesis that can be targeted is sufficiently novel to be potentially of significant interest to the field. My concern is that there is little focus on understanding how this rewiring occurs, for example, how the translation of RBPs is regulated specifically? What are the specific effects of inhibiting ERK or CK2 on the RAS-induced interactome?
> Our RIC analysis (Figure 1C) reveals that the RNA binding activity of 23 RBPs is increased downstream of RAS, while 42 RBPs show a decrease in their RNA-binding activity. While we don't know how the decrease is specifically mediated, our data in Figure 2A-C implies that phosphorylation is likely associated with the observed increase, at least for several of the identified RBPs, although this is only a correlation. To prove causality, the same work we did for Nucleolin needs to be performed for each of these RBPs (e.g. generation of phospho-mimicking and phospho-defective mutants, and showing the effect of these mutagenesis on the RNA binding activity). To perform all these experiments for every detected RBP is simply unrealistic, hence we decided on the approach of choosing one attractive target (Nucleolin) and validating it in depth.

Instead, the investigators focus on one attractive but logical target, nucleolin. As commented on specifically below, the characterisation of the regulation of nucleolin by RAS/ERK/CK2 is mostly well investigated in the revised manuscript but with some limitations. However, the impact of this manuscript is limited by the fact that the use of nucleolin knockdown or CX-5461 treatment to demonstrate specificity of response could equally be explained by a simple requirement for each for elevated ribosome biogenesis rather than a role for increased binding of nucleolin upon RAS driven activation of CK2 dependent nucleolin phosphorylation > It is the data in Figures 4G & 4H that reveal a role for phospho-regulation of Nucleolin in control of rRNA synthesis (not the Nucleolin knockdown or the CX-5461 treatment experiments that the reviewer is referring to). This is described in detail in the results section (lines 379-399), and then discussed in detail in our discussion section (lines 550-560). In short, these results suggest that the novel phopsho-regulation of Nucleolin by Kras revealed in our paper, is crucial for pre-rRNA synthesis, but not other further downstream steps of ribosome biogenesis (e.g. pre-rRNA processing and RP accumulation).

To Comment specifically on the negative comments of Referee 2:
Referee 2 made several suggestions regarding data presentation and additional experiments that have in most part been addressed. However, the key point made by this reviewer questioned the novelty of the study being reliant on demonstrating Ras activation of ERK leads CK2-dependent phosphorylation of nucleolin and that the experiments addressing this are not sufficiently convincing. In my opinion, the authors have partially addressed this important concern. The revised manuscript now uses phosphor-specific antibodies to show RAS induces CK2 phosphorylation, but I

think these experiments should be performed +/- imatinib to demonstrate the dependence on ERK > We suspect that the reviewer meant Trametinib (to inhibit MEK-ERK signalling) and not imatinib (a Tyrosine kinase inhibitor). If so, we would like to highlight that this was already done in the first revised manuscript (please see Figures 2E and 2F. The data is both with or without Trametinib to show dependence on ERK). As the reviewer had missed this point, in the newly revised manuscript we have modified the text to better highlight it (see manuscript lines 245-249).

The referee suggested using a phosphor-tag gel to reveal evidence of nucleolin phosphorylation and the authors provide new data demonstrating that gel retardation of MYC-tagged nucleolin in response to RAS was abrogated by ERK and CK2 inhibitors. This is a key experiment but would have been more convincing if the authors demonstrated phosphorylation on the relevant nucleolin sites. As they are proficient in phosphopeptide anaylsis, this should be straight forward on immunoprecipitated, exogenously expressed protein. > Although it is correct that an MS based analysis instead of Phos-tag could have been used for these validations, the point of the Phos-Tag work was to validate our phospho-proteomics data by method other than MS, and was specifically requested by the previous reviewer (see reviewer #2 comment 6). To address the point raised about the relevant sites of phosphorylation, we discussed this with the editor (Dr Boehm), and it was decided that the best approach would be to use our Ncl S4A (phospho-defective) mutant to show that the Kras-induced shift in Phos-tag gel migration of Ncl is dependent on the relevant Ncl residues. The results of this new experiment is now included in the revised manuscript (new Fig EV2G).

Collectively, on this specific point (the mechanism of Ncl phospho-regulation), we believe that the evidence provided in support of Kras activation of Erk1/2 leading to CK2 activation and phosphorylation of Nucleolin, is very substantial. Briefly, we show:

1. Quantitative phospho-proteomics data demonstrating that Kras results in phosphorylation of the S28, S34, S40 & S41 of Nucleolin (Fig 2A).
2. Quantitative phospho-proteomics data showing that these specific phosphorylations are lost upon inhibition of Erk1/2 activity (Fig 2C), thereby demonstrating Erk1/2 dependence.
3. Kinase prediction analysis of the specific Ncl sites strongly implicating CK2 as the culprit kinase (Fig 2D).
4. Phospho-motif analysis of the phospho-proteomics data suggesting that CK2 is amongst the activated kinases downstream of Kras (Fig EV2A).
5. Western blot analysis showing CK2 phosphorylation by Erk1/2 on specific residues that are known to activate the kinase (Fig 2E and 2F).
6. Western blot analysis showing Erk1/2 mediated increase in phosphorylation of CK2 substrates downstream or Kras, thereby proving CK2 activation (Fig EV2D).
7. Phos-tag analysis validating that Ncl phosphorylation downstream of Kras depends on both Erk1/2 and CK2 (Fig EV2F)
8. Phos-tag analysis validating that Ncl phosphorylation downstream of Kras occurs on the same sites initially identified by phospho-proteomics (Fig EV2G).

In our opinion, the above listed results overwhelmingly support our postulated mechanism of Ncl phosphorylation downstream of Kras, through Erk1/2 mediated CK2 activation.

Referee #4:

This is a very interesting and novel study. While it is true that a number of individual RNA binding proteins (RBPs) have increased expression in certain cancers, as far as I am aware, this is only the

second study that has been carried out which examines how the global RBPome is changed in tumorigenesis. Moreover, this research differs from prior work, which examined the RBPs in a metastatic melanoma, and this is the first time that the role of signalling to RBPome, in this case via Ras, has been examined at a cell wide level. The data very clearly show that nucleolin is a key target of Ras (amongst other interesting RBPs), and that moreover, via iCLP, that rRNA production and ribosome biogenesis is upregulated in a nucleolin-dependent manner. This is important since the role of ribosome biogenesis in cancer development is somewhat under-appreciated. This final point is very much strengthened by the animal data to show that PDAC dependency can be targeted by inhibition of ribosome biogenesis with CX-5461. The additional experiments carried out in response the original reviewers' comments have improved the manuscript and I have no specific comments to make about the data.

> We thank the reviewer for their kind words, particularly highlighting the novelty of our findings in the context of previous research.

Dr. Faraz K Mardakheh
Barts Cancer Institute, Queen Mary University of London
Centre for Cancer Cell and Molecular Biology
Charterhouse square
London EC1M 6BQ
United Kingdom

11th Feb 2023

Re: EMBOJ-2022-110902R1
An RNA-binding switch drives ribosome biogenesis and pancreatic tumorigenesis downstream of RAS oncogene

Dear Dr. Mardakheh, dear Faraz,

Thank you again for submitting a revised version of your manuscript in response to referee #3's remaining concerns. As we had discussed, we again contacted this reviewer and have now received her/his comments (please see below). The referee still finds that further mechanistic insight into the role of Ncl phosphorylation would be of interest, but also acknowledges the added data and the interest for the field. Thus, after discussing amongst the team, I am happy to say that we have decided to proceed with manuscript. Therefore, I would ask you to please resolve a number of editorial issues that are listed in detail below. Please use the document that the data editors have added their comments to for any changes. If you have any further questions regarding the specific points listed below or the final manuscript version, please contact us.

Thank you again for giving us the chance to consider your manuscript for The EMBO Journal.

Kind regards,

Stefanie

Stefanie Boehm
Editor
The EMBO Journal

Formatting changes required for revised version of the manuscript

1) Please update the "Disclosure and competing interests" section to "Disclosure and Competing Interests Statement" and explicitly state if there is nothing to declare.

2) CRediT has replaced the traditional author contributions section because it offers a systematic machine readable author contributions format that allows for more effective research assessment. Please *remove* the Authors Contributions from the manuscript and use the free text boxes beneath each contributing author's name in our system to add specific details

3) Please upload each dataset EV1-14 individually and add a legend to each file, for example as a separate sheet in the excel file.

4) At EMBO Press we ask authors to provide source data for the main and EV figures. Our source data coordinator will contact you to discuss which figure panels we would need source data for and will also provide you with helpful tips on how to upload and organize the files.

5) Our data editors have raised several queries with the data descriptors in the *figure legends*, which you will find as comments in the Word document EMBOJ-2022-110902_data_editing.docx (as part of your submission and attached to this message). I would appreciate if you incorporated the requested final text modifications and answered the figure legend queries directly in this version, uploading the edited main text document upon resubmission with changes/additions still highlighted via the "Track changes" option.

Further information is available in our Guide For Authors:
https://www.embopress.org/page/journal/14602075/authorguide

****

In the interest of ensuring the conceptual advance provided by the work, we recommend submitting a revision within 3 months (12th May 2023). Please discuss the revision progress ahead of this time with the editor if you require more time to complete the revisions. Use the link below to submit your revision:

Link Not Available

****

Referee #3:

As stated in my initial review, the demonstration that RAS/ERK driven changes in the interaction of a network of RNA binding proteins and that this can result in altered ribosome biogenesis that can be targeted is sufficiently novel to be potentially of significant interest to the field. To clarify, the in vivo importance of phosphorylation of Ncl is not addressed by nucleolin knockdown or CX-5461 treatment. However, the manuscript has been further improved by inclusion of the new Phos-Tag data demonstrating lack of Ras-dependent bandshift of the Ncl S4A mutant is an important addition providing convincing evidence that K-Ras induces CK2 dependent on nucleolin. I apologise for missing the +/- trametinib experiment shown in Figs 2 E and F.

I do believe that the authors have missed a great opportunity to extend their interesting observations about the role of Ncl phosphorylation in driving rRNA synthesis and
was disappointed in the authors lack of response to the question of how this overall re-wiring of the RNA interactome. For example, no commentary was included on the regulation of RBP translation and its possible roles. To simply state that it was not possible to perform all these experiments for all the RBPs is missing the point. They do identify the requirement for additional factors for Ras induced ribosome biogenesis following the stimulation of rRNA synthesis which adds an additional level of understanding and potential investigation.

The authors addressed the remaining minor changes.

3rd Revision - Editorial Decision **12th Mar 2023**

Dear Dr Mardakheh,

Thank you for submitting the revised version of your manuscript and your patience with our response. I have now evaluated your amended manuscript and concluded that the remaining minor concerns have been sufficiently addressed.

Thus, I am pleased to inform you that your manuscript has been accepted for publication in the EMBO Journal.

Please note that it is EMBO Journal policy for the transcript of the editorial process (containing referee reports and your response letter) to be published as an online supplement to each paper.

Also, in case you might NOT want the transparent process file published at all, you will also need to inform us via email immediately. More information is available here:
https://www.embopress.org/page/journal/14602075/authorguide#transparentprocess

----

Please note that in order to be able to start the production process, our publisher will need and contact you shortly regarding the page charge authorisation and licence to publish forms.

Authors of accepted peer-reviewed original research articles may choose to pay a fee in order for their published article to be made freely accessible to all online immediately upon publication. The EMBO Open fee is fixed at $6,540 USD / £5,310 GBP / €5,900 EUR (+ VAT where applicable).

We offer two licenses for Open Access papers, CC-BY and CC-BY-NC-ND.
For more information on these licenses, please visit:
http://creativecommons.org/licenses/by-nc-nd/3.0/deed.en_US

Should you be planning a Press Release on your article, please get in contact with embojournal@wiley.com as early as possible, in order to coordinate publication and release dates.

On a different note, I would like to alert you that EMBO Press is currently developing a new format for a video-synopsis of work published with us, which essentially is a short, author-generated film explaining the core findings in hand drawings, and, as we believe, can be very useful to increase visibility of the work. This has proven to offer a nice opportunity for exposure i.p. for the first author(s) of the study. Please see the following link for representative examples and their integration into the article web page:
https://www.embopress.org/video_synopses
https://www.embopress.org/doi/full/10.15252/embj.2019103932

Please let me know, should you be interested to engage in commissioning a similar video synopsis for your work. According operation instructions are available and intuitive.

Finally, we have noted that the submitted version of your article is also posted on the preprint platform bioRxiv. We would appreciate if you could alert bioRxiv on the acceptance of this manuscript at The EMBO Journal in order to allow for an update of the entry status. Thank you in advance!

If you have any questions, please do not hesitate to call or email the Editorial Office.

Thank you again for this contribution to The EMBO Journal and congratulations on a successful publication! Please consider us again in the future for your most exciting work.

Best regards,

Daniel Klimmeck

Daniel Klimmeck, PhD
Senior Editor
The EMBO Journal
EMBO
Postfach 1022-40
Meyerhofstrasse 1
D-69117 Heidelberg
contact@embojournal.org
Submit at: http://emboj.msubmit.net

EMBO Press Author Checklist

Corresponding Author Name: Faraz K. Mardakheh	
Journal Submitted to: EMBO Journal	
Manuscript Number: EMBOJ-2022-110902R-Q	

USEFUL LINKS FOR COMPLETING THIS FORM
The EMBO Journal - Author Guidelines
EMBO Reports - Author Guidelines
Molecular Systems Biology - Author Guidelines
EMBO Molecular Medicine - Author Guidelines

Reporting Checklist for Life Science Articles (updated January 2022)
This checklist is adapted from Materials Design Analysis Reporting (MDAR) Checklist for Authors. MDAR establishes a minimum set of requirements in transparent reporting in the life sciences (see Statement of Task: 10.31222/osf.io/9sm4x). Please follow the journal's guidelines in preparing your manuscript.
Please note that a copy of this checklist will be published alongside your article.

Abridged guidelines for figures
1. Data
The data shown in figures should satisfy the following conditions:
- the data were obtained and processed according to the field's best practice and are presented to reflect the results of the experiments in an accurate and unbiased manner.
- ideally, figure panels should include only measurements that are directly comparable to each other and obtained with the same assay.
- plots include clearly labeled error bars for independent experiments and sample sizes. Unless justified, error bars should not be shown for technical replicates.
- if n<5, the individual data points from each experiment should be plotted. Any statistical test employed should be justified.
- Source Data should be included to report the data underlying figures according to the guidelines set out in the authorship guidelines on Data Presentation.

2. Captions
Each figure caption should contain the following information, for each panel where they are relevant:
- a specification of the experimental system investigated (eg cell line, species name).
- the assay(s) and method(s) used to carry out the reported observations and measurements.
- an explicit mention of the biological and chemical entity(ies) that are being measured.
- an explicit mention of the biological and chemical entity(ies) that are altered/varied/perturbed in a controlled manner.
- the exact sample size (n) for each experimental group/condition, given as a number, not a range;
- a description of the sample collection allowing the reader to understand whether the samples represent technical or biological replicates (including how many animals, litters, cultures, etc.).
- a statement of how many times the experiment shown was independently replicated in the laboratory.
- definitions of statistical methods and measures:
  - common tests, such as t-test (please specify whether paired vs. unpaired), simple χ2 tests, Wilcoxon and Mann-Whitney tests, can be unambiguously identified by name only, but more complex techniques should be described in the methods section;
  - are tests one-sided or two-sided?
  - are there adjustments for multiple comparisons?
  - exact statistical test results, e.g., P values = x but not P values < x;
  - definition of 'center values' as median or average;
  - definition of error bars as s.d. or s.e.m.

Please complete ALL of the questions below.
Select "Not Applicable" only when the requested information is not relevant for your study.

Materials

Newly Created Materials	Information included in the manuscript?	In which section is the information available? (Reagents and Tools Table, Materials and Methods, Figures, Data Availability Section)
New materials and reagents need to be available; do any restrictions apply?	Not Applicable	

Antibodies	Information included in the manuscript?	In which section is the information available? (Reagents and Tools Table, Materials and Methods, Figures, Data Availability Section)
For **antibodies** provide the following information:<br>- Commercial antibodies: RRID (if possible) or supplier name, catalogue number and or/clone number<br>- Non-commercial: RRID or citation	Yes	Reagents and Tools table

DNA and RNA sequences	Information included in the manuscript?	In which section is the information available? (Reagents and Tools Table, Materials and Methods, Figures, Data Availability Section)
**Short novel DNA or RNA including primers, probes**: provide the sequences.	Yes	Reagents and Tools table

Cell materials	Information included in the manuscript?	In which section is the information available? (Reagents and Tools Table, Materials and Methods, Figures, Data Availability Section)
**Cell lines:** Provide species information, strain. Provide accession number in repository **OR** supplier name, catalog number, clone number, and/**OR** RRID.	Yes	Materials and methods
**Primary cultures:** Provide species, strain, sex of origin, genetic modification status.	Not Applicable	
Report if the cell lines were recently **authenticated** (e.g., by STR profiling) and tested for mycoplasma contamination.	Yes	Materials and methods

Experimental animals	Information included in the manuscript?	In which section is the information available? (Reagents and Tools Table, Materials and Methods, Figures, Data Availability Section)
**Laboratory animals or Model organisms:** Provide species, strain, sex, age, genetic modification status. Provide accession number in repository **OR** supplier name, catalog number, clone number, **OR** RRID.	Yes	Materials and methods
**Animal observed in or captured from the field:** Provide species, sex, and age where possible.	Not Applicable	
Please detail **housing and husbandry conditions**.	Not Applicable	

Plants and microbes	Information included in the manuscript?	In which section is the information available? (Reagents and Tools Table, Materials and Methods, Figures, Data Availability Section)
**Plants:** provide species and strain, ecotype and cultivar where relevant, unique accession number if available, and source (including location for collected wild specimens).	Not Applicable	
**Microbes:** provide species and strain, unique accession number if available, and source.	Not Applicable	

Human research participants	Information included in the manuscript?	In which section is the information available? (Reagents and Tools Table, Materials and Methods, Figures, Data Availability Section)
If collected and within the bounds of privacy constraints report on age, sex and gender or ethnicity for all study participants.	Not Applicable	

Core facilities	Information included in the manuscript?	In which section is the information available? (Reagents and Tools Table, Materials and Methods, Figures, Data Availability Section)
If your work benefited from core facilities, was their service mentioned in the acknowledgments section?	Yes	Acknowledgements

Design

Study protocol	Information included in the manuscript?	In which section is the information available? (Reagents and Tools Table, Materials and Methods, Figures, Data Availability Section)
If study protocol has been **pre-registered, provide DOI in the manuscript**. For clinical trials, provide the trial registration number **OR** cite DOI.	Not Applicable	
Report the **clinical trial registration number** (at ClinicalTrials.gov or equivalent), where applicable.	Not Applicable	

Laboratory protocol	Information included in the manuscript?	In which section is the information available? (Reagents and Tools Table, Materials and Methods, Figures, Data Availability Section)
Provide DOI OR other citation details if **external detailed step-by-step protocols** are available.	Not Applicable	

Experimental study design and statistics	Information included in the manuscript?	In which section is the information available? (Reagents and Tools Table, Materials and Methods, Figures, Data Availability Section)
Include a statement about **sample size** estimate even if no statistical methods were used.	Not Applicable	
Were any steps taken to minimize the effects of subjective bias when allocating animals/samples to treatment (e.g. **randomization procedure**)? If yes, have they been described?	Yes	Relevant figure legends
Include a statement about **blinding** even if no blinding was done.	Yes	Relevant figure legends
Describe **inclusion/exclusion criteria** if samples or animals were excluded from the analysis. Were the criteria pre-established?<br><br>If sample or data points were omitted from analysis, report if this was due to attrition or intentional exclusion and provide justification.	Not Applicable	
For every figure, are **statistical tests** justified as appropriate? Do the data meet the assumptions of the tests (e.g., normal distribution)? Describe any methods used to assess it. Is there an estimate of variation within each group of data? Is the variance similar between the groups that are being statistically compared?	Yes	Materials and methods

Sample definition and in-laboratory replication	Information included in the manuscript?	In which section is the information available? (Reagents and Tools Table, Materials and Methods, Figures, Data Availability Section)
In the figure legends: state number of times the experiment was **replicated** in laboratory.	Yes	Figure Legends
In the figure legends: define whether data describe **technical or biological replicates**.	Yes	Figure Legends

Ethics

Ethics	Information included in the manuscript?	In which section is the information available? (Reagents and Tools Table, Materials and Methods, Figures, Data Availability Section)
Studies involving **human participants**: State details of **authority granting ethics approval** (IRB or equivalent committee(s), provide reference number for approval.	Not Applicable	
Studies involving **human participants**: Include a statement confirming that **informed consent** was obtained from all subjects and that the experiments conformed to the principles set out in the WMA Declaration of Helsinki and the Department of Health and Human Services Belmont Report.	Not Applicable	
Studies involving **human participants:** For publication of **patient photos**, include a statement confirming that consent to publish was obtained.	Not Applicable	
Studies involving experimental **animals**: State details of **authority granting ethics approval** (IRB or equivalent committee(s), provide reference number for approval. Include a statement of compliance with ethical regulations.	Yes	Materials and Methods
Studies involving **specimen and field samples:** State if relevant **permits** obtained, provide details of authority approving study; if none were required, explain why.	Not Applicable	

Dual Use Research of Concern (DURC)	Information included in the manuscript?	In which section is the information available? (Reagents and Tools Table, Materials and Methods, Figures, Data Availability Section)
Could your study fall under dual use research restrictions? Please check biosecurity documents and list of **select agents and toxins** (CDC): https://www.selectagents.gov/sat/list.htm	Not Applicable	
If you used a select agent, is the security level of the lab appropriate and reported in the manuscript?	Not Applicable	
If a study is subject to dual use research of concern regulations, is the name of the **authority granting approval and reference number** for the regulatory approval provided in the manuscript?	Not Applicable	

Reporting

The MDAR framework recommends adoption of discipline-specific guidelines, established and endorsed through community initiatives. Journals have their own policy about requiring specific guidelines and recommendations to complement MDAR.

Adherence to community standards	Information included in the manuscript?	In which section is the information available? (Reagents and Tools Table, Materials and Methods, Figures, Data Availability Section)
State if relevant guidelines or checklists (e.g., **ICMJE, MIBBI, ARRIVE, PRISMA**) have been followed or provided.	Not Applicable	
For **tumor marker prognostic studies,** we recommend that you follow the **REMARK** reporting guidelines (see link list at top right). See author guidelines, under 'Reporting Guidelines'. Please confirm you have followed these guidelines.	Not Applicable	
For **phase II and III randomized controlled trials**, please refer to the **CONSORT** flow diagram (see link list at top right) and submit the CONSORT checklist (see link list at top right) with your submission. See author guidelines, under 'Reporting Guidelines'. Please confirm you have submitted this list.	Not Applicable	

Data Availability

Data availability	Information included in the manuscript?	In which section is the information available? (Reagents and Tools Table, Materials and Methods, Figures, Data Availability Section)
Have **primary datasets** been deposited according to the journal's guidelines (see 'Data Deposition' section) and the respective accession numbers provided in the Data Availability Section?	Yes	Data Availability section
Were **human clinical and genomic datasets** deposited in a public access-controlled repository in accordance to ethical obligations to the patients and to the applicable consent agreement?	Not Applicable	
Are **computational models** that are central and integral to a study available without restrictions in a machine-readable form? Were the relevant accession numbers or links provided?	Not Applicable	
If publicly available data were reused, provide the respective **data citations in the reference list**.	Not Applicable	
